# The complexity landscape of viral genomes

**DOI:** 10.1093/gigascience/giac079

**Published:** 2022-08-11

**Authors:** Jorge Miguel Silva, Diogo Pratas, Tânia Caetano, Sérgio Matos

**Affiliations:** Institute of Electronics and Informatics Engineering of Aveiro, University of Aveiro, Campus Universitário de Santiago, 3810-193 Aveiro, Portugal; Institute of Electronics and Informatics Engineering of Aveiro, University of Aveiro, Campus Universitário de Santiago, 3810-193 Aveiro, Portugal; Department of Electronics Telecommunications and Informatics, University of Aveiro, Campus Universitario de Santiago, 3810-193 Aveiro, Portugal; Department of Virology, University of Helsinki, Haartmaninkatu 3, 00014 Helsinki, Finland; Department of Biology, University of Aveiro, Campus Universitario de Santiago, 3810-193 Aveiro, Portugal; Institute of Electronics and Informatics Engineering of Aveiro, University of Aveiro, Campus Universitário de Santiago, 3810-193 Aveiro, Portugal; Department of Electronics Telecommunications and Informatics, University of Aveiro, Campus Universitario de Santiago, 3810-193 Aveiro, Portugal

**Keywords:** viruses, genomics, sequence analysis, data compression, cladograms, viral classification, algorithmic information theory

## Abstract

**Background:**

Viruses are among the shortest yet highly abundant species that harbor minimal instructions to infect cells, adapt, multiply, and exist. However, with the current substantial availability of viral genome sequences, the scientific repertory lacks a complexity landscape that automatically enlights viral genomes’ organization, relation, and fundamental characteristics.

**Results:**

This work provides a comprehensive landscape of the viral genome’s complexity (or quantity of information), identifying the most redundant and complex groups regarding their genome sequence while providing their distribution and characteristics at a large and local scale. Moreover, we identify and quantify inverted repeats abundance in viral genomes. For this purpose, we measure the sequence complexity of each available viral genome using data compression, demonstrating that adequate data compressors can efficiently quantify the complexity of viral genome sequences, including subsequences better represented by algorithmic sources (e.g., inverted repeats). Using a state-of-the-art genomic compressor on an extensive viral genomes database, we show that double-stranded DNA viruses are, on average, the most redundant viruses while single-stranded DNA viruses are the least. Contrarily, double-stranded RNA viruses show a lower redundancy relative to single-stranded RNA. Furthermore, we extend the ability of data compressors to quantify local complexity (or information content) in viral genomes using complexity profiles, unprecedently providing a direct complexity analysis of human herpesviruses. We also conceive a features-based classification methodology that can accurately distinguish viral genomes at different taxonomic levels without direct comparisons between sequences. This methodology combines data compression with simple measures such as GC-content percentage and sequence length, followed by machine learning classifiers.

**Conclusions:**

This article presents methodologies and findings that are highly relevant for understanding the patterns of similarity and singularity between viral groups, opening new frontiers for studying viral genomes’ organization while depicting the complexity trends and classification components of these genomes at different taxonomic levels. The whole study is supported by an extensive website (https://asilab.github.io/canvas/) for comprehending the viral genome characterization using dynamic and interactive approaches.

Key points

- We provide a comprehensive landscape of viral genomes' complexity.
- We demonstrate that data compressors can efficiently quantify the complexity of viral genome sequences, including subsequences better represented by algorithmic sources.
- We identify and quantify inverted repeats abundance in viral genomes.
- We use minimal bidirectional complexity profiles as local measures of the viral genome.
- We present an in-depth complexity analysis of the human herpesviruses.
- We show that the viral genome redundancy, GC-content, and size are efficient features to accurately distinguish between viral genomes at different taxonomic levels.
- Our work opens new frontiers for studying viral genomes’ complexity while depicting complexity trends in viral genomes.

## Introduction

Viruses are a strong driving force of life and evolution. They are the shortest and most abundant life realm, estimated at around 10^31^ particles [1]. Likewise, viruses occupy almost every ecosystem [2–4] and infect all types of life forms [5,6].

Viruses depend on the host’s cell for replication. This dependence has forced viruses to interact with cellular pathways to successfully hijack and customize the host cell machinery for viral production. This interaction generated a long-standing effect of adaptation and counteradaptation between host and viruses for gene expression and nucleic acid synthesis. Furthermore, during their replication, viruses can perform horizontal gene transfer, which increases the host species’ genetic diversity analogously to the process of sexual reproduction [7].

Despite the significant impact that viruses have on the evolution of living beings and the ecosystem, our understanding of viruses is still relatively limited compared with other realms of life. In particular, the complexity landscape of viruses is unknown. For example, what are the most redundant and complex viral DNA/RNA sequences? Which viruses contain more genomic inversions? How does the complexity distribution of viruses describe their morphology and behavior? What can be uncovered by analyzing the complexity of the viral genomes regarding viral processes? Moreover, is the information uncovered shared between the same viral groups? By studying the complexity of viral sequences and performing information quantification, one might be able to answer some of these questions.

Complexity analysis of the genome sequences is not new and is frequently performed by data compressors, which serve as an upper bound to Kolmogorov complexity. Many examples of these studies appeared after creating the first compressor for DNA sequences [8]. Specifically, data compression has been used to detect repeated sequences in the *Plasmodium falciparum* DNA, and observed patterns were related to large-scale chromosomal organization and gene expression control [8]. The XMAligner tool [9] was created for pairwise genome local alignment, which considers a pair of nucleotides from 2 sequences related if their mutual information in context is significant. To measure the information content of nucleotides in sequences, they used a lossless compression method. Graph compression was used for comparing large biological networks [10]. This method was done by compressing the original network structure and then measuring the similarity of the 2 networks using the compression ratio of the concatenated networks. The method was applied to several organisms, showing an efficient capability to measure the similarities between metabolic networks. Data compression was used to approximate the Kolmogorov complexity and applied to data derived from sequence alignment data [11]. This process identified a novel way of predicting 3 different aspects of protein structure: secondary structures, interresidue contacts, and the dynamics of switching between different protein states. An analysis of the complexity of different DNA genomes was performed, demonstrating various evolution-related findings linked with complexity, notably that archaea have a higher relative complexity than bacteria and eukaryotes on a global scale. Furthermore, viruses have the most complex sequences according to their size [12]. Metagenomic composition analysis of a sedimentary ancient DNA sample was performed using relative compression of whole-genome sequences [13]. The results showed that several viruses and bacteria expressed high levels of similarity relative to the samples. Finally, an alignment-free tool was created to accurately find genomic rearrangements of DNA sequences following previous studies, which took alignment-based approaches or performed fluorescence in situ hybridization (FISH) [14].

Given the applicability of compression methods in the analysis of genomic sequences and intending to better understand viruses, in this article, we perform an extensive complexity analysis of the viral world through the automatic computational analysis of its genome complexity and associated characteristics. Specifically, we use a genomic compressor to analyze the complexity across viral taxonomies and quantify the algorithmic information embedded in viral genome sequences better represented by small programs. Several questions arise when addressing this problem: How much information is present in a viral genome? What is the best way to quantify the information in a viral genome? What type of information can we retrieve from analyzing the complexity of the viral genome? We use unsupervised probabilistic and algorithmic information quantification of viral genomes to answer these questions. We use a high-quality database using the NCBI reference database with 12,168 complete reference genomes from 9,605 viral taxa.

Since studying the complexity of a DNA/RNA sequence requires efficient data compressors that take into account the probabilistic and algorithmic characteristics of the data, we compared several state-of-the-art genomic data compressors and another approximation of the Kolmogorov complexity besides data compression. This comparison was made to evaluate their ability to detect inverted repeats (IRs) with increasing levels of mutations. The results show that GeCo3 could detect and compress IRs, unlike other programs, using appropriate computational resources.

Consequently, GeCo3 was used to analyze viruses’ complexity and overall abundance of inverted repeats and construct cladograms. The results of our study show several insights into patterns between the complexity and viral groups and that these measurements can perform viral genome authentication and classification with high accuracy without directly comparing the sequences but instead using the individual features.

The following section describes the article’s background and related work. A description of the methods follows and the results obtained. Finally, we discuss the significant results obtained, draw conclusions, and point out possible future work lines.

## Background

This article shows that the efficient use of specific data compressors to quantify data complexity (Kolmogorov complexity) profoundly impacts viral genomes identification, classification, and organization. For introducing several concepts, this section provides an overview of the viral nature, Kolmogorov complexity and data compression, and the role of inverted repeats in the genome sequence.

### Viruses' microbiology

Viruses are submicroscopic biological infectious agents that require living cells of an organism to be active for replication [15] (for more information regarding viral morphology and genome, see the supplementary material of this article).

They have a vast size variation, ranging from around 10 nm with small genomes to viruses with similar dimensions and genome sizes to bacteria and archaea [16, 17]. These viruses are called giant viruses and contain many unique genes currently not found in other life forms.

There can also be hybrid viruses [18], making it difficult to identify species [19]. There are several possible combinations for the creation of a hybrid virus. One possible way is the infection of a host’s cell by 2 or more related viruses and consequential exchange of sequences between viruses. The result is the creation of a new variant derived from the parental genomes. Another possible way is the recombination of RNA viral genomes with the host’s RNA. Finally, there is evidence that small DNA viruses could have been created by recombination events between RNA viruses and DNA plasmids [18].

Although the origin of viruses is still uncertain, they play an essential role in the evolution of living organisms since they are horizontal gene transfer vehicles. This biological phenomenon increases genetic diversity. Furthermore, it occasionally allows viral genetic material to integrate into the host genomes, transferred vertically to its offspring. This property is so preponderant in evolution that the origin of the eukaryotic nucleus might be related to this process [20–22].

Additionally, viral genomic integration allows us to infer the evolutionary distance between hosts by observing the shared virus integrated into their genomes. For instance, in humans, viruses frequently establish persisting infections [23] and imprint their genetic material in the tissues throughout life, displaying phylogeography patterns. These can be used as markers to understand the human population history and migrations better and provide new insights into unidentified individuals’ origins on both global and local scales [24]. In this respect, the JC polyomavirus is one of the most comprehensively studied viruses. Its genotype-specific global spread has been suggested to indicate the origins of modern [25] and ancient humans [26–28]. Furthermore, a worldwide study supported the co-dispersal of this virus with major human migratory routes and its co-divergence with human mitochondrial and nuclear markers [29].

Thus, computer analysis of viral and host DNA sequences is fundamental to understanding the evolutionary relationships between different viruses and their hosts, identifying modern viruses’ ancestors, and better understanding their behavior and function. Also, the genomic sequences encode the production of proteins and their high-dimensional folding structure [30,31]. Therefore, the direct study of viral genome sequences also develops knowledge of the viral mechanism of protein formation and assembly.

### Inverted repeats

IRs are nucleotide sequences with a downstream reverse complement copy, causing a self-complementary base-pairing region [32]. Consequently, IRs usually fold into different secondary structures (hairpin- and cruciform-like structures, pseudoknots) that participate or interfere in many cellular processes in all forms of life, including DNA replication [33,34]. Due to these traits, IRs play an essential role in genome instability [35], contributing to mutability. This mutability can create diseases in the short term [36] but across long periods leads to cellular evolution and genetic diversity [37]. In many viruses, IRs in pseudoknots are involved in ribosomal frameshifting. This translational mechanism allows the production of different proteins encoded by overlapping open reading frames (ORFs) of the same messenger RNA (mRNA) [38, 39]. This feature allows them to encode a more significant amount of genetic information in small genomes and constitutes another level of gene regulation [40].

The genomes of some viruses, such as parvovirus, are flanked by inverted terminal repeats (ITRs) that form hairpin structures functioning as a duplex origin of replication sequence [33, 41]. Therefore, these ITRs contain most of the *cis*-acting information needed for viral replication and viral packaging [41]. In adeno-associated viruses, ITRs are essential for intermolecular recombination and circularization of genomes [42]. IRs can also function as termination transcription signals, especially in giant viruses [43, 44].

### Kolmogorov complexity and data compression

Solomonoff, Kolmogorov, and Chaitin [45–48] described the notion of data complexity by showing that there is at least 1 minimal algorithm among all the algorithms that decode strings from their codes. For all strings, this algorithm allows codes as short as any other, up to an additive constant that depends only on the strings themselves. Concretely, algorithmic information is a measure that quantifies the information of a string *x* by determining its complexity *K*(*x*) by

(1) \begin{equation*}
K(x) := \min _p\lbrace l(p):U(p)=x\rbrace , \end{equation*}

where *K*(*s*) is defined by a shortest length *l* of a binary program *p* that computes the string *x* on a universal Turing machine *U* and halts [47]. This notion that the complexity of a string can be defined as the length of a shortest binary program that outputs that string was universally adopted and is the standard to perform information quantification. It differs from Shannon’s entropy because it recognizes that the source creates structures that follow algorithmic schemes [49, 50], rather than regarding the machine as generating symbols from a probabilistic function.

While the Kolmogorov complexity is noncomputable, it can be approximated with programs for such purpose. A possible approximation is the coding theorem method (CTM) [51] and its improved version, the block decomposition method (BDM) [52], which approximate local estimations of algorithmic complexity, providing a closer relationship to the algorithmic nature. This approximation decomposes the quantification of complexity for segmented regions using small Turing machines [51]. For modeling the statistical nature, such as noise, it commutes into a Shannon entropy quantification. This approach has shown encouraging results for many distinct purposes [53–55]. However, it has also shown underestimation issues related to side information [56].

The classical approximation of the Kolmogorov complexity is performed using data compressors with probabilistic and algorithmic schemes [57]. Data compressors are a natural solution to measure complexity since, with the appropriate decoder, the bitstream produced by a lossless compression algorithm allows the reconstruction of the original data and, therefore, can be seen as an upper bound of the algorithmic complexity of the sequence. For a definition of safe approximation, see Bloem et al. [58].

In genomics, sequences can be codified as messages using a 4-symbol alphabet (Σ = {*A, C, G, T*} for DNA sequences and Σ = {*A, C, G, U*} for RNA sequences). These messages contain instructions for survival and replication of the organism, its morphology, and historical marks from previous generations [59]. Initially, genomic sequences were compressed with general-purpose data compressors such as gzip [60], bzip2 [61], or LZMA [62]. However, this paradigm shifted toward using a specific compression algorithm after introducing BioCompress [63]. Genomic compressors can outperform general-purpose compressors since they are designed to consider specific genomic properties such as the presence of a high number of copies and substitutional mutations and multiple rearrangements, such as inverted repeats [64,65].

Given this advantage of using specific compressors for the compression of genomic data, several algorithms have emerged to model these genomic data behaviors [66]. Specifically, several algorithms have been created to model repetitions and inverted repetitions in the genome regions through simple bit encoding, dictionary approaches, and context modeling [67–77].

Currently, state-of-the-art compressors have different objectives, such as optimizing for compression strength or prioritizing a balance between compression speed and compression capability. Examples of the latter are NAF (Nucleotide Archival Format) [78, 79] and MBGC (Multiple Bacteria Genome Compressor) [80], which are more suitable for collections of data and frequently used by computational biologists. Compressors focused on compressibility at the expense of more computational resources, on the other hand, generally apply statistical and algorithmic model mixtures combined with arithmetic encoding. Among the best compressors regarding compression ratio performance for various genomic sequences, the best results are provided by cmix [81], XM [82], Jarvis [83], and Geco3 [84]. For additional information regarding data compressors’ compressibility capacity of genomic sequences, see Kryukov et al. [85]. Cmix [81] is a general-purpose lossless data compression program that optimizes compression ratio at the cost of high CPU/memory usage. It is based on PAQ compressors [86,87] but dramatically increases the amount of processing per input bit and computational memory. Current updates include LSTM (Long Short-Term Memory)–based models [88]. The XM compressor [82] uses 3 types of experts: repeat models, a low-order context model, and a short-memory context model. On the other hand, Jarvis [83] uses a competitive prediction model that estimates for each symbol the best class of models to be used. There are 2 classes of models: weighted context models and weighted stochastic repeat models, where both classes of models use specific subprograms to handle inverted repeats efficiently. Finally, GeCo3 [84], currently one of the best-performing reference-free data compressors, uses neural networks to improve upon the results of specific genomic models of GeCo2 [89]. Specifically, the neural networks are used in mixing multiple contexts and substitution-tolerant context models of GeCo2. Furthermore, GeCo3 has embedded subprograms capable of detecting genome-specific patterns, such as inverted repeats.

## Methods

This section describes the measures used in this article. Specifically, we first define information-based measures: the normalized block decomposition method (NBDM), the normalized compression (NC) with different subprograms, the normalized compression capacity (NCC), the difference between NCs, and the minimal bidirectional complexity profiles. Afterward, we define the GC-content and the compression benchmark performed. Finally, we describe the classification pipeline—specifically, the features and classifiers used and the metrics utilized for evaluating the model’s performance.

### Information-based measures

This section describes 2 approximations of the Kolmogorov complexity, one based on the decomposition of a string into blocks and their approximation based on the output of small Turing machines (BDM) and another based on data compression. The data compression approach was utilized to compute the NC and construct the minimal bidirectional complexity profiles. Therefore, in this subsection, we describe the NC, the minimal bidirectional complexity profiles, and the NBDM.

#### NBDM

A possible approximation of the Kolmogorov complexity is given by using small Turing machines (TMs), which approximate the components of a broader representation. The CTM uses the algorithmic probability between a string’s production frequency from a random program and its algorithmic complexity. The more frequent a string is, the lower its Kolmogorov complexity, and the lower frequency strings have, the higher Kolmogorov complexity is. The BDM increases the capability of a CTM, approximating local estimations of algorithmic information based on Solomonoff-Levin’s algorithmic probability theory. In practice, it approximates the algorithmic information, and when it loses accuracy, it approximates the Shannon entropy. Since in this article we use BDM to perform a comparison with the NC, we considered the normalization of the BDM (NBDM) according to Silva et al. [56]. In this case, the NBDM is computed as

(2) \begin{equation*}
\mathrm{NBDM}(x) = \frac{BDM(x)}{|x|\log _2{\mathrm{|\Sigma |}}} = \frac{BDM(x)}{2\times |x|}. \end{equation*}

where *x* is a string, *BDM*(*x*) is the BDM value of the string, |Σ| is the number of different elements in *x* (size of the alphabet), and |*x*| is the length of *x*. Since we have a 4-symbol alphabet (Σ = {*A, C, G, T*} for DNA sequences and Σ = {*A, C, G, U*} for RNA sequences), |Σ| = 4, log_2_(4) = 2. Although BDM has difficulty dealing with full-information quantification due to the block representability, it has proven to be a helpful tool for measuring and identifying data content similar to simple algorithms [56].

#### NC

An efficient compressor provides an upper-bound approximation for the Kolmogorov complexity. Specifically, *K*(*x*) < *C*(*x*) ≤ |*x*|log_2_|Σ|, where *K*(*x*) is the Kolmogorov complexity of the string *x* in bits, *C*(*x*) is the compressed size of *x* in bits, and |*x*| is the length of string *x*. This relation neglects the constant that asymptotically becomes irrelevant. Usually, an efficient data compressor is a program that approximates both probabilistic and algorithmic sources using affordable computational resources (time and memory). Although the algorithmic nature may be more complex to model, data compressors can have embedded subprograms to handle this nature. The normalized version, known as the NC, is defined by

(3) \begin{equation*}
\mathrm{NC}(x) = \frac{C(x)}{|x|\log _2{\mathrm{|\Sigma |}}} = \frac{C(x)}{2\times |x|}. \end{equation*}

Given the normalization, the NC enables to compare the proportions of information contained in the strings independently from their sizes [12]. If the compressor is efficient, then it can approximate the quantity of probabilistic-algorithmic information in data using affordable computational resources. In our work, to determine the NC, we made use of the state-of-the-art genome compressor GeCo3 [84], with the level 16 that yielded the best average results (benchmark provided in the Results section).

Besides the computation of the NC using the standard configuration of this model, we also computed the NC using GeCo3 with 3 subprogram configurations. These subprogram configurations address the use or absence of inverted repetitions, namely:

- *IR*_0_ → uses the regular context model without IR detection,
- *IR*_1_ → uses IR detection simultaneously with the regular context model, and
- *IR*_2_ → uses IR detection subprogram without regular context models.

There was a need to determine the sequences with the highest NCC in some cases. When the compressor was only using the subprogram *IR*_2_, NCC was computed as $NCC_{IR_{2}}(x)= 1 - NC_{IR_{2}}$. Only positive values were considered to filter computations where the compressor could not compress the sequence sufficiently. Another measure used to quantify inverted repeats was the difference between $NC_{IR_{0}}$ and $NC_{IR_{1}}$.

#### Minimal bidirectional complexity profiles

A complexity profile is a numerical sequence describing for each symbol (*x_i_*) of a sequence *x* the number of bits required for its compression, assuming a causal order [90]. A minimal bidirectional complexity, *B*(*x*), profile assumes the minimal representation of compressing the sequences using both directions independently, namely, $\overrightarrow{C}(x_i)$ as from the beginning to the end of the sequence and $\overleftarrow{C}(x_i)$ as from the end to the beginning [91]. Accordingly, these profiles are defined as

(4) \begin{equation*}
B(x_i)=\min \lbrace \overrightarrow{C}(x_i), \overleftarrow{C}(x_i)\rbrace . \end{equation*}

The construction of these profiles follows a pipeline formed of many transformations, including reversing, segmenting, inverting, and using specific low-pass filters after data compression to achieve better visualization. For computing these profiles, we use the GTO toolkit [92].

The generation of these profiles is robust to localize specific features in the sequences, namely, low- and high-complexity sequences, inverted repeat regions, and duplications, among others.

### Other measures

The 2 other measures used to perform viral analysis and classification are the GC-content (GC) and the length of the viral genome |*x*|.

GC-content (GC) represents the proportion of guanine (G) and cytosine (C) bases out the quaternary alphabet (Σ = {*A, C, G, T*/*U*}). This includes thymine (T) in DNA and uracil (U) in RNA. The GC percentage is given by the number of cytosine (C) and guanine (G) bases in a viral genome *x* with length |*x*| according to

(5) \begin{equation*}
\mathcal {GC}(x) = \frac{100}{|x|} \sum _{i=1}^{|x|}\mathcal {N}(x_i || x_i \in \Xi ), \end{equation*}

where *x_i_* is each symbol of *x* (assuming causal order), Ξ is a subset of the genomic alphabet containing the symbols {*G, C*}, and $\mathcal {N}$ is the program that counts the numbers of symbols in Ξ.

GC-content is variable between different organisms and correlates with the organism’s life-history traits, genome size [93], and GC-biased gene conversion [94]. Furthermore, in RNA viruses, excess C to U substitutions accounted for 11–14% of the sequence variability of viruses, indicating that a decrease in GC-content is a potent driver of RNA viruses’ diversification and longer-term evolution [95]. As such, this measure helps perform viral classification.

On the other hand, it was shown that the number of base stackings (typical arrangement of nucleobases found in the 3-dimensional structure of nucleic acids) is one of the most critical elements contributing to the thermal stability of double-stranded nucleic acids. Furthermore, due to the relative locations of exocyclic groups, GC pairings have higher stacking energy than AT or AU pairs [96]. This energy accumulation in the GC pair in an organism’s genome makes the DNA more prone to mutation. Thus, over time, a species tends to decrease its GC-content to become more stable [97], giving us further information regarding viral characterization.

### Data description

The data set is composed of 12,163 complete reference genomes from 9,605 viral taxa retrieved from the NCBI database on 22 January 2021.^1^ The download was performed in a custom manner to retrieve the taxonomic ID, host, and geolocation of each reference genome. The metadata header was removed from each sequence using the GTO toolkit [92], where any nucleotide outside the quaternary alphabet {*A, C, G, T*/*U*}, was replaced by a random nucleotide from the quaternary alphabet. Notice that the sequences with symbols outside the alphabet are scarce. Finally, the type of genome and the taxonomic description of each sequence were retrieved using Entrez-direct [98].

Then, the retrieved NCBI sequences were filtered to remove possibly contaminated or poorly sequenced sequences. First, using the taxonomic metadata, sequences that did not hold complete taxonomic information down to the genus rank and any sequences that maintained a taxonomic description of unclassified were removed. Second, we applied a filter to remove outlier sequences. Specifically, after computing all sequences’ length, GC-content, and normalized complexities, sequences whose measure fell outside μ ± 3 × σ (approximately 0.03% of all sequences) of any measure were removed. A total of 182 sequences were removed since they most likely have errors in the assembly process or contamination. After filtering, we kept 6,091 of the initial 12,163 sequences.

### Data compressors and level selection benchmark

First, we tested cmix and GeCo3 regarding compression ratio and time required per sequence compression. This was followed by selection of a total of 19 levels of models in GeCo3 to determine the best level configuration to compress the viral sequences. These levels correspond to the default 13 levels of the GeCo3 compressor and 6 others built for this task. The list of the levels used is shown in Supplementary Table S1, and the description of parameters can be found in Supplementary Table S2. The 13 default levels of the compressor have increasingly higher complexity and take longer to run since they use higher-context models. Therefore, since the first and lightest level performed best, the other 6 custom-built levels were also built with lightweight models.

### Classification

We tested several machine learning algorithms to perform the genomic and taxonomic classification task—namely, the classifiers used were linear discriminant analysis (LDA) [99], Gaussian naive Bayes (GNB) [100], K-nearest neighbors (KNN) [101], support vector machine (SVM) [102], and XGBoost classifier (XGB) [103].

Linear discriminant analysis is a generalization of Fisher’s linear discriminant, a method used in statistics and other fields to find a linear combination of features that separates classes of objects. The resulting combination can be used as a linear classifier [99]. Gaussian naive Bayes is defined as a supervised machine learning classification algorithm based on the Bayes theorem following Gaussian normal distribution [100]. K-nearest neighbors is another approach to data classification, taking distance functions into account and performing classification predictions based on the majority vote of its neighbors [101]. Support vector machines are supervised learning models with associated learning algorithms that construct a hyperplane in a high-dimensional space using data and perform classification [102]. Finally, XGBoost [103] is an efficient open-source implementation of the gradient boosted trees algorithm. Gradient boosting is a supervised learning algorithm that predicts a target variable by combining the estimates of a set of simpler models. Specifically, new models are created that predict the residuals or errors of prior models and then added together to make the final prediction. This task uses a gradient descent algorithm to minimize the loss when adding new models. XGBoost can use this method in both regression and classification predictive modeling problems.

The accuracy and weighted F1-score were used to select and evaluate the classification performance of the measures. Accuracy is the proportion between correct classifications and the total number of cases examined, while the F1-score is computed using the precision and recall of the test. We utilized the weighted version of the F1-score due to the presence of imbalanced classes.

For comparison of the obtained results, we assessed the outcomes obtained using a random classifier. For that purpose, for each task, we determined the probability of a random sequence being correctly classified (*p_hit_*) as

(6) \begin{equation*}
p_{hit} = \sum _{i=0}^{n} [ p(c_i ) * p_{correct}(c_i) ], \end{equation*}

where *p*(*c_i_*) is the probability of each class, determined as

\begin{equation*} p(c_i )=\frac{|samples_{class}|}{|samples_{total}|}. \end{equation*}

On the other hand, *p_correct_*(*c_i_*) is the probability of that class being correctly classified. In the case of a random classifier,

\begin{equation*} p_{correct}(c_i) = \frac{1}{|classes|}. \end{equation*}

## Results

The results reported in this article can be computed using the minimal characteristics described in the supplementary subsection entitled Software and Hardware recommendations and using the procedures described in the supplementary subsection entitled Reproducibility. The following subsections describe the data, the compression level selection benchmark, the synthetic sequence benchmark, the viral genome analysis and cladograms, and the viral classification application.

### Data compressors and level selection benchmark results

Viral genomes have specific characteristics, for example, short length, high average complexity, and specific structures, that require the proper optimization of the data compressor to provide higher modeling adaptability and efficiency. Cmix and GeCo3 are state-of-the-art genomic compressors. To assess the viability of each compressor, we tested their computational time and NC values on a small sample consisting of 8 medium-size viral genomes. The results, presented in Supplementary Figure S2, show that the compression ratio of GeCo3 is, on average, slightly better, with a much more reasonable computational time (on average, 3 orders of magnitude faster than cmix). As such, for the remaining of the work, we consider the GeCo3 compressor.

On the other hand, GeCo3 contains many types of compression levels [84]. Therefore, we applied GeCo3 to each viral genome from the data set using 19 different levels and computed its NC.

**Figure 1:**
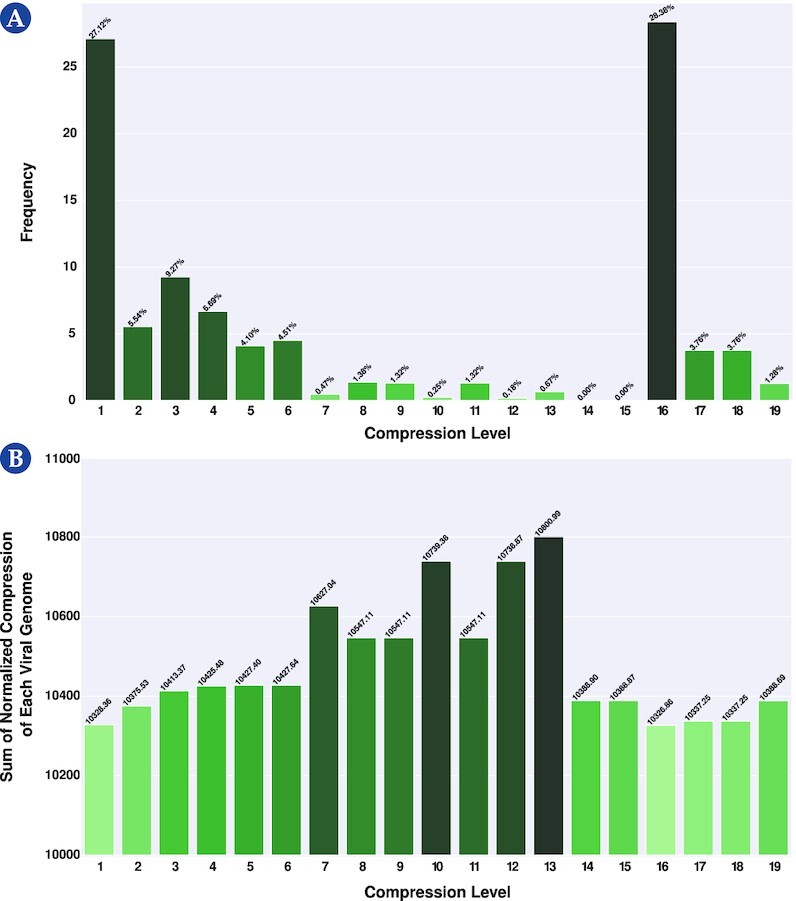
: Selection of a level for GeCo3 from a pool of 19 levels. (A) Frequency where each level provided the best NC results. (B) The sum for each level of the NC from the compression of all reference genomes. For better visualization, please visit the website https://asilab.github.io/canvas/.

We evaluated the frequency where each level yielded the lowest NC (provided the best compression for a given sequence; Fig. 1A) and determined the sum of the NC from the compression of all reference genomes for each model (Fig. 1B). Overall, we selected level 16 because it provided the lowest NC on average (28.38% as the best compression level) and the lowest NC sum from compressing all reference genomes. This level is constituted by a mixture using a neural network with the following models:

- Model 1 → context order of 1, alpha parameter of 1 (without inverted repeats), and gamma parameter of 0.7
- Model 2 → context order of 12, alpha parameter of 1/50 (with inverted repeats), and gamma parameter of 0.97

The chosen level is constituted by 2 models with a small and average context model. This configuration performed better because most viral genomes are small and compact, where a small genomic space usually separates repetitions and IRs. Therefore, the depth of the models is more adapted to provide higher efficiency to the average of the viral genomes than, for example, a higher-context model (higher than 13) that can perform marginally better in more extensive and repetitive sequences but that loses sensitivity in the average of the genomes.

### Synthetic sequence benchmark

**Figure 2:**
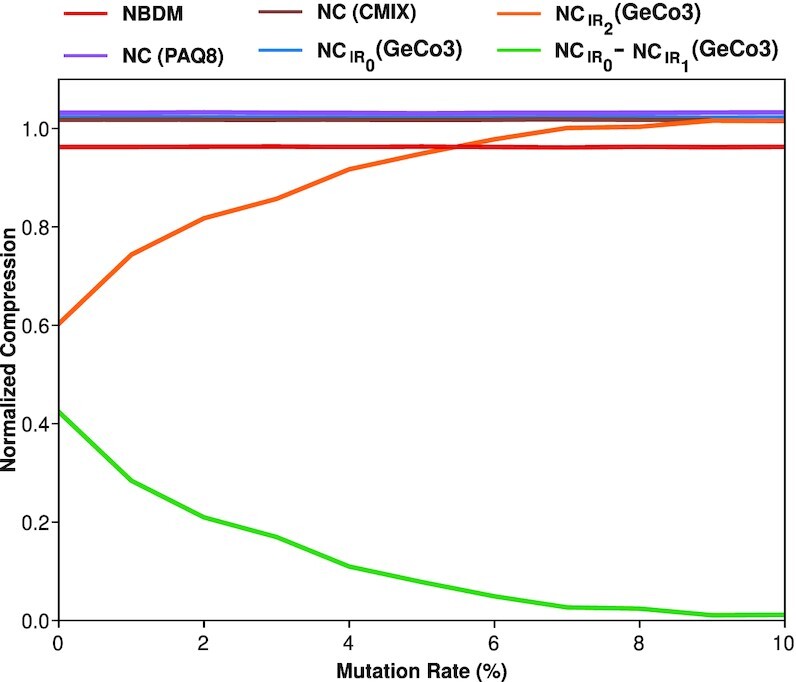
: Plot describing the variation of normalized compression (NC) and normalized block decomposition method (NBDM) with an increase of mutation rate of a sequence (0–10%). The NC was computed using the state-of-the-art genomic compressor (GeCo3 [84]) and a general-purpose compressor (PAQ8 [104]). The NBDM (red line), the NC value using cmix (brown line), and PAQ8 (purple line) are depicted. Furthermore, the GeCo3 compressor with (*IR*_2_) and without (*IR*_0_) the IR detection subprogram is shown with orange and blue lines, respectively. Finally, the green line shows the difference between $NC_{IR_{0}}-NC_{IR_{1}}$.

Viral genomes can contain IRs whose subsequences are better described using simple algorithmic approaches. To benchmark the capability of different programs to quantify IRs accurately, we created a genomic sequence of 10,000 nucleotides in which the last 5,000 were inverted repeats of the first 5,000. This size was chosen since the median size of the viral genomes is 9,836 bases, which is close to the total size of the synthetic sequence generated. This sequence was mutated incrementally from 0% to 10%, meaning that the number of IRs decreases with the increase of nucleotide substitutions. For each sequence, the NC was computed with (Fig. 2) (i) GeCo3, without and with the IR detection program (*IR*_0_ and *IR*_2_, respectively); (ii) PAQ8; and (iii) Cmix. Additionally, the NBDM was also computed as a more prone measure of algorithmic nature quantification. Results show that GeCo3 with the *IR*_2_ subprogram compresses the sequences better than the other programs since its NC is lower at a 0% mutational rate (Fig. 2). All other compressors (cmix and PAQ8) could not detect IRs and compress the sequence. Furthermore, NBDM also cannot detect the IRs because it provides the same high value across sequences with various mutation rates. It is also evident that GeCo3 with *IR*_2_ can detect IRs even in the presence of substantial mutations (5% of mutation) and takes into account different levels of nucleotide substitutions because it increases with the increase of the mutational rate (i.e., decrease of IRs). The difference between $NC_{IR_{0}}$ and $NC_{IR_{1}}$, both computed with GeCo3, was also analyzed. Its profile is inverse to the *IR*_2_ and confirms that nucleotide substitutions’ accumulation decreases the number of IRs in the sequence.

### Viral genome analysis and cladograms

**Figure 3:**
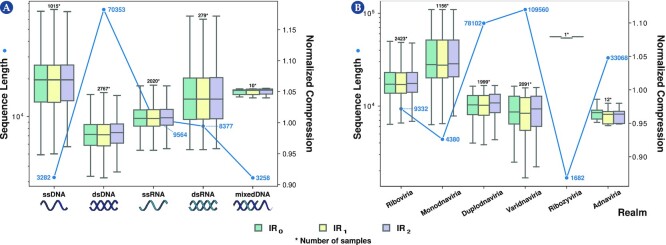
: Average normalized compression (ANC) and average sequence length per viral group. The values were obtained for genome type (A) and realm (B). To view all boxplots by groups of realm, kingdom, phylum, class, order, family, and genus, please visit the website https://asilab.github.io/canvas/.

The core of the viral genomes was analyzed in terms of complexity landscape, including the trends, singularities, and patterns for both the use or absence of IRs. The NC, using GeCo3, with *IR*_0_, *IR*_1_, and *IR*_2_ subprograms, was determined and the $NCC_{IR_2}$ was calculated. The outcome was interpreted according to the genome type or the taxonomic group, together with the average of their genome sizes (Fig. 3 and Supplementary Table S3). Notice that the NC enables to compare proportions of the absence of redundancy independently from the sizes of the genomes. This value is complementary to the normalized redundancy. Specifically, consider the redundancy (R) of a sequence *x* as *R*(*x*) = *log*_2_(*A*)|*x*| − *C*(*x*), where |*x*| is the length of the sequence, *A* is the cardinally of the sequences’ alphabet, and *C*(*x*) is the compressed size of *x* in bits, and the normalized redundancy (NR) as $NR(x) = 1 -\: (C(x)/(log_2(A)|x|))$.

#### Complexity landscape according to genome type

According to NCBI, the virus’s genomes herein analyzed are of 5 types: double-stranded DNA (dsDNA), single-stranded DNA (ssDNA), double-stranded RNA (dsRNA), single-stranded RNA (ssRNA), and mixed DNA. Results show that ssDNA, followed by mixed-DNA and dsRNA viruses, are the genomes with higher NC, whereas dsDNA genomes have the lowest (Fig. 3A; Supplementary Table S3). In general, smaller genomes are less complex and are more likely to contain fewer repeats and, hence, less redundancy, and the ssDNA, mixed-DNA, and dsRNA genomes have smaller average sequence lengths (3,282 bp, 3,258 bp, and 8,377 bp; Supplementary Table S3).

According to the NCC and the $NC_{IR_0} - NC_{IR_1}$ difference results, dsDNA and ssDNA have the most significant quantities of IRs than the other genome types. This can be due to ITRs present at the ends of some dsDNA viruses, such as adenovirus and ampullaviruses, and ssDNA virus as parvoviruses or other important IR structures that perform ribosomal frameshifting.

#### Complexity landscape according to taxonomic level

In complexity analysis of viral genomic sequences, when considering the realm taxonomic level (Fig. 3B), the lowest NC values were obtained for Adnaviria, Varidnaviria, and Duplodnaviria (Supplementary Tables S4 and S5). These results are consistent with the genomic grouping since they are composed exclusively of dsDNA viruses and have the highest sequence lengths. Thus, generally, an inverse correlation between genome size and NC was also observed as with the genome type analysis (Figs. 3A and B) and occurs across all taxonomic levels (Supplementary Table S5). However, within these 3 realms, Adnaviria has the lowest sequence length and presented a higher compressibility than Varidnaviria and Duplodnaviria, suggesting that the last are highly complex.

Regarding IRs, Adnaviria was the realm where the highest compression was obtained using the *IR*_2_ subprogram (highest rate of IRs; Supplementary Table S6). Consequently, its only recognized kingdom, Zilligvirae, has also one of the highest NCC values (Supplementary Table S6). Adnaviria is a realm constituted of mostly A-form dsDNA viruses, and the ends of their genomes contain ITRs [105]. A-form is proposed to be an adaptation allowing DNA survival under extreme conditions since their hosts are hyperthermophile and acidophile microorganisms from the archaea domain [105, 106]. The fact that Adnaviria presented the lowest NC might indicate that their genomes require redundancy to survive such extreme environments. The kingdom Trapavirae, belonging to the realm Monodnaviria, is also composed by dsDNA viruses that infect halophilic archaea. Together with kingdom Zilligvirae, Trapavirae presented the highest difference between IRs and standard compression (Supplementary Table S7). These results also support the fact that IRs can stabilize the DNA of viruses that exist in extreme environments. It has already been demonstrated that archaeal viruses with linear genomes use diverse solutions for protection and replication of the genome ends, such as including covalently closed hairpins and terminal IRs [107].

At the family level, Botourmiaviridae presented the highest complexity, followed by Alphasatellitidae and Tolecusatellitidae families (Supplementary Table S5). Botourmiaviridae is composed of ssRNA viruses that infect plants and filamentous fungi [108]. Curiously, plants and fungi have higher redundancy despite the lower redundancy of their pathogens. Alphasatellitidae and Tolecusatellitidae are families of satellite viruses that depend on the presence of another virus (helper viruses) to replicate their genomes. These satellite viruses have minimal genomes, making sense that they possess very low redundancy. Regarding IRs, Malacoherpesviridae, Herpesviridae, and Rudiviridae contained the highest $NC_{IR_0} - NC_{IR_1}$ difference (Supplementary Table S7). Malacoherpesviridae and Herpesviridae are dsDNA viruses evolutionarily close since they belong to the order Herpesvirales [109]. Malacoherpesviridae encompasses the genera *Aurivirus* and *Ostreavirus*, which infect molluscs. Herpesviridae are also known as herpesviruses and have reptiles, birds, and mammals as hosts. This family will be discussed in more detail in the following subsection. Rudiviridae is a family of viruses with linear dsDNA genomes that also infect archaea. The virus of these families is highly thermostable and can act as a template for site-selective and spatially controlled chemical modification. Furthermore, the 2 strands of the DNA are covalently linked at both ends of the genomes, which have long ITRs [110]. Again, these IRs could be an adaptation to stabilize the genome.

#### Complexity landscape of the family Herpesviridae

**Figure 4:**
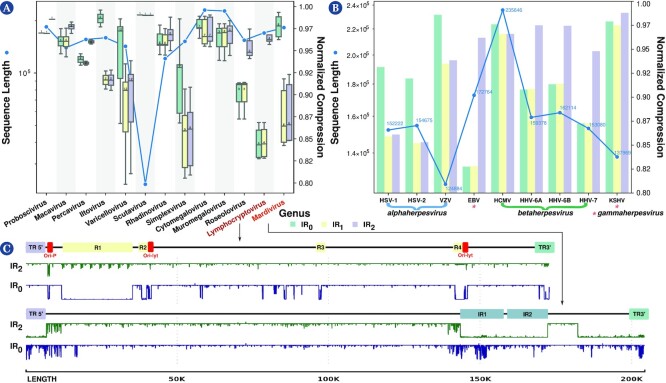
: Average normalized compression (ANC) and average sequence length per the genera of the Herpesviridae family (A) and for various human herpesviruses (B). In the boxplot where the genera of the Herpesviridae family are displayed, 2 genera were selected, one with a low level of inverted repeats (*Lymphocryptovirus*) and one with a high level (*Mardivirus*). Then, a representative reference sequence was selected (*Lymphocryptovirus*—human herpesvirus 4 or Epstein–Barr virus, NCBI Reference Sequence: $NC\_024450.1$; *Mardivirus*—Falconid herpesvirus 1 strain S-18, NCBI Reference Sequence: $NC\_009334.1$) and minimal bidirectional complexity profiles were created (C).

Here we analyzed the complexity landscape of the genera of the family Herpesviridae in more detail, and results show a significant variation between them (Fig. 4A). Mardivirus had the highest $NC_{IR_0}-NC_{IR_1}$ difference among all viruses, and only 3 other genera (out of 13) of herpesviruses were within the 10 highest differences list (Supplementary Table S7). Indeed, the genus *Mardivirus* had the highest compression, whereas the genus *Lymphocryptovirus* possessed very low compression with the *IR*_2_ subprogram. We performed the minimal bidirectional complexity profiles of 1 sequence of each virus to visualize their distribution of complexity locally (Fig. 4C). As we can see, human herpesvirus 4 (also known as Epstein–Barr virus [EBV]) has more internal repeats (Fig. 4C, *IR*_0_ profile) detected and fewer IRs (Fig. 4B; *IR*_2_ profile). The opposite occurs with the Falconid herpesvirus 1 strain S-18, where IRs are more prominent than internal repetitions. Furthermore, notice that these regions determined with compression profiles coincide with actual regions detected in the genome with other methods (Fig. 4C; first profile).

A particular group of family Herpesviridae are the human herpesviruses (HHVs). These viruses are involved in globally prevalent infections and cancers and characterized by lifelong persistence with reactivations that can potentially manifest life-threatening conditions [111]. Globally, the HHVs present a higher redundancy relative to other viruses (Fig. 4B). These viruses are divided into (i) the alpha-subfamily members, namely, herpes simplex virus types 1 and 2 (HSV-1 and HSV-2) and varicella-zoster virus (VZV); (ii) the beta-subfamily of human cytomegalovirus (HCMV) and human herpesviruses 6A, 6B, and 7 (HHV-6A, HHV-6B, and HHV-7); and (iii) the gamma-subfamily of EBV and Kaposi’s sarcoma–associated herpesvirus (KSHV). Specifically, EBV, one of the most potent cell transformation and growth-inducing viruses known, capable of *immortalizing* human B lymphocytes, contains a higher redundancy than the other HHVs (Fig. 4B). The other gamma-herpesvirus, KSHV, is the genome with the highest $NC_{IR_1}$ (Fig. 4B). Unlike the beta- and gamma-subfamilies, the alpha-subfamily is characterized by a substantial quantity of IRs, as suggested by the NCs with *IR*_1_ and *IR*_2_ configurations (Fig. 4B). The VZV has the shortest genome and the highest NC within this group. These differences might be justified by the different rates of evolution within these genomes [112]. Considering the beta-subfamily members, HCMV contains a small proportion of IRs while having a substantially high NC relative to other HHVs being analyzed. Since the HCMV has the largest genome, this was surprising because the NC typically has an inverse correlation with the genome size and the quantity of IRs. The other beta-subfamily members are the human herpesviruses 6A, 6B, and 7, which produced lower NCs (with *IR*_1_ and *IR*_2_ configurations) compared to the other HHVs, with a low quantity of IRs, an effect that their integrating function might favor. For instance, HHV-6A and 6B can integrate their genomes into the telomeres of latently infected cells [113,114]. Thus, their genomes contain subsequences similar to the human telomere regions that can be formed by internal nucleotide repetitions [115]. As such, these are sequences with very low complexity and, hence, highly compressible.

#### Alternative visualization methods of the viral complexity landscape

**Figure 5:**
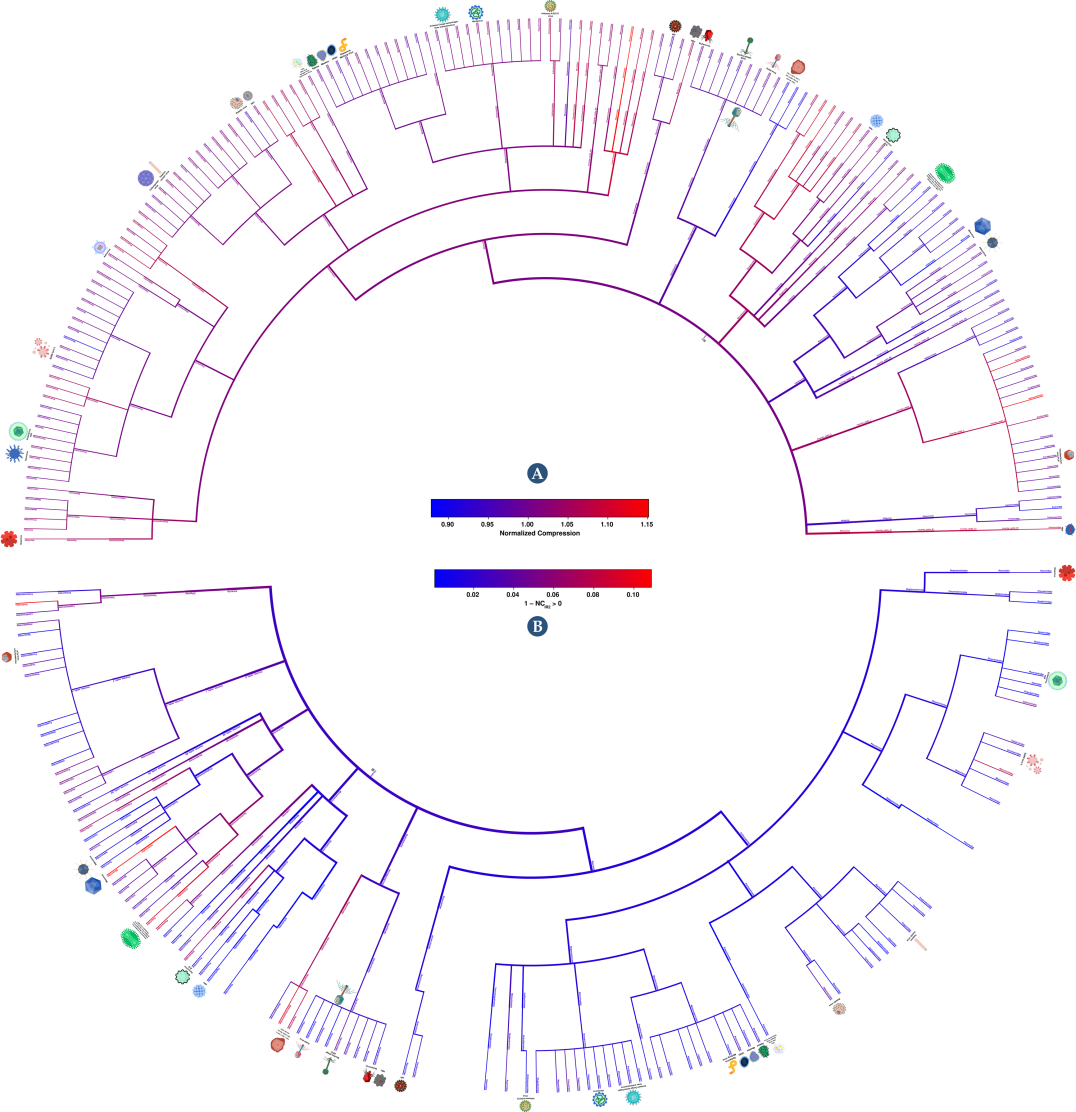
: Cladograms showing average normalized compression (NC) of each viral group (A) and the normalized compression capacity (NCC) (B). NCC results were obtained by $NCC= 1- NC_{IR_{2}} > 0$. The red color depicts the highest complexity and the blue the lowest. The first cladogram describes the NC of each taxonomic branch. Red color shows genomes with less redundancy and blue ones with more redundancy. On the other hand, the second cladogram depicts the prevalence of inverted repeats on each taxonomic branch. Red indicates branches with genomes with a high percentage of inverted repeats, whereas blue shows branches with a low percentage. For better visualization, please visit the website https://asilab.github.io/canvas/.

Cladograms were generated depicting the redundancy (NC; Fig. 5A) and the prevalence of inverted repeats (NCC; Fig. 5B) on each taxonomic branch. In addition, we performed the same analysis to portray the relation between inverted and internal repetitions (Supplementary Fig. S3). These cladograms show the broad picture of the regions with more complex and less redundant sequences, regions rich in inverted repeats, and regions with a higher prevalence of inverted repeats relative to standard repetitions in the genomes.

**Figure 6:**
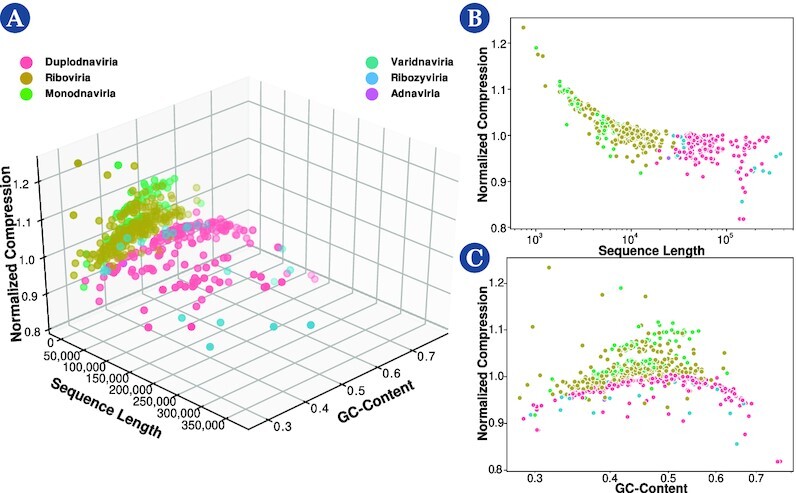
: Scatterplots of normalized compression versus sequence length and GC-content (A), scatterplots of normalized compression versus sequence length (B), and normalized compression versus GC-content (C).

Another way to analyze the results is by producing 3-dimensional scatterplots of randomly sampled values obtained from computing the features' sequence length (SL), NC, and GC-content (GC; Fig. 6A) or 2-dimensional scatterplots of their projections (Fig. 6B and C), both concerning a particular taxonomic level (herein realm). Analyzing the sequence length projections (Fig. 6B), it is evident that there is a logarithmic downtrend of the NC with the increase in sequence length. Thus, although longer sequences have, on average, greater complexity (absolute quantities), they have higher redundancy, which the data compressor takes advantage of to perform a better compression. On the other hand, the NC versus the GC-content displays a normal distribution around the 0.5 GC-mark, with higher complexities associated with similar frequency of occurrence of the 4 bases A, C, G, and T/U (Fig. 6C). This result also makes sense since, in principle, a well-distributed frequency of bases makes more complex sequences to compress. More importantly, the NC, GC, and SL seem to discriminate between different taxonomic groups (Fig. 6). As such, in the following section, we analyze the classification capability of these features.

### Viral classification

Although sequence alignment is essential for genomic analysis, the fact that pairwise and multiple alignment methods are often slow methods led to the popularization of fast alignment-free methods for sequence comparison. Most alignment-free methods are based on word frequencies for words of a fixed length or word-matching statistics. Others use the length of maximal word matches, and others rely on spaced-word matches (SpaM). These inexact word matches allow mismatches at certain predefined positions and can accurately estimate phylogenetic distances between DNA or protein sequences using a stochastic model of molecular evolution [116]. This approach has also been updated as the multiple spaced-word matches (multi-SpaM) method, which is based on multiple sequence comparison and maximum likelihood [117]. Regarding viral sequences, many studies were performed on alignment-free sequence comparison and classification. For instance, Garcia et al. [118] developed a dynamic programming algorithm for creating a classification tree using metagenome viruses. For the classification tree creation, *k*-mer profiles of each metagenome virus were created, and proportional similarity scores were generated and clustered. Using the JGI metagenomic and NCBI databases, the authors were able to identify the correct virus (including its parent in the classification tree) 82% of the time. Zhang et al. [119] created an alignment-free method that employed *k*-mers as genomic features for a large-scale comparison of complete viral genomes. After determining the optimal *k* for all 3,905 complete viral genomes, a dendrogram was created, which shows consistency with the viral Taxonomy of Viruses (ICTV) and the Baltimore classification of viruses. He et al. [120] proposed an alignment-free sequence comparison method for viral genomes based on the location correlation coefficient. When applied to the evolutionary analysis of the common human viruses, including severe acute respiratory syndrome coronavirus 2 (SARS-CoV-2), dengue virus, hepatitis B virus, and human rhinovirus, it achieves the same or even better results than alignment-based methods. Finally, Huang et al. [121] proposed a classification method based on discriminant analysis employing the first and second moments of positions of each nucleotide of the genome sequences as features, performed classification of genomes regarding their Baltimore classification and family (12 families), and obtained a maximum value of accuracy of 88.65% and 85.91%, respectively.

With these considerations in mind, we created an alignment-free feature-based classification method in this section. We performed 8 different classification tasks for each viral sequence from the data set. Specifically, the sequences were classified regarding their genome type, realm, kingdom, phylum, class, order, family, and genus.

We conducted a random 80–20 train–test split on the data set to perform viral classification. Due to classes being imbalanced in the data set, we performed several actions. First, we did not consider classes with fewer than 4 samples. As such, depending on the classification task, the number of samples decreased from 6,091 to the values shown in Supplementary Table S8 (N. Classes column). Second, we performed the train–test split in a stratified way to ensure the representability of each label in the train and test sets. Finally, instead of performing *k*-fold cross-validation, we performed the random train–test split 50 times, and we retrieved the average of the evaluation metrics. Then, we computed the accuracy and the weighted F1-score to select the best-performing method.

Considering these works, herein we perform feature-based classification. As described in the Method section, we applied 5 types of classifiers: LDA [99], GNB [100], KNN [101], SVM [102], and XGB [103].

Furthermore, we performed classification using 7 different features: SL, GC-content (GC), the NC values for the best-performing model, and the NC of the same model with IR configuration to 0, 1, and 2.

These 7 features were fed to all the classifiers, and the accuracy and weighted F1-score were measured to determine which classifier was best suited for this task.

Supplementary Tables S8 and S9 depict the accuracy and weighted F1-score values obtained for each classifier. For all classification tasks, the best-performing classifier was the XGBoost classifier.

Following this, we analyzed if all features were necessary. For that purpose, the XGBoost classifier was used with only the NC feature, the NC with SL and GC, and, finally, using all features. The obtained accuracies are shown in Table 1, and the weighted F1-score results are shown in Supplementary Table S10. The best results are obtained when using all features. This improvement increased when the number of classes was higher, demonstrating that the different compression subprograms (*IR*_0_, *IR*_1_, and *IR*_2_) are more helpful in classifying more specific taxonomic groups.

**Table 1.** Results obtained for viral taxonomic classification task regarding the genome type, realm, kingdom, phylum, class, order, family, and genus using XGBoost classifier. The features used were the genome’s sequence length (SL), the GC-content (GC), and the normalized compression (NC) values for the best model, the same model with IR configuration to 0, 1, and 2. The results correspond to the accuracy (ACC) and the probability of a random sequence being correctly classified (*p_hit_*) using a random classifier (*p_hit_*(*C_Random_*)).

Classification	N. Classes	N. Samples	$\boldsymbol {p_{hit}(C_{Random})}$	$\boldsymbol {ACC_{NC}}$	$\boldsymbol {ACC_{NC+GC}}$	$\boldsymbol {ACC_{NC+SL+GC}}$	$\boldsymbol {ACC_{All~without~SQ}}$	$\boldsymbol {ACC_{All~Features}}$
**Genome**	5	6089	20.00	75.57	80.60	87.11	81.24	**87.25**
**Realm**	5	5799	20.00	77.90	84.56	92.25	86.16	**92.57**
**Kingdom**	10	5788	10.00	76.44	82.51	90.82	84.06	**90.96**
**Phylum**	17	5778	5.88	63.97	70.69	82.36	73.21	**83.41**
**Class**	34	5845	2.94	59.83	65.90	79.05	68.66	**80.23**
**Order**	48	5838	2.08	58.44	65.08	78.20	67.88	**79.62**
**Family**	102	5990	0.98	43.35	54.06	72.46	58.34	**74.46**
**Genus**	360	4673	0.28	35.59	50.02	67.32	54.23	**68.71**

The results show a decrease in accuracy and F1-score when there is an increase in the number of classes. Specifically, we obtained the best performance in the realm classification of the virus (accuracy, 92.57%; F1-score, 0.9234) and our lowest performance in genus classification (accuracy, 68.71%; F1-score, 0.6561). This decrease is mainly because the average number of samples per class decreases as the number of classes increases. As such, many classes may still have an insufficient number of samples to be accurately classified. Supplementary Figure S4 represents the number of samples (genome sequences) per viral genus. Furthermore, part of the classification inaccuracies can be explained by possible errors in the assembly process of the original sequence or eventual subsequence contamination of parts of the genomes. Moreover, other inaccuracies could be due to several genomes being reconstructed using older methods that have been improved since then [122].

Despite being pertinent, the alignment-free studies are not directly comparable due to sample size, absence of classification metrics, and source code. Furthermore, the method proposed in this work is not only alignment free but also feature based, providing a higher level of flexibility since it does not resort directly to the reference genomes but instead to features that the biological sequences share. Therefore, we compared our results with the outcome obtained using a random classifier as a measure of comparison. Specifically, for each task, we determined the probability of a random sequence being correctly classified (*p_hit_*). Overall, there is a vast improvement relative to the random classifier, showing the importance of the features used in the classification process. These classification results seem promising, showing that this metric can be utilized for viral taxonomic classification if enough sequence samples are provided.

## Discussion

The usage of a specialized compressor is crucial to accurately quantify the complexity present in a genome and detect the intrinsic algorithmic nature of the data. Genomic data are highly heterogeneous and have high substitution mutations and data rearrangements, such as fusions, translocations, and inversions [64, 65]. Therefore, the ability of a genomic data compressor to adapt to these heterogeneous data, being able to perform an accurate structure modeling and detect repetitions in the presence of the high substitutional mutations and rearrangements in genomic data, is fundamental to achieve high compressibility of the genome sequence. This article evaluates the capacity to identify data-specific patterns in genomic sequences by comparing the potential of 3 methods to recognize IRs. Precisely, the NBDM was estimated, and the NC was computed using a genomic compressor (GeCo3 [84]) and a general-purpose data compressor (cix and PAQ8 [86,87]). When GeCo3 had the subprogram activated that detects IRs ($NC_{IR_2}$), it showed substantially higher compression than general-purpose because cmix and PAQ use models that do not consider these specific properties of the genomic sequences. The same occurs when comparing GeCo3 ($NC_{IR_2}$) with NBDM, showing that despite NBDM being able to detect small subprograms in synthetic data [56], it cannot detect IRs in genomic data. Moreover, GeCo3 compression capability was resistant to substitutional mutation up to 10%, showing that it can also deal with this extreme nature of genomic data, namely, approximate IRs.

On average, RNA viruses mutate faster than DNA viruses, double-strand viruses mutate slower than single-stranded viruses, and genome size correlates negatively with mutation rate [123]. In this article, we have shown that the redundancy of dsDNA is higher than ssDNA, but for RNA viruses, the opposite occurs. The sequences used in this study to measure a lower NC (higher normalized redundancy) of the ssRNA to dsRNA have approximately the same length. However, the data set of dsRNA has less than 1 order of magnitude in the number of sequences. This difference is natural since the ssRNA is much more abundant than dsRNA. Nevertheless, this discrepancy could justify the higher normalized redundancy of ssRNA in the first instance. However, although the lower average NC values of ssRNA are similar to dsRNA, the dsRNA has higher NC extremes. Therefore, we argue that this difference in the number of sequences in the dsRNA is not significant in changing the lower average of the ssRNA. Also, ssRNA are more prone to mutation than dsRNA [124]. On the other hand, extensive C to U mutations have been reported in many mammalian RNA viruses [95]. This behavior was detected during a much faster evolution of the SARS-CoV-2, an ssRNA virus [125]. Therefore, the faster average decrease of GC-content in ssRNA viruses explains a decrease in the ssRNA entropy and, hence, average NC. A higher GC-content (approximately 2%) of the dsRNA over ssRNA strengthens these outcomes (Supplementary Table S3).

We performed an analysis of the human herpesvirus regarding their genome complexity and IR abundance. Specifically, we analyzed the various behaviors of their subfamilies and identified that different complexities could be representative of the different rates of evolution within these genomes. Finally, we suggest that maybe a higher compressibility and abundance of inversions present in herpesvirus are associated with viral genome integration.

Lastly, we evaluated the capability of using complexity measures to perform viral classification at different taxonomic levels. Notably, results showed that we can automatically and accurately distinguish between viral genomes at different taxonomic levels using the XGBoost classifier with all features (NC with different configurations, GC-content, and SL). However, a decrease in accuracy when approaching the lowest taxonomic levels was observed, which can be increased with future entries to the database. Furthermore, when analyzing viral sequences from environmental samples or integrated genome samples, the length of the original viral genome is often not known. Therefore, we computed the accuracy of a model that does not include this feature. Although we obtained a lower accuracy and F1-score, the results indicate that the method is still reliable for fast and efficient viral taxonomic identification in these scenarios.

Finally, despite the high accuracy results obtained, further improvement of the results may be possible in the classification by adding the transcribed viral proteome information.

## Conclusion

This article shows that the efficient approximation of the Kolmogorov complexities of viral sequences as measures that quantify the absence of redundancy have a profound impact on genome identification, classification, and organization.

For computing an upper bound of the sequence complexity, we benchmark a specific data compressor (GeCo3), after optimization, against other approaches. Specifically, GeCo3 was compared with high compression ratio general-purpose data compressors (PAQ and cmix) and a measure that combines small algorithmic programs and Shannon entropy (BDM). Unlike the other approaches, we show that GeCo3 can efficiently address and quantify regions properly described by simple algorithmic sources, namely, inverted repeats (exact and approximate), among other characteristics.

Using an optimized compression level of GeCo3 in an extensive viral data set, we provide a comprehensive landscape of the viral genome’s complexity, comparing the viral genomes at several taxonomic levels while identifying the genome regarding the lowest and highest proportion of complexity. Specifically, on average, dsDNA viruses are the most redundant (less complex) according to their size, and ssDNA viruses are the less redundant. Contrarily, dsRNA viruses show a lower redundancy relative to ssRNA viruses.

We have performed an in-depth analysis of the human herpesvirus regarding their genome complexity and abundance of IRs. We suggest that a higher compressibility and abundance of inversions in herpesvirus may be associated with viral genome integration.

We describe and use minimal bidirectional complexity profiles of one sequence of each virus to visualize the distribution of complexity of these sequences locally. These profiles can describe actual regions detected in the genome with other methods, proving the description capability of data compression at a structural level.

We reveal the importance of efficient data compression in genome classification tasks, explicitly showing that the complexity, when combined with simple measures (GC-content and size), is efficient in accurately distinguishing between viral genomes at different taxonomic levels without using direct comparisons between sequences.

The methods and results presented in this work provide new frontiers for studying viral genomes’ complexity while magnifying the importance of developing efficient data compression methods for automatic and accurate viral analysis.

## Availability of source code and requirements

- Project name: C.A.N.V.A.S. (Complexity ANalysis of VirAl Sequences)
- Project home page: https://github.com/jorgeMFS/canvas
- Operating system(s): Linux
- Programming language: Bash; Python
- Other requirements: Python v3.6; Conda v4.3.27
- License: MIT License
- RRID:SCR_022552
- biotools:canvas1

The reproduction guidelines are available in the Reproducibility section of the supplementary material.

## Additional Files

**Supplementary Table S1**. Depiction of the parameters used in the 6 custom levels.

**Supplementary Table S2**. Depiction of the parameters used in the template of a target context model.

**Supplementary Table S3**. Depiction of the genome type by the highest normalized compression (NC), normalized compression capacity (NCC), and difference. NCC is computed by $NCC= 1- NC_{IR_{2}} > 0$, and the difference as $difference = NC_{IR_{0}}- NC_{IR_{1}}$. Furthermore, the table shows the genomes’ average sequence length (SL) and GC-content (GC).

**Supplementary Table S4**. Depiction of the top NC values by taxonomic group. Three main groups separate the table. The first represents the highest 10 NC values using standard settings NC (best-performing model); the second group shows the top 10 lowest NC values obtained using the *IR*_2_ subprogram. Finally, the third group shows the top 10 highest values of the difference between NC using *IR*_0_ and *IR*_1_ subprograms.

**Supplementary Table S5**. Depiction of the taxonomic groups with the highest NC values. The table shows each group’s average normalized compression, sequence length, and GC-content.

**Supplementary Table S6**. Depiction of the taxonomic groups with the highest normalized compression capacity (NCC) using only the inverted repeats subprogram *IR*_2_. The top results were obtained by $NCC= 1- NC_{IR_{2}} > 0$. Besides the normalized compression capacity, the table shows each group’s average sequence length and GC-content.

**Supplementary Table S7**. Depiction of the taxonomic groups with the highest difference of values between $NC_{IR_{0}} - NC_{IR_{1}}$. The table shows each group’s average $difference = NC_{IR_{0}}- NC_{IR_{1}}$, sequence length, and GC-content.

**Supplementary Table S8**. Accuracy (ACC) results obtained for viral taxonomic classification tasks regarding genome type, realm, kingdom, phylum, class, order, family, and genus. The classifiers used were linear discriminant analysis (LDA), Gaussian naive Bayes (GNB), K-nearest neighbors (KNN), support vector machine (SVM), and XGBoost classifier (XGB).

**Supplementary Table S9**. F1-score (F1) results obtained for viral taxonomic classification tasks regarding genome type, realm, kingdom, phylum, class, order, family, and genus. The classifiers used were linear discriminant analysis (LDA), Gaussian naive Bayes (GNB), K-nearest neighbors (KNN), support vector machine (SVM), and XGBoost classifier (XGB).

**Supplementary Table S10**. F1-score (F1) obtained for the viral taxonomic classification task regarding genome type, realm, kingdom, phylum, class, order, family, and genus. The features used were the genome’s sequence length (SL), the GC-content (GC) and the normalized compression (NC) values for the best model, the same model with IR configuration to 0, 1, and 2.

**Supplementary Fig. S1**. Illustrations of types of virus morphology. Virus (A) is a helical virus, where the capsoid has a helical shape that envelops the genomic material; virus (B) is icosahedral following cubic symmetry; virus (C) depicts a complex virus, namely, a bacteriophage with a prolate capsid protecting the genomic material; and (D) is virus covered by a viral envelop.

**Supplementary Fig. S2**. Comparison between cmix and GeCo3 when applied to various human herpesviruses regarding computational time and compression ratio obtained (NC).

**Supplementary Fig. S3**. Cladogram showing average *difference* ($NC_{IR_{0}}- NC_{IR_{1}}> 0$). Red depicts the branches where, on average, the genome possesses more inverted repetitions than internal repetitions (higher difference), whereas blue represents the branches with fewer inverted repetitions than internal repetitions (smaller difference).

**Supplementary Fig. S4**. Frequency of genome sequences per viral genus.

giac079_GIGA-D-22-00044_Original_Submission

giac079_GIGA-D-22-00044_Revision_1

giac079_GIGA-D-22-00044_Revision_2

giac079_GIGA-D-22-00044_Revision_3

giac079_GIGA-D-22-00044_Revision_4

giac079_Response_to_Reviewer_Comments_Original_Submission

giac079_Response_to_Reviewer_Comments_Revision_1

giac079_Response_to_Reviewer_Comments_Revision_2

giac079_Response_to_Reviewer_Comments_Revision_3

giac079_Reviewer_1_Report_Original_Submission Anne Kupczok -- 4/10/2022 Reviewed

giac079_Reviewer_1_Report_Revision_1 Anne Kupczok -- 6/6/2022 Reviewed

giac079_Reviewer_2_Report_Original_Submission Kirill Kryukov, Ph.D. -- 4/12/2022 Reviewed

giac079_Reviewer_2_Report_Revision_1 Kirill Kryukov, Ph.D. -- 6/2/2022 Reviewed

giac079_Supplemental_File

## Website

The website of this article is available at https://asilab.github.io/canvas/. This site showcases, among other things, the pipeline of this study, the compressor’s model selection, the detection of inverted repeats in synthetic genomic sequences, the viral genome characterization with regards to genome and type of taxonomic group, and the computed cladograms with a magnifier to allow a better observation of the normalized complexity results with illustrative examples of viruses. Snapshots of our code and other data further supporting this work are openly available in the GigaScience respository, GigaDB [126].

## Abbreviations

A: adenine; ANC: average normalized compression; BDM: block decomposition method; C: cytosine; CTM: coding theorem method; dsDNA: double-stranded DNA; dsRNA: double-stranded RNA; EBV: Epstein–Barr virus; G: guanine; GC: GC-content; GNB: Gaussian naive Bayes; HCMV: human cytomegalovirus; HHV: human herpesvirus; HSV-1: herpes simplex virus 1; HSV-2: herpes simplex virus 2; IR: inverted repeat; K: Kolmogorov complexity; KNN: K-nearest neighbors; KSHV: Kaposi’s sarcoma–associated herpesvirus; LDA: linear discriminant analysis; LSTM: Long Short-Term Memory; MGGC: Multiple Bacteria Genome Compressor; mRNA: messenger RNA; NAF: nucleotide archival format; NBDM: normalized block decomposition method; NC: normalized compression; NR: normalized redundancy; R: redundancy; RdRp: RNA-dependent RNA polymerase; SL: sequence length; ssDNA: single-stranded DNA; ssRNA: single-stranded RNA; SVM: support vector machine; T: thymine; TM: Turing machine; U: uracil; VZV: varicella-zoster virus; XGB: XGBoost.

## Competing Interests

The authors declare no competing interests.

## Funding

This work was partially funded by national funds through the Foundation for Science and Technology (FCT), in the context of the project UIDB/00127/2020. J.M.S. acknowledges the FCT grant SFRH/BD/141851/2018. D.P. is funded by national funds through FCT–Fundação para a Ciência e a Tecnologia, I.P., under the Scientific Employment Stimulus—Institutional Call—reference CEECINST/00026/2018. T.C. is funded by national funds (OE), through FCT–Fundação para a Ciência e a Tecnologia, I.P., in the scope of the framework contract foreseen in the numbers 4, 5, and 6 of the article 23, of the Decree-Law 57/2016, of August 29, changed by Law 57/2017, of July (CEECIND/01463/2017). Thanks are due to FCT/MCTES for the financial support to CESAM (UIDP/50017/2020+UIDB/50017/2020) through national funds.

## Authors’ Contributions

J.M.S. and D.P. designed the experiment, executed data analysis, and wrote the manuscript. All authors analyzed and discussed the results and revised the manuscript.

## Footnotes

1
https://www.ncbi.nlm.nih.gov/labs/virus/vssi/#/virus?SeqType_s=Nucleotide&VirusLineage_ss=Viruses,%20taxid:10239&SourceDB_s=RefSeq&GenomeCompleteness_s=complete&CreateDate_dt=1998-01-01T00:00:00.00Z%20TO%202021-01-22T23:59:59.00Z
