# Supplementary material for: The complexity landscape of viral genomes
Source: Gigascience. 2022 Aug 11;11:giac079. doi: 10.1093/gigascience/giac079 (PMC9366995; doi:10.1093/gigascience/giac079)
Supplement: giac079_GIGA-D-22-00044_Revision_3 [file giac079_giga-d-22-00044_revision_3.pdf]

GigaScience

# The complexity landscape of viral genomes

--Manuscript Draft--

| | |
|---|---|
| **Manuscript Number:** | GIGA-D-22-00044R3 |
| **Full Title:** | The complexity landscape of viral genomes |
| **Article Type:** | Research |



**Funding Information:**

| | |
|---|---|
| FCT – Fundação para a Ciência e a Tecnologia (CEECINST/00026/2018) | Dr. Diogo Pratas |
| FCT/MCTES (UIDP/50017/2020+UIDB/50017/2020+LA/P/0094/2020) | Dr. Tânia Caetano |
| FCT – Fundação para a Ciência e a Tecnologia (SFRH/BD/141851/2018) | Mr. Jorge Miguel Ferreira da Silva |
| FCT – Fundação para a Ciência e a Tecnologia (UIDB/00127/2020) | Not applicable |
| FCT – Fundação para a Ciência e a Tecnologia (CEECIND/01463/2017) | Dr. Tânia Caetano |




**Abstract:**

Background:

Viruses are amongst the shortest yet highly abundant species that harbour minimal instructions to infect cells, adapt, multiply, and exist. However, with the current substantial availability of viral genome sequences, the scientific repertory lacks a complexity landscape that automatically enlights viral genomes' organization, relation, and fundamental characteristics.

Results:

This work provides a comprehensive landscape of the viral genome's complexity (or quantity of information), identifying the most redundant and complex groups regarding their genome sequence while providing their distribution and characteristics at a large and local scale. Moreover, we identify and quantify inverted repeats abundance in viral genomes.
For this purpose, we measure the sequence complexity of each available viral genome using data compression, demonstrating that adequate data compressors can efficiently quantify the complexity of viral genome sequences, including sub-sequences better represented by algorithmic sources (e.g., inverted repeats).
Using a state-of-the-art genomic compressor on an extensive viral genomes database, we show that dsDNA viruses are, on average, the most redundant viruses while ssDNA viruses are the least. Contrarily, dsRNA viruses show a lower redundancy relative to ssRNA. Furthermore, we extend the ability of data compressors to quantify local complexity (or information content) in viral genomes using complexity profiles, unprecedently providing a direct complexity analysis of human Herpesviruses. We also conceive a features-based classification methodology that can accurately distinguish viral genomes at different taxonomic levels without direct comparisons between sequences. This methodology combines data compression with simple measures such as GC-content percentage and sequence length, followed by machine learning classifiers.

Conclusions:

This manuscript presents methodologies and findings that are highly relevant for understanding the patterns of similarity and singularity between viral groups, opening new frontiers for studying viral genomes' organization while depicting the complexity trends and classification components of these genomes at different taxonomic levels. The whole study is supported by an extensive website ( https://asilab.github.io/canvas/ ) for comprehending the viral genome characterization using dynamic and interactive


Powered by Editorial Manager® and ProduXion Manager® from Aries Systems Corporation

| | |
|---|---|
| | approaches. |
| **Corresponding Author:** | Jorge Miguel Ferreira da Silva<br>Universidade de Aveiro Instituto de Engenharia Eletrónica e Informática de Aveiro<br>Esmoriz, Seleccione um PORTUGAL |
| **Corresponding Author Secondary Information:** | |
| **Corresponding Author's Institution:** | Universidade de Aveiro Instituto de Engenharia Eletrónica e Informática de Aveiro |
| **Corresponding Author's Secondary Institution:** | |
| **First Author:** | Jorge Miguel Ferreira da Silva |
| **First Author Secondary Information:** | |
| **Order of Authors:** | Jorge Miguel Ferreira da Silva |
| | Diogo Pratas, Ph.D. |
| | Tânia Caetano, Ph.D. |
| | Sérgio Matos, Professor |
| **Order of Authors Secondary Information:** | |
| **Response to Reviewers:** | We greatly appreciate the opportunity given to review our manuscript. We hope we have been able to address the editor's concerns and that our manuscript is now suitable for publication in GigaScience. |
| **Additional Information:** | |
| **Question** | **Response** |
| Are you submitting this manuscript to a special series or article collection? | No |
| **Experimental design and statistics**<br><br>Full details of the experimental design and statistical methods used should be given in the Methods section, as detailed in our Minimum Standards Reporting Checklist. Information essential to interpreting the data presented should be made available in the figure legends.<br><br>Have you included all the information requested in your manuscript? | Yes |
| **Resources**<br><br>A description of all resources used, including antibodies, cell lines, animals and software tools, with enough information to allow them to be uniquely identified, should be included in the Methods section. Authors are strongly | Yes |

| | |
|---|---|
| encouraged to cite Research Resource Identifiers (RRIDs) for antibodies, model organisms and tools, where possible.<br><br>Have you included the information requested as detailed in our Minimum Standards Reporting Checklist? | |
| **Availability of data and materials**<br><br>All datasets and code on which the conclusions of the paper rely must be either included in your submission or deposited in publicly available repositories (where available and ethically appropriate), referencing such data using a unique identifier in the references and in the "Availability of Data and Materials" section of your manuscript.<br><br>Have you have met the above requirement as detailed in our Minimum Standards Reporting Checklist? | Yes |

Click here to access/download;LaTeX - Main Document (TeX file);main.tex

*GigaScience*, 2022, 1–11

doi: xx.xxxx/xxxx
Manuscript in Preparation
Paper

PAPER

# The complexity landscape of viral genomes

Jorge Miguel Silva[1,*], Diogo Pratas[1,2,3], Tânia Caetano[4] and Sérgio Matos[1,2]

[1]Institute of Electronics and Informatics Engineering of Aveiro, University of Aveiro, Portugal and [2]Department of Electronics, Telecommunications and Informatics, University of Aveiro, Portugal and [3]Department of Virology, University of Helsinki, Finland and [4]CESAM and Department of Biology, University of Aveiro, Aveiro, Portugal

*Correspondence address. Jorge Miguel Silva E-mail: jorge.miguel.ferreira.silva@ua.pt https://orcid.org/0000-0002-6331-6091

## Abstract

**Background:** Viruses are amongst the shortest yet highly abundant species that harbour minimal instructions to infect cells, adapt, multiply, and exist. However, with the current substantial availability of viral genome sequences, the scientific repertory lacks a complexity landscape that automatically enlights viral genomes' organization, relation, and fundamental characteristics.
**Results:** This work provides a comprehensive landscape of the viral genome's complexity (or quantity of information), identifying the most redundant and complex groups regarding their genome sequence while providing their distribution and characteristics at a large and local scale. Moreover, we identify and quantify inverted repeats abundance in viral genomes. For this purpose, we measure the sequence complexity of each available viral genome using data compression, demonstrating that adequate data compressors can efficiently quantify the complexity of viral genome sequences, including sub-sequences better represented by algorithmic sources (e.g., inverted repeats). Using a state-of-the-art genomic compressor on an extensive viral genomes database, we show that dsDNA viruses are, on average, the most redundant viruses while ssDNA viruses are the least. Contrarily, dsRNA viruses show a lower redundancy relative to ssRNA. Furthermore, we extend the ability of data compressors to quantify local complexity (or information content) in viral genomes using complexity profiles, unprecedently providing a direct complexity analysis of human Herpesviruses. We also conceive a features-based classification methodology that can accurately distinguish viral genomes at different taxonomic levels without direct comparisons between sequences. This methodology combines data compression with simple measures such as GC-content percentage and sequence length, followed by machine learning classifiers.
**Conclusions:** This manuscript presents methodologies and findings that are highly relevant for understanding the patterns of similarity and singularity between viral groups, opening new frontiers for studying viral genomes' organization while depicting the complexity trends and classification components of these genomes at different taxonomic levels. The whole study is supported by an extensive website (https://asilab.github.io/canvas/) for comprehending the viral genome characterization using dynamic and interactive approaches.
**Key words**: Viruses; Genomics; Sequence-analysis; Data Compression; Cladograms; Viral Classification; Algorithmic Information Theory.

## Introduction

Viruses are a strong driving force of life and evolution. They are the shortest and most abundant life realm, estimated at around $10^{31}$ particles [1]. Likewise, viruses occupy almost every ecosystem [2, 3, 4] and infect all types of life forms [5, 6].

Viruses depend on the host's cell for replication. This dependence has forced viruses to interact with cellular pathways to successfully hijack and customize the host cell machinery for viral production. This interaction generated a long-standing effect of adaptation and counter-adaptation between host and viruses for gene expression and nucleic acid synthesis. Furthermore, during their replication, viruses can perform horizontal gene transfer, which increases the host species' genetic diver-

**Compiled on:** July 13, 2022.
Draft manuscript prepared by the author.

**Key Points**

- We provide a comprehensive landscape of the viral genomes complexity.
- We demonstrate that data compressors can efficiently quantify the complexity of viral genome sequences, including subsequences better represented by algorithmic sources.
- We identify and quantify inverted repeats abundance in viral genomes.
- We use minimal bi-directional complexity profiles as local measures of the viral genome.
- We present an in-depth complexity analysis of the human herpesviruses.
- We show that the viral genome redundancy, GC-content, and size are efficient features to accurately distinguish between viral genomes at different taxonomic levels.
- Our work opens new frontiers for studying viral genomes' complexity while depicting complexity trends in viral genomes.

sity analogously to the process of sexual reproduction [7].

Despite the significant impact that viruses have on the evolution of living beings and the ecosystem, our understanding of viruses is still relatively limited compared with other realms of life. In particular, the complexity landscape of viruses is unknown. For example, what are the most redundant and complex viral DNA/RNA sequences? Which viruses contain more genomic inversions? How does the complexity distribution of viruses describe their morphology and behaviour? What can be uncovered by analyzing the complexity of the viral genomes regarding viral processes? Moreover, is the information uncovered shared between the same viral groups? By studying the complexity of viral sequences and performing information quantification, one might be able to answer some of these questions.

Complexity analysis of the genome sequences is not new and is frequently performed by data compressors, which serve as an upper bound to Kolmogorov Complexity. Many examples of these studies appeared after creating the first compressor for DNA sequences [8]. Specifically, data compression has been used to detect repeated sequences in the Plasmodium falciparum DNA, and observed patterns were related to large-scale chromosomal organization and gene expression control [8]. The XMAligner tool [9] was created for pairwise genome local alignment, which considers a pair of nucleotides from two sequences related if their mutual information in context is significant. To measure the information content of nucleotides in sequences, they used a lossless compression method. Graph compression was used for comparing large biological networks [10]. This method was done by compressing the original network structure and then measuring the similarity of the two networks using the compression ratio of the concatenated networks. The method was applied to several organisms, showing an efficient capability to measure the similarities between metabolic networks. Data compression was used to approximate the Kolmogorov complexity and applied to data derived from sequence alignment data [11]. This process identified a novel way of predicting three different aspects of protein structure: secondary structures, inter-residue contacts and the dynamics of switching between different protein states. An analysis of the complexity of different DNA genomes was performed, demonstrating various evolution-related findings linked with complexity, notably that archaea have a higher relative complexity than bacteria and eukaryotes on a global scale. Furthermore, viruses have the most complex sequences according to their size [12]. Metagenomic composition analysis of a sedimentary ancient DNA sample was performed using relative compression of whole-genome sequences [13]. The results showed that several viruses and bacteria expressed high levels of similarity relative to the samples. Finally, an alignment-free tool was created to accurately find genomic rearrangements of DNA sequences following previous studies, which took alignment-based approaches or performed FISH [14].

Given the applicability of compression methods in the analysis of genomic sequences and intending to better understand viruses, in this manuscript, we perform an extensive complexity analysis of the viral world through the automatic computational analysis of its genome complexity and associated characteristics. Specifically, we use a genomic compressor to analyze the complexity across viral taxonomies and quantify the algorithmic information embedded in viral genome sequences better represented by small programs. Several questions arise when addressing this problem: How much information is present in a viral genome? What is the best way to quantify the information in a viral genome? What type of information can we retrieve from analyzing the complexity of the viral genome? We use unsupervised probabilistic and algorithmic information quantification of viral genomes to answer these questions. We use a high-quality database using the NCBI reference database with 12,168 complete reference genomes from 9,605 viral taxa.

Since studying the complexity of a DNA/RNA sequence requires efficient data compressors that take into account the probabilistic and algorithmic characteristics of the data, we compared several state-of-the-art genomic data compressors and another approximation of the Kolmogorov complexity besides data compression. This comparison was made to evaluate their ability to detect Inverted Repeats (IRs) with increasing levels of mutations. The results show that GeCo3 could detect and compress IRs, unlike other programs, using appropriate computational resources.

Consequently, GeCo3 was used to analyze viruses' complexity and overall abundance of inverted repeats and construct cladograms. The results of our study show several insights into patterns between the complexity and viral groups and that these measurements can perform viral genome authentication and classification with high accuracy without directly comparing the sequences but instead using the individual features.

The following section describes the paper's background and related work. A description of the methods follows and the results obtained. Finally, we discuss the significant results obtained, draw conclusions, and point out possible future work lines.

## Background

This manuscript shows that the efficient use of specific data compressors to quantify data complexity (Kolmogorov complexity) profoundly impacts viral genomes identification, classification, and organization. For introducing several concepts, this section provides an overview of the viral nature, Kol-

mogorov complexity and data compression, and the role of inverted repeats in the genome sequence.

## Viruses Microbiology

Viruses are submicroscopic biological infectious agents that require living cells of an organism to be active for replication [15] (for more information regarding viral morphology and genome see the Supplementary material of this article).

They have a vast size variation, ranging from around 10 nm with small genomes to viruses with similar dimensions and genome sizes to Bacteria and archaea [16, 17]. These viruses are called giant viruses and contain many unique genes currently not found in other life forms.

There can also be hybrid viruses [18], making it difficult to identify species [19]. There are several possible combinations for the creation of a hybrid virus. One possible way of occurring is the infection of a host's cell by two or more related viruses and consequential exchange of sequences between viruses. The result is the creation of a new variant derived from the parental genomes. Another possible way is the recombination of RNA viral genomes with the host's RNA. Finally, there is evidence that small DNA viruses could have been created by recombination events between RNA viruses and DNA plasmids [18].

Although the origin of viruses is still uncertain, they play an essential role in the evolution of living organisms since they are horizontal gene transfer vehicles. This biological phenomenon increases genetic diversity. Furthermore, it occasionally allows viral genetic material to integrate into the host genomes, transferred vertically to its offspring. This property is so preponderant in evolution that the origin of the eukaryotic nucleus might be related to this process [20, 21, 22].

Additionally, viral genomic integration allows us to infer the evolutionary distance between hosts by observing the shared virus integrated into their genomes. For instance, in humans, viruses frequently establish persisting infections [23] and imprint their genetic material in the tissues throughout life, displaying phylogeographies patterns. These can be used as markers to understand the human population history and migrations better and provide new insights into unidentified individuals' origins on both global and local scales [24]. In this respect, the JC polyomavirus is one of the most comprehensively studied viruses. Its genotype-specific global spread has been suggested to indicate the origins of modern [25] and ancient humans [26, 27, 28]. Furthermore, a worldwide study supported the co-dispersal of this virus with major human migratory routes and its co-divergence with human mitochondrial and nuclear markers [29].

Thus, computer analysis of viral and host DNA sequences is fundamental to understanding the evolutionary relationships between different viruses and their hosts, identifying modern viruses' ancestors, and better understanding their behaviour and function. Also, the genomic sequences encode the production of proteins and their high-dimensional folding structure [30, 31]. Therefore, the direct study of viral genome sequences also develops the knowledge of the viral mechanism of protein formation and assembly.

## Inverted Repeats

Inverted Repeats (IRs) are nucleotide sequences with a downstream reverse complement copy, causing a self-complementary base-pairing region [32]. Consequently, IRs usually fold into different secondary structures (hairpin- and cruciform-like structures, pseudoknots) that participate or interfere in many cellular processes in all forms of life, including DNA replication [33, 11]. Due to these traits, IRs play an essential role in genome instability [35], contributing to mutability. This mutability can create diseases in the short term [36], but across long periods leads to cellular evolution and genetic diversity [37]. In many viruses, IRs in pseudoknots are involved in ribosomal frameshifting. This translational mechanism allows the production of different proteins encoded by overlapping open reading frames (ORFs) of the same mRNA [38, 39]. This feature allows them to encode a more significant amount of genetic information in small genomes and constitutes another level of gene regulation [40].

The genomes of some viruses, such as parvovirus, are flanked by inverted terminal repeats (ITRs) that form hairpin structures functioning as a duplex origin of replication sequence [33, 41]. Therefore, these ITRs contain most of the cis-acting information needed for viral replication and viral packaging [41]. In adeno-associated viruses, ITRs are essential for intermolecular recombination and circularization of genomes [42]. IRs can also function as termination transcription signals, especially in giant viruses [43, 44].

## Kolmogorov Complexity and Data Compression

Solomonoff, Kolmogorov, and Chaitin [45, 46, 47, 48] described the notion of data complexity by showing that there is at least one minimal algorithm among all the algorithms that decode strings from their codes. For all strings, this algorithm allows codes as short as any other, up to an additive constant that depends only on the strings themselves. Concretely, algorithmic information is a measure that quantifies the information of a string $x$ by determining its complexity $K(x)$ by

$$K(x) := \min_{p}\{l(p) : U(p) = x\}, \quad (1)$$

where $K(s)$ is defined by a shortest length $l$ of a binary program $p$ that computes the string $x$ on a universal Turing machine $U$ and halts [47]. This notion that the complexity of a string can be defined as the length of a shortest binary program that outputs that string was universally adopted and is the standard to perform information quantification. It differs from Shannon's entropy because it recognises that the source creates structures which follow algorithmic schemes [49, 50], rather than regarding the machine as generating symbols from a probabilistic function.

While the Kolmogorov complexity is non-computable, it can be approximated with programs for such purpose. A possible approximation is the Coding Theorem Method (CTM) [51], and its improved version, the Block Decomposition Method (BDM) [52], which approximate local estimations of algorithmic complexity providing a closer relationship to the algorithmic nature. This approximation decomposes the quantification of complexity for segmented regions using small Turing machines [51]. For modelling the statistical nature, such as noise, it commutes into a Shannon entropy quantification. This approach has shown encouraging results for many distinct purposes [53, 54, 55]. However, it has also shown underestimation issues related to side information [56].

The classical approximation of the Kolmogorov complexity is performed using data-compressors with probabilistic and algorithmic schemes [57]. Data compressors are a natural solution to measure complexity, since, with the appropriate decoder, the bitstream produced by a lossless compression algorithm allows the reconstruction of the original data and, therefore, can

be seen as an upper bound of the algorithmic complexity of the sequence. For a definition of safe approximation, see [58].

In genomics, sequences can be codified as messages using a four symbol alphabet ($\Sigma = \{A, C, G, T\}$ for DNA sequences and $\Sigma = \{A, C, G, U\}$ for RNA sequences). These messages contain instructions for survival and replication of the organism, its' morphology and historical marks from previous generations [59]. Initially, genomic sequences were compressed with general-purpose data-compressors such as gzip [60], bzip2 [61], or LZMA [62]. However, this paradigm shifted towards using a specific compression algorithm after introducing BioCompress [63]. Genomic compressors can outperform general-purpose compressors since they are designed to consider specific genomic properties such as the presence of a high number of copies and substitutional mutations, and multiple rearrangements, such as inverted repeats [64, 65].

Given this advantage of using specific compressors for the compression of genomic data, several algorithms have emerged to model these genomic data behaviours [66]. Specifically, several algorithms have been created to model repetitions and inverted repetitions in the genome regions through simple bit encoding, dictionary approaches and context modelling [67, 68, 69, 70, 71, 72, 73, 74, 75, 76, 77].

Currently, state-of-the-art compressors have different objectives, such as optimizing for compression strength or prioritizing a balance between compression speed and compression capability. Examples of the latter are NAF (Nucleotide Archival Format) [78, 79] and MBGC (Multiple Bacteria Genome Compressor) [80], which are more suitable for collections of data and frequently used by computational biologists. Compressors focused on compressibility at the expense of more computational resources, on the other hand, generally apply statistical and algorithmic model mixtures combined with arithmetic encoding. Among the best compressors regarding compression ratio performance for various genomic sequences, the best results are provided by cmix [81], XM [82], Jarvis [83], and Geco3 [84]. For additional information regarding data compressors' compressibility capacity of genomic sequences, see [85]. Cmix [81] is a general-purpose lossless data compression program that optimises compression ratio at the cost of high CPU/memory usage. It is based on PAQ compressors [86, 87] but dramatically increases the amount of processing per input bit and computational memory. Current updates include LSTM (Long Short-Term Memory) based models [88]. The XM compressor [82] uses three types of experts: repeat models, a low-order context model, and a short memory context model. On the other hand, Jarvis [83] uses a competitive prediction model that estimates for each symbol the best class of models to be used. There are two classes of models: weighted context models and weighted stochastic repeat models, where both classes of models use specific sub-programs to handle inverted repeats efficiently. Finally, GeCo3 [84], currently one of the best performing reference-free data compressors, uses neural networks to improve upon the results of specific genomic models of GeCo2 [89]. Specifically, the neural networks are used in mixing multiple contexts and substitution-tolerant context models of GeCo2. Furthermore, GeCo3 has embedded subprograms capable of detecting genome-specific patterns, such as inverted repeats.

## Methods

This section describes the measures used in this paper. Specifically, we first define information-based measures: the Normalized Block Decomposition Method, the Normalized Compression (NC) with different subprograms, the Normalized Compression Capacity (NCC), the difference between NCs, and the minimal bi-directional complexity profiles. Afterwards, we define the GC-Content, and the compression benchmark performed. Finally, we described the classification pipeline. Specifically, the features and classifiers used and the metrics utilized for evaluating the model's performance.

### Information-based measures

This section describes two approximations of the Kolmogorov complexity, one based on the decomposition of a string into blocks and their approximation based on the output of small Turing machines (Block Decomposition Method) and another based on data compression. The data compression approach was utilized to compute the Normalized Compression and construct the minimal bi-directional complexity profiles. Therefore, in this subsection, we describe the Normalized Compression (NC), the minimal bi-directional complexity profiles, and the Normalized Block Decomposition Method (NBDM).

#### *Normalized Block Decomposition Method (NBDM)*

A possible approximation of the Kolmogorov complexity is given by using small Turing machines (TM), which approximate the components of a broader representation. The Coding Theorem Method (CTM) uses the algorithmic probability between a string's production frequency from a random program and its algorithmic complexity. The more frequent a string is, the lower its Kolmogorov complexity, and the lower frequency strings have, the higher Kolmogorov complexity is. The Block Decomposition Method (BDM) increases the capability of a CTM, approximating local estimations of algorithmic information based on Solomonoff-Levin's algorithmic probability theory. In practice, it approximates the algorithmic information, and when it loses accuracy, it approximates the Shannon entropy. Since in this article we use BDM to perform a comparison with the Normalized Compression, we considered the normalization of the BDM (NBDM) according to [56]. In this case, the NBDM is computed as

$$\mathrm{NBDM}(x) = \frac{BDM(x)}{|x| \log_2 |\Sigma|} = \frac{BDM(x)}{2 \times |x|}. \quad (2)$$

where $x$ is a string, $BDM(x)$ is the BDM value of the string, $|\Sigma|$ the number of different elements in $x$ (size of the alphabet) and $|x|$ the length of $x$. Since we have a four symbol alphabet ($\Sigma = \{A, C, G, T\}$ for DNA sequences and $\Sigma = \{A, C, G, U\}$ for RNA sequences), $|\Sigma| = 4, \log_2(4) = 2$. Although BDM has difficulty dealing with full information quantification due to the block representability, it has proven to be a helpful tool for measuring and identifying data content similar to simple algorithms [56].

#### *Normalized Compression (NC)*

An efficient compressor provides an upper bound approximation for the Kolmogorov complexity. Specifically, $K(x) < C(x) \leq |x| \log_2 |\Sigma|$, where $K(x)$, is the Kolmogorov complexity of the string $x$ in bits, $C(x)$ is the compressed size of $x$ in bits, and $|x|$ is the length of string $x$. This relation neglects the constant that asymptotically becomes irrelevant. Usually, an efficient data compressor is a program that approximates both probabilistic and algorithmic sources using affordable computational resources (time and memory). Although the algorithmic nature may be more complex to model, data compressors can have embedded sub-programs to handle this nature. The normalized version, known as the Normalized Compression (NC), is defined by

$$\text{NC}(x) = \frac{C(x)}{|x| \log_2 |\Sigma|} = \frac{C(x)}{2 \times |x|}. \quad (3)$$

Given the normalization, the NC enables to compare the proportions of information contained in the strings independently from their sizes [12]. If the compressor is efficient, then it can approximate the quantity of probabilistic-algorithmic information in data using affordable computational resources. In our work, to determine the NC, we made use of the state-of-the-art genome compressor GeCo3 [84], with the level 16 that yielded the best average results (benchmark provided in the results section).

Besides the computation of the NC using the standard configuration of this model, we also computed the NC using GeCo3 with three subprogram configurations. These subprogram configurations address the use or absence of inverted repetitions, namely:

- $IR_0 \rightarrow$ uses the regular context model without IR detection;
- $IR_1 \rightarrow$ uses IR detection simultaneously with the regular context model;
- $IR_2 \rightarrow$ uses IR detection sub-program without regular context models.

There was a need to determine the sequences with the highest normalized compression capacity (*NCC*) in some cases. When the compressor was only using the subprogram $IR_2$, *NCC* was computed as $NCC_{IR_2}(x) = 1 - NC_{IR_2}$. Only positive values were considered to filter computations where the compressor could not compress the sequence sufficiently. Another measure used to quantify inverted repeats was the difference between $NC_{IR_0}$ and $NC_{IR_1}$.

***Minimal bi-directional complexity profiles***

A complexity profile is a numerical sequence describing for each symbol ($x_i$) of a sequence $x$ the number of bits required for its compression assuming a causal order [90]. A minimal bi-directional complexity, $B(x)$, profile assumes the minimal representation of compressing the sequences using both directions independently, namely $\overrightarrow{C}(x_i)$ as from the beginning to the end of the sequence, and $\overleftarrow{C}(x_i)$ as from the end to the beginning [91]. Accordingly, these profiles are defined as

$$B(x_i) = \min\{\overrightarrow{C}(x_i), \overleftarrow{C}(x_i)\}. \quad (4)$$

The construction of these profiles follows a pipeline formed of many transformations, including reversing, segmenting, inverting, and the use of specific low-pass filters after data compression to achieve better visualization. For computing these profiles, we use the GTO toolkit [92].

The generation of these profiles is robust to localize specific features in the sequences, namely low and high complexity sequences, inverted repeat regions, duplications, among others.

## Other Measures

The two other measures used to perform viral analysis and classification are the GC-Content (GC) and the length of the viral genome $|x|$.

GC-Content (GC) represents the proportion of guanine (G) and cytosine (C) bases out the quaternary alphabet ($\Sigma = \{A, C, G, T/U\}$). This includes thymine (T) in DNA and uracil (U) in RNA. The GC percentage is given by the number of cytosine (C) and guanine (G) bases in a viral genome $x$ with length $|x|$ according to

$$\mathcal{GC}(x) = \frac{100}{|x|} \sum_{i=1}^{|x|} \mathcal{N}(x_i || x_i \in \Xi), \quad (5)$$

where $x_i$ is each symbol of $x$ (assuming causal order), $\Xi$ is a subset of the genomic alphabet containing the symbols $\{G, C\}$ and $\mathcal{N}$ the program that counts the numbers of symbols in $\Xi$.

GC-content is variable between different organisms and correlates with the organism's life-history traits, genome size [93], and GC-biased gene conversion [94]. Furthermore, in RNA viruses, excess C to U substitutions accounted for 11–14% of the sequence variability of viruses, indicating that a decrease in GC-content is a potent driver of RNA viruses' diversification and longer-term evolution [95]. As such, this measure helps perform viral classification.

On the other hand, it was shown that the number of base stackings (typical arrangement of nucleobases found in the three-dimensional structure of nucleic acids) is one of the most critical elements contributing to the thermal stability of double-stranded nucleic acids. Furthermore, due to the relative locations of exocyclic groups, GC pairings have higher stacking energy than AT or AU pairs [96]. This energy accumulation in the GC pair in an organism's genome makes the DNA more prone to mutation. Thus, over time, a species tends to decrease its GC-content to become more stable [97], giving us further information regarding viral characterization.

## Data Description

The dataset is composed of 12,163 complete reference genomes from 9,605 viral taxa retrieved from NCBI database on 22 of January 2021 using the following footnote url[1]. The download was performed in a custom manner to retrieve the taxonomic id, host and geolocation of each reference genome. The metadata header was removed from each sequence using the GTO toolkit [92], where any nucleotide outside the quaternary alphabet $\{A, C, G, T/U\}$, was replaced by a random nucleotide from the quaternary alphabet. Notice that the sequences with symbols outside the alphabet are scarce. Finally, the type of genome and the taxonomic description of each sequence were retrieved using Entrez-direct [98].

Then, the retrieved NCBI sequences were filtered to remove possibly contaminated or poorly sequenced sequences. Firstly, using the taxonomic metadata, sequences that did not hold complete taxonomic information down to the genus rank and any sequences that maintained a taxonomic description of unclassified were removed. Secondly, we applied a filter to remove outlier sequences. Specifically, after computing all sequences' length, GC-Content, and Normalized Complexities, sequences whose measure fell outside $\mu \pm 3 \times \sigma$ (approximately 0.03% of all sequences) of any measure were removed. A total of 182 sequences were removed since they most likely have errors in the assembly process or contamination. After filtering, we kept 6,091 of the initial 12,163 sequences.

1 https://www.ncbi.nlm.nih.gov/labs/virus/vssi/#/virus?SeqType_s=Nucleotide&VirusLineage_ss=Viruses,%20taxid:10239&SourceDB_s=RefSeq&GenomeCompleteness_s=complete&CreateDate_dt=1998-01-01T00:00:00.00Z%20TO%202021-01-22T23:59:59.00Z

## Data compressors and Level selection benchmark

First, we tested cmix and GeCo3 regarding compression ratio and time required per sequence compression. This was followed by selection of a total of 19 levels of models in GeCo3 to determine the best level configuration to compress the viral sequences. These levels correspond to the default 13 levels of the GeCo3 compressor and 6 others built for this task. The list of the levels used are shown in Table S1, and the description of parameters can be found in Table S2. The 13 default levels of the compressor have increasingly higher complexity and take longer to run since they use higher context models. Therefore, since the first and lightest level performed best, the other six custom-build levels were also built with lightweight models.

## Classification

We tested several machine learning algorithms to perform the genomic and taxonomic classification task, namely, the classifiers used were Linear Discriminant Analysis (LDA) [99], Gaussian Naive Bayes (GNB) [100], K-Nearest Neighbors (KNN) [101], Support Vector Machine (SVM) [102], and XGBoost classifier (XGB)[103].

Linear Discriminant Analysis is a generalization of Fisher's linear discriminant, a method used in statistics and other fields, to find a linear combination of features that separates classes of objects. The resulting combination can be used as a linear classifier [99]. Gaussian Naive Bayes is defined as a supervised machine learning classification algorithm based on the Bayes theorem following Gaussian normal distribution [100]. K-Nearest Neighbors is another approach to data classification, taking distance functions into account and performing classification predictions based on the majority vote of its neighbors [101]. Support Vector machines are supervised learning models with associated learning algorithms that construct a hyperplane in a high-dimensional space using data and perform classification [102]. Finally, XGBoost [103] is an efficient open-source implementation of the gradient boosted trees algorithm. Gradient boosting is a supervised learning algorithm that predicts a target variable by combining the estimates of a set of simpler models. Specifically, new models are created that predict the residuals or errors of prior models and then added together to make the final prediction. This task uses a gradient descent algorithm to minimize the loss when adding new models. XGBoost can use this method in both regression and classification predictive modeling problems.

The accuracy and weighted F1-score were used to select and evaluate the classification performance of the measures. Accuracy is the proportion between correct classifications and the total number of cases examined, while the F1-score is computed using the precision and recall of the test. We utilized the weighted version of the F1-Score due to the presence of imbalanced classes.

For comparison of the obtained results, we assessed the outcomes obtained using a random classifier. For that purpose, for each task, we determined the probability of a random sequence being correctly classified ($p_{hit}$) as

$$p_{hit} = \sum_{i=0}^{n} [p(c_i) * p_{correct}(c_i)], \quad (6)$$

where $p(c_i)$ is the probability of each class, determined as

$$p(c_i) = \frac{|samples_{class}|}{|samples_{total}|}.$$

On the other hand, $p_{correct}(c_i)$ is the probability of that class being correctly classified. In the case of a random classifier,

$$p_{correct}(c_i) = \frac{1}{|classes|}.$$

# Results

The results reported in this manuscript can be computed using the minimal characteristics described in Supplementary Subsection entitled Software and Hardware recommendations and using the procedures described in Supplementary Subsection entitled Reproducibility. The following subsections describe the data, the compression level selection benchmark, the synthetic sequence benchmark, the viral genome analysis and cladograms, and the viral classification application.

## Data compressors and Level selection benchmark results

Viral genomes have specific characteristics, for example, short length, high average complexity, and specific structures, that require the proper optimization of the data compressor to provide higher modelling adaptability and efficiency. Cmix and GeCo3 are state-of-the-art genomic compressors. To assess the viability of each compressor, we tested their computational time and NC values on a small sample consisting of 8 medium size viral genomes. The results, presented in Figure S2 of the supplementary material, show that the compression ratio of GeCo3 is, on average, slightly better, with a much more reasonable computational time (on average, three orders of magnitude faster than cmix). As such, for the remaining of the work, we consider the GeCo3 compressor.

On the other hand, GeCo3 contains many types of compression levels [84]. Therefore, we applied GeCo3 to each viral genome from the dataset using 19 different levels and computed its normalized compression (NC).

We evaluated the frequency where each level yielded the lowest NC (provided the best compression for a given sequence; Figure 1 A) and determined the sum of the NC from the compression of all reference genomes for each model (Figure 1 B). Overall, we selected level 16 because it provided the lowest NC on average (28.38% as the best compression level) and the lowest NC sum from compressing all reference genomes. This level is constituted by a mixture using a neural network with the following models:

- Model 1 → context-order of 1, alpha parameter of 1 (without inverted repeats), and gamma parameter of 0.7;
- Model 2 → context-order of 12, alpha parameter of 1/50 (with inverted repeats), and gamma parameter of 0.97.

The chosen level is constituted by two models with a small and average context model. This configuration performed better because most viral genomes are small and compact, where a small genomic space usually separates repetitions and IRs. Therefore, the depth of the models is more adapted to provide higher efficiency to the average of the viral genomes than, for example, a higher context model (higher than 13) that can perform marginally better in more extensive and repetitive sequences, but that loses sensitivity in the average of the genomes.

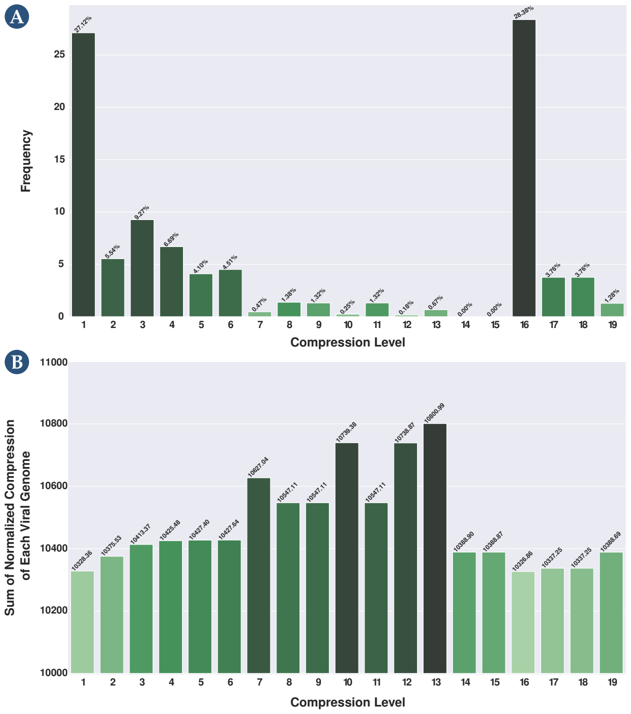


**Figure 1.** Selection of a level for GeCo3 from a pool of 19 levels. (**A**) depicts the frequency where each level provided the best NC results, and (**B**) shows for each level the sum of the NC from the compression of all reference genomes. For better visualization, please visit the website https://asilab.github.io/canvas/.

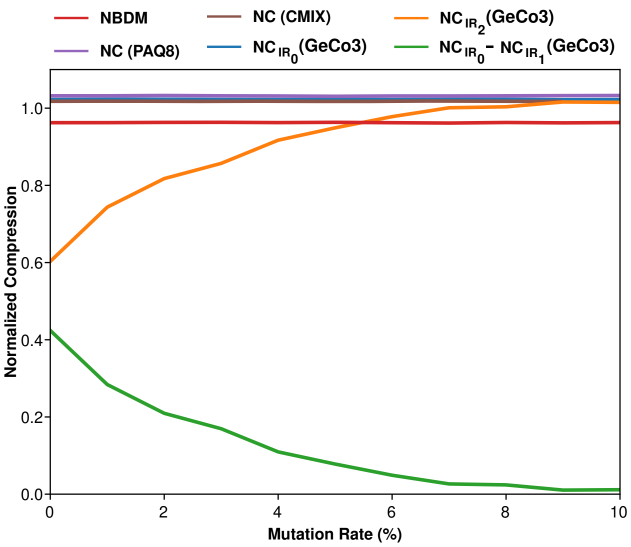


**Figure 2.** Plot describing the variation of Normalized Compression (*NC*) and Normalized Block Decomposition Method (NBDM) with an increase of mutation rate of a sequence (0%–10%). The NC was computed using the state-of-the-art genomic compressor (GeCo3 [84]) and a general-purpose compressor (PAQ8 [104]). A red line depicts the NBDM, the NC value using cmix with brown, and PAQ8 by a purple line. Furthermore, the GeCo3 compressor with ($IR_2$) and without the IR detection subprogram ($IR_0$) is shown in orange and blue lines, respectively. Finally, the green line shows the difference between $NC_{IR_0} - NC_{IR_1}$.

## Synthetic sequence benchmark

Viral genomes can contain IRs that are subsequences better described using simple algorithmic approaches. To benchmark the capability of different programs to quantify IRs accurately, we created a genomic sequence of 10,000 nucleotides in which the last 5,000 were inverted repeats of the first 5,000. This size was chosen since the median size of the viral genomes is 9,836 bases, which is close to the total size of the synthetic sequence generated. This sequence was mutated incrementally from 0% to 10%, meaning that the number of IRs decreases with the increase of nucleotide substitutions. For each sequence, the NC was computed with (Figure 2): i) GeCo3, without and with the IR detection program ($IR_0$ and $IR_2$, respectively), ii) PAQ8 and iii) Cmix. Additionally, the Normalized Block Decomposition Method (NBDM) was also computed as a more prone measure of algorithmic nature quantification. Results show that GeCo3 with the $IR_2$ subprogram compresses the sequences better than the other programs since its NC is lower at 0% mutational rate (Figure 2). All other compressors (cmix and PAQ8) could not detect IRs and compress the sequence. Furthermore, NBDM can also not detect the IRs because it provides the same high value across sequences with various mutation rates. It is also evident that GeCo3 with $IR_2$ can detect IRs even in the presence of substantial mutations (5% of mutation) and takes into account different levels of nucleotide substitutions because it increases with the increase of the mutational rate (i.e. decrease of IRs). The difference between $NC_{IR_0}$ and $NC_{IR_1}$, both computed with GeCO3, was also analyzed. Its profile is inverse to the $IR_2$ and confirms that nucleotide substitutions' accumulation decreases the number of IRs in the sequence.

## Viral genome analysis and cladograms

The core of the viral genomes was analyzed in terms of complexity landscape, including the trends, singularities, and patterns for both the use or absence of IRs. The NC, using GeCo3, with $IR_0$, $IR_1$ and $IR_2$ subprograms was determined and the $NCC_{IR_2}$ was calculated. The outcome was interpreted according to the genome type or the taxonomic group, together with the average of their genome sizes (Figure 3 and Table S3). Notice that the NC enables to compare proportions of the absence of redundancy independently from the sizes of the genomes. This value is complementary to the normalized redundancy. Specifically, consider the redundancy (R) of a sequence x, as $R(x) = log_2(A)|x| - C(x)$, where $|x|$ is the length of the sequence, A is the cardinally of the sequences' alphabet and C(x) is the compressed size of x in bits, and the normalized redundancy (NR) as $NR(x) = 1 - (C(x)/(log_2(A)|x|))$.

### *Complexity landscape according to genome type*

According to NCBI, the virus's genomes herein analyzed are of five types: dsDNA, ssDNA, dsRNA, ssRNA and mixed-DNA. Results show that ssDNA, followed by mixed-DNA and dsRNA viruses, are the genomes with higher NC, whereas dsDNA genomes have the lowest (Figure 3 A; Table S3). In general, smaller genomes are less complex and are more likely to contain fewer repeats and, hence, less redundancy, and the ssDNA, mixed-DNA and dsRNA genomes have smaller average sequence lengths (3282 bp, 3258 bp, and 8377 bp; Table S3).

According to the *NCC* and the $NC_{IR_0} - NC_{IR_1}$ difference results, dsDNA and ssDNA have most significant quantities of IRs than the other genome types. This can be due to ITRs present at the ends of some dsDNA viruses, such as Adenovirus and Ampullaviruses, and ssDNA virus as Parvoviruses, or other IRs structures important that perform ribosomal frameshifting.

### *Complexity landscape according to taxonomic level*

In complexity analysis of viral genomic sequences, when considering the Realm taxonomic level (Figure 3 B), the lowest NC values were obtained for Adnaviria, Varidnaviria and Duplodnaviria (Table S4 and S5). These results are consistent with the genomic grouping since they are composed exclusively of dsDNA viruses and have the highest sequence lengths. Thus,

generally, an inverse correlation between genome size and NC was also observed as with the genome type analysis (Figure 3 A and B) and occurs across all taxonomic levels (Table S5). However, within these three Realms, Adnaviria has the lowest sequence length and presented a higher compressibility than Varidnaviria and Duplodnaviria, suggesting that the last are highly complex.

Regarding IRs, Adnaviria was the realm where the highest compression was obtained using the $IR_2$ subprogram (highest rate of IRs; Table S6). Consequently, its only recognized kingdom, Zilligvirae, has also one of the highest NCC values (Table S6). Adnaviria is a realm constituted of mostly A-form dsDNA viruses, and the ends of their genomes contain ITRs [105]. A-form is proposed to be an adaptation allowing DNA survival under extreme conditions since their hosts are hyperthermophiles and acidophiles microorganisms from the archaea domain [105, 106]. The fact that Adnaviria presented the lowest NC might indicate that their genomes require redundancy to survive such extreme environments. The kingdom Trapavirae, belonging to the realm Monodnaviria, is also composed by dsDNA viruses that infect halophilic archaea. Together with kingdom Zilligvirae, Trapavirae presented the highest difference between IRs and standard compression (Table S7). These results also support the fact that IRs can stabilize the DNA of viruses that exist in extreme environments. It has already been demonstrated that archaeal viruses with linear genomes use diverse solutions for protection and replication of the genome ends, such as including covalently closed hairpins and terminal IRs [107].

At the family level, Botourmiaviridae presented the highest complexity, followed by Alphasatellitidae and Tolecusatellitidae families (Table S5). Botourmiaviridae is composed of ssRNA viruses that infect plants, and filamentous fungi [108]. Curiously, plants and fungi have higher redundancy despite the lower redundancy of their pathogens. Alphasatellitidae and Tolecusatellitidae are families of satellite viruses that depend on the presence of another virus (helper viruses) to replicate their genomes. These satellite viruses have minimal genomes, making sense that they possess very low redundancy. Regarding IRs, Malacoherpesviridae, Herpesviridae, and Rudiviridae contained the highest $NC_{IR_0} - NC_{IR_1}$ difference (Table S7). Malacoherpesviridae and Herpesviridae are dsDNA viruses evolutionarily close since they belong to the order Herpesvirales [109]. Malacoherpesviridae encompasses the genera Aurivirus and Ostreavirus, which infect molluscs. Herpesviridae are also known as herpesviruses and have reptiles, birds and mammals as hosts. This family will be discussed in more detail in the following subsection. Rudiviridae is a family of viruses with linear dsDNA genomes that also infect archaea. The virus of these families are highly thermostable and can act as a template for site-selective and spatially controlled chemical modification. Furthermore, the two strands of the DNA are covalently linked at both ends of the genomes, which have long ITRs [110]. Again, these IRs could be an adaptation to stabilize the genome.

### *Complexity landscape of the family Herpesviridae*

Here we analyzed the complexity landscape of the genera of the family Herpesviridae in more detail, and results show a significant variation between them (Figure 4 A). Mardivirus had the highest $NC_{IR_0} - NC_{IR_1}$ difference among all viruses, and only other three genera (out of thirteen) of herpesviruses were within the ten highest differences list (Table S7). Indeed, the genus Mardivirus had the highest compression, whereas the genus Lymphocryptovirus possessed very low compression with the $IR_2$ subprogram. We performed the minimal bidirectional complexity profiles of one sequence of each virus to visualize their distribution of complexity locally (Figure 4 C). As we can see, Human herpesvirus 4 (also known as Epstein-Barr virus) has more internal repeats (Figure 4 C, $IR_0$ profile) detected and fewer IRs (Figure 4 B; $IR_2$ profile). The opposite occurs with the Falconid herpesvirus-1 strain S-18, where IRs are more prominent than internal repetitions. Furthermore, notice that these regions determined with compression profiles coincide with actual regions detected in the genome with other methods (Figure 4 C; first profile).

A particular group of family Herpesviridae are the human herpesviruses (HHVs). These viruses are involved in globally prevalent infections and cancers and characterized by lifelong persistence with reactivations that can potentially manifest life-threatening conditions [111]. Globally, the HHVs present a higher redundancy relative to other viruses (Figure 4 B). These viruses are divided into: i) the alpha-subfamily members, namely herpes simplex virus type 1 and 2 (HSV-1 and HSV-2) and varicella-zoster virus (VZV), ii) the beta-subfamily of human cytomegalovirus (HCMV) and human herpesviruses 6A, 6B, and 7 (HHV-6A, HHV-6B, and HHV-7) and iii) the gamma-subfamily of Epstein-Barr virus (EBV) and Kaposi's sarcoma-associated herpesvirus (KSHV). Specifically, the EBV, one of the most potent cell transformation and growth-inducing viruses known, capable of *immortalizing* human B lymphocytes, contains a higher redundancy than the other HHVs (Figure 4 B). The other gamma-herpesvirus, KSHV, is the genome with the highest $NC_{IR_1}$ (Figure 4 B). Unlike the beta- and gamma-subfamilies, the alpha-subfamily is characterized by a substantial quantity of IRs, as suggested by the NCs with $IR_1$ and $IR_2$ configurations (Figure 4 B). The VZV has the shortest genome and the highest NC within this group. These differences might be justified by the different rates of evolution within these genomes [112]. Considering the beta-subfamily members, HCMV contains a small proportion of IRs while having a substantial-high NC relative to other HHVs being analyzed. Since the HCMV has the largest genome, this was surprising because the NC typically has an inverse correlation with the genome size and the quantity of IRs. The other beta-subfamily members are the Human Herpesvirus 6A, 6B, and 7, which produced lower NCs (with $IR_1$ and $IR_2$ configurations) compared to the other HHVs, with a low quantity of IRs, an effect that their integrating function might favour. For instance, HHV-6A and 6B can integrate their genomes into the telomeres of latently infected cells [113, 114]. Thus, their genomes contain subsequences similar to the human telomere regions that can be formed by internal nucleotide repetitions [115]. As such, these are sequences with very low complexity and, hence, highly compressible.

### *Alternative visualization methods of the viral complexity landscape*

Cladograms were generated depicting the redundancy (NC; Figure 5 A) and the prevalence of inverted repeats (NCC; Figure 5 B) on each taxonomic branch. In addition, we performed the same analysis to portray the relation between inverted and internal repetitions (Figure S3). These cladograms show the broad picture of the regions with more complex and less redundant sequences, regions rich in inverted repeats, and regions with a higher prevalence of inverted repeats relative to standard repetitions in the genomes.

Another way to analyze the results is by producing 3D-scatter plots of randomly sampled values obtained from computing the features sequence length (SL), NC and GC-content (GC; Figure 6 A) or 2D-scatter plots of their projections (Figure 6 B and 6 C), both concerning a particular taxonomic level (herein Realm). Analyzing the sequence length projections (Figure 6 B), it is evident that there is a logarithmic downtrend of the NC with the increase in sequence length. Thus, although longer

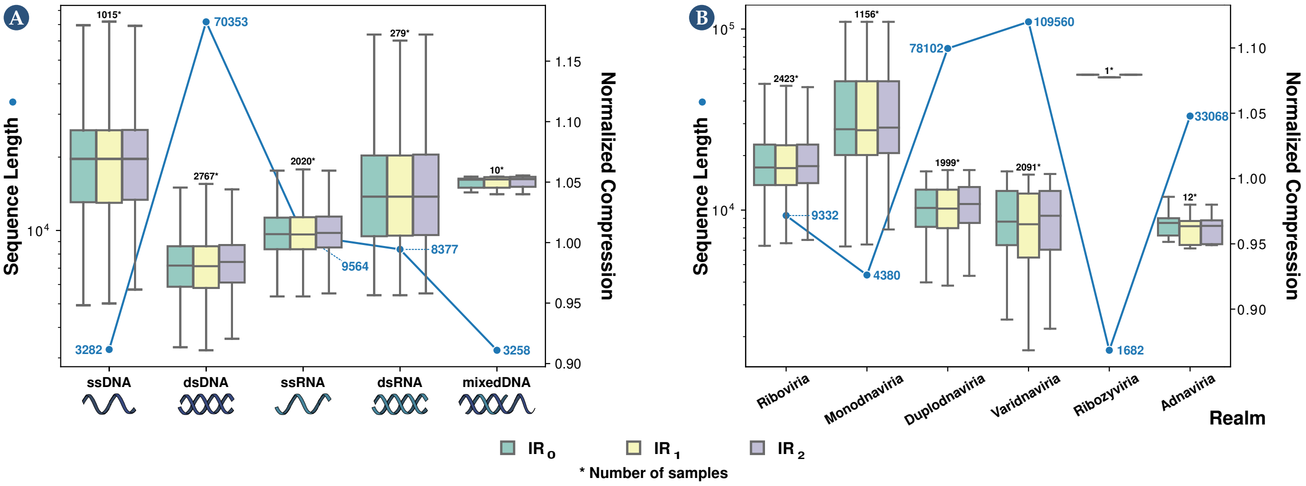


**Figure 3.** Average Normalized Compression (ANC) and average sequence length per viral group. The values were obtained for genome type (*A*) and realm (*B*). To view all boxplots by groups of realm, kingdom, phylum, class, order, family, and genus, please visit the website https://asilab.github.io/canvas/.

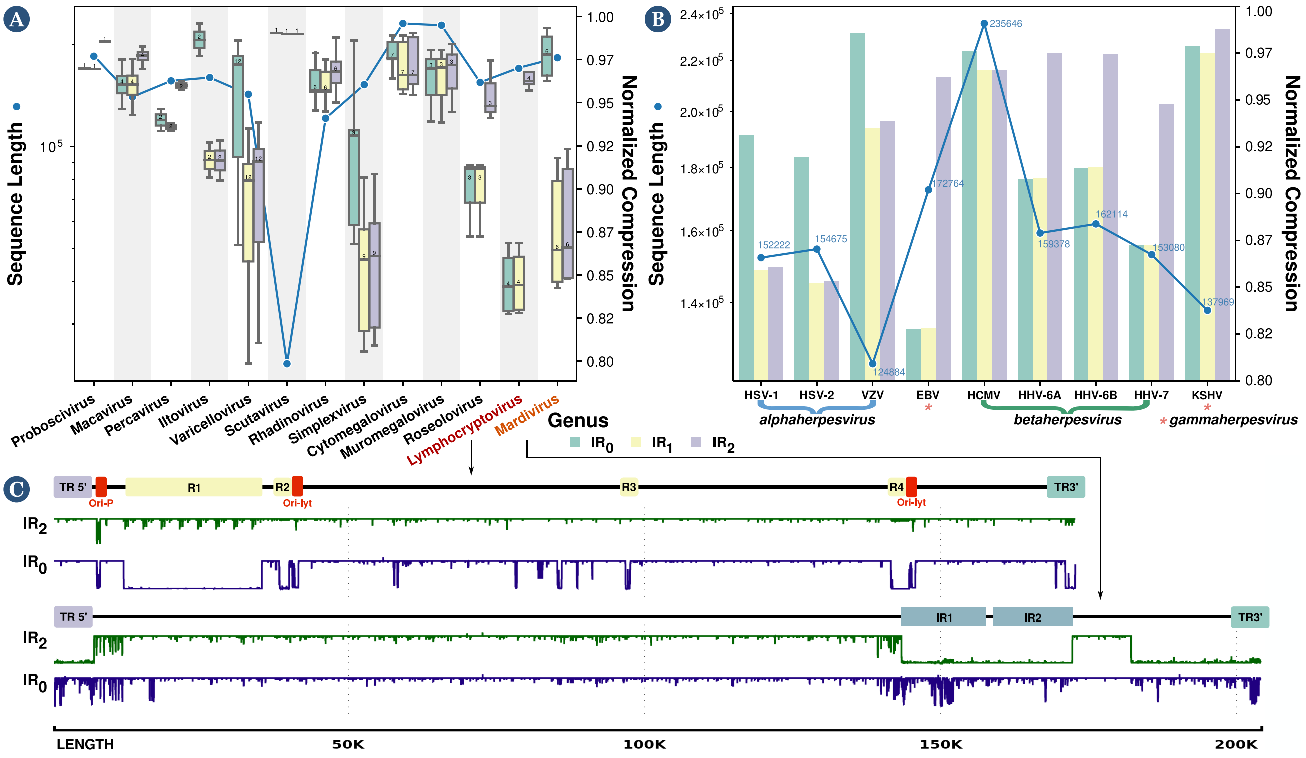


**Figure 4.** Average Normalized Compression (ANC) and average sequence length per the genera of the Herpesviridae family (**A**) and for various Human Herpesviruses (**B**). In the boxplot where the genera of the Herpesviridae family are displayed, two genera were selected, one with a low level of inverted repeats (Lymphocryptovirus) and one with a high (Mardivirus). Then, a representative reference sequence was selected (Lymphocryptovirus - Human herpesvirus 4 or EBV, NCBI Reference Sequence: *NC*_024450.1; Mardivirus - Falconid herpesvirus 1 strain S-18, NCBI Reference Sequence: *NC*_009334.1) and created minimal bi-directional complexity profiles (**C**).

sequences have, on average, greater complexity (absolute quantities), they have higher redundancy, which the data compressor takes advantage of to perform a better compression. On the other hand, the NC vs the GC-content displays a normal distribution around the 0.5 GC-mark, with higher complexities associated with similar frequency of occurrence of the four bases A, C, G, T/U (Figure 6 C). This result also makes sense since, in principle, a well-distributed frequency of bases makes more complex sequences to compress. More importantly, the NC, GC and SL seem to discriminate between different taxonomic groups (Figure 6). As such, in the following section, we analyze the classification capability of these features.

## Viral Classification

Although sequence alignment is essential for genomic analysis, the fact that pairwise and multiple alignment methods are often slow methods led to the popularization of fast alignment-free methods for sequence comparison. Most alignment-free methods are based on word frequencies for words of a fixed length or word-matching statistics. Others use the length of maximal word matches, and others rely on spaced-word matches (SpaM). These inexact word matches allow mismatches at certain pre-defined positions and can accurately estimate phylogenetic distances between DNA or protein sequences using a stochastic model of molecular evolution [116].

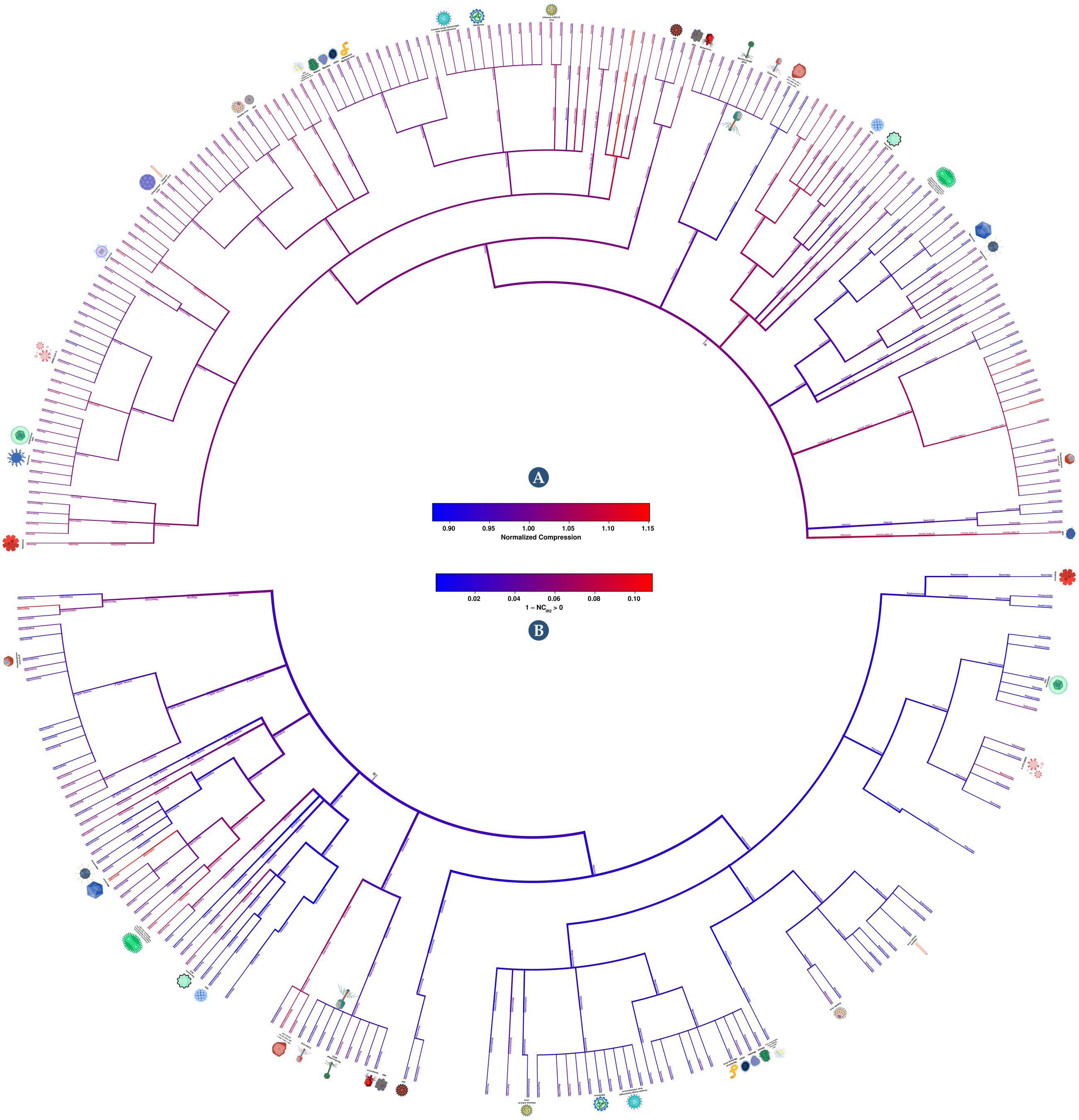


**Figure 5.** Cladograms showing average NC of each viral group (**A**), and the normalized compression capacity (*NCC*) (**B**). *NCC* results were obtained by $NCC = 1 - NC_{IR_2} > 0$. The colour red depicts the highest complexity, and the blue the lowest. The first cladogram describes the NC of each taxonomic branch. Red colour show genomes with less redundancy, and blue ones with more redundancy. On the other hand, the second cladogram depicts the prevalence of inverted repeats on each taxonomic branch. Red indicates branches with genomes with a high percentage of inverted repeats, whereas blue shows branches with a low percentage. For better visualization, please visit the website https://asilab.github.io/canvas/.

This approach has also been updated as the Multiple Spaced-Word Matches (Multi-SpaM) method, which is based on multiple sequence comparison and maximum likelihood [117]. Regarding viral sequences, many studies were performed on alignment-free sequence comparison and classification. For instance, Garcia et al. [118] developed a dynamic programming algorithm for creating a classification tree using metagenome viruses. For the classification tree creation, k-mer profiles of each metagenome virus were created, and proportional similarity scores were generated and clustered. Using the JGI metagenomic and NCBI databases, the authors were able to identify the correct virus (including its parent in the classification tree) 82% of the time. Zhang et al. [119] created an alignment-free method that employed k-mers as genomic features for a large-scale comparison of complete viral genomes. After determining the optimal k for all 3,905 complete viral genomes, a dendrogram was created, which shows consistency with the viral taxonomy of the ICTV and the Baltimore classification of viruses. He et al. [120] proposed an alignment-free sequence comparison method for viral genomes based on the location correlation coefficient. When applied to the evolutionary analysis of the common human viruses, including SARS-CoV-2, Dengue virus, Hepatitis B virus, and human rhinovirus and achieves the same or even better results than alignment-based methods. Finally, Huang et al. [121] proposed a classification method based on discriminant analysis employing the first and second moments

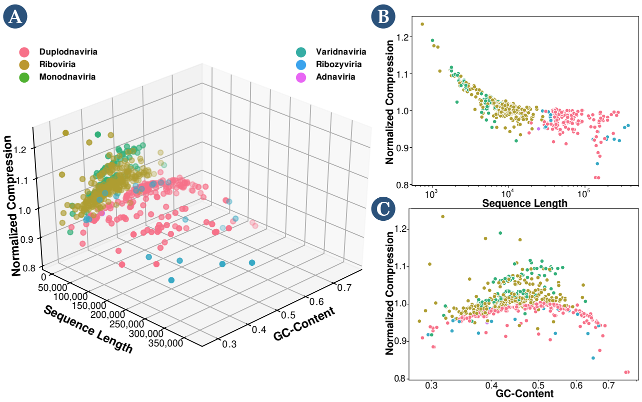


**Figure 6.** Scatter-plots of Normalized Compression vs. Sequence Length and GC-Content (**A**), Scatter-plots of Normalized Compression vs. Sequence Length (**B**) and Normalized Compression vs. GC-Content (**C**).

of positions of each nucleotide of the genome sequences as features and performed classification of genomes regarding their Baltimore classification and family (12 families) and obtained a maximum value of accuracy of 88.65% and 85.91%, respectively.

With these considerations in mind, we created an alignment-free feature-based classification method in this section. We performed eight different classification tasks for each viral sequence from the dataset. Specifically, the sequences were classified regarding their genome type, realm, kingdom, phylum, class, order, family, and genus.

We conducted a random 80-20 train-test split on the dataset to perform viral classification. Due to classes being imbalanced in the dataset, we performed several actions. First, we did not consider classes with less than four samples. As such, depending on the classification task, the number of samples decreased from 6,091 to the values shown in Table S8 (N. Classes column). Secondly, we performed the train-test split in a stratified way to ensure the representability of each label in the train and test sets. Finally, instead of performing k-fold cross-validation, we performed the random train-test split fifty times, and we retrieved the average of the evaluation metrics. Then, we computed the Accuracy and the Weighted F1-score to select the best performing method.

Considering these works, herein we perform feature-based classification. As described in the method section, we applied 5 types of classifiers: Linear Discriminant Analysis (LDA) [99], Gaussian Naive Bayes (GNB) [100], K-Nearest Neighbors (KNN) [101], Support Vector Machine (SVM) [102] and XGBoost classifier (XGB) [103].

Furthermore, we performed classification using seven different features: sequence length (SL), GC-content (GC), the Normalized Compression (NC) values for the best performing model, and the NC of the same model with IR configuration to 0, 1 and 2.

These seven features were fed to all the classifiers, and the accuracy and weighted F1-score were measured to determine which classifier was best suited for this task.

Tables S8 and S9 depict the accuracy and weighted F1-score values obtained for each classifier. For all classification tasks, the best performing classifier was the XGBoost classifier.

Following this, we analyzed if all features were necessary. For that purpose, the XGBoost classifier was used with only the NC feature, the NC with SL and GC, and finally, using all features. The obtained accuracies are shown in Table 1, and the weighted F1-score results are shown in Table S10. The best results are obtained when using all features. This improvement increased when the number of classes was higher, demonstrating that the different compression subprograms ($IR_0$, $IR_1$, and $IR_2$) are more helpful in classifying more specific taxonomic groups.

The results show a decrease in accuracy and F1-score when there is an increase in the number of classes. Specifically, we obtained the best performance in the realm classification of the virus (accuracy - 92.57%, F1-score - 0.9234) and our lowest performance in genus classification (accuracy - 68.71%, F1-score - 0.6561). This decrease is mainly because the average number of samples per class decreases as the number of classes increases. As such, many classes may still have an insufficient number of samples to be accurately classified. Figure S4 represents the number of samples (genome sequences) per viral genus. Furthermore, part of the classification inaccuracies can be explained by possible errors in the assembly process of the original sequence or eventual sub-sequence contamination of parts of the genomes. Moreover, other inaccuracies could be due to several genomes being reconstructed using older methods that have been improved since then [122].

Despite being pertinent, the alignment-free studies are not directly comparable due to sample size, absence of classification metrics and source code. Furthermore, the method proposed in this work is not only alignment-free but also feature-based, providing a higher level of flexibility since it does not resort directly to the reference genomes but instead to features that the biological sequences share. Therefore, we compared our results with the outcome obtained using a random classifier as a measure of comparison. Specifically, for each task, we determined the probability of a random sequence being correctly classified ($p_{hit}$). Overall there is a vast improvement relative to the random classifier, showing the importance of the features used in the classification process. These classification results seem promising, showing that this metric can be utilized for viral taxonomic classification if enough sequence samples are provided.

## Discussion

The usage of a specialized compressor is crucial to accurately quantify the complexity present in a genome and detect the intrinsic algorithmic nature of the data. Genomic data is highly heterogeneous and has high substitution mutations and data rearrangements, such as fusions, translocations, and inversions [64, 65]. Therefore, the ability of a genomic data compressor to adapt to this heterogeneous data, being able to perform an accurate structure modelling and detect repetitions in the presence of the high substitutional mutations and rearrangements in genomic data is fundamental to achieve high compressibility of the genome sequence. This article evaluates the capacity to identify data-specific patterns in genomic sequences by comparing the potential of three methods to recognize IRs. Precisely, the NBDM was estimated, and the NC was computed using a genomic compressor (GeCo3 [84]) and a general-purpose data-compressor (cix and PAQ8 [86, 87]). When GeCo3 had the subprogram activated that detects IRs ($NC_{IR_2}$), it showed substantially higher compression than general-purpose because cmix and PAQ use models that do not consider these specific properties of the genomic sequences. The same occurs when comparing GeCo3 ($NC_{IR_2}$) with NBDM, showing that despite NBDM being able to detect small subprograms in synthetic data [56], it cannot detect IRs in genomic data. Moreover, GeCo3 compression capability was resistant to substitutional mutation up to 10%, showing that it can also deal with this extreme nature of genomic data, namely

**Table 1.** Results obtained for viral taxonomic classification task regarding the genome type, realm, kingdom, phylum, class, order, family, and genus using XGBoost classifier. The features used were the genome's sequence length (SL), the GC-content (GC) and the Normalized Compression (NC) values for the best model, the same model with IR configuration to 0, to 1 and 2. The results correspond to the accuracy (ACC), and the probability of a random sequence being correctly classified ($p_{hit}$) using a random classifier ($p_{hit}(C_{Random})$.

| Classification | N. Classes | N. Samples | $p_{hit}(C_{Random})$ | $ACC_{NC}$ | $ACC_{NC+GC}$ | $ACC_{NC+SL+GC}$ | $ACC_{All\ without\ SQ}$ | $ACC_{All\ Features}$ |
|---|---|---|---|---|---|---|---|---|
| **Genome** | 5 | 6089 | 20.00 | 75.57 | 80.60 | 87.11 | 81.24 | **87.25** |
| **Realm** | 5 | 5799 | 20.00 | 77.90 | 84.56 | 92.25 | 86.16 | **92.57** |
| **Kingdom** | 10 | 5788 | 10.00 | 76.44 | 82.51 | 90.82 | 84.06 | **90.96** |
| **Phylum** | 17 | 5778 | 5.88 | 63.97 | 70.69 | 82.36 | 73.21 | **83.41** |
| **Class** | 34 | 5845 | 2.94 | 59.83 | 65.90 | 79.05 | 68.66 | **80.23** |
| **Order** | 48 | 5838 | 2.08 | 58.44 | 65.08 | 78.20 | 67.88 | **79.62** |
| **Family** | 102 | 5990 | 0.98 | 43.35 | 54.06 | 72.46 | 58.34 | **74.46** |
| **Genus** | 360 | 4673 | 0.28 | 35.59 | 50.02 | 67.32 | 54.23 | **68.71** |

approximate IRs.

On average, RNA viruses mutate faster than DNA viruses, double-strand viruses mutate slower than single-stranded viruses, and genome size correlates negatively with mutation rate [123]. In this article, we have shown that the redundancy of dsDNA is higher than ssDNA, but for RNA viruses, the opposite occurs. The sequences used in this study to measure a lower NC (higher normalized redundancy) of the ssRNA to dsRNA have approximately the same length. However, the dataset of dsRNA has less than one order of magnitude in the number of sequences. This difference is natural since the ssRNA is much more abundant than dsRNA. Nevertheless, this discrepancy could justify the higher normalized redundancy of ssRNA in the first instance. However, although the lower average NC values of ssRNA are similar to dsRNA, the dsRNA has higher NC extremes. Therefore, we argue that this difference in the number of sequences in the dsRNA is not significant in changing the lower average of the ssRNA. Also, ssRNA are more prone to mutation than dsRNA [124]. On the other hand, extensive C to U mutations have been reported in many mammalian RNA viruses [95]. This behaviour was detected during a much faster evolution of the SARS-CoV- 2, an ssRNA virus [125]. Therefore, the faster average decrease of GC-content in ssRNA viruses explains a decrease in the ssRNA entropy and, hence, average NC. A higher GC-content (approximately 2%) of the dsRNA over ssRNA strengthens these outcomes (Table S3).

We performed an analysis of the human herpesvirus regarding their genome complexity and IRs abundance. Specifically, we analyzed the various behaviours of their subfamilies and identified that different complexities could be representative of the different rates of evolution within these genomes. Finally, we suggest that maybe a higher compressibility and abundance of inversions present in herpesvirus are associated with viral genome integration.

Lastly, we evaluated the capability of using complexity measures to perform viral classification at different taxonomic levels. Notably, results showed that we can automatically and accurately distinguish between viral genomes at different taxonomic levels using the XGBoost classifier with all features (NC with different configurations, GC-content and SL). However, a decrease in accuracy when approaching the lowest taxonomic levels was observed, which can be increased with future entries to the database. Furthermore, when analysing viral sequences from environmental samples or integrated genome samples, the length of the original viral genome is often not known. Therefore, we computed the accuracy of a model that does not include this feature. Although we obtain a lower accuracy and F1-score, the results indicate that the method is still reliable for fast and efficient viral taxonomic identification in these scenarios.

Finally, despite the high accuracy results obtained, further improvement of the results may be possible in the classification by adding the transcribed viral proteome information.

## Conclusion

This manuscript shows that the efficient approximation of the Kolmogorov complexities of viral sequences as measures that quantify the absence of redundancy have a profound impact on genomes identification, classification, and organization.

For computing an upper bound of the sequence complexity, we benchmark a specific data compressor (GeCo3), after optimization, against other approaches. Specifically, GeCo3 was compared with high compression ratio general-purpose data compressors (PAQ and cmix) and a measure that combines small algorithmic programs and Shannon entropy (BDM). Unlike the other approaches, we show that GeCo3 can efficiently address and quantify regions properly described by simple algorithmic sources, namely inverted repeats (exact and approximate), among other characteristics.

Using an optimized compression level of GeCo3 in an extensive viral dataset, we provide a comprehensive landscape of the viral genome's complexity, comparing the viral genomes at several taxonomic levels while identifying the genome regarding the lowest and highest proportion of complexity. Specifically, on average, dsDNA viruses are the most redundant (less complex) according to their size, and ssDNA viruses are the less redundant. Contrarily, dsRNA viruses show a lower redundancy relative to ssRNA viruses.

We perform an in-depth analysis of the human herpesvirus regarding their genome complexity and abundance of IRs. We suggest that a higher compressibility and abundance of inversions in herpesvirus may be associated with viral genome integration.

We describe and use minimal bi-directional complexity profiles of one sequence of each virus to visualize the distribution of complexity of these sequences locally. These profiles can describe actual regions detected in the genome with other methods, proving the description capability of data compression at a structural level.

We reveal the importance of efficient data compression in genome classification tasks, explicitly showing that the complexity, when combined with simple measures (GC-content and size), is efficient in accurately distinguishing between viral genomes at different taxonomic levels without using direct comparisons between sequences.

The methods and results presented in this work provide new frontiers for studying viral genomes' complexity while magnifying the importance of developing efficient data compression

methods for automatic and accurate viral analysis.

## Availability of source code and requirements

- Project name: C.A.N.V.A.S. (Complexity ANalysis of VirAl Sequences)
- Project home page: https://github.com/jorgeMFS/canvas
- Operating system(s): Linux
- Programming language: Bash; Python.
- Other requirements: Python v3.6; Conda v4.3.27.
- License: MIT License.
- RRID:SCR_022552.
- biotools:canvas1.

The reproduction guidelines are available in the Reproducibility section of the Supplementary Material.

## Availability of supporting data and materials

## Website

The website of this paper is available at https://asilab.github.io/canvas/. This site showcases, among other things, the pipeline of this study, the compressor's model selection, the detection of inverted repeats in synthetic genomic sequences, the viral genome characterization with regards to genome and type of taxonomic group, and the computed cladograms with a magnifier to allow a better observation of the normalized complexity results with illustrative examples of viruses. Snapshots of our code and other data further supporting this work are openly available in the GigaScience respository, GigaDB [126].

## Declarations

### List of abbreviations

A → adenine
ANC → Average Normalized Compression
BDM → Block Decomposition Method
C → cytosine
CTM → Coding Theorem Method
dsDNA → double-stranded deoxyribonucleic acid
dsRNA → double-stranded ribonucleic acid
EBV→ Epstein-Barr virus
G → guanine
GC → GC-Content
GNB → Gaussian Naive Bayes
HCMV→ human cytomegalovirus
HHVs→ human herpesviruses
HSV-1 → herpes simplex virus 1
HSV-2 → herpes simplex virus 2
IR → inverted repeats
K→ Kolmogorov complexity
KNN → K-Nearest Neighbors
KSHV→ Kaposi's sarcoma-associated herpesvirus
LDA → Discriminant Analysis
LSTM → Long Short-Term Memory
MBGC → Multiple Bacteria Genome Compressor
mRNA → messenger ribonucleic acid
NAF → Nucleotide Archival Format
NBDM → Normalized Block Decomposition Method
NC → Normalized Compression
NCC → normalized compression capacity
NR → normalized redundancy
R→ redundancy
RdRp → RNA-dependent RNA polymerase
SL → Sequence Length
SNP→ Single Nucleotide Polymorphis
ssDNA → single-stranded deoxyribonucleic acid
ssRNA → single-stranded ribonucleic acid
SVM → Support Vector Machine
T → thymine
TM → Turing machines
U → uracil
VZV→ varicella-zoster virus
XGB → XGBoost

### Competing Interests

The authors declare no competing interests.

### Funding

This work was partially funded by National Funds through the FCT - Foundation for Science and Technology, in the context of the project UIDB/00127/2020. J.M.S. acknowledges the FCT grant SFRH/BD/141851/2018. D.P. is funded by national funds through FCT – Fundação para a Ciência e a Tecnologia, I.P., under the Scientific Employment Stimulus - Institutional Call - reference CEECINST/00026/2018. T.C. is funded by national funds (OE), through FCT – Fundação para a Ciência e a Tecnologia, I.P., in the scope of the framework contract foreseen in the numbers 4, 5 and 6 of the article 23, of the Decree-Law 57/2016, of August 29, changed by Law 57/2017, of July (CEECIND/01463/2017). Thanks are due to FCT/MCTES for the financial support to CESAM (UIDP/50017/2020+UIDB/50017/2020), through national funds.

### Author's Contributions

J.M.S. and D.P. designed the experiment, executed data analysis and wrote the manuscript. All authors analysed and discussed the results and revised the manuscript.

## References

1. Hendrix RW, Hatfull GF, Ford ME, Smith MC, Burns RN. Evolutionary relationships among diverse bacteriophages and prophages: all the world's a phage. In: Horizontal gene transfer Elsevier; 2002.p. 133–VI.
2. O'Leary NA, Wright MW, Brister JR, Ciufo S, Haddad D, McVeigh R, et al. Reference sequence (RefSeq) database at NCBI: current status, taxonomic expansion, and functional annotation. Nucleic acids research 2016;44(D1):D733–D745.
3. Edwards RA, Rohwer F. Viral metagenomics. Nature Reviews Microbiology 2005;3(6):504–510.
4. Lawrence CM, Menon S, Eilers BJ, Bothner B, Khayat R, Douglas T, et al. Structural and functional studies of archaeal viruses. Journal of Biological Chemistry 2009;284(19):12599–12603.
5. Koonin EV, Senkevich TG, Dolja VV. The ancient Virus World and evolution of cells. Biology direct 2006;1(1):29.
6. Nayfach S, Roux S, Seshadri R, Udwary D, Varghese N, Schulz F, et al. A genomic catalog of Earth's microbiomes. Nature biotechnology 2021;39(4):499–509.
7. Fermin G. Virion Structure, Genome Organization, and Taxonomy of Viruses. Viruses 2018;p. 17.

8. Stern L, Allison L, Coppel RL, Dix TI. Discovering patterns in Plasmodium falciparum genomic DNA. Molecular and Biochemical Parasitology 2001;118(2):175–186.
9. Cao MD, Dix TI, Allison L. A genome alignment algorithm based on compression. BMC bioinformatics 2010;11(1):1–16.
10. Hayashida M, Akutsu T. Comparing biological networks via graph compression. In: BMC systems biology, vol. 4 BioMed Central; 2010. p. 1–11.
11. Bywater RP. Prediction of protein structural features from sequence data based on Shannon entropy and Kolmogorov complexity. PloS one 2015;10(4):e0119306.
12. Pratas D, Pinho AJ. On the approximation of the Kolmogorov complexity for DNA sequences. In: Iberian Conference on Pattern Recognition and Image Analysis Springer; 2017. p. 259–266.
13. Pratas D, Pinho AJ. Metagenomic composition analysis of sedimentary ancient DNA from the Isle of Wight. In: 2018 26th European Signal Processing Conference (EUSIPCO) IEEE; 2018. p. 1177–1181.
14. Hosseini M, Pratas D, Morgenstern B, Pinho AJ. Smash++: an alignment-free and memory-efficient tool to find genomic rearrangements. GigaScience 2020;9(5):giaa048.
15. Editorial. Microbiology by numbers. Nature Reviews Microbiology 2011;9:628.
16. Claverie JM, Ogata H, Audic S, Abergel C, Suhre K, Fournier PE. Mimivirus and the emerging concept of "giant" virus. Virus research 2006;117(1):133–144.
17. Claverie JM, Abergel C, Ogata H. Mimivirus. In: Lesser Known Large dsDNA Viruses Springer; 2009.p. 89–121.
18. Foster JE, Fermin G. Chapter 4 - Origins and Evolution of Viruses. In: Tennant P, Fermin G, Foster JE, editors. Viruses Academic Press; 2018.p. 83–100.
19. Amorim A, Pereira F, Alves C, García O. Species assignment in forensics and the challenge of hybrids. Forensic Science International: Genetics 2020;48:102333.
20. Martin W, Koonin EV. Introns and the origin of nucleus–cytosol compartmentalization. Nature 2006;440(7080):41–45.
21. Cavalier-Smith T. Origin of the cell nucleus, mitosis and sex: roles of intracellular coevolution. Biology direct 2010;5(1):7.
22. Takemura M. Medusavirus Ancestor in a Proto-Eukaryotic Cell: Updating the Hypothesis for the Viral Origin of the Nucleus. Frontiers in Microbiology 2020;11:2169.
23. Toppinen M, Sajantila A, Pratas D, Hedman K, Perdomo MF. The Human Bone Marrow Is Host to the DNAs of Several Viruses. Frontiers in Cellular and Infection Microbiology 2021;11.
24. Toppinen M, Pratas D, Väisänen E, Söderlund-Venermo M, Hedman K, Perdomo MF, et al. The landscape of persistent human DNA viruses in femoral bone. Forensic Science International: Genetics 2020;48:102353.
25. Ikegaya H, Iwase H. Trial for the geographical identification using JC viral genotyping in Japan. Forensic science international 2004;139(2-3):169–172.
26. Agostini HT, Yanagihara R, Davis V, Ryschkewitsch CF, Stoner GL. Asian genotypes of JC virus in Native Americans and in a Pacific Island population: markers of viral evolution and human migration. Proceedings of the National Academy of Sciences 1997;94(26):14542–14546.
27. Sugimoto C, Kitamura T, Guo J, Al-Ahdal MN, Shchelkunov SN, Otova B, et al. Typing of urinary JC virus DNA offers a novel means of tracing human migrations. Proceedings of the National Academy of Sciences 1997;94(17):9191–9196.
28. Sugimoto C, Hasegawa M, Zheng HY, Demenev V, Sekino Y, Kojima K, et al. JC virus strains indigenous to northeastern Siberians and Canadian Inuits are unique but evolutionally related to those distributed throughout Europe and Mediterranean areas. Journal of Molecular Evolution 2002;55(3):322–335.
29. Forni D, Cagliani R, Clerici M, Pozzoli U, Sironi M. You will never walk alone: codispersal of JC polyomavirus with human populations. Molecular biology and evolution 2020;37(2):442–454.
30. Senior AW, Evans R, Jumper J, Kirkpatrick J, Sifre L, Green T, et al. Protein structure prediction using multiple deep neural networks in the 13th Critical Assessment of Protein Structure Prediction (CASP13). Proteins: Structure, Function, and Bioinformatics 2019;87(12):1141–1148.
31. Senior AW, Evans R, Jumper J, Kirkpatrick J, Sifre L, Green T, et al. Improved protein structure prediction using potentials from deep learning. Nature 2020;577(7792):706–710.
32. Hosseini M, Pratas D, Pinho AJ. On the role of inverted repeats in DNA sequence similarity. In: International Conference on Practical Applications of Computational Biology & Bioinformatics Springer; 2017. p. 228–236.
33. Toppinen M, et al. Parvoviral genomes in human soft tissues and bones over decades. PhD thesis, Helsingin yliopisto; 2021.
11. Peck KM, Lauring AS. Complexities of viral mutation rates. Journal of virology 2018;92(14):e01031–17.
35. Voineagu I, Narayanan V, Lobachev KS, Mirkin SM. Replication stalling at unstable inverted repeats: interplay between DNA hairpins and fork stabilizing proteins. Proceedings of the National Academy of Sciences 2008;105(29):9936–9941.
36. Bissler JJ. DNA inverted repeats and human disease. Front Biosci 1998;3(4):d408–d418.
37. Lin CT, Lin WH, Lyu YL, Whang-Peng J. Inverted repeats as genetic elements for promoting DNA inverted duplication: implications in gene amplification. Nucleic Acids Research 2001;29(17):3529–3538.
38. Atkins JF, Loughran G, Bhatt PR, Firth AE, Baranov PV. Ribosomal frameshifting and transcriptional slippage: From genetic steganography and cryptography to adventitious use. Nucleic acids research 2016;44(15):7007–7078.
39. Namy O, Moran SJ, Stuart DI, Gilbert RJ, Brierley I. A mechanical explanation of RNA pseudoknot function in programmed ribosomal frameshifting. Nature 2006;441(7090):244–247.
40. Mikl M, Pilpel Y, Segal E. High-throughput interrogation of programmed ribosomal frameshifting in human cells. Nature communications 2020;11(1):1–18.
41. Cotmore SF, Tattersall P. Parvoviruses: small does not mean simple. Annual review of virology 2014;1:517–537.
42. Yan Z, Zak R, Zhang Y, Engelhardt JF. Inverted terminal repeat sequences are important for intermolecular recombination and circularization of adeno-associated virus genomes. Journal of virology 2005;79(1):364–379.
43. Byrne D, Grzela R, Lartigue A, Audic S, Chenivesse S, Encinas S, et al. The polyadenylation site of Mimivirus transcripts obeys a stringent 'hairpin rule'. Genome research 2009;19(7):1233–1242.
44. Claverie JM, Abergel C. Mimivirus and its virophage. Annual review of genetics 2009;43:49–66.
45. Solomonoff RJ. A formal theory of inductive inference. Part I. Information and control 1964;7(1):1–22.
46. Solomonoff RJ. A formal theory of inductive inference. Part II. Information and control 1964;7(2):224–254.
47. Kolmogorov AN. Three approaches to the quantitative definition of information'. Problems of information transmission 1965;1(1):1–7.

48. Chaitin GJ. On the length of programs for computing finite binary sequences. Journal of the ACM (JACM) 1966;13(4):547–569.
49. Hammer D, Romashchenko A, Shen A, Vereshchagin N. Inequalities for Shannon entropy and Kolmogorov complexity. Journal of Computer and System Sciences 2000;60(2):442–464.
50. Henriques T, Gonçalves H, Antunes L, Matias M, Bernardes J, Costa-Santos C. Entropy and compression: two measures of complexity. Journal of Evaluation in Clinical Practice 2013;19(6):1101–1106.
51. Soler-Toscano F, Zenil H, Delahaye JP, Gauvrit N. Calculating Kolmogorov complexity from the output frequency distributions of small Turing machines. PloS one 2014;9(5).
52. Zenil H, Hernández-Orozco S, Kiani NA, Soler-Toscano F, Rueda-Toicen A, Tegnér J. A decomposition method for global evaluation of Shannon entropy and local estimations of algorithmic complexity. Entropy 2018;20(8):605.
53. Zenil H, Soler-Toscano F, Dingle K, Louis AA. Correlation of automorphism group size and topological properties with program-size complexity evaluations of graphs and complex networks. Physica A: Statistical Mechanics and its Applications 2014;404:341–358.
54. Kempe V, Gauvrit N, Forsyth D. Structure emerges faster during cultural transmission in children than in adults. Cognition 2015;136:247–254.
55. Zenil H, Soler-Toscano F, Delahaye JP, Gauvrit N. Two-dimensional Kolmogorov complexity and an empirical validation of the Coding theorem method by compressibility. PeerJ Computer Science 2015;1:e23.
56. Silva JM, Pratas D, Antunes R, Matos S, Pinho AJ. Automatic analysis of artistic paintings using information-based measures. Pattern Recognition 2021;114:107864.
57. Li M, Vitányi P, et al. An introduction to Kolmogorov complexity and its applications, vol. 3. Springer; 2008.
58. Bloem P, Mota F, de Rooij S, Antunes L, Adriaans P. A safe approximation for Kolmogorov complexity. In: International Conference on Algorithmic Learning Theory Springer; 2014. p. 336–350.
59. Dougherty ER, Shmulevich I. Genomic signal processing and statistics, vol. 2. Hindawi Publishing Corporation; 2005.
60. Gailly J, Adler M. The gzip home page; accessed May 16, 2020, http://www.gzip.org/.
61. bzip2; accessed May 16, 2020, http://www.bzip.org/.
62. Pavlov I. 7-Zip; accessed May 16, 2020, https://www.7-zip.org/.
63. Grumbach S, Tahi F. Compression of DNA sequences. In: [Proceedings] DCC93: Data Compression Conference IEEE; 1993. p. 340–350.
64. Rieseberg LH. Chromosomal rearrangements and speciation. Trends in ecology & evolution 2001;16(7):351–358.
65. Roeder GS, Fink GR. DNA rearrangements associated with a transposable element in yeast. Cell 1980;21(1):239–249.
66. Hernaez M, Pavlichin D, Weissman T, Ochoa I. Genomic data compression. Annual Review of Biomedical Data Science 2019;2:19–37.
67. Grumbach S, Tahi F. A new challenge for compression algorithms: genetic sequences. Information Processing & Management 1994;30(6):875–886.
68. Manzini G, Rastero M. A simple and fast DNA compressor. Software: Practice and Experience 2004;34(14):1397–1411.
69. Cherniavsky N, Ladner R. Grammar-based compression of DNA sequences. DIMACS Working Group on The Burrows-Wheeler Transform 2004;21.
70. Korodi G, Tabus I. An efficient normalized maximum likelihood algorithm for DNA sequence compression. ACM Transactions on Information Systems (TOIS) 2005;23(1):3–34.
71. Vey G. Differential direct coding: a compression algorithm for nucleotide sequence data. Database 2009;2009.
72. Mishra KN, Aaggarwal A, Abdelhadi E, Srivastava D. An efficient horizontal and vertical method for online DNA sequence compression. International Journal of Computer Applications 2010;3(1):39–46.
73. Rajeswari PR, Apparao A. GENBIT Compress-Algorithm for repetitive and non repetitive DNA sequences. International Journal of Computer Science and Information Technology 2010;2:25–29.
74. Gupta A, Agarwal S. A novel approach for compressing DNA sequences using semi-statistical compressor. International Journal of Computers and Applications 2011;33(3):245–251.
75. Zhu Z, Zhou J, Ji Z, Shi YH. DNA sequence compression using adaptive particle swarm optimization-based memetic algorithm. IEEE Transactions on Evolutionary Computation 2011;15(5):643–658.
76. Pinho AJ, Ferreira PJ, Neves AJ, Bastos CA. On the representability of complete genomes by multiple competing finite-context (Markov) models. PloS one 2011;6(6):e21588.
77. Pratas D, Pinho AJ, Ferreira PJ. Efficient compression of genomic sequences. In: 2016 Data Compression Conference (DCC) IEEE; 2016. p. 231–240.
78. Kryukov K, Ueda MT, Nakagawa S, Imanishi T. Nucleotide Archival Format (NAF) enables efficient lossless reference-free compression of DNA sequences. Bioinformatics 2019;35(19):3826–3828.
79. Kryukov K. Kirillkryukov/NAF: Nucleotide archival format - compressed file format for DNA/RNA/protein sequences; accessed May 5, 2022, https://github.com/KirillKryukov/naf.
80. Grabowski S, Kowalski TM. MBGC: Multiple Bacteria Genome Compressor. GigaScience 2022;11.
81. Knoll B. Byronknoll/cmix: Cmix is a lossless data compression program aimed at optimizing compression ratio at the cost of high CPU/memory usage.Byron Knoll; accessed May 5, 2022, https://github.com/byronknoll/cmix.
82. Cao MD, Dix TI, Allison L, Mears C. A simple statistical algorithm for biological sequence compression. In: 2007 Data Compression Conference (DCC'07) IEEE; 2007. p. 43–52.
83. Pratas D, Hosseini M, Silva JM, Pinho AJ. A reference-free lossless compression algorithm for DNA sequences using a competitive prediction of two classes of weighted models. Entropy 2019;21(11):1074.
84. Silva M, Pratas D, Pinho AJ. Efficient DNA sequence compression with neural networks. GigaScience 2020 11;9(11). Giaa119.
85. Kryukov K, Ueda MT, Nakagawa S, Imanishi T. Sequence Compression Benchmark (SCB) database—A comprehensive evaluation of reference-free compressors for FASTA-formatted sequences. GigaScience 2020;9(7):giaa072.
86. Knoll B, de Freitas N. A machine learning perspective on predictive coding with PAQ8. In: 2012 Data Compression Conference IEEE; 2012. p. 377–386.
87. Buchner AJ. PAQ; accessed May 16, 2020, https://github.com/JohannesBuchner/paq/.
88. Hochreiter S, Schmidhuber J. Long short-term memory. Neural computation 1997;9(8):1735–1780.
89. Pratas D, Hosseini M, Pinho AJ. GeCo2: An optimized tool for lossless compression and analysis of DNA sequences. In: International Conference on Practical Applications of Computational Biology & Bioinformatics Springer; 2019. p. 137–145.

90. Pinho AJ, Garcia SP, Pratas D, Ferreira PJ. DNA sequences at a glance. PloS one 2013;8(11):e79922.
91. Pinho AJ, Pratas D, Ferreira PJ, Garcia SP. Symbolic to numerical conversion of DNA sequences using finite-context models. In: 2011 19th European Signal Processing Conference IEEE; 2011. p. 2024–2028.
92. Almeida JR, Pinho AJ, Oliveira JL, Fajarda O, Pratas D. GTO: a toolkit to unify pipelines in genomic and proteomic research. SoftwareX 2020;12:100535.
93. Romiguier J, Ranwez V, Douzery EJ, Galtier N. Contrasting GC-content dynamics across 33 mammalian genomes: relationship with life-history traits and chromosome sizes. Genome research 2010;20(8):1001–1009.
94. Duret L, Galtier N. Biased gene conversion and the evolution of mammalian genomic landscapes. Annual review of genomics and human genetics 2009;10:285–311.
95. Simmonds P, Ansari MA. Extensive C-> U transition biases in the genomes of a wide range of mammalian RNA viruses; potential associations with transcriptional mutations, damage-or host-mediated editing of viral RNA. PLoS pathogens 2021;17(6):e1009596.
96. Yakovchuk P, Protozanova E, Frank-Kamenetskii MD. Base-stacking and base-pairing contributions into thermal stability of the DNA double helix. Nucleic acids research 2006;34(2):564–574.
97. Chen H, Skylaris CK. Analysis of DNA interactions and GC content with energy decomposition in large-scale quantum mechanical calculations. Physical Chemistry Chemical Physics 2021;23(14):8891–8899.
98. Kans J. Entrez direct: E-utilities on the UNIX command line. National Center for Biotechnology Information (US); 2020.
99. McLachlan GJ. Discriminant analysis and statistical pattern recognition, vol. 544. John Wiley & Sons; 2004.
100. Rish I, et al. An empirical study of the naive Bayes classifier. In: IJCAI 2001 workshop on empirical methods in artificial intelligence, vol. 3; 2001. p. 41–46.
101. Guo G, Wang H, Bell D, Bi Y, Greer K. KNN Model-Based Approach in Classification. Springer Berlin Heidelberg 2003;p. 986–996.
102. Cristianini N, Shawe-Taylor J, et al. An introduction to support vector machines and other kernel-based learning methods. Cambridge university press; 2000.
103. Chen T, Guestrin C. XGBoost: A Scalable Tree Boosting System. In: Proceedings of the 22nd ACM SIGKDD International Conference on Knowledge Discovery and Data Mining KDD ’16, New York, NY, USA: ACM; 2016. p. 785–794.
104. Mahoney M, of Computer Sciences FTD, editor, Data compression programs. Overview over PAQ based compression software. http://mattmahoney. net/dc . . . ; 2009.
105. Prangishvili D, Rensen E, Mochizuki T, Krupovic M, et al. ICTV virus taxonomy profile: Tristromaviridae. Journal of General Virology 2019;100(2):135–136.
106. Krupovic M, Kuhn JH, Wang F, Baquero DP, Dolja VV, Egelman EH, et al. Adnaviria: a new realm for archaeal filamentous viruses with linear A-form double-stranded DNA genomes. Journal of Virology 2021;p. JVI–00673.
107. Krupovic M, Cvirkaite-Krupovic V, Iranzo J, Prangishvili D, Koonin EV. Viruses of archaea: structural, functional, environmental and evolutionary genomics. Virus research 2018;244:181–193.
108. Ayllón MA, Turina M, Xie J, Nerva L, Marzano SYL, Donaire L, et al. ICTV virus taxonomy profile: Botourmiaviridae. The Journal of general virology 2020;101(5):454.
109. Savin KW, Cocks BG, Wong F, Sawbridge T, Cogan N, Savage D, et al. A neurotropic herpesvirus infecting the gastropod, abalone, shares ancestry with oyster herpesvirus and a herpesvirus associated with the amphioxus genome. Virology journal 2010;7(1):1–9.
110. King AM, Lefkowitz E, Adams MJ, Carstens EB. Virus taxonomy: ninth report of the International Committee on Taxonomy of Viruses, vol. 9. Elsevier; 2011.
111. Pyöriä L, Jokinen M, Toppinen M, Salminen H, Vuorinen T, Hukkanen V, et al. HERQ-9 is a new multiplex PCR for differentiation and quantification of all nine human herpesviruses. Msphere 2020;5(3):e00265–20.
112. Baines JD, Pellett PE. Genetic comparison of human alphaherpesvirus genomes. Human herpesviruses: biology, therapy, and immunoprophylaxis 2007;.
113. Liu X, Kosugi S, Koide R, Kawamura Y, Ito J, Miura H, et al. Endogenization and excision of human herpesvirus 6 in human genomes. PLoS Genetics 2020;16(8):e1008915.
114. Rajaby R, Zhou Y, Meng Y, Zeng X, Li G, Wu P, et al. SurVirus: a repeat-aware virus integration caller. Nucleic acids research 2021;49(6):e33–e33.
115. Aimola G, Beythien G, Aswad A, Kaufer BB. Current understanding of human herpesvirus 6 (HHV-6) chromosomal integration. Antiviral research 2020;176:104720.
116. Morgenstern B. Sequence comparison without alignment: The SpaM approaches. In: Multiple Sequence Alignment Springer; 2021.p. 121–134.
117. Dencker T, Leimeister CA, Gerth M, Bleidorn C, Snir S, Morgenstern B. ‘Multi-SpaM’: a maximum-likelihood approach to phylogeny reconstruction using multiple spaced-word matches and quartet trees. NAR Genomics and Bioinformatics 2019 10;2(1). Lqz013.
118. Garcia BJ, Simha R, Garvin M, Furches A, Jones P, Gazolla JG, et al. A k-mer based approach for classifying viruses without taxonomy identifies viral associations in human autism and plant microbiomes. Computational and structural biotechnology journal 2021;19:5911–5919.
119. Zhang Q, Jun SR, Leuze M, Ussery D, Nookaew I. Viral phylogenomics using an alignment-free method: A three-step approach to determine optimal length of k-mer. Scientific reports 2017;7(1):1–13.
120. He L, Sun S, Zhang Q, Bao X, Li PK. Alignment-free sequence comparison for virus genomes based on location correlation coefficient. Infection, Genetics and Evolution 2021;96:105106.
121. Smyth GK. Statistical applications in genetics and molecular biology. Linear models and empirical Bayes methods for assessing differential expression in microarray experiments 2004;.
122. Lu J, Salzberg SL. Removing contaminants from databases of draft genomes. PLoS computational biology 2018;14(6):e1006277.
123. Sanjuán R, Domingo-Calap P. Mechanisms of viral mutation. Cellular and molecular life sciences 2016;73(23):4433–4448.
124. Mahy BW. The evolution and emergence of RNA viruses. Emerging infectious diseases 2010;16(5):899.
125. Simmonds P. Rampant C→ U hypermutation in the genomes of SARS-CoV-2 and other coronaviruses: causes and consequences for their short-and long-term evolutionary trajectories. Msphere 2020;5(3):e00408–20.
126. Silva JM, Pratas D, Caetano T, Matos S. Supporting data for "The complexity landscape of viral genomes". GigaScience Database 2022.

## Supplementary Material

Here, we depict the supplementary material of the article. The supplementary material is described in five main sections: Compression Model Benchmark, Viral Genome Analysis, Classification, Software and Hardware recommendations, and Reproducibility. The Compression Model Benchmark, Viral Genome Analysis and Classification sections have auxiliary material to their corresponding sections of the main article. On the other hand, the Software and Hardware recommendations section defines minimum requirements, and the Reproducibility section describes how to reproduce the results obtained in this article.

### Viruses Microbiology Additional Information.

Viruses can exist outside of their host in the form of independent particles named virions composed of the genetic material (DNA or RNA) enclosed by the capsid. This protein shell protects the viral genome, and at the same time, it is extracellular and promotes its entry into the host cells [1].

Most of the viruses possess capsids with helical (Figure S1 A) or icosahedral (Figure S1 B) arrangements [2, 3]. Different viruses, like bacteriophages, have developed other structures composed of elongated capsids attached to a cylindrical tailed sheet (Figure S1 C) [4]. Others have an outer lipid bilayer named viral envelope (Figure S1 D), which is constituted by a modified form of the host's cell membranes. Viroids have naked genomes without any protective layer. Like viruses, they use the host's machinery to replicate, but their genomes do not encode proteins [5]. Furthermore, some viruses are dependent on another virus species in the host cell to be transmitted to new cells. They were named 'satellites' and may represent evolutionary intermediates of viroids and viruses [6, 7]. Regarding mutability, the viral and viroid realm is the most mutable [8] of the realms.

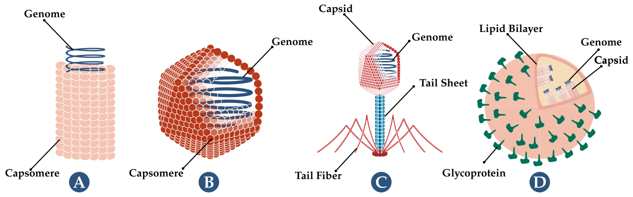


**Figure S1.** Illustrations of types of virus morphology. Virus (**A**) is a helical virus, where the capsoid has a helical shape that envelops the genomic material, virus (**B**) is icosahedral following cubic symmetry, (**C**) depicts a complex virus, namely a bacteriophage with a prolate capsid protecting the genomic material, and (**D**) is virus covered by a viral envelop.

Viral genomes can be of double-stranded DNA (dsDNA), single-stranded DNA (ssDNA), double-stranded RNA (dsRNA) or single-stranded RNA (ssRNA) nature, being linear or circular molecules [9]. The ssRNA viruses can be further classified as positive- or negative-ssRNA, depending on the sense of their RNA strand. These features determine the viral replication and mRNA synthesis pathways. For instance, (+)-ssRNA is directly translated into proteins by the host cell's ribosomes, acting as mRNA. On the other hand, (-)-ssRNA needs to be converted to a (+)-ssRNA by an RNA-dependent RNA polymerase (RdRp) before translation. RdRp also transcribes dsRNA to mRNA (using the negative strand as a template), and it is indispensable for the replication of RNA viral genomes. Finally, ssDNA and dsDNA usually use the host's DNA-dependent RNA polymerase to form mRNA. However, before this process, ssDNA is converted to a dsDNA by a DNA polymerase upon cell invasion [10], which is also the enzyme involved in the replication of DNA viruses. The RdRps have a high error rate due to their low proofreading activity and, therefore, replication of RNA viruses is much more prone to mutation than that of DNA viruses [11].

### Data compressors and Level selection benchmark

Herein, it is depicted the supplementary material to the Data compressors and Level selection benchmark.

Figure S2 shows the compression-time and compression-ratio of various Human Herpesviruses genome sequences between cmix and GeCo3 compression.

Table S1 describes the parameters used in the six custom build levels. The flag *"tm"* is the template of a target context model, the flag *"lr"* defines the learning rate, and the flag *"hs"* defines the number of hidden nodes for the neural network.

**Table S1.** Depiction of the parameters used in the six custom levels.

| Level | Values |
|---|---|
| 1 | -tm 1:1:0:0:0.7/0:0:0 -tm 12:20:1:1:0.97/1:1:0.97 |
| 2 | -tm 1:1:0:0:0.7/0:0:0 -tm 12:20:1:1:0.97/2:1:0.97 |
| 3 | -tm 1:1:0:0:0.7/0:0:0 -tm 12:50:1:1:0.97/0:0:0.97 |
| 4 | -tm 1:1:0:0:0.7/0:0:0 -tm 12:20:1:1:0.97/0:0:0.97 -lr 0.05 -hs 40 |
| 5 | -tm 1:1:0:0:0.7/0:0:0 -tm 12:20:1:1:0.97/0:0:0.97 -lr 0.15 -hs 40 |
| 6 | -tm 1:1:0:0:0.7/0:0:0 -tm 12:20:1:1:0.97/0:0:0.97 -lr 0.3 -hs 40 |

Table S2 describes the parameters used in the template of a target context model. The template has the flag *"tm"* and follows the

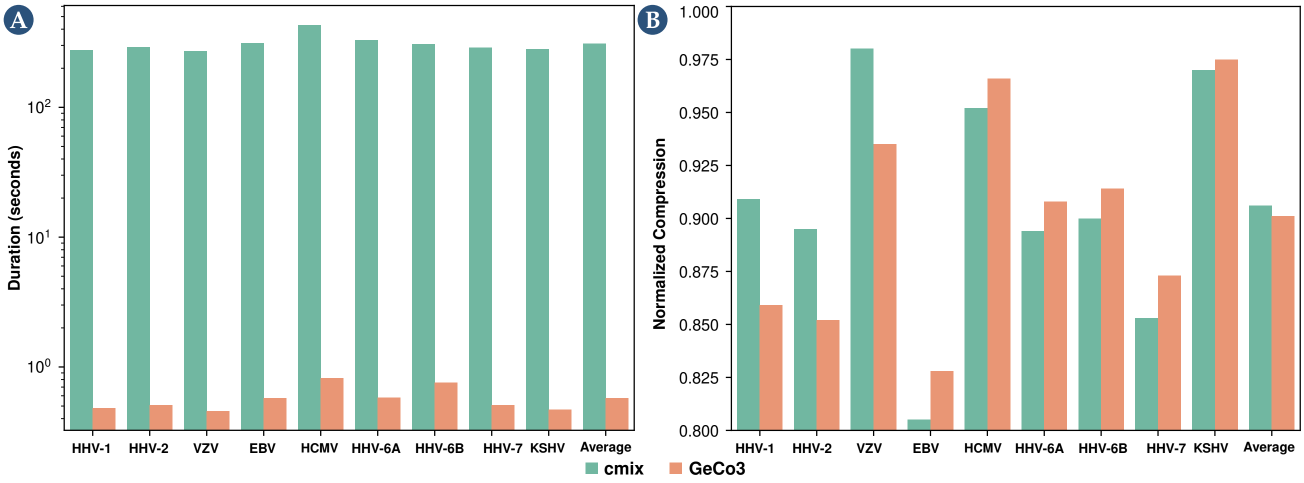


**Figure S2.** Comparison between cmix and GeCo3 when applied to various Human Herpesviruses regarding computational time and compression ratio obtained (NC).

model "[NB_C]:[NB_D]:[NB_I]:[NB_H]:[NB_G]/[NB_S]:[NB_E]:[NB_A]".

**Table S2.** Depiction of the parameters used in the template of a target context model.

| Parameter | Values | Description |
|---|---|---|
| **[NB_C]** | integer [1;20] | Order size of the regular context model. The higher the value of the regular context model, the more RAM it uses but, usually, are related to a better compression score. |
| **[NB_D]** | integer [1;5000] | Denominator to build alpha, which is a parameter estimator. Alpha is given by 1/[NB_D]. Higher values are usually used with higher [NB_C] and are related to sure bets. When [NB_D] is one, the probabilities assume a Laplacian distribution. |
| **[NB_I]** | integer {0,1,2} | Number to define if a sub-program that addresses the specific properties of DNA sequences (inverted repeats) is used or not. The number 2 turns ON this sub-program without the regular context model (only inverted repeats). The number 1 turns ON the sub-program using at the same time the regular context model. The number 0 does not contemplate its use (inverted repeats OFF). This sub-program increases the necessary time to compress, but it does not affect the RAM. |
| **[NB_H]** | integer [1;254] | Size of the cache-hash for deeper context models, namely for [NB_C] >14. When the [NB_C] <= 14 use, for example, 1 as a default. The RAM is highly dependent of this value (higher value stand for higher RAM). |
| **[NB_G]** | real [0;1) | Real number to define gamma. This value represents the decaying forgetting factor of the regular context model in the definition. |
| **[NB_S]** | integer [0;20] | The maximum number of editions allowed to use a substitutional tolerant model with the same memory model of the regular context model with an order size equal to [NB_C]. The value 0 stands for turning the tolerant context model off. When the model is on, it pauses when the number of editions is higher than [NB_C]. When it is turned on when a full match of size [NB_C] is seen again, this is a probabilistic-algorithmic model advantageous to handle the high substitutional nature of genomic sequences. When [NB_S] >0, the compressor used more processing time but used the same RAM and, usually, achieved a substantial higher compression ratio. The impact of this model is usually only noticed for [NB_C] >= 14. |
| **[NB_E]** | integer [1;5000] | Denominator to build alpha for substitutional tolerant context model. It is analogous to [NB_D]. However, it is only used in the probabilistic model for computing the statistics of the substitutional tolerant context model. |
| **[NB_A]** | real [0;1) | Real number to define gamma. This value represents the decaying forgetting factor of the substitutional tolerant context model in the definition. Its definition and use are analogous to [NB_G]. |

## Viral Genome Analysis

We present the supplementary material discussed in the Viral Genome Analysis of this main article. Table S3 depicts the genome types ordered by the highest normalized compression (NC), normalized compression capacity (*NCC*) and *difference*. *NCC* is computed by $NCC = 1 - NC_{IR_2} > 0$, and the difference as $difference = NC_{IR_0} - NC_{IR_1}$. Furthermore, the Table shows the genomes' average Sequence Length (SL) and GC-Content (GC).

Table S4 depicts the top Normalized Compression (NC) values by taxonomic group. Three main groups separate the Table. The first represents the highest 10 NC values using standard settings NC (best performing model); the second group shows the top 10 lowest NC values obtained using the $IR_2$ subprogram. Finally, the third group shows the top 10 highest values of the difference between NC using $IR_0$ and $IR_1$ subprograms.

Tables S5,S6,and S7 organize the top taxa (by taxonomic group) regarding their normalized compression (NC), normalized compression capacity (*NCC*) and *difference*. The tables also shows the genomes' average Sequence Length and GC-Content.

Finally, Figure S3 depicts the cladogram with average NC *difference* ($NC_{IR_0} - NC_{IR_1} > 0$) for each viral taxonomic group up to the viral genus. The colour red depicting the highest NC *difference*, and the blue the lowest.

**Table S3.** Depiction of the genome type by the highest normalized compression (NC), normalized compression capacity (*NCC*) and *difference*. *NCC* is computed by $NCC = 1 - NC_{IR_2} > 0$, and the difference as $difference = NC_{IR_0} - NC_{IR_1}$. Furthermore, the Table shows the genomes' average Sequence Length (SL) and GC-Content (GC).

| **Normalized Compression** | | | | **Inverted Repeats** | | | | **Difference** | | | |
|---|---|---|---|---|---|---|---|---|---|---|---|
| **Genome** | **NC** | **SL** | **GC** | **Genome** | **NCC** | **SL** | **GC** | **Genome** | **difference** | **SL** | **GC** |
| **ssDNA** | 1.065 | 3282 | 0.447 | **dsDNA** | 0.029 | 84721 | 0.485 | **ssDNA** | 0.006 | 4672 | 0.435 |
| **mixedDNA** | 1.050 | 3258 | 0.491 | **ssDNA** | 0.026 | 5981 | 0.389 | **dsDNA** | 0.006 | 80636 | 0.470 |
| **dsRNA** | 1.047 | 8377 | 0.456 | **ssRNA** | 0.015 | 13425 | 0.393 | **mixedDNA** | 0.002 | 3311 | 0.434 |
| **ssRNA** | 1.013 | 9564 | 0.437 | **dsRNA** | 0.015 | 19911 | 0.396 | **dsRNA** | 0.001 | 6186 | 0.431 |
| **dsDNA** | 0.977 | 70353 | 0.481 | | | | | **ssRNA** | 0.001 | 10197 | 0.433 |

**Table S4.** Depiction of the top NC values by taxonomic group. Three main groups separate the Table. The first represents the highest 10 NC values using standard settings NC (best performing model); the second group shows the top 10 lowest NC values obtained using the $IR_2$ subprogram. Finally, the third group shows the top 10 highest values of the difference between NC using $IR_0$ and $IR_1$ subprograms.

| *NC* | **Top** | **Realm** | **Kingdom** | **Phylum** | **Class** | **Order** | **Family** | **Genus** |
|---|---|---|---|---|---|---|---|---|
| *Best performing model* | **1** | Ribozyviria | Shotokuvirae | Lenarviricota | Miaviricetes | Ourlivirales | Botourmiaviridae | Clostunsatellite |
| | **2** | Monodnaviria | Sangervirae | Cressdnaviricota | Arfiviricetes | Cirlivirales | Alphasatellitidae | Milvetsatellite |
| | **3** | Riboviria | Orthornavirae | Duplornaviricota | Chunqiuviricetes | Cremevirales | Tolecusatellitidae | Aumaivirus |
| | **4** | Duplodnaviria | Pararnavirae | Phixviricota | Magsaviricetes | Muvirales | Circoviridae | Virtovirus |
| | **5** | Varidnaviria | Loebvirae | Kitrinoviricota | Amabiliviricetes | Nodamuvirales | Genomoviridae | Mivedwarsatellite |
| | **6** | Adnaviria | Trapavirae | Cossaviricota | Duplopiviricetes | Wolframvirales | Nodaviridae | Babusatellite |
| | **7** | - | Heunggongvirae | Pisuviricota | Allassoviricetes | Durnavirales | Kolmioviridae | Fabenesatellite |
| | **8** | - | Bamfordvirae | Negarnaviricota | Repensiviricetes | Levivirales | Smacoviridae | Ourmiavirus |
| | **9** | - | Helvetiavirae | Artverviricota | Yunchangviricetes | Geplafuvirales | Qinviridae | Albetovirus |
| | **10** | - | Zilligvirae | Hofneiviricota | Insthoviricetes | Goujianvirales | Narnaviridae | Geminialphasatellitinae |
| *Inverted Repeat Subprogram ($NC_{IR_2}$)* | **1** | Adnaviria | Loebvirae | Peploviricota | Pokkesviricetes | Imitervirales | Mimiviridae | Betaentomopoxvirus |
| | **2** | Varidnaviria | Zilligvirae | Nucleocytoviricota | Herviviricetes | Chitovirales | Rudiviridae | Oryzopoxvirus |
| | **3** | Duplodnaviria | Helvetiavirae | Hofneiviricota | Maveriviricetes | Herpesvirales | Poxviridae | Vespertilionpoxvirus |
| | **4** | Monodnaviria | Bamfordvirae | Taleaviricota | Mouviricetes | Priklausovirales | Malacoherpesviridae | Simplexvirus |
| | **5** | Riboviria | Heunggongvirae | Dividoviricota | Faserviricetes | Polivirales | Plectroviridae | Cafeteriavirus |
| | **6** | Ribozyviria | Trapavirae | Uroviricota | Tokiviricetes | Ligamenvirales | Mononiviridae | Mardivirus |
| | **7** | - | Shotokuvirae | Saleviricota | Laserviricetes | Tubulavirales | Herpesviridae | Cervidpoxvirus |
| | **8** | - | Pararnavirae | Preplasmiviricota | Megaviricetes | Halopanivirales | Lavidaviridae | Varicellovirus |
| | **9** | - | Orthornavirae | Negarnaviricota | Naldaviricetes | Pimascovirales | Bidnaviridae | Ostreavirus |
| | **10** | - | Sangervirae | Cossaviricota | Milneviricetes | Lefavirales | Polydnaviridae | Vespertiliovirus |
| *$NC_{IR_0} - NC_{IR_1}$* | **1** | Adnaviria | Zilligvirae | Peploviricota | Herviviricetes | Herpesvirales | Malacoherpesviridae | Mardivirus |
| | **2** | Varidnaviria | Trapavirae | Taleaviricota | Mouviricetes | Polivirales | Herpesviridae | Ostreavirus |
| | **3** | Duplodnaviria | Bamfordvirae | Nucleocytoviricota | Tokiviricetes | Chitovirales | Rudiviridae | Iltovirus |
| | **4** | Monodnaviria | Heunggongvirae | Saleviricota | Pokkesviricetes | Ligamenvirales | Bidnaviridae | Leporipoxvirus |
| | **5** | Ribozyviria | Shotokuvirae | Cossaviricota | Quintoviricetes | Piccovirales | Poxviridae | Simplexvirus |
| | **6** | Riboviria | Helvetiavirae | Dividoviricota | Huolimaviricetes | Haloruvirales | Polydnaviridae | Varicellovirus |
| | **7** | - | Loebvirae | Hofneiviricota | Megaviricetes | Cirlivirales | Ampullaviridae | Aurivirus |
| | **8** | - | Sangervirae | Cressdnaviricota | Laserviricetes | Pimascovirales | Nudiviridae | Oryzopoxvirus |
| | **9** | - | Orthornavirae | Preplasmiviricota | Arfiviricetes | Algavirales | Parvoviridae | Vespertilionpoxvirus |
| | **10** | - | Pararnavirae | Duplornaviricota | Faserviricetes | Kalamavirales | Ascoviridae | Entnonagintavirus |

**Table S5.** Depiction of the taxonomic groups with the highest NC values. The Table shows each group's average Normalized Compression, Sequence Length and GC-Content.

| Taxonomic Group | Taxonomic Name | Normalized Compression | Sequence Length | GC-Content |
|---|---|---|---|---|
| **Super-Realm** | Viruses | 1.007 | 36067 | 0.460 |
| **Realm** | Ribozyviria | 1.080 | 1682 | 0.588 |
| | Monodnaviria | 1.046 | 4380 | 0.450 |
| | Riboviria | 1.016 | 9332 | 0.438 |
| | Duplodnaviria | 0.972 | 78102 | 0.500 |
| | Varidnaviria | 0.957 | 109560 | 0.448 |
| | Adnaviria | 0.948 | 33068 | 0.353 |
| **Kingdom** | Shotokuvirae | 1.049 | 4200 | 0.447 |
| | Sangervirae | 1.026 | 5518 | 0.435 |
| | Orthornavirae | 1.018 | 9472 | 0.438 |
| | Pararnavirae | 0.995 | 7787 | 0.433 |
| | Loebvirae | 0.994 | 7332 | 0.483 |
| | Trapavirae | 0.993 | 10151 | 0.564 |
| | Heunggongvirae | 0.972 | 78102 | 0.500 |
| | Bamfordvirae | 0.957 | 112955 | 0.441 |
| | Helvetiavirae | 0.949 | 24833 | 0.665 |
| | Zilligvirae | 0.948 | 33068 | 0.353 |
| **Phylum** | Lenarviricota | 1.094 | 2654 | 0.476 |
| | Cressdnaviricota | 1.067 | 3134 | 0.453 |
| | Duplornaviricota | 1.045 | 9418 | 0.456 |
| | Phixviricota | 1.026 | 5518 | 0.435 |
| | Kitrinoviricota | 1.018 | 8548 | 0.474 |
| | Cossaviricota | 1.013 | 6260 | 0.436 |
| | Pisuviricota | 1.012 | 10580 | 0.442 |
| | Negarnaviricota | 1.012 | 9620 | 0.397 |
| | Artverviricota | 0.995 | 7787 | 0.433 |
| | Hofneiviricota | 0.994 | 7332 | 0.483 |
| **Class** | Miaviricetes | 1.151 | 1792 | 0.514 |
| | Arfiviricetes | 1.085 | 2557 | 0.464 |
| | Chunqiuviricetes | 1.075 | 3870 | 0.503 |
| | Magsaviricetes | 1.073 | 3730 | 0.513 |
| | Amabiliviricetes | 1.072 | 2703 | 0.586 |
| | Duplopiviricetes | 1.066 | 3298 | 0.467 |
| | Allassoviricetes | 1.063 | 3753 | 0.493 |
| | Repensiviricetes | 1.063 | 3281 | 0.451 |
| | Yunchangviricetes | 1.061 | 3987 | 0.358 |
| | Insthoviricetes | 1.054 | 5784 | 0.425 |
| **Order** | Ourlivirales | 1.151 | 1792 | 0.514 |
| | Cirlivirales | 1.103 | 1864 | 0.471 |
| | Cremevirales | 1.078 | 2572 | 0.478 |
| | Muvirales | 1.075 | 3870 | 0.503 |
| | Nodamuvirales | 1.073 | 3730 | 0.513 |
| | Wolframvirales | 1.072 | 2703 | 0.586 |
| | Durnavirales | 1.066 | 3298 | 0.467 |
| | Levivirales | 1.063 | 3753 | 0.493 |
| | Geplafuvirales | 1.063 | 3281 | 0.451 |
| | Goujianvirales | 1.061 | 3987 | 0.358 |
| **Family** | Botourmiaviridae | 1.151 | 1792 | 0.514 |
| | Alphasatellitidae | 1.143 | 1296 | 0.418 |
| | Tolecusatellitidae | 1.116 | 1347 | 0.389 |
| | Circoviridae | 1.103 | 1864 | 0.471 |
| | Genomoviridae | 1.096 | 2201 | 0.517 |
| | Nodaviridae | 1.080 | 3368 | 0.514 |
| | Kolmioviridae | 1.080 | 1682 | 0.588 |
| | Smacoviridae | 1.078 | 2572 | 0.478 |
| | Qinviridae | 1.075 | 3870 | 0.503 |
| | Narnaviridae | 1.072 | 2703 | 0.586 |
| **Genus** | Clostunsatellite | 1.192 | 1008 | 0.423 |
| | Milvetsatellite | 1.186 | 1022 | 0.402 |
| | Aumaivirus | 1.185 | 1168 | 0.510 |
| | Virtovirus | 1.180 | 1150 | 0.442 |
| | Mivedwarsatellite | 1.179 | 1014 | 0.402 |
| | Babusatellite | 1.178 | 1104 | 0.437 |
| | Fabenesatellite | 1.176 | 1007 | 0.385 |
| | Ourmiavirus | 1.167 | 1605 | 0.519 |
| | Albetovirus | 1.167 | 1221 | 0.426 |
| | Geminialphasatellitinae | 1.131 | 1370 | 0.418 |

**Table S6.** Depiction of the taxonomic groups with the highest normalized compression capacity (*NCC*) using only the inverted repeats subprogram $IR_2$. The top results were obtained by $NCC = 1 - NC_{IR_2} > 0$. Besides the normalized compression capacity, the Table shows each group's average Sequence Length and GC-Content.

| Group | Taxonomic Group | $NCC = 1 - NC_{IR_2} > 0$ | Sequence Legth | GC-Content |
|---|---|---|---|---|
| **Super-Realm** | Viruses | 0.026 | 66796 | 0.462 |
| **Realm** | Adnaviria | 0.052 | 33068 | 0.353 |
| | Varidnaviria | 0.038 | 110591 | 0.447 |
| | Duplodnaviria | 0.028 | 82677 | 0.499 |
| | Monodnaviria | 0.022 | 6958 | 0.399 |
| | Riboviria | 0.015 | 13682 | 0.391 |
| **Kingdom** | Loebvirae | 0.053 | 7371 | 0.385 |
| | Zilligvirae | 0.052 | 33068 | 0.353 |
| | Helvetiavirae | 0.050 | 24833 | 0.665 |
| | Bamfordvirae | 0.038 | 114079 | 0.440 |
| | Heunggongvirae | 0.028 | 82677 | 0.499 |
| | Trapavirae | 0.021 | 12225 | 0.577 |
| | Shotokuvirae | 0.016 | 6184 | 0.378 |
| | Pararnavirae | 0.016 | 9610 | 0.378 |
| | Orthornavirae | 0.015 | 14012 | 0.393 |
| | Sangervirae | 0.005 | 4421 | 0.321 |
| **Phylum** | Peploviricota | 0.068 | 168832 | 0.534 |
| | Nucleocytoviricota | 0.063 | 210417 | 0.389 |
| | Hofneiviricota | 0.053 | 7371 | 0.385 |
| | Taleaviricota | 0.052 | 33068 | 0.353 |
| | Dividoviricota | 0.050 | 24833 | 0.665 |
| | Uroviricota | 0.026 | 79042 | 0.497 |
| | Saleviricota | 0.021 | 12225 | 0.577 |
| | Preplasmiviricota | 0.017 | 32147 | 0.483 |
| | Negarnaviricota | 0.016 | 12180 | 0.376 |
| | Cossaviricota | 0.016 | 6128 | 0.378 |
| **Class** | Pokkesviricetes | 0.072 | 190762 | 0.365 |
| | Herviviricetes | 0.068 | 168832 | 0.534 |
| | Maveriviricetes | 0.066 | 18227 | 0.290 |
| | Mouviricetes | 0.066 | 8377 | 0.299 |
| | Faserviricetes | 0.053 | 7371 | 0.385 |
| | Tokiviricetes | 0.052 | 33068 | 0.353 |
| | Laserviricetes | 0.050 | 24833 | 0.665 |
| | Megaviricetes | 0.046 | 248459 | 0.436 |
| | Naldaviricetes | 0.040 | 132022 | 0.410 |
| | Milneviricetes | 0.029 | 11079 | 0.349 |
| **Order** | Imitervirales | 0.109 | 899501 | 0.256 |
| | Chitovirales | 0.091 | 193551 | 0.356 |
| | Herpesvirales | 0.068 | 168832 | 0.534 |
| | Priklausovirales | 0.066 | 18227 | 0.290 |
| | Polivirales | 0.066 | 8377 | 0.299 |
| | Ligamenvirales | 0.055 | 34464 | 0.343 |
| | Tubulavirales | 0.053 | 7371 | 0.385 |
| | Halopanivirales | 0.050 | 24833 | 0.665 |
| | Pimascovirales | 0.043 | 162587 | 0.456 |
| | Lefavirales | 0.040 | 132022 | 0.410 |
| **Family** | Mimiviridae | 0.109 | 899501 | 0.256 |
| | Rudiviridae | 0.103 | 30804 | 0.299 |
| | Poxviridae | 0.091 | 193551 | 0.356 |
| | Malacoherpesviridae | 0.091 | 209479 | 0.427 |
| | Plectroviridae | 0.080 | 7045 | 0.248 |
| | Mononiviridae | 0.077 | 41178 | 0.275 |
| | Herpesviridae | 0.074 | 158421 | 0.539 |
| | Lavidaviridae | 0.066 | 18227 | 0.290 |
| | Bidnaviridae | 0.066 | 8377 | 0.299 |
| | Polydnaviridae | 0.055 | 306235 | 0.377 |
| **Genus** | Betaentomopoxvirus | 0.174 | 247441 | 0.195 |
| | Oryzopoxvirus | 0.164 | 185139 | 0.236 |
| | Vespertilionpoxvirus | 0.156 | 176688 | 0.236 |
| | Simplexvirus | 0.144 | 148626 | 0.694 |
| | Cafeteriavirus | 0.127 | 617453 | 0.233 |
| | Mardivirus | 0.121 | 177993 | 0.509 |
| | Cervidpoxvirus | 0.115 | 166259 | 0.262 |
| | Varicellovirus | 0.107 | 139331 | 0.560 |
| | Ostreavirus | 0.107 | 207439 | 0.387 |
| | Vespertiliovirus | 0.103 | 7970 | 0.228 |

**Table S7.** Depiction of the taxonomic groups with the highest difference of values between $NC_{IR_0} - NC_{IR_1}$. The Table shows each group's average *difference* = $NC_{IR_0} - NC_{IR_1}$, Sequence Length and GC-Content.

| Taxonomic Group | Taxonomic Name | $NC_{IR_0} - NC_{IR_1} > 0$ | Sequece Length | GC-Content |
|---|---|---|---|---|
| **Super-Realm** | Viruses | 0.004 | 44293 | 0.451 |
| **Realm** | Adnaviria | 0.019 | 35299 | 0.322 |
| | Varidnaviria | 0.007 | 111364 | 0.443 |
| | Duplodnaviria | 0.007 | 78316 | 0.512 |
| | Monodnaviria | 0.005 | 5359 | 0.436 |
| | Ribozyviria | 0.002 | 1682 | 0.588 |
| | Riboviria | 0.001 | 9847 | 0.431 |
| **Kingdom** | Zilligvirae | 0.019 | 35299 | 0.322 |
| | Trapavirae | 0.009 | 16113 | 0.503 |
| | Bamfordvirae | 0.007 | 114249 | 0.437 |
| | Heunggongvirae | 0.007 | 78316 | 0.512 |
| | Shotokuvirae | 0.005 | 5124 | 0.434 |
| | Helvetiavirae | 0.004 | 27439 | 0.664 |
| | Loebvirae | 0.002 | 8519 | 0.453 |
| | Sangervirae | 0.001 | 4552 | 0.426 |
| | Orthornavirae | 0.001 | 10049 | 0.430 |
| | Pararnavirae | 0.001 | 8050 | 0.435 |
| **Phylum** | Peploviricota | 0.050 | 159507 | 0.557 |
| | Taleaviricota | 0.019 | 35299 | 0.322 |
| | Nucleocytoviricota | 0.013 | 210797 | 0.381 |
| | Saleviricota | 0.009 | 16113 | 0.503 |
| | Cossaviricota | 0.007 | 5450 | 0.433 |
| | Dividoviricota | 0.004 | 27439 | 0.664 |
| | Hofneiviricota | 0.002 | 8519 | 0.453 |
| | Cressdnaviricota | 0.002 | 4539 | 0.438 |
| | Preplasmiviricota | 0.002 | 32788 | 0.483 |
| | Duplornaviricota | 0.001 | 8140 | 0.389 |
| **Class** | Herviviricetes | 0.050 | 159507 | 0.557 |
| | Mouviricetes | 0.029 | 8377 | 0.299 |
| | Tokiviricetes | 0.019 | 35299 | 0.322 |
| | Pokkesviricetes | 0.017 | 193309 | 0.354 |
| | Quintoviricetes | 0.011 | 5164 | 0.446 |
| | Huolimaviricetes | 0.009 | 16113 | 0.503 |
| | Megaviricetes | 0.005 | 247791 | 0.441 |
| | Laserviricetes | 0.004 | 27439 | 0.664 |
| | Arfiviricetes | 0.004 | 5459 | 0.432 |
| | Faserviricetes | 0.002 | 8519 | 0.453 |
| **Order** | Herpesvirales | 0.050 | 159507 | 0.557 |
| | Polivirales | 0.029 | 8377 | 0.299 |
| | Chitovirales | 0.022 | 196072 | 0.341 |
| | Ligamenvirales | 0.019 | 35299 | 0.322 |
| | Piccovirales | 0.011 | 5164 | 0.446 |
| | Haloruvirales | 0.009 | 16113 | 0.503 |
| | Cirlivirales | 0.008 | 2114 | 0.476 |
| | Pimascovirales | 0.005 | 169619 | 0.458 |
| | Algavirales | 0.005 | 339710 | 0.413 |
| | Kalamavirales | 0.004 | 15181 | 0.459 |
| **Family** | Malacoherpesviridae | 0.062 | 209479 | 0.427 |
| | Herpesviridae | 0.050 | 155406 | 0.564 |
| | Rudiviridae | 0.035 | 30804 | 0.299 |
| | Bidnaviridae | 0.029 | 8377 | 0.299 |
| | Poxviridae | 0.022 | 196072 | 0.341 |
| | Polydnaviridae | 0.019 | 306235 | 0.377 |
| | Ampullaviridae | 0.012 | 23814 | 0.346 |
| | Nudiviridae | 0.012 | 127615 | 0.416 |
| | Parvoviridae | 0.011 | 5164 | 0.446 |
| | Ascoviridae | 0.010 | 172411 | 0.453 |
| **Genus** | Mardivirus | 0.103 | 177993 | 0.509 |
| | Ostreavirus | 0.072 | 207439 | 0.387 |
| | Iltovirus | 0.070 | 155856 | 0.546 |
| | Leporipoxvirus | 0.066 | 160815 | 0.415 |
| | Simplexvirus | 0.061 | 148626 | 0.694 |
| | Varicellovirus | 0.061 | 139331 | 0.560 |
| | Aurivirus | 0.052 | 211518 | 0.468 |
| | Oryzopoxvirus | 0.050 | 185139 | 0.236 |
| | Vespertilionpoxvirus | 0.046 | 176688 | 0.236 |
| | Entnonagintavirus | 0.036 | 29564 | 0.558 |

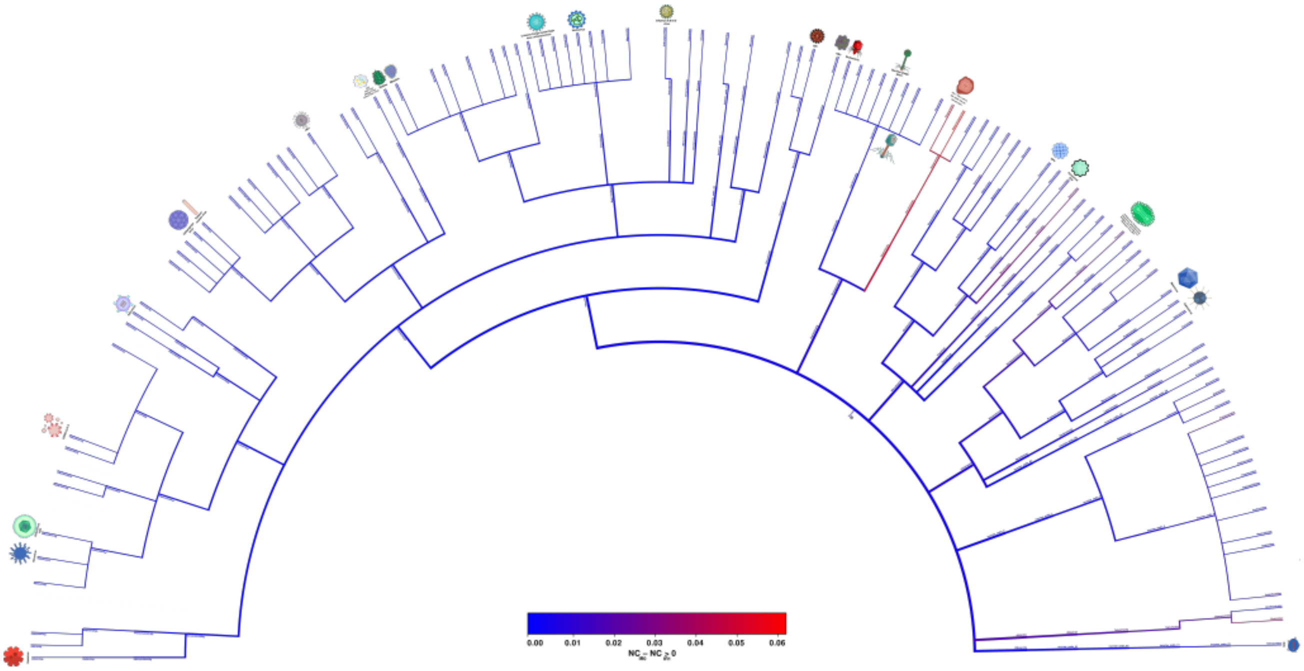


**Figure S3.** Cladogram showing average *difference* ($NC_{IR_0} - NC_{IR_1} > 0$).The colour red depicts the branches where on average, the genome possesses more inverted repetitions than internal repetitions (higher difference), whereas the blue colour represents the branches with fewer inverted repetitions than internal repetitions (smaller difference).

## Classification

Herein, we show the supplementary classification tables that are discussed in the classification subsection of this article.

Figure S4 represents the number of samples (genome sequences) per viral genus.

Table S8 and Table S9 show the values obtained using different classifiers for accuracy and F1-score, respectively. In both cases, the XGBoost classifier had the best performance.

Table S10 displays the XGBoost classifier F1-score results when using different sets of features. With the notable exception of the type of genome classification, the best results were obtained using all features.

**Table S8.** Accuracy (ACC) results obtained for viral taxonomic classification tasks regarding genome type, realm, kingdom, phylum, class, order, family, and genus. The classifiers used were Linear Discriminant Analysis (LDA), Gaussian Naive Bayes (GNB), K-Nearest Neighbors (KNN), Support Vector Machine (SVM), and XGBoost classifier (XGB).

| Classification | N. Classes | N. Samples | $ACC_{LDA}$ | $ACC_{GNB}$ | $ACC_{SVM}$ | $ACC_{KNN}$ | $ACC_{XGB}$ |
|---|---|---|---|---|---|---|---|
| **Genome** | 5 | 6089 | 67.32 | 74.14 | 72.41 | 84.4 | **87.25** |
| **Realm** | 5 | 5799 | 75.95 | 80.95 | 81.38 | 88.71 | **92.57** |
| **Kingdom** | 10 | 5788 | 73.49 | 78.76 | 78.41 | 85.49 | **90.96** |
| **Phylum** | 17 | 5778 | 61.59 | 56.75 | 55.88 | 71.28 | **83.41** |
| **Class** | 34 | 5845 | 51.15 | 52.95 | 47.56 | 63.47 | **80.23** |
| **Order** | 48 | 5838 | 48.89 | 55.65 | 48.89 | 60.62 | **79.62** |
| **Family** | 102 | 5990 | 36.64 | 43.24 | 27.05 | 42.99 | **74.46** |
| **Genus** | 360 | 4673 | 44.6 | 36.79 | 18.82 | 17.65 | **68.71** |

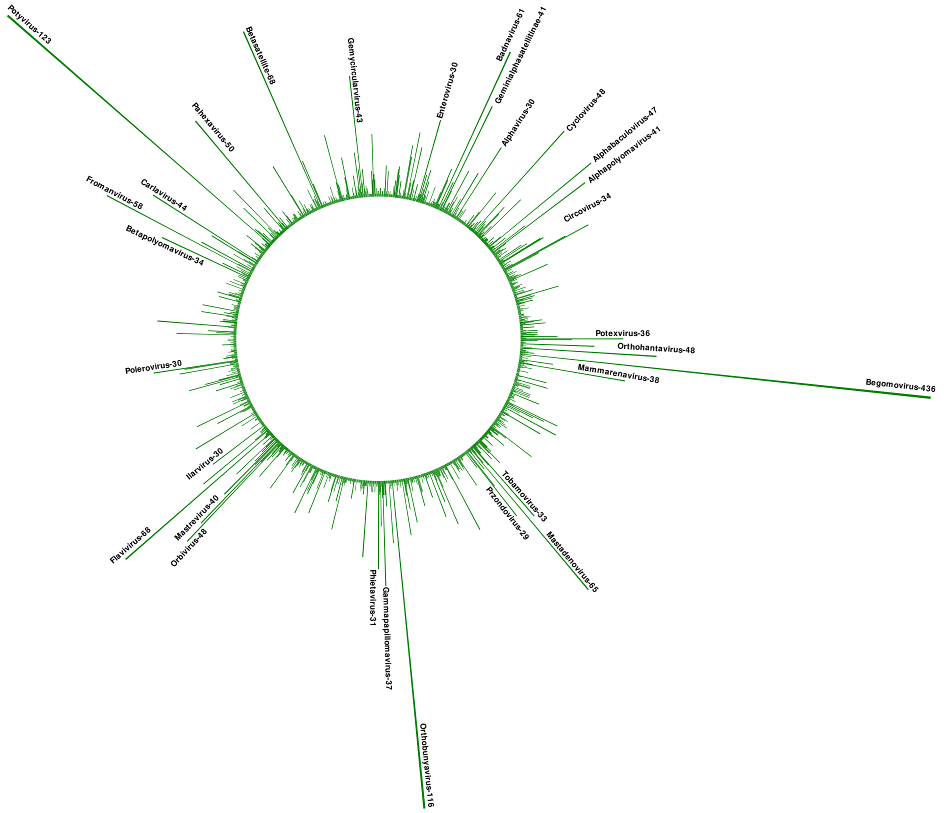


**Figure S4.** Frequency of genome sequences per viral genus.

**Table S9.** F1-score (F1) results obtained for viral taxonomic classification tasks regarding genome type, realm, kingdom, phylum, class, order, family, and genus. The classifiers used were Linear Discriminant Analysis (LDA), Gaussian Naive Bayes (GNB), K-Nearest Neighbors (KNN), Support Vector Machine (SVM), and XGBoost classifier (XGB).

| Classification | N. Classes | N. Samples | $F1_{LDA}$ | $F1_{GNB}$ | $F1_{SVM}$ | $F1_{KNN}$ | $F1_{XGB}$ |
|---|---|---|---|---|---|---|---|
| **Genome** | 5 | 6089 | 0.6549 | 0.736 | 0.6989 | 0.836 | **0.8662** |
| **Realm** | 5 | 5799 | 0.7496 | 0.8001 | 0.7949 | 0.8817 | **0.9234** |
| **Kingdom** | 10 | 5788 | 0.7238 | 0.7640 | 0.7512 | 0.8410 | **0.9039** |
| **Phylum** | 17 | 5778 | 0.5824 | 0.5226 | 0.4435 | 0.6891 | **0.8299** |
| **Class** | 34 | 5845 | 0.4780 | 0.4562 | 0.3803 | 0.5896 | **0.7963** |
| **Order** | 48 | 5838 | 0.4435 | 0.4798 | 0.3832 | 0.5462 | **0.7884** |
| **Family** | 102 | 5990 | 0.3042 | 0.3517 | 0.1681 | 0.3429 | **0.7323** |
| **Genus** | 360 | 4673 | 0.3600 | 0.2956 | 0.0682 | 0.0621 | **0.6561** |

**Table S10.** F1-score (F1) obtained for the viral taxonomic classification task regarding genome type, realm, kingdom, phylum, class, order, family, and genus. The features used were the genome's sequence length (SL), the GC-content (GC) and the Normalized Compression (NC) values for the best model, the same model with IR configuration to 0, 1 and 2.

| Classification | N. Classes | N. Samples | $F1_{NC}$ | $F1_{NC+GC}$ | $F1_{NC+SL+GC}$ | $F1_{All\ without\ SQ}$ | $F1_{AllFeatures}$ |
|---|---|---|---|---|---|---|---|
| **Genome** | 5 | 6089 | 0.7490 | 0.7988 | 0.8649 | 0.8051 | **0.8662** |
| **Realm** | 5 | 5799 | 0.7726 | 0.8401 | 0.9200 | 0.8569 | **0.9234** |
| **Kingdom** | 10 | 5788 | 0.7518 | 0.8131 | 0.9026 | 0.8295 | **0.9039** |
| **Phylum** | 17 | 5778 | 0.6234 | 0.6926 | 0.8194 | 0.7188 | **0.8299** |
| **Class** | 34 | 5845 | 0.5742 | 0.6404 | 0.7844 | 0.6705 | **0.7963** |
| **Order** | 48 | 5838 | 0.5568 | 0.6292 | 0.7736 | 0.6598 | **0.7884** |
| **Family** | 102 | 5990 | 0.4112 | 0.5187 | 0.7118 | 0.5636 | **0.7323** |
| **Genus** | 360 | 4673 | 0.3248 | 0.4661 | 0.6417 | 0.5089 | **0.6561** |

## Software and Hardware recommendations

The experiences of the manuscript can be replicated using a laptop, desktop, or server computer running Arch linux or Linux Ubuntu (for example, 18.04 LTS or higher) with GCC (`https://gcc.gnu.org`), git and git LFS, Conda (`https://docs.conda.io`) and python version 3.6. The hardware must contain at least 8 GB of RAM and a 100 GB disk.

## Reproducibility

***Creating Project and intalling tools***

The descriptions of reproducion is depicted bellow, for more detail see https://github.com/jorgeMFS/canvas. Install Git LFS:

```
mkdir -p gitLFS
cd gitLFS/
wget https://github.com/git-lfs/git-lfs/releases/download/v2.9.0/git-lfs-linux-amd64-v2.9.0.tar.gz
tar -xf git-lfs-linux-amd64-v2.9.0.tar.gz
chmod 755 install.sh
sudo ./install.sh
```

Get CANVAS project, create the docker and run it:

```
git clone https://github.com/jorgeMFS/canvas.git
cd canvas
docker-compose build
docker-compose up -d && docker exec -it canvas bash && docker-compose down
```

Inside the docker, give run permissions to the files and install tools using :

```
chmod +x *.sh
bash Make.sh;
```

***Replication of the Results***

The code was created in order to allow independent replication and reproduction of each step, this was done due to the extensive processing time required to filter and rearrange viral DB and extract the features and taxonomic information of each viral sequence. If you wish to rebuild database and feature reports extracted, see the Database reconstruction subsection.

To obtain the Human Herpesvirus, plot run:

```
cd python || exit;
python compare_cmix_hhv.py
```

To obtain the Compression Benchmark plots, run:

```
cd python || exit;
python select_best_nc_model.py;
```

To perform the synthetic sequence test, run:

```
cd scripts || exit;
bash Stx_seq_test.sh;
```

To perform classification, run the following code:

```
cd python || exit;
python prepare_classification.py; #recreate classification dataset
python classifier.py; #perform classifications
```

To perform the complete IR analysis and create:

- boxplots;
- 2d scatter plots;
- 3d scatter plots;
- top taxonomic group lists;
- Occurrence of each Genus.

Execute this code:

```
cd python || exit;
python ir_analysis.py; # Performs complete IR analysis
```

To perform the Human Herpesvirus analysis and obtain the plots, run:

```
cd scripts || exit;
bash Herpesvirales.sh;
```

***Database reconstruction***

To run the pipeline and obtain all the Reports in the folder reports, use the following commands. Note that if you wish to recreate the features reports, you must perform the database reconstruction task first.

If you wish to reconstruct the viral database, run the following script:

```
cd scripts || exit;
bash Build_DB.sh;
```

To create the features for analysis and classification (very time consuming, can take several days), run:

```
cd scripts || exit;
bash Process_features.sh;
```

To recreate the compression reports used for benchmark (very time consuming, can take several hours), run:

```
cd scripts || exit;
bash Compress.sh;
```

***Cladograms***

The Cladograms require GUI application. As such, the reproduction of the cladograms has to be performed outside of the docker on the Ubuntu system on the /canvas folder:

```
chmod +x *.sh
bash so_dependencies.sh #install Ubuntu system dependencies required for the script to run and Anaconda
conda create -n canvas python=3.6
conda activate canvas
bash Make.sh #install python libs
bash Install_programs.sh #install tools using conda
```

Afterwards, to obtain the Cladogram plots, run:

```
cd python || exit;
python phylo_tree.py;
```

## References


1. Strauss JH, Strauss EG. CHAPTER 1 - Overview of Viruses and Virus Infection. In: Strauss JH, Strauss EG, editors. Viruses and Human Disease (Second Edition), second edition ed. London: Academic Press; 2008.p. 1–33.
2. Lidmar J, Mirny L, Nelson DR. Virus shapes and buckling transitions in spherical shells. Physical Review E 2003;68(5):051910.
3. Vernizzi G, de la Cruz MO. Faceting ionic shells into icosahedra via electrostatics. Proceedings of the National Academy of Sciences 2007;104(47):18382–18386.
4. Luque A, Reguera D. The structure of elongated viral capsids. Biophysical journal 2010;98(12):2993–3003.
5. Tsagris EM, Martínez de Alba ÁE, Gozmanova M, Kalantidis K. Viroids. Cellular microbiology 2008;10(11):2168–2179.
6. Krupovic M, Cvirkaite-Krupovic V. Virophages or satellite viruses? Nature Reviews Microbiology 2011;9(11):762–763.
7. Dimmock NJ, Easton AJ, Leppard KN. Introduction to modern virology. John Wiley & Sons; 2016.
8. Gago S, Elena SF, Flores R, Sanjuán R. Extremely high mutation rate of a hammerhead viroid. Science 2009;323(5919):1308–1308.
9. Simón D, Cristina J, Musto H. Nucleotide composition and codon usage across viruses and their respective hosts. Frontiers in Microbiology 2021;12.
10. Baltimore D. Expression of animal virus genomes. Bacteriological reviews 1971;35(3):235–241.
11. Peck KM, Lauring AS. Complexities of viral mutation rates. Journal of virology 2018;92(14):e01031–17.

Click here to access/download;Manuscript;main.pdf

Your PDF file "main.pdf" cannot be opened and processed. Please see the common list of problems, and suggested resolutions below.

Reason:

Other Common Problems When Creating a PDF from a PDF file
-----------------------------------------------------------------

You will need to convert your PDF file to another format or fix the current PDF file, then re-submit it.

Click here to access/download;Figure;HHV.pdf

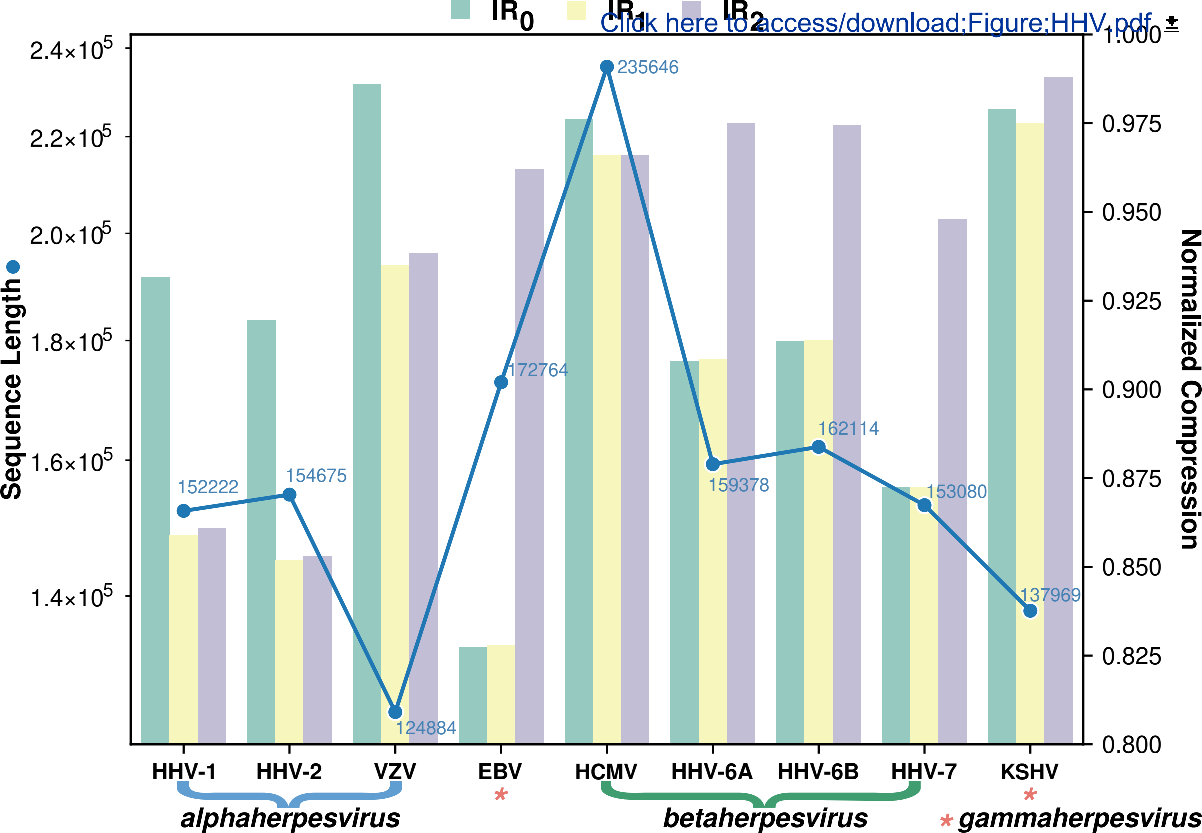

Click here to access/download
Supplementary Material
main.aux
