# Supplementary material for: The complexity landscape of viral genomes
Source: Gigascience. 2022 Aug 11;11:giac079. doi: 10.1093/gigascience/giac079 (PMC9366995; doi:10.1093/gigascience/giac079)

GigaScience

The complexity landscape of viral genomes

--Manuscript Draft--

**Manuscript Number:**	GIGA-D-22-00044
**Full Title:**	The complexity landscape of viral genomes
**Article Type:**	Research
**Funding Information:**	FCT – Fundação para a Ciência e a Tecnologia (SFRH/BD/141851/2018) — Mr. Jorge Miguel Ferreira da Silva<br>FCT – Fundação para a Ciência e a Tecnologia (UIDB/00127/2020) — Not applicable<br>FCT – Fundação para a Ciência e a Tecnologia (CEECINST/00026/2018) — Dr. Tânia Caetano<br>FCT/MCTES (UIDP/50017/2020+UIDB/50017/2020) — Dr. Tânia Caetano

Funding source	Recipient
FCT – Fundação para a Ciência e a Tecnologia (SFRH/BD/141851/2018)	Mr. Jorge Miguel Ferreira da Silva
FCT – Fundação para a Ciência e a Tecnologia (UIDB/00127/2020)	Not applicable
FCT – Fundação para a Ciência e a Tecnologia (CEECINST/00026/2018)	Dr. Tânia Caetano
FCT/MCTES (UIDP/50017/2020+UIDB/50017/2020)	Dr. Tânia Caetano

Abstract:

Background:

Viruses are amongst the shortest yet highly abundant species that harbor minimal instructions to infect cells, adapt, multiply, and exist. With the current substantial availability of viral genome sequences, the scientific repertory lacks a complexity landscape that automatically enlights viral genomes' organization, relation, and fundamental characteristics.

Results:

This work provides a comprehensive landscape of the viral genome's complexity (or quantity of information), identifying the most redundant and complex groups regarding their genome sequence while providing their distribution and characteristics at a large and local scale. For this purpose, we measure the sequence complexity of each available viral genome using data compression, demonstrating that adequate data compressors can efficiently quantify the complexity of viral genome sequences, including sub-sequences better represented by algorithmic sources (e.g., inverted repeats). Using a state-of-the-art genomic compressor on an extensive viral genomes database, we show that dsDNA viruses are on average the most redundant viruses while ssDNA viruses are the lowest. Contrarily, dsRNA viruses show a lower redundancy relative to ssRNA. We extend the ability of data compressors to quantify local complexity (or information content) in viral genomes using complexity profiles, unprecedently providing a direct complexity analysis to human Herpesviruses. We also conceive a features-based classification methodology that can accurately distinguish viral genomes at different taxonomic levels without using direct comparisons between sequences. This methodology works by combining data compression with simple measures such as GC-content percentage and sequence length followed by machine learning classifiers.

Conclusions:

This manuscript's presents methodologies and findings that are highly relevant for understanding the patterns of similarity and singularity between viral groups, opening new frontiers for studying viral genomes' organization while depicting the complexity trends and classification components of these genomes at different taxonomic levels. The whole study is supported by an extensive website (https://asilab.github.io/canvas/) for comprehending the viral genome characterization using dynamic and interactive approaches.

Corresponding Author: Jorge Miguel Ferreira da Silva
Universidade de Aveiro Instituto de Engenharia Eletrónica e Informática de Aveiro
Esmoriz, Seleccione um PORTUGAL

Corresponding Author Secondary

Powered by Editorial Manager® and ProduXion Manager® from Aries Systems Corporation

**Information:**	
**Corresponding Author's Institution:**	Universidade de Aveiro Instituto de Engenharia Eletrónica e Informática de Aveiro
**Corresponding Author's Secondary Institution:**	
**First Author:**	Jorge Miguel Ferreira da Silva
**First Author Secondary Information:**	
**Order of Authors:**	Jorge Miguel Ferreira da Silva
	Diogo Pratas, Ph.D.
	Tânia Caetano, Ph.D.
	Sérgio Matos, Professor
**Order of Authors Secondary Information:**	
**Additional Information:**	
**Question**	**Response**
Are you submitting this manuscript to a special series or article collection?	No
**Experimental design and statistics**<br><br>Full details of the experimental design and statistical methods used should be given in the Methods section, as detailed in our Minimum Standards Reporting Checklist. Information essential to interpreting the data presented should be made available in the figure legends.<br><br>Have you included all the information requested in your manuscript?	Yes
**Resources**<br><br>A description of all resources used, including antibodies, cell lines, animals and software tools, with enough information to allow them to be uniquely identified, should be included in the Methods section. Authors are strongly encouraged to cite Research Resource Identifiers (RRIDs) for antibodies, model organisms and tools, where possible.<br><br>Have you included the information requested as detailed in our Minimum Standards Reporting Checklist?	Yes

**Availability of data and materials**<br><br>All datasets and code on which the conclusions of the paper rely must be either included in your submission or deposited in publicly available repositories (where available and ethically appropriate), referencing such data using a unique identifier in the references and in the “Availability of Data and Materials” section of your manuscript.<br><br>Have you have met the above requirement as detailed in our Minimum Standards Reporting Checklist?	Yes

Manuscript Click here to access/download;Manuscript;manuscript.pdf

OXFORD (GIGA)^n SCIENCE

GigaScience, 2022, 1–9

doi: xx.xxxx/xxxx
Manuscript in Preparation
Paper

PAPER

The complexity landscape of viral genomes

Jorge Miguel Silva[1,*], Diogo Pratas[1,2,3], Tânia Caetano[4] and Sérgio Matos[1,2]

[1]Institute of Electronics and Informatics Engineering of Aveiro, University of Aveiro, Portugal and [2]Department of Electronics, Telecommunications and Informatics, University of Aveiro, Portugal and [3]Department of Virology, University of Helsinki, Finland and [4]CESAM and Department of Biology, University of Aveiro, Aveiro, Portugal

[*]Correspondence address. Jorge Miguel Silva E-mail: jorge.miguel.ferreira.silva@ua.pt https://orcid.org/0000-0002-6331-6091

Abstract

Background: Viruses are amongst the shortest yet highly abundant species that harbor minimal instructions to infect cells, adapt, multiply, and exist. With the current substantial availability of viral genome sequences, the scientific repertory lacks a complexity landscape that automatically enlights viral genomes' organization, relation, and fundamental characteristics.
Results: This work provides a comprehensive landscape of the viral genome's complexity (or quantity of information), identifying the most redundant and complex groups regarding their genome sequence while providing their distribution and characteristics at a large and local scale. For this purpose, we measure the sequence complexity of each available viral genome using data compression, demonstrating that adequate data compressors can efficiently quantify the complexity of viral genome sequences, including sub-sequences better represented by algorithmic sources (e.g., inverted repeats). Using a state-of-the-art genomic compressor on an extensive viral genomes database, we show that dsDNA viruses are on average the most redundant viruses while ssDNA viruses are the lowest. Contrarily, dsRNA viruses show a lower redundancy relative to ssRNA. We extend the ability of data compressors to quantify local complexity (or information content) in viral genomes using complexity profiles, unprecedently providing a direct complexity analysis to human Herpesviruses. We also conceive a features-based classification methodology that can accurately distinguish viral genomes at different taxonomic levels without using direct comparisons between sequences. This methodology works by combining data compression with simple measures such as GC-content percentage and sequence length followed by machine learning classifiers.
Conclusions: This manuscript's presents methodologies and findings that are highly relevant for understanding the patterns of similarity and singularity between viral groups, opening new frontiers for studying viral genomes' organization while depicting the complexity trends and classification components of these genomes at different taxonomic levels. The whole study is supported by an extensive website (https://asilab.github.io/canvas/) for comprehending the viral genome characterization using dynamic and interactive approaches.

Key words: Viruses; Genomics; Sequence-analysis; Data Compression; Phylogenetic-Tree; Viral Classification; Algorithmic Information Theory

Introduction

Viruses are a strong drive force of life and evolution. On average, viruses are the shortest and most abundant life realm, being estimated in around 10^{31} particles while occupying almost every ecosystem [1, 2, 3] and infecting all types of life forms, namely eukaryotes and prokaryotes [4, 5].

The dependence on the host's cell forces viruses to interact with cellular pathways to successfully hijack and customise the host cell machinery for viral production has generated a long-standing effect of adaptation and counter-adaptation between host and viruses for gene expression and nucleic acid synthe-

Compiled on: March 3, 2022.
Draft manuscript prepared by the author.

Key Points

- We provide a comprehensive landscape of the viral genomes complexity.
- We demonstrate that data compressors can efficiently quantify the complexity of viral genome sequences, including subsequences better represented by algorithmic sources.
- We identify the viral genomes with lower and higher quantity of inversions.
- We use minimal bi-directional complexity profiles as local measures of the viral genome.
- We present an in-depth complexity analysis of the human herpesviruses.
- We show that the viral genome redundancy, GC-content, and size are efficient features to accurately distinguish between viral genomes at different taxonomic levels.
- Our work opens new frontiers for studying viral genomes' complexity while depicting complexity trends in viral genomes.

sis. In addition to this co-evolution, during their replication, viruses can perform horizontal gene transfer, increasing the host species' genetic diversity analogously to the process of sexual reproduction [6].

Despite the significant impact that viruses have on the evolution of living beings and the ecosystem, their understanding is still relatively limited compared with other realms of life. In particular, the complexity landscape of viruses is unknown. What are the most redundant and complex viral DNA/RNA sequences? Which viruses contain more genetic inversions? How does the complexity distribution of viruses describe their morphology and behaviour? Additionally, analyzing the complexity of the genome sequence may uncover important information regarding viral processes and distinguish among viral characteristics. Studying the complexity (or quantity of information) of a DNA/RNA sequence requires efficient data compressors that take into account the probabilistic and algorithmic characteristics of the data.

Already, several studies have shown the high capability of data compressors as approximations of complexity. In genomics, for example, it has been used to analyse the complexity of different DNA genomes [7], perform rearrangement detection [8] and sequence clustering [9], compute phylogenetic trees [10], perform protein structure prediction [11], compare biological networks [12], and utilised in metagenomic applications [13].

This manuscript presents an extensive complexity analysis of the viral world through the automatic computational analysis of its genome complexity and associated characteristics. Specifically, we use a genomic compressor to analyse the complexity across viral taxonomies and quantify the algorithmic information embedded in viral genome sequences better represented by small programs. Several questions arise when addressing this problem: How much information is present in a viral genome? What is the best way to quantify the information in a viral genome? What type of information can we retrieve from analysing the complexity of the viral genome? To answer them, we use unsupervised probabilistic and algorithmic information quantification in viral genomes. To achieve this, we built a high-quality viral genomes database using the NCBI reference database with 12,168 complete reference genomes from 9,605 viral taxa.

To perform the complexity analysis of these genomes, we made use of the state-of-the-art genome compressor GeCo3 [14]. We compressed each viral sequence using 19 different levels and calculated its normalized compression (NC) to determine the best models to perform the analysis. With this level, we compare the compression of GeCo3 with one of the best general-purpose compressors (PAQ8) and the Block Decomposition Method (BDM) on a synthetic sequence with embedded inverted repeats (IRs). The results show that, unlike other programs, GeCo3 is capable of detecting and compressing IRs. We use this knowledge to analyse viruses regarding their complexity and overall abundance of inverted repeats and construct phylogenetic trees. We provide several insights into patterns between the complexity and viral groups. Finally, we show that these measurements can perform viral genome authentication and classification with high accuracy without directly comparing the sequences but rather using the individual features. We demonstrate that efficient data compression is crucial for understanding the viral organization according to the high reported classification accuracy.

This article is organized as follows. In the next section, we describe this paper's background and related work, followed by a description of the methods. Then, we present the main results. Finally, we review the results obtained in the discussion, draw conclusions, and point out possible future work lines.

Background

This manuscript shows that the efficient use of specific data compressors to quantify data complexity (Kolmogorov complexity) profoundly impacts viral genomes identification, classification, and organization. For introducing several concepts, this section provides an overview of the viral nature, Kolmogorov complexity and data compression, and the role of inverted repeats (IR) in the genome sequence.

Viruses Microbiology

Viruses are submicroscopic biological infectious agents that require living cells of an organism to be active for replication [15]. Viruses can exist outside of their host in the form of independent particles named virions, that are composed of the genetic material (DNA or RNA) enclosed by the capsid, which is a protein shell that protects the viral genome while it is extracellular and promotes its entry in the host cells [16].

The majority of the viruses possess capsids with helical (Figure 1 A) or icosahedral (1 B) arrangement [17, 18].

Other viruses, like bacteriophages, have developed other structures composed by elongated capsids attached to a cylindrical tailed sheet (Figure 1 C) [19].

Others have an outer lipid bilayer named viral envelope (Figure 1 D), which is constituted by a modified form of the host's cell membranes. Viroids have naked genomes, without any protective layer. Like viruses, they use the host's machinery for their replication, but their genomes do not encode proteins [20]. Furthermore, some viruses are dependent on the presence of another virus species in the host cell to be transmitted to new cells. They were named 'satellites' and may represent evolutionary intermediates of viroids and viruses [21, 22].

Viral genomes can be of double-stranded DNA (dsDNA), single-stranded DNA (ssDNA), double-stranded RNA (dsRNA)

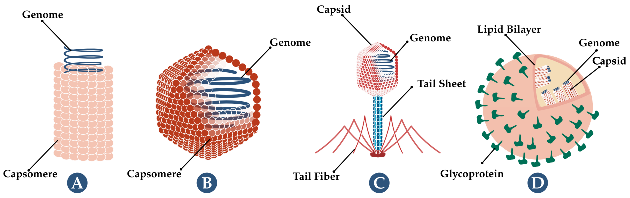

Figure 1. Illustrations of types of virus morphology. Virus **(A)** is a helical virus, where the capsoid has an helical shape that envelops the genomic material, virus **(B)** is icosahedral following cubic symmetry , **(C)** is virus covered by a viral envelop and **(D)** depicts a complex virus, namely a bacteriophage with a prolate capsid protecting the genomic material.

or single-stranded RNA (ssRNA) nature, being linear or circular molecules [23]. The ssRNA viruses can be further classified as positive- or negative-ssRNA, depending on the sense of their RNA strand. These features determine the viral replication and mRNA synthesis pathways. For instance, (+)-ssRNA is directly translated into proteins by the host cell's ribosomes, acting as mRNA. On the other hand, (-)-ssRNA needs to be converted to a (+)-ssRNA by and RNA-dependent RNA polymerase (RdRp) before translation. RdRp also transcribes dsRNA to mRNA (using the negative strand as template) and it is indispensable for the replication of RNA viral genomes. Finally, ssDNA and dsDNA normally make use of the host's DNA-dependent RNA polymerase to form mRNA. However, before this process, ssDNA is converted to a dsDNA by a DNA polymerase upon cell invasion [24], which is also the enzyme involved in the replication of DNA viruses. The RdRps have a high error rate due to their low proofreading activity and, therefore, replication of RNA viruses is much more prone to mutation than that of DNA viruses [25].

Viruses have a huge size variation, ranging from around 10 nm with small genomes to viruses with similar dimensions and genome size of Bacteria and archaea [26, 27]. These viruses are called giant viruses and contain many unique genes currently not found in other life forms.

Although the origin of viruses is still uncertain, they play an important role in the evolution of living organisms since they are horizontal gene transfer vehicles, a biological phenomenon that increases genetic diversity. It allows viral genetic material to occasionally integrate into the host genomes being transferred vertically to its offspring. This property is so preponderant in evolution that the origin of the eukaryotic nucleus might be related with this process [28, 29, 30].

Additionally, viral genomic integration allows to infer the evolutionary distance between hosts by observing the shared virus integrated into their genomes. For instance, in humans, viruses frequently establish persisting infections and imprint their genetic material in the tissues throughout life, displaying phylogeographies patterns. These can be used as markers to better understand the human population history and migrations and provide new insights into unidentified individuals' origins in both global and local scales [31]. In this respect, the JC polyomavirus is one of the most comprehensively studied virus. Its genotype-specific global spread has been suggested to indicate the origins of modern [32] and ancient humans [33, 34, 35]. Furthermore, a worldwide study supported the co-dispersal of this virus with major human migratory routes and its co-divergence with human mitochondrial and nuclear markers [36].

Thus, performing computer analysis of viral and host DNA sequences is fundamental to understand the evolutionary relationships between different viruses and their hosts, identify the ancestors of modern viruses, and better understand their behavior and function. Also, the genomic sequences encode not only production of proteins, but also their high-dimensional folding structure [37, 38]. Therefore, the direct study of viral genome sequences also develops the knowledge of the viral mechanism of protein formation and assembly.

Inverted Repeats

IRs are nucleotide sequences that have a downstream reverse complement copy, causing a self-complementary base pairing region [39]. Consequently, IRs normally fold into different secondary structures (hairpin- and cruciform-like structures, pseudoknots) that participate or interfere in many cellular processes in all forms of life, including DNA replication [40, 25]. Due to these traits, IRs perform an essential role in genome instability [41], which contributes to mutability. In the short term, this mutability can create diseases [42], but across long periods lead to cellular evolution, and genetic diversity [43]. In many viruses, IRs in the form of pseudoknots are involved in ribosomal frameshifting, a translational mechanism that allows the production of different proteins encoded by overlapping open reading frames (ORFs) of the same mRNA [44, 45]. This feature allows them to encode a larger amount of genetic information in small genomes and constitutes another level of gene regulation [46]. The genomes of some viruses, such as parvovirus, are flanked by inverted terminal repeats (ITRs) that form hairpin structures functioning as a duplex origin of replication sequence [40, 47]. Therefore, these ITRs contain most of the cis-acting information needed for viral replication as well as viral packaging [47]. In adeno-associated viruses, ITRs are essential for intermolecular recombination and circularization of genomes [48]. IRs can also function as termination transcription signals, especially in giant viruses [49, 50].

Kolmogorov Complexity and Data Compression

Solomonoff, Kolmogorov, and Chaitin [51, 52, 53, 54] described the notion of complexity by showing that there is at least one optimal algorithm among all the algorithms that decode strings from their codes. For all strings, this algorithm allows codes as short as any other, up to an additive constant that depends only on the strings themselves. Concretely, algorithmic information is a measure that quantifies the information of a string x by determining its complexity $K(x)$ by

$$K(x) := \min_{p}\{l(p) : U(p) = x\}, \tag{1}$$

where $K(s)$ is defined by a shortest length l of a binary program p that computes the string x on a universal Turing machine U and halts [53]. This notion that the complexity of a string can be defined as the length of a shortest binary program that outputs that string was universally adopted and is the standard to perform information quantification. It differs from Shannon's entropy because it recognises that the source creates structures which follow algorithmic schemes [55, 56], rather than regarding the machine as generating symbols from a probabilistic function.

While the Kolmogorov complexity is non-computable, it can be approximated with programs for such purpose. A possible approximation is the Coding Theorem Method (CTM) [57], and its improved version, the Block Decomposition Method (BDM) [58], which approximate local estimations of algorithmic complexity providing a closer relationship to the algorithmic nature. This approximation decomposes the quantification of complexity for segmented regions using small Turing machines [57]. For modelling the statistical nature, such as noise, it commutes into a Shannon entropy quantification. This approach has shown encouraging results for many distinct purposes [59, 60, 61].

The classical approximation of the Kolmogorov complexity is performed using data-compressors with probabilistic and algorithmic schemes. Data compressors are a natural solution to measure complexity, since, with the appropriate decoder, the bitstream produced by a lossless compression algorithm allows the reconstruction of the original data and, therefore, can be seen as an upper bound of the algorithmic complexity of the sequence. For a definition of safe approximation, see [62].

In genomics, sequences can be codified as messages using a four symbol alphabet ($\Sigma = \{A, C, G, T\}$ for DNA sequences and $\Sigma = \{A, C, G, U\}$ for RNA sequences). These messages contain instructions for survival and replication of the organism, its' morphology and historical marks from previous generations [63]. Initially, genomic sequences were compressed with general-purpose data-compressors such as gzip [64], bzip2 [65], or LZMA[66]. However, this paradigm shifted towards using a specific compression algorithm after introducing BioCompress [67]. Genomic compressors can outperform general-purpose compressors since they are designed to consider specific genomic properties such as the presence of a high number of copies and substitutional mutations, and multiple rearrangements, such as inverted repeats [68, 69].

Given this advantage of using specific compressors for the compression of genomic data, several algorithms have emerged to model these genomic data behaviours [70]. Specifically, algorithms have been created that model repetitions and inverted repetitions in the genome regions through simple bit encoding, dictionary approaches and context modelling [71, 72, 73, 74, 75, 76, 77, 78, 79, 80, 81].

Currently, the state-of-the-art genomic compressors apply statistical and algorithmic model mixtures combined with arithmetic encoding. The best compression ratio performance for various genomic sequences is provided by XM [82], Jarvis [83], and Geco3 [14]. The XM compressor [82] uses three types of experts: repeat models, a low-order context model, and a short memory context model. On the other hand, Jarvis [83] uses a competitive prediction model that estimates for each symbol the best class of models to be used. There are two classes of models: weighted context models and weighted stochastic repeat models, where both classes of models use specific sub-programs to handle inverted repeats efficiently. Finally, GeCo3 [14], currently the best performing reference-free compressor, uses neural networks to improve upon the results of specific genomic models of GeCo2 [84]. Specifically, the neural networks are used in mixing multiple contexts, and substitution-tolerant context models of GeCo2 [84]. Furthermore, GeCo3 possess embedded subprograms capable of detecting genome-specific patterns, such as inverted repeats.

Methods

This section describes the measures used in this paper. Specifically, we first define information-based measures: the Normalized Block Decomposition Method, the Normalized Compression (NC) with different subprograms, the normalized compression capacity (NCC), the difference between NCs, and the minimal bi-directional complexity profiles. Afterwards, we define the GC-Content, and the compression benchmark performed. Finally, we described the classification pipeline. Specifically, the features and classifiers used and the metrics utilized for evaluating the model's performance.

Information-based measures

This section describes two approximations of the Kolmogorov complexity, one based on the decomposition of a string into blocks and their approximation based on the output of small Turing machines (Block Decomposition Method) and another based on data compression. The data compression approach was utilized to compute the Normalized Complexity and construct the minimal bi-directional complexity profiles. Therefore, we describe the Normalized Compression (NC), the minimal bi-directional complexity profiles, and the Normalized Block Decomposition Method (NBDM), in this subsection.

Normalized Block Decomposition Method (NBDM)

A possible approximation of the Kolmogorov complexity is given by using small Turing machines (TM), which approximate the components of a broader representation. The Coding Theorem Method (CTM) uses the algorithmic probability between a string's production frequency from a random program and its algorithmic complexity. The more frequent a string is, the lower its Kolmogorov complexity, and the lower frequency strings have, the higher Kolmogorov complexity is. The Block Decomposition Method (BDM) increases the capability of a CTM, approximating local estimations of algorithmic information based on Solomonoff-Levin's algorithmic probability theory. In practice, it approximates the algorithmic information and, when it loses accuracy, it approximates the Shannon entropy. Since in this article we use BDM to perform a comparison with the Normalized Compression, we considered the normalization of the BDM (NBDM) according to [85]. In this case, the NBDM is computed as

$$\mathrm{NBDM}(x) = \frac{BDM(x)}{|x| \log_2 |A|} = \frac{BDM(x)}{2 \times |x|}. \qquad (2)$$

where x is a string, $BDM(x)$ is the BDM value of the string, $|A|$ the number of different elements in x (size of the alphabet) and $|x|$ the length of x. Since we have a four symbol alphabet ($\Sigma = \{A, C, G, T\}$ for DNA sequences and $\Sigma = \{A, C, G, U\}$ for RNA sequences), $|A| = 4, \log_2 4 = 2$. Although BDM has difficulty dealing with full information quantification due to the block representability, it has proven to be a helpful tool for measuring and identifying data content similar to simple algorithms [85].

Normalized Compression (NC)

An efficient compressor, $C(x)$, provides an upper bound approximation for the Kolmogorov complexity ($K(x)$), where $K(x) < C(x) \leq |x|$ ($|x|$ is the length of string x in the appropriate scale). Usually, an efficient data compressor is a program that approximates both probabilistic and algorithmic sources using affordable computational resources (time and memory). Although the algorithmic nature may be more complex to model, data compressors can have embedded sub-programs to handle this nature. The normalized version, known as the Normalized Compression (NC), is defined by

$$\mathrm{NC}(x) = \frac{C(x)}{|x| \log_2 |A|} = \frac{C(x)}{2 \times |x|}, \qquad (3)$$

where $C(x)$ is the compressed size of x in bits. Given the normalization, the NC enables to compare the proportions of information contained in the strings independently from their sizes [7]. If the compressor is efficient, then it can approximate the quantity of probabilistic-algorithmic information in data using affordable computational resources. In our work, to determine the NC, we made use of the state-of-the-art genome compressor GeCo3 [14], with the level that yielded the best average results (benchmark provided in the results section).

Besides the computation of the NC using the standard configuration of this model, we also computed the NC using GeCo3 with three subprogram configurations. These subprogram con-

figurations address the use or absence of inverted repetitions, namely:

- $IR_0 \rightarrow$ uses the regular context model without IR detection;
- $IR_1 \rightarrow$ uses IR detection simultaneously with the regular context model;
- $IR_2 \rightarrow$ uses IR detection sub-program without regular context models.

There was a need to determine the sequences with the highest normalized compression capacity (NCC) in some cases. When the compressor was only using the subprogram IR_2, NCC was computed as $NCC_{IR_2}(x) = 1 - NC_{IR_2}$. Only positive values were considered to filter computations where the compressor could not compress the sequence sufficiently. Another measure used to quantify inverted repeats was the difference between NC_{IR_0} and NC_{IR_1}.

Minimal bi-directional complexity profiles

A complexity profile is a numerical sequence describing for each symbol (x_i) of a sequence x the number of bits required for its compression assuming a causal order [86]. A minimal bi-directional complexity, $B(x)$, profile assumes the minimal representation of compressing the sequences using both directions independently, namely $\overrightarrow{C}(x_i)$ as from the beginning to the end of the sequence, and $\overleftarrow{C}(x_i)$ as from the end to the beginning. Accordingly, these profiles are defined as

$$B(x_i) = \min\{\overrightarrow{C}(x_i), \overleftarrow{C}(x_i)\}. \tag{4}$$

The construction of these profiles follows a pipeline formed of many transformations, including reversing, segmenting, inverting, and the use of specific low-pass filters after data compression to achieve better visualization. For computing these profiles, we use the GTO toolkit [87].

The generation of these profiles is robust to localize specific features in the sequences, namely low and high complexity sequences, inverted repeat regions, duplications, among others.

Other Measures

The two other measures used to perform viral analysis and classification are the GC-Content (GC) and the length of the viral genome $|x|$.

GC-Content (GC) represents the proportion of guanine (G) and cytosine (C) bases out the quaternary alphabet $\{A, C, G, T/U\}$. This includes thymine (T) in DNA and uracil (U) in RNA. The GC percentage is given by the number of cytosine (C) and guanine (G) bases in a viral genome x with length $|x|$ according to

$$\mathcal{GC}(x) = \frac{100}{|x|} \sum_{i=1}^{|x|} \mathcal{N}(x_i || x_i \in \Xi), \tag{5}$$

where x_i is each symbol of x (assuming causal order), Ξ is a subset of the genomic alphabet containing the symbols $\{G, C\}$ and $\mathcal{N}$ the program that counts the numbers of symbols in Ξ.

GC-content is variable between different organisms. In addition, the GC-content value correlates with the organism's life-history traits, genome size [88], and GC-biased gene conversion [89]. As such, this measure is useful to perform viral classification. Furthermore, an organism with a genome high in GC-content is rich in energy and more prone to mutation. Thus, over time, a species tends to decrease its GC-content to become more stable [90], giving us further information regarding viral characterization.

Data Description

The dataset is composed of 12,163 complete reference genomes from 9,605 viral taxa retrieved from NCBI database on 22 of January 2021 using the following url https://tinyurl.com/ncbidtbs. The download was performed in a custom manner to retrieve the taxonomic id, host and geolocation of each reference genome. The metadata header was removed from each sequence using the GTO toolkit [87], where any nucleotide outside the quaternary alphabet $\{A, C, G, T/U\}$, was replaced by a random nucleotide from the quaternary alphabet. Notice that the sequences with symbols outside the alphabet are scarce. Finally, the type of genome and the taxonomic description of each sequence were retrieved using Entrez-direct [91].

Then, the retrieved NCBI sequences were filtered to remove possibly contaminated or poorly sequenced sequences. Firstly, using the taxonomic metadata, sequences that did not hold complete taxonomic information down to the genus rank and any sequences that maintained a taxonomic description of unclassified were removed. Secondly, a filter was applied to remove outlier sequences. Specifically, after computing all sequences' length, GC-Content, and Normalized Complexities, sequences whose measure fell outside $\mu \pm 3 \times \sigma$ (approximately 0.03% of all sequences) of any measure were removed. After filtering, 6,091 of the initial 12,163 sequences were kept.

Compression Model Benchmark

We selected a total of 19 levels of models to determine the best level configuration to compress the viral sequences. These levels correspond to the default 13 levels of the GeCo3 compressor and 6 others built for this task. The list of the levels used are shown in Table S1, and the description of parameters can be found in Table S2. The 13 default levels of the compressor have increasingly higher complexity and take longer to run since they use higher context models. Therefore, since the first and lightest level performed best, the other six custom-build levels were also built with small models.

Classification

We tested several machine learning algorithms to perform the genomic and taxonomic classification task, namely, the classifiers used were Linear Discriminant Analysis (LDA) [92], Gaussian Naive Bayes (GNB) [93], K-Nearest Neighbors (KNN) [94], Support Vector Machine (SVM) [95], and XGBoost classifier (XGB)[96].

Linear Discriminant Analysis is a generalization of Fisher's linear discriminant, a method used in statistics and other fields, to find a linear combination of features that separates classes of objects. The resulting combination can be used as a linear classifier [92]. Gaussian Naive Bayes is defined as a supervised machine learning classification algorithm based on the Bayes theorem following Gaussian normal distribution [93]. K-Nearest Neighbors is another approach to data classification, taking distance functions into account and performing classification predictions based on the majority vote of its neighbors [94]. Support Vector machines are supervised learning models with associated learning algorithms that construct a hyperplane in a high-dimensional space using data and perform classification [95]. Finally, XGBoost [96] is an efficient open-source implementation of the gradient boosted trees algorithm. Gradient boosting is a supervised learning algorithm that predicts a target variable by combining the estimates of a set of simpler models. Specifically, new models are created that predict the residuals or errors of prior models and then added together to make the final prediction. This task uses a gradient descent

algorithm to minimize the loss when adding new models. XGBoost can use this method in both regression and classification predictive modeling problems.

The accuracy and weighted F1-score were used to select and evaluate the classification performance of the measures. Accuracy is the proportion between correct classifications and the total number of cases examined, while the F1-score is computed using the precision and recall of the test. We utilized the weighted version of the F1-Score due to the presence of imbalanced classes.

For comparison of the obtained results, we assessed the outcomes obtained using a random classifier. For that purpose, for each task, we determined the probability of a random sequence being correctly classified (p_{hit}) as

$$p_{hit} = \sum_{i=0}^{n} [p(c_i) * p_{correct}(c_i)], \quad (6)$$

where $p(c_i)$ is the probability of each class, determined as

$$p(c_i) = \frac{|samples_{class}|}{|samples_{total}|}.$$

On the other hand, $p_{correct}(c_i)$ is the probability of that class being correctly classified. In the case of a random classifier,

$$p_{correct}(c_i) = \frac{1}{|classes|}.$$

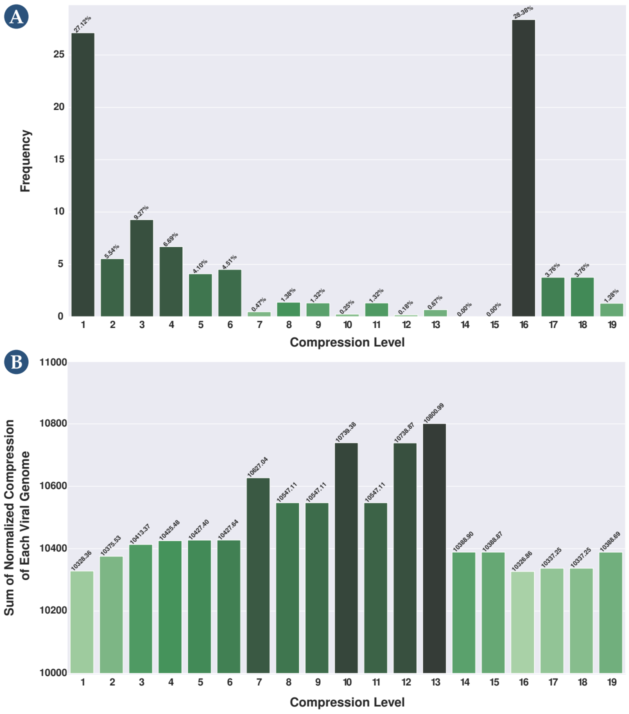

Figure 2. Selection of a level for GeCo3 from a pool of 19 levels. **A** depicts the frequency where each level provided the best NC results, and **B** shows for each level the sum of the NC from the compression of all reference genomes. For better visualization, visit the website https://asilab.github.io/canvas/.

Results

The results reported in this manuscript can be computed using the minimal characteristics described in Supplementary Subsection entitled Software and Hardware recommendations and using the procedures described in Supplementary Subsection entitled Reproducibility. The following subsections describe the data, the compression level selection benchmark, the synthetic sequence benchmark, the viral genome analysis and phylogenetic trees, and the viral classification application.

Level selection benchmark

Viral genomes have specific characteristics, for example, short length, high average complexity, and specific structures, that require the proper optimization of the data compressor to provide higher modeling adaptability and efficiency. GeCo3 is a state-of-the-art genomic compressor that contains many types of compression levels [14]. Herein, we used this tool to compress each viral genome from the dataset using 19 different levels and computed its normalized compression (NC).

We evaluated the frequency where each level yielded the lowest NC (provided the best compression for a given sequence; Figure 2 A) and determined the sum of the NC from the compression of all reference genomes for each model (Figure 2 B). Overall, level 16 was selected because it provided the lowest NC on average (selected 28.38% as the best compression level) and provided the lowest sum of the NC from compressing all reference genomes. This level is constituted by a mixture using a neural network with the following models:

- Model 1 → context-order of 1, alpha parameter of 1 (without inverted repeats), and gamma parameter of 0.7;
- Model 2 → context-order of 12, alpha parameter of 1/50 (with inverted repeats), and gamma parameter of 0.97.

The chosen level is constituted by two models with a small and average context model. This configuration performed better because most of the viral genomes are small and compact, where repetitions and IRs are usually separated by a small genomic space. Therefore, the depth of the models is more adapted to provide higher efficiency to the average of the viral genomes than, for example, a higher context model (higher than 13) that can perform marginally better in more extensive and repetitive sequences but that loses sensitivity in the average of the genomes.

Synthetic sequence benchmark

Viral genomes can contain IRs that are subsequences better described using simple algorithmic approaches. To benchmark the capability of different programs to quantify IRs accurately, we created a genomic sequence of 10,000 nucleotides in which the last 5,000 were inverted repeats of the first 5,000. This sequence was mutated incrementally from 0% to 10%, meaning that the number of IRs will decrease with the increase of nucleotide substitutions. For each sequence, the NC was computed with (Figure 3): i) GeCo3, without and with IR detection program (IR_0 and IR_2, respectively) and ii) PAQ8 data compressor (one of the best general-purpose data compressors). Additionally, the Normalized Block Decomposition Method (NBDM) was also computed, as a measure more prone for the algorithmic nature quantification. Results show that GeCo3 with the IR_2 subprogram compresses the sequences better than the other programs since its NC is lower at 0% mutational rate (Figure 3). NBDM can also not detect the IRs because it provides the same high value across all sequences with various mutation rates. It is also evident that GeCo3 with IR_2 can detect IRs even in the presence of substantial mutations (5% of mutation) and takes into account different levels of nucleotide substitutions because increases with the increase of the mutational rate (i.e. decrease of IRs). The difference between NC_{IR_0} and NC_{IR_1}, both computed with GeCO3, was also analyzed. Its profile is inverse

to the IR_2 and confirms that nucleotide substitutions' accumulation decreases the number of IRs in the sequence.

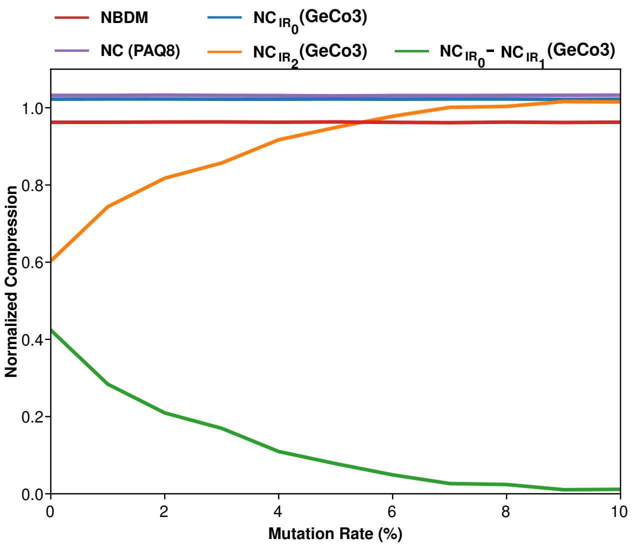

Figure 3. Plot describing the variation of Normalized Compression (*NC*) and Normalized Block Decomposition Method (NBDM) with an increase of mutation rate of a sequence (0%-10%). The NC was computed using the state-of-the-art genomic compressor (GeCo3 [14]) and a general-purpose compressor (PAQ8 [97]). The NBDM is depicted by a red line and the NC value using PAQ8 by a purple line. Furthermore, the GeCo3 compressor with (IR_2) and without the IR detection subprogram (IR_0) is shown in orange and blue lines, respectively. Finally, the green line shows the difference between NC_{IR_0} − NC_{IR_1}.

Viral genome analysis and Phylogenetic trees

The core of the viral genomes was analyzed in terms of complexity landscape, including the trends, singularities, and patterns for both the use or absence of IRs. The NC, using GeCo3, with IR_0, IR_1 and IR_2 subprograms was determined and the NCC_{IR_2} was calculated. The outcome was interpreted according to the genome type or the taxonomic group, together with the average of their genome sizes (Figure 4 and Table S3). Notice that the NC enables to compare proportions of the absence of redundancy independently from the sizes of the genomes. This value is complementary to the normalized redundancy. Specifically, consider the redundancy (R) of a sequence x, as $R(x) = log_2(A)|x| - C(x)$, where $|x|$ is the length of the sequence, A is the cardinally of the sequences' alphabet and C(x) is the compressed size of x in bits, and the normalized redundancy (NR) as $NR(x) = 1-(C(x)/log_2(A)|x|)$.

Complexity landscape according to genome type

According to NCBI, the virus's genomes herein analyzed are of five types: dsDNA, ssDNA, dsRNA, ssRNA and mixed-DNA. Results show that ssDNA, followed by mixed-DNA and dsRNA viruses, are the genomes with higher NC, whereas dsDNA genomes have the lowest (Figure 4 A; Table S3). In general, smaller genomes are less complex and are more likely to contain fewer repeats and, hence, less redundancy, and the ssDNA, mixed-DNA and dsRNA genomes have smaller average sequence lengths (3282 bp, 3258 bp, and 8377 bp; Table S3).

According to the *NCC* and the NC_{IR_0}−NC_{IR_1} difference results, dsDNA and ssDNA have most significant quantities of IRs than the other genome types. This can be due to ITRs present at the ends of some dsDNA viruses, such as Adenovirus and Ampullaviruses, and ssDNA virus as Parvoviruses, or other IRs structures important that perform ribosomal frameshifting.

Complexity landscape according to taxonomic level

In complexity analysis of viral genomic sequences, when considering the Realm taxonomic level (Figure 4 B), the lowest NC values were obtained for Adnaviria, Varidnaviria and Duplodnaviria (Table S4 and S5). These results are consistent with the genomic grouping since they are composed exclusively of dsDNA viruses and have the highest sequence lengths. Thus, generally, an inverse correlation between genome size and NC was also observed as with the genome type analysis (Figure 4 A and B) and occurs across all taxonomic levels (Table S5). However, within these three Realms, Adnaviria has the lowest sequence length and presented a lower NC than Varidnaviria and Duplodnaviria, suggesting that the last are highly complex.

Regarding IRs, Adnaviria was the realm where the highest compression was obtained using the IR_2 subprogram (highest rate of IRs; Table S6). Consequently, its only recognized kingdom, Zilligvirae, has also one of the highest NCC values (Table S6). Adnaviria is a realm constituted of mostly A-form dsDNA viruses, and the ends of their genomes contain ITRs [98]. A-form is proposed to be an adaptation allowing DNA survival under extreme conditions since their hosts are hyperthermophiles and acidophiles microorganisms from the archaea domain [98, 99]. The fact that Adnaviria presented the lowest NC might indicate that their genomes require redundancy to survive such extreme environments. The kingdom Trapavirae, belonging to the realm Monodnaviria, is also composed by dsDNA viruses that infect halophilic archaea. Together with kingdom Zilligvirae, Trapavirae presented the highest difference between IRs and standard compression (Table S7). These results also support the fact that IRs can stabilize the DNA of viruses that exist in extreme environments. It has already been demonstrated that archaeal viruses with linear genomes use diverse solutions for protection and replication of the genome ends, such as including covalently closed hairpins and terminal IRs [100].

At the family level, Botourmiaviridae presented the highest complexity, followed by Alphasatellitidae and Tolecusatellitidae families (Table S5). Botourmiaviridae is composed of ssRNA viruses that infect plants, and filamentous fungi [101]. Curiously, plants and fungi have higher redundancy despite the lower redundancy of their pathogens. Alphasatellitidae and Tolecusatellitidae are families of satellite viruses that depend on the presence of another virus (helper viruses) to replicate their genomes. These satellite viruses have minimal genomes, making sense that they possess very low redundancy. Regarding IRs, Malacoherpesviridae, Herpesviridae, and Rudiviridae contained the highest NC_{IR_0}−NC_{IR_1} difference (Table S7). Malacoherpesviridae and Herpesviridae are dsDNA viruses evolutionarily close since they belong to the order Herpesvirales [102]. Malacoherpesviridae encompasses the genera Aurivirus and Ostreavirus, which infect molluscs. Herpesviridae are also known as herpesviruses and have reptiles, birds and mammals as hosts. This family will be discussed in more detail in the following subsection. Rudiviridae is a family of viruses with linear dsDNA genomes that also infect archaea. The virus of these families are highly thermostable and can act as a template for site-selective and spatially controlled chemical modification. Furthermore, the two strands of the DNA are covalently linked at both ends of the genomes, which have long ITRs [103]. Again, these IRs could be an adaptation to stabilize the genome.

Complexity landscape of the family Herpesviridae

Here we analyzed the complexity landscape of the genera of the family Herpesviridae in more detail, and results show a significant variation between them (Figure 5 A). Mardivirus had the highest NC_{IR_0} − NC_{IR_1} difference among all viruses, and only other three genera (out of thirteen) of herpesviruses

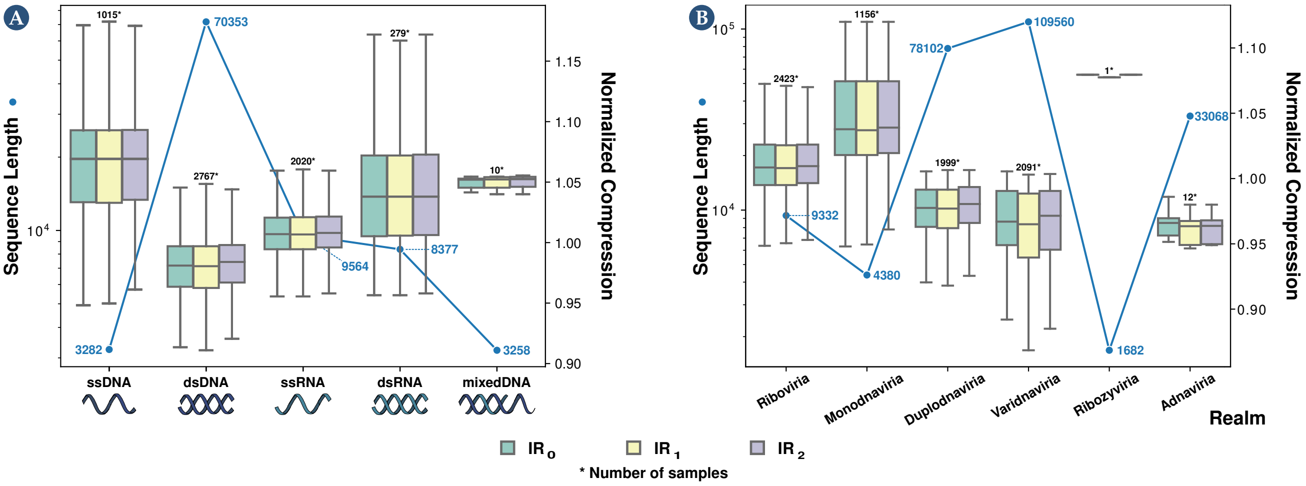

Figure 4. Average Normalized Compression (ANC) and average sequence length per viral group. The values were obtained for genome type (*A*) and realm (*B*). To view all boxplots by groups of realm, kingdom, phylum, class, order, family, and genus, visit the website https://asilab.github.io/canvas/.

were within the ten highest differences list (Table S7). Indeed, the genus Mardivirus had the highest compression, whereas the genus Lymphocryptovirus possessed very low compression with the IR_2 subprogram. We performed the minimal bi-directional complexity profiles of one sequence of each virus to visualize their distribution of complexity locally (Figure 5 C). As we can see, Human herpesvirus 4 (also known as Epstein-Barr virus) has more internal repeats (Figure 5 C, IR_0 profile) detected and fewer IRs (Figure 5 B; IR_2 profile). The opposite occurs with the Falconid herpesvirus-1 strain S-18, where IRs are more prominent than internal repetitions. Furthermore, notice that these regions determined with compression profiles coincide with actual regions detected in the genome with other methods (Figure 5 C; first profile).

A particular group of family Herpesviridae are the human herpesviruses (HHVs). These viruses are involved in globally prevalent infections and cancers and characterized by lifelong persistence with reactivations that can potentially manifest life-threatening conditions [104]. Globally, the HHVs present a higher redundancy relative to other viruses (Figure 5 B). These viruses are divided into: i) the alpha-subfamily members, namely herpes simplex virus type 1 and 2 (HSV-1 and HSV-2) and varicella-zoster virus (VZV), ii) the beta-subfamily of human cytomegalovirus (HCMV) and human herpesviruses 6A, 6B, and 7 (HHV-6A, HHV-6B, and HHV-7) and iii) the gamma-subfamily of Epstein-Barr virus (EBV) and Kaposi's sarcoma-associated herpesvirus (KSHV). Specifically, the EBV, one of the most potent cell transformation and growth-inducing viruses known, capable of immortalizing human B lymphocytes, contains a higher redundancy than the other HHVs (Figure 5 B). The other gamma-herpesvirus, KSHV, is the genome with the highest NC_{IR_1} (Figure 5 B). Unlike the beta- and gamma-subfamilies, the alpha-subfamily is characterized by a substantial quantity of IRs, as suggested by the NCs with IR_1 and IR_2 configurations (Figure 5 B). The VZV has the shortest genome and the highest NC within this group. These differences might be justified by the different rates of evolution within these genomes [105]. Considering the beta-subfamily members, HCMV contains a small proportion of IRs while having a substantial-high NC relative to most other HHVs being analyzed. Since the HCMV has the largest genome, this was surprising because the NC typically has an inverse correlation with the genome size and the quantity of IRs. The other beta-subfamily members are the Human Herpesvirus 6A, 6B, and 7, which produced lower NCs (with IR_1 and IR_2 configurations) compared to the other HHVs, with a low quantity of IRs, an effect that their integrating function might favour. For instance, HHV-6A and 6B integrate their genomes into the telomeres of latently infected cells [106, 107]. Thus, their genomes contain subsequences similar to the human telomere regions that can be formed by internal nucleotide repetitions [108]. As such, these are sequences with very low complexity and, hence, highly compressible.

Alternative visualization methods of the viral complexity landscape

Phylogenetic trees were generated depicting the redundancy (NC; Figure 6 A) and the prevalence of inverted repeats (NCC; Figure 6 B) on each taxonomic branch. In addition, we performed the same analysis to portray the relation between inverted and internal repetitions (Figure S1). These phylogenetic trees show the broad picture of the regions with more complex and less redundant sequences, regions rich in inverted repeats, and regions with a higher prevalence of inverted repeats relative to standard repetitions in the genomes.

Another way to analyze the results is by producing 3D-scatter plots of randomly sampled values obtained from computing the features sequence length (SL), NC and GC-content (GC; Figure 7 A) or 2D-scatter plots of their projections (Figure 7 B and 7 C), both concerning a particular taxonomic level (herein Realm). Analyzing the sequence length projections (Figure 7 B), it is evident that there is a logarithmic downtrend of the NC with the increase in sequence length. Thus, although longer sequences have, on average, greater complexity (absolute quantities), they have higher redundancy, which the data compressor takes advantage of to perform a better compression. On the other hand, the NC vs the GC-content displays a normal distribution around the 0.5 GC-mark, with higher complexities associated with similar frequency of occurrence of the four bases A, C, G, T/U (Figure 7 C). This result also makes sense since, in principle, a well-distributed frequency of bases makes more complex sequences to compress. More importantly, the NC, GC and SL seem to discriminate between different taxonomic groups (Figure 7). As such, in the following section, we analyze the classification capability of these features.

Viral Classification

In this section, we performed eight different classification tasks for each viral sequence from the dataset. Specifically, the sequences were classified regarding their genome type, realm, kingdom, phylum, class, order, family, and genus.

We conducted a random 80-20 train-test split on the

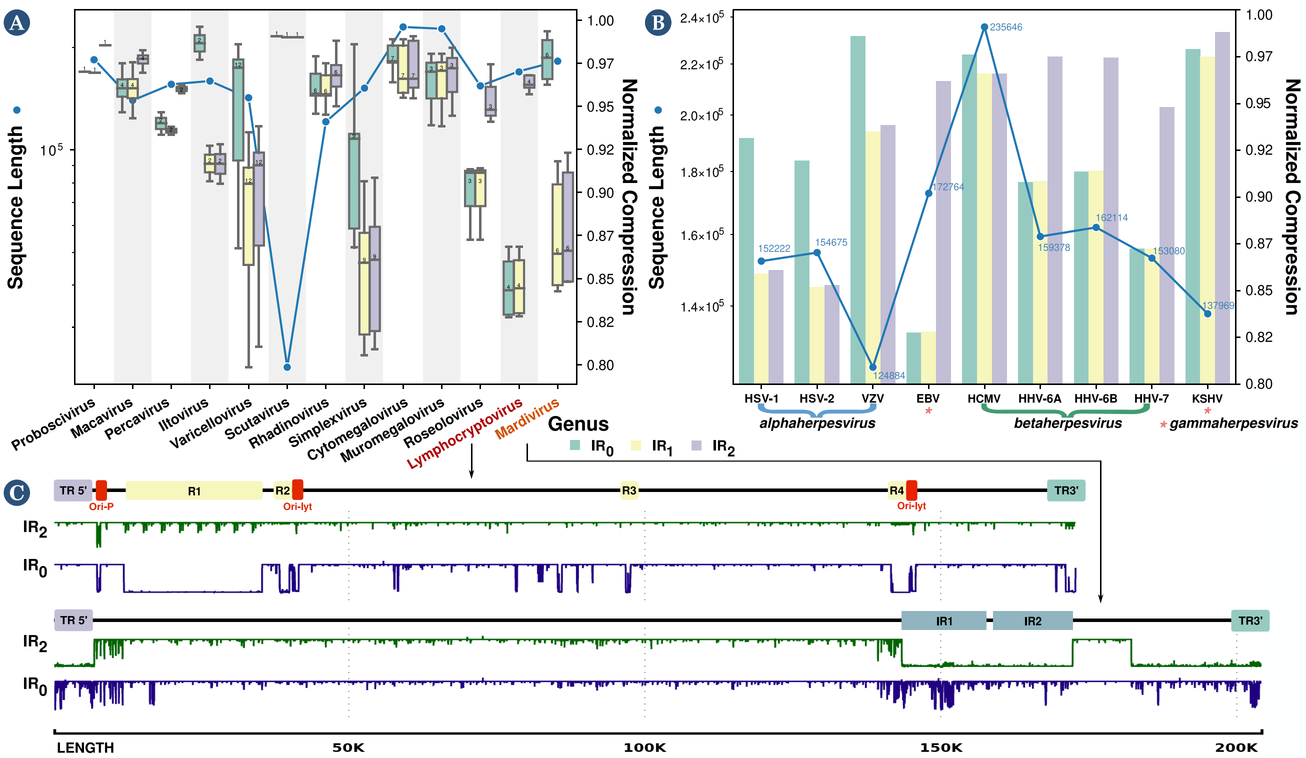

Figure 5. Average Normalized Compression (ANC) and average sequence length per the genera of the Herpesviridae family (*A*) and for various Human Herpesviruses (from type 1 to 8, *B*). In the boxplot where the genera of the Herpesviridae family are displayed, two genera were selected, one with a low level of inverted repeats (Lymphocryptovirus) and one with a high (Mardivirus). Then, a representative reference sequence was selected (Lymphocryptovirus - Human herpesvirus 4 or EBV, NCBI Reference Sequence: *NC*_024450.1; Mardivirus - Falconid herpesvirus 1 strain S-18, NCBI Reference Sequence: *NC*_009334.1) and created minimal bi-directional complexity profiles (**C**).

dataset to perform viral classification. Due to classes being imbalanced in the dataset, several actions were performed. First, we did not consider classes with less than four samples. As such, depending on the classification task, the number of samples decreased from 6,091 to the values shown in Table S8 (N. Classes column). Secondly, we performed the train-test split in a stratified way to ensure the representability of each label in the train and test sets. Finally, instead of performing k-fold cross-validation, we performed the random train-test split fifty times, and we retrieved the average of the evaluation metrics. Then, we computed the Accuracy and the Weighted F1-score to select the best performing method.

As described in the method section, we applied 5 types of classifiers: Linear Discriminant Analysis (LDA) [92], Gaussian Naive Bayes (GNB) [93], K-Nearest Neighbors (KNN) [94], Support Vector Machine (SVM) [95] and XGBoost classifier (XGB)[96].

Furthermore, we performed classification using seven different features: sequence length (SL), GC-content (GC), the Normalized Compression (NC) values for the best performing model, and the NC of the same model with IR configuration to 0, 1 and 2.

These seven features were fed to all the classifiers, and the accuracy and weighted F1-score were measured to determine which classifier was best suited for this task.

Tables S8 and S9 depict the accuracy and weighted F1-score values obtained for each classifier. For all classification tasks, the best performing classifier was the XGBoost classifier.

Following this, we analyzed if all features were necessary. For that purpose, the XGBoost classifier was used with only the NC feature, the NC with SL and GC, and finally, using all features. The obtained accuracies are shown in Table 1, and the weighted F1-score results are shown in Table S10. Except for the genome classification, where the usage of the NC, GC and SQ provided slightly better results than using all features, the remaining results yielded the best result when using all features. This improvement increased when the number of classes was higher, demonstrating that the different compression subprograms (IR_0, IR_1, and IR_2) are more helpful in classifying more specific taxonomic groups.

Regarding the results, there is decrease in accuracy and F1-score when there is an increase in the number of classes. Specifically, we obtained the best performance in the realm classification of the virus (accuracy - 92.41%, F1-score - 0.9214) and our lowest performance in genus classification (accuracy - 68.42%, F1-score - 0.6525). This decrease is mainly because the average number of samples per class decreases as the number of classes increases. As such, many classes may have insufficient number of samples to be accurately classified. Figure S2 represents the number of samples (genome sequences) per viral genus. We minimized this impact by removing classes from the classification that possessed less than four samples. Furthermore, part of the classification inaccuracies can be explained by possible errors in the assembly process of the original sequence or eventual sub-sequence contamination of parts of the genomes. Moreover, other inaccuracies could be due to several genomes being reconstructed using older methods that have been improved since then [109].

As far as we know, this is the first attempt at performing this type of reference-free classification. As such, for comparison purposes, we assessed the outcomes obtained using a random classifier. Specifically, for each task, we determined the probability of a random sequence being correctly classified (p_{hit}). Overall there is a vast improvement relative to the random classifier, showing the importance of the features used in the classification process. These classification results seem promising, showing that this metric can be utilized for viral taxonomic classification if enough sequence samples are pro-

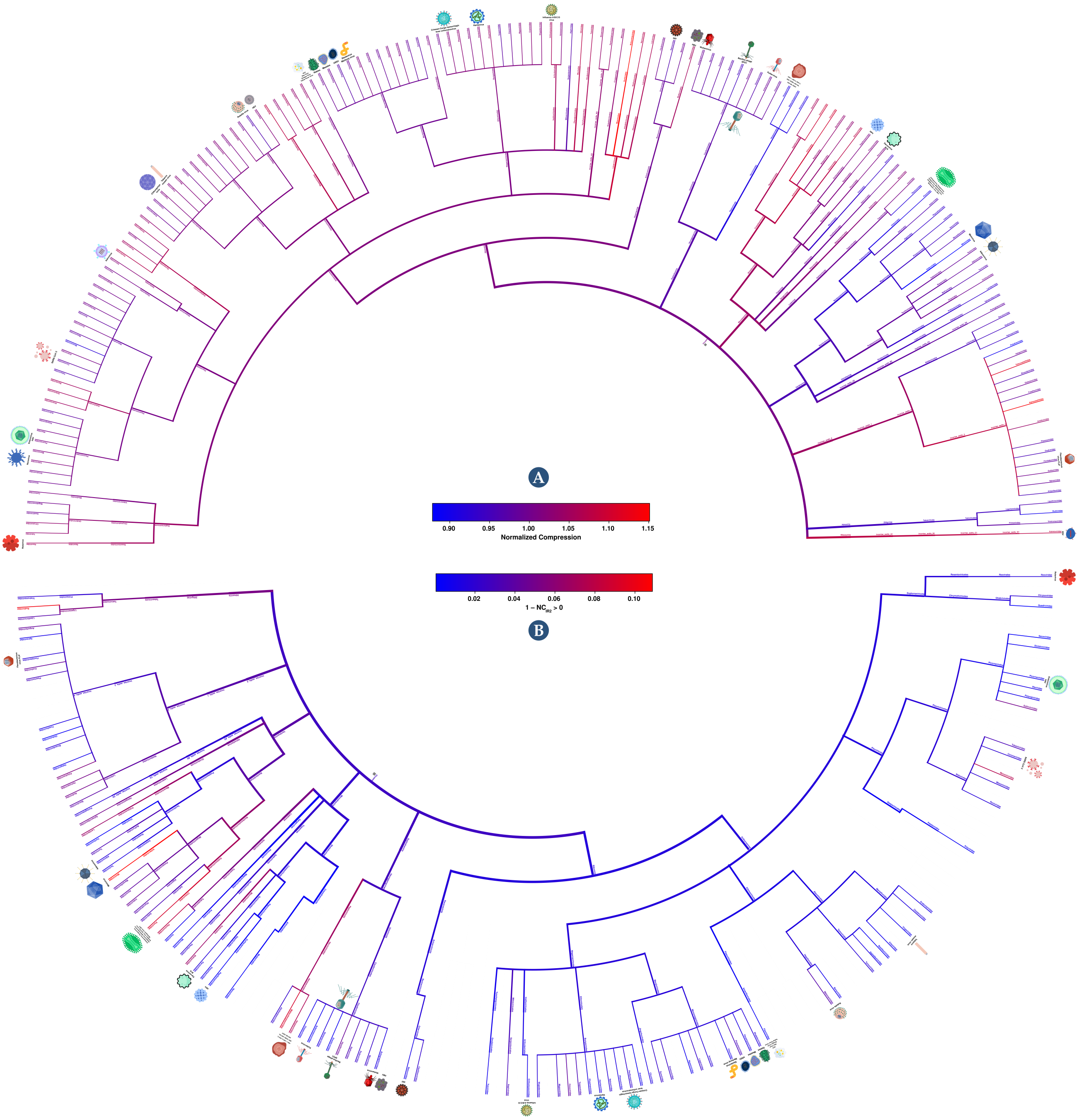

Figure 6. Phylogenetic tree showing average NC of each viral group (**A**), and the normalized compression capacity (*NCC*) (**B**). *NCC* results were obtained by $NCC = 1 - NC_{IR_2} > 0$. The colour red depicts the highest complexity, and the blue the lowest. The first phylogenetic tree describes the NC of each taxonomic branch. Red colour show genomes with less redundancy, and blue ones with more redundancy. On the other hand, the second tree depicts the prevalence of inverted repeats on each taxonomic branch. Red indicates branches with genomes with a high percentage of inverted repeats, whereas blue shows branches with a low percentage. For better visualization, visit the website https://asilab.github.io/canvas/.

vided.

Discussion

The usage of a specialized compressor is crucial to quantify the complexity present in a genome accurately. Specialized compressors outperform general-purpose compressors because they take into account the intrinsic nature of the data. Genomic data is highly heterogeneous and has high substitution mutations and data rearrangements, such as fusions, translocations, and inversions [68, 69]. Therefore, the ability of a genomic data compressor to adapt to this heterogeneous data, being able to perform an accurate structure modelling and detect repetitions in the presence of the high substitutional mutations and rearrangements in genomic data is fundamental to achieve high compressibility of the genome sequence. This article evaluates the capacity to identify data-specific patterns in genomic sequences by comparing the potential of tree methods to recognize IRs. Precisely, the NBDM was estimated, and the NC was computed using a genomic compressor (GeCo3 [14]) and a general-purpose data-compressor (PAQ8 [110, 111]). When GeCo3 had the subprogram activated that detects IRs (NC_{IR_2}), it showed substantially higher compression than general-purpose because PAQ uses models that do not consider these specific properties of the genomic se-

Table 1. Results obtained for viral taxonomic classification task regarding the genome type, realm, kingdom, phylum, class, order, family, and genus using XGBoost classifier. The features used were the genome's sequence length (SL), the GC-content (GC) and the Normalized Compression (NC) values for the best model, the same model with IR configuration to 0, to 1 and 2. The results correspond to the accuracy (ACC), and the probability of a random sequence being correctly classified (p_{hit}) using a random classifier ($p_{hit}(C_{Random})$.

Classification	N. Classes	N. Samples	$p_{hit}(C_{Random})$	ACC_{NC}	$ACC_{NC+SL+GC}$	$ACC_{AllFeatures}$
**Genome**	5	6089	20.00	75.48	87.06	**87.09**
**Realm**	5	5799	20.00	78.05	92.22	**92.41**
**Kingdom**	10	5788	10.00	76.22	90.57	**90.89**
**Phylum**	17	5778	5.88	64.18	82.42	**83.39**
**Class**	34	5845	2.94	60.03	79.01	**80.47**
**Order**	48	5838	2.08	58.35	78.02	**79.52**
**Family**	102	5990	0.98	43.38	72.86	**74.53**
**Genus**	360	4673	0.28	35.28	66.93	**68.42**

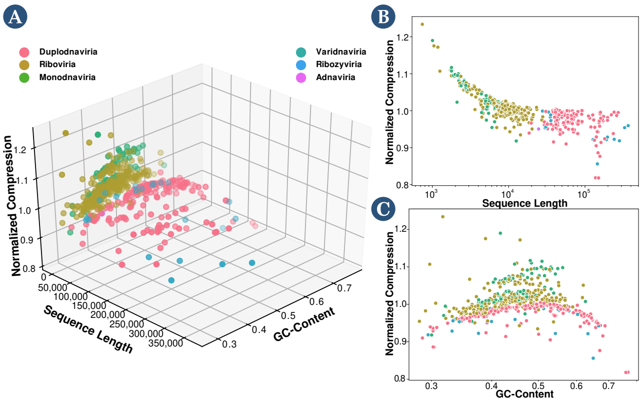

Figure 7. Scatter-plots of Normalized Compression vs. Sequence Length and GC-Content (**A**), Scatter-plots of Normalized Compression vs. Sequence Length (**B**) and Normalized Compression vs. GC-Content (**C**).

quences. The same occurs when comparing GeCo3 (NC_{IR_2}) with NBDM, showing that despite NBDM being able to detect small subprograms in synthetic data [85], it cannot detect IRs in genomic data. Moreover, GeCo3 compression capability was resistant to substitutional mutation up to 10%, showing that it can also deal with this extreme nature of genomic data, namely approximate IRs.

On average, RNA viruses mutate faster than DNA viruses, double-strand viruses mutate slower than single-stranded viruses, and genome size correlates negatively with mutation rate [112]. In this article, we have shown that the redundancy of dsDNA is higher than ssDNA, but for RNA viruses, the opposite occurs. The sequences used in this study to measure a lower NC (higher normalized redundancy) of the ssRNA to dsRNA have approximately the same length. However, the dataset of dsRNA has less than one order of magnitude in the number of sequences. This difference is natural since the ssRNA is much more abundant than dsRNA. Nevertheless, this discrepancy could justify the higher normalized redundancy of ssRNA in the first instance. However, although the lower average NC values of ssRNA are similar to dsRNA, the dsRNA has higher NC extremes. Therefore, we argue that this difference in the number of sequences in the dsRNA is not significant in changing the lower average of the ssRNA. Also, ssRNA are more prone to mutation than dsRNA [113]. On the other hand, extensive C to U mutations have been reported in many mammalian RNA viruses [114]. This behaviour was detected during a much faster evolution of the SARS-CoV- 2, an ssRNA virus [115]. Therefore, the faster average decrease of GC-content in ssRNA viruses explains a decrease in the ssRNA entropy and, hence, average NC. A higher GC-content (approximately 2%) of the dsRNA over ssRNA strengthens these outcomes (Table S3).

We performed an analysis of the human herpesvirus regarding their genome complexity and IRs abundance. Specifically, we analyzed the various behaviours of their subfamilies and identified that different complexities could be representative of the different rates of evolution within these genomes. Finally, we suggest that maybe a lower NC and abundance of inversions present in herpesvirus are associated with viral genome integration.

Lastly, we evaluated the capability of using complexity measures to perform viral classification at different taxonomic levels. Notably, results showed that we can automatically and accurately distinguish between viral genomes at different taxonomic levels using the XGBoost classifier with all features (NC with different configurations, GC-content and SL). However, a decrease in accuracy when approaching the lowest taxonomic levels was observed, which can be increased with future entries to the database. Finally, despite the high accuracy results obtained, further improvement of the results may be possible in the classification by adding the transcribed viral proteome information.

Conclusion

This manuscript shows that the efficient approximation of the Kolmogorov complexities of viral sequences as measures that quantify the absence of redundancy have a profound impact on genomes identification, classification, and organization.

For computing an upper bound of the sequence complexity, we benchmark a specific data compressor (GeCo3), after optimization, against other approaches, namely a high compression ratio general-purpose data compressor (PAQ) and a measure that combines small algorithmic programs and Shannon entropy (BDM). Unlike the other approaches, we show that GeCo3 can efficiently address and quantify regions properly described by simple algorithmic sources, namely exact and approximate inverted repeats, among other characteristics.

Using an optimized compression level of GeCo3 in an extensive viral dataset, we provide a comprehensive landscape of the viral genome's complexity, comparing the viral genomes at several taxonomic levels while identifying the genome regarding the lowest and highest proportion of complexity. Specifically, on average, dsDNA viruses are the most redundant (less complexity) according to their size, and ssDNA the less redundant. Contrarily, dsRNA shows a lower redundancy relative to ssRNA.

We perform an in-depth analysis of the human herpesvirus regarding their genome complexity and abundance of IRs. We induce that a lower NC and abundance of inversions present in herpesvirus may be associated with viral genome integration.

We describe and use minimal bi-directional complexity profiles of one sequence of each virus to visualize the distribution of complexity of these sequences locally. These profiles can describe actual regions detected in the genome with other meth-

ods, proving the description capability of data compression at a structural level.

We reveal the importance of efficient data compression in genome classification tasks, explicitly showing that the complexity, when combined with simple measures (GC-content and size), is efficient to accurately distinguish between viral genomes at different taxonomic levels without using direct comparisons between sequences.

The methods and results presented in this work provide new frontiers for studying viral genomes' complexity while magnifying the importance of developing efficient data compression methods for automatic and accurate viral analysis.

Availability of source code and requirements

- Project name: C.A.N.V.A.S. (Complexity ANalysis of VirAl Sequences)
- Project home page: https://github.com/jorgeMFS/canvas
- Operating system(s): Linux
- Programming language: Bash; Python.
- Other requirements: Python v3.6; Conda v4.3.27.
- License: e.g. MIT License.

To see the reproductions guidelines go to the Reproducibility section of the Supplementary Material.

Availability of supporting data and materials

Website

You can access a support website to this paper at https://asilab.github.io/canvas/. This site showcases, among other things, the pipeline of this study, the compressor's model selection, the detection of inverted repeats in synthetic genomic sequences, the viral genome characterization with regards to genome and type of taxonomic group, and the computed phylogenetic trees with a magnifier to allow a better observation of the normalized complexity results with illustrative examples of viruses.

Declarations

List of abbreviations

A → adenine
ANC → Average Normalized Compression
BDM → Block Decomposition Method
C → cytosine
CTM → Coding Theorem Method
dsDNA → double-stranded deoxyribonucleic acid
dsRNA → double-stranded ribonucleic acid
EBV→ Epstein-Barr virus
G → guanine
GC → GC-Content
GNB → Gaussian Naive Bayes
HCMV→ human cytomegalovirus
HHVs→ human herpesviruses
HSV-1 → herpes simplex virus 1
HSV-2 → herpes simplex virus 2
IR → inverted repeats
K→ Kolmogorov complexity
KNN → K-Nearest Neighbors
KSHV→ Kaposi's sarcoma-associated herpesvirus
LDA → Discriminant Analysis
mRNA → messenger ribonucleic acid
NBDM → Normalized Block Decomposition Method
NC → Normalized Compression
NCC → normalized compression capacity
NR → normalized redundancy
R→ redundancy
RdRp → RNA-dependent RNA polymerase
SL → Sequence Length
SNP→ Single Nucleotide Polymorphis
ssDNA → single-stranded deoxyribonucleic acid
ssRNA → single-stranded ribonucleic acid
SVM → Support Vector Machine
T → thymine
TM → Turing machines
U → uracil
VZV→ varicella-zoster virus
XGB → XGBoost

Competing Interests

The authors declare no competing interests.

Funding

This work was partially funded by National Funds through the FCT - Foundation for Science and Technology, in the context of the project UIDB/00127/2020. J.M.S. acknowledges the FCT grant SFRH/BD/141851/2018. D.P. is funded by national funds through FCT – Fundação para a Ciência e a Tecnologia, I.P., under the Scientific Employment Stimulus - Institutional Call - reference CEECINST/00026/2018. T.C. is funded by national funds (OE), through FCT – Fundação para a Ciência e a Tecnologia, I.P., in the scope of the framework contract foreseen in the numbers 4, 5 and 6 of the article 23, of the Decree-Law 57/2016, of August 29, changed by Law 57/2017, of July (CEECIND/01463/2017). Thanks are due to FCT/MCTES for the financial support to CESAM (UIDP/50017/2020+UIDB/50017/2020), through national funds.

Author's Contributions

J.M.S. and D.P. designed the experiment, executed data analysis and wrote the manuscript. All authors analysed and discussed the results and revised the manuscript.

References

1. O'Leary NA, Wright MW, Brister JR, Ciufo S, Haddad D, McVeigh R, et al. Reference sequence (RefSeq) database at NCBI: current status, taxonomic expansion, and functional annotation. Nucleic acids research 2016;44(D1):D733–D745.
2. Edwards RA, Rohwer F. Viral metagenomics. Nature Reviews Microbiology 2005;3(6):504–510.
3. Lawrence CM, Menon S, Eilers BJ, Bothner B, Khayat R, Douglas T, et al. Structural and functional studies of archaeal viruses. Journal of Biological Chemistry 2009;284(19):12599–12603.
4. Koonin EV, Senkevich TG, Dolja VV. The ancient Virus World and evolution of cells. Biology direct 2006;1(1):29.
5. Nayfach S, Roux S, Seshadri R, Udwary D, Varghese N, Schulz F, et al. A genomic catalog of Earth's microbiomes. Nature biotechnology 2021;39(4):499–509.
6. Fermin G. Virion Structure, Genome Organization, and Taxonomy of Viruses. Viruses 2018;p. 17.

7. Pratas D, Pinho AJ. On the approximation of the Kolmogorov complexity for DNA sequences. In: Iberian Conference on Pattern Recognition and Image Analysis Springer; 2017. p. 259–266.
8. Hosseini M, Pratas D, Morgenstern B, Pinho AJ. Smash++: an alignment-free and memory-efficient tool to find genomic rearrangements. GigaScience 2020;9(5):giaa048.
9. Cilibrasi R, Vitányi PM. Clustering by compression. IEEE Transactions on Information theory 2005;51(4):1523–1545.
10. Li M, Chen X, Li X, Ma B, Vitányi PM. The similarity metric. IEEE transactions on Information Theory 2004;50(12):3250–3264.
11. Bywater RP. Prediction of protein structural features from sequence data based on Shannon entropy and Kolmogorov complexity. PloS one 2015;10(4):e0119306.
12. Hayashida M, Akutsu T. Comparing biological networks via graph compression. In: BMC systems biology, vol. 4 BioMed Central; 2010. p. 1–11.
13. Pratas D, Pinho AJ. Metagenomic composition analysis of sedimentary ancient DNA from the Isle of Wight. In: 2018 26th European Signal Processing Conference (EUSIPCO) IEEE; 2018. p. 1177–1181.
14. Silva M, Pratas D, Pinho AJ. Efficient DNA sequence compression with neural networks. GigaScience 2020 11;9(11). Giaa119.
15. Editorial. Microbiology by numbers. Nature Reviews Microbiology 2011;9:628.
16. Strauss JH, Strauss EG. CHAPTER 1 - Overview of Viruses and Virus Infection. In: Strauss JH, Strauss EG, editors. Viruses and Human Disease (Second Edition), second edition ed. London: Academic Press; 2008.p. 1–33.
17. Lidmar J, Mirny L, Nelson DR. Virus shapes and buckling transitions in spherical shells. Physical Review E 2003;68(5):051910.
18. Vernizzi G, de la Cruz MO. Faceting ionic shells into icosahedra via electrostatics. Proceedings of the National Academy of Sciences 2007;104(47):18382–18386.
19. Luque A, Reguera D. The structure of elongated viral capsids. Biophysical journal 2010;98(12):2993–3003.
20. Tsagris EM, Martínez de Alba ÁE, Gozmanova M, Kalantidis K. Viroids. Cellular microbiology 2008;10(11):2168–2179.
21. Krupovic M, Cvirkaite-Krupovic V. Virophages or satellite viruses? Nature Reviews Microbiology 2011;9(11):762–763.
22. Dimmock NJ, Easton AJ, Leppard KN. Introduction to modern virology. John Wiley & Sons; 2016.
23. Simón D, Cristina J, Musto H. Nucleotide composition and codon usage across viruses and their respective hosts. Frontiers in Microbiology 2021;12.
24. Baltimore D. Expression of animal virus genomes. Bacteriological reviews 1971;35(3):235–241.
25. Peck KM, Lauring AS. Complexities of viral mutation rates. Journal of virology 2018;92(14):e01031–17.
26. Claverie JM, Ogata H, Audic S, Abergel C, Suhre K, Fournier PE. Mimivirus and the emerging concept of "giant" virus. Virus research 2006;117(1):133–144.
27. Claverie JM, Abergel C, Ogata H. Mimivirus. In: Lesser Known Large dsDNA Viruses Springer; 2009.p. 89–121.
28. Martin W, Koonin EV. Introns and the origin of nucleus–cytosol compartmentalization. Nature 2006;440(7080):41–45.
29. Cavalier-Smith T. Origin of the cell nucleus, mitosis and sex: roles of intracellular coevolution. Biology direct 2010;5(1):7.
30. Takemura M. Medusavirus Ancestor in a Proto-Eukaryotic Cell: Updating the Hypothesis for the Viral Origin of the Nucleus. Frontiers in Microbiology 2020;11:2169.
31. Toppinen M, Pratas D, Väisänen E, Söderlund-Venermo M, Hedman K, Perdomo MF, et al. The landscape of persistent human DNA viruses in femoral bone. Forensic Science International: Genetics 2020;48:102353.
32. Ikegaya H, Iwase H. Trial for the geographical identification using JC viral genotyping in Japan. Forensic science international 2004;139(2-3):169–172.
33. Agostini HT, Yanagihara R, Davis V, Ryschkewitsch CF, Stoner GL. Asian genotypes of JC virus in Native Americans and in a Pacific Island population: markers of viral evolution and human migration. Proceedings of the National Academy of Sciences 1997;94(26):14542–14546.
34. Sugimoto C, Kitamura T, Guo J, Al-Ahdal MN, Shchelkunov SN, Otova B, et al. Typing of urinary JC virus DNA offers a novel means of tracing human migrations. Proceedings of the National Academy of Sciences 1997;94(17):9191–9196.
35. Sugimoto C, Hasegawa M, Zheng HY, Demenev V, Sekino Y, Kojima K, et al. JC virus strains indigenous to northeastern Siberians and Canadian Inuits are unique but evolutionally related to those distributed throughout Europe and Mediterranean areas. Journal of Molecular Evolution 2002;55(3):322–335.
36. Forni D, Cagliani R, Clerici M, Pozzoli U, Sironi M. You will never walk alone: codispersal of JC polyomavirus with human populations. Molecular biology and evolution 2020;37(2):442–454.
37. Senior AW, Evans R, Jumper J, Kirkpatrick J, Sifre L, Green T, et al. Protein structure prediction using multiple deep neural networks in the 13th Critical Assessment of Protein Structure Prediction (CASP13). Proteins: Structure, Function, and Bioinformatics 2019;87(12):1141–1148.
38. Senior AW, Evans R, Jumper J, Kirkpatrick J, Sifre L, Green T, et al. Improved protein structure prediction using potentials from deep learning. Nature 2020;577(7792):706–710.
39. Hosseini M, Pratas D, Pinho AJ. On the role of inverted repeats in DNA sequence similarity. In: International Conference on Practical Applications of Computational Biology & Bioinformatics Springer; 2017. p. 228–236.
40. Toppinen M, et al. Parvoviral genomes in human soft tissues and bones over decades. PhD thesis; 2021.
41. Voineagu I, Narayanan V, Lobachev KS, Mirkin SM. Replication stalling at unstable inverted repeats: interplay between DNA hairpins and fork stabilizing proteins. Proceedings of the National Academy of Sciences 2008;105(29):9936–9941.
42. Bissler JJ. DNA inverted repeats and human disease. Front Biosci 1998;3(4):d408–d418.
43. Lin CT, Lin WH, Lyu YL, Whang-Peng J. Inverted repeats as genetic elements for promoting DNA inverted duplication: implications in gene amplification. Nucleic Acids Research 2001;29(17):3529–3538.
44. Atkins JF, Loughran G, Bhatt PR, Firth AE, Baranov PV. Ribosomal frameshifting and transcriptional slippage: From genetic steganography and cryptography to adventitious use. Nucleic acids research 2016;44(15):7007–7078.
45. Namy O, Moran SJ, Stuart DI, Gilbert RJ, Brierley I. A mechanical explanation of RNA pseudoknot function in programmed ribosomal frameshifting. Nature 2006;441(7090):244–247.
46. Mikl M, Pilpel Y, Segal E. High-throughput interrogation of programmed ribosomal frameshifting in human cells. Nature communications 2020;11(1):1–18.
47. Cotmore SF, Tattersall P. Parvoviruses: small does not mean simple. Annual review of virology 2014;1:517–537.
48. Yan Z, Zak R, Zhang Y, Engelhardt JF. Inverted termi-

nal repeat sequences are important for intermolecular recombination and circularization of adeno-associated virus genomes. Journal of virology 2005;79(1):364–379.
49. Byrne D, Grzela R, Lartigue A, Audic S, Chenivesse S, Encinas S, et al. The polyadenylation site of Mimivirus transcripts obeys a stringent 'hairpin rule'. Genome research 2009;19(7):1233–1242.
50. Claverie JM, Abergel C. Mimivirus and its virophage. Annual review of genetics 2009;43:49–66.
51. Solomonoff RJ. A formal theory of inductive inference. Part I. Information and control 1964;7(1):1–22.
52. Solomonoff RJ. A formal theory of inductive inference. Part II. Information and control 1964;7(2):224–254.
53. Kolmogorov AN. Three approaches to the quantitative definition of information'. Problems of information transmission 1965;1(1):1–7.
54. Chaitin GJ. On the length of programs for computing finite binary sequences. Journal of the ACM (JACM) 1966;13(4):547–569.
55. Hammer D, Romashchenko A, Shen A, Vereshchagin N. Inequalities for Shannon entropy and Kolmogorov complexity. Journal of Computer and System Sciences 2000;60(2):442–464.
56. Henriques T, Gonçalves H, Antunes L, Matias M, Bernardes J, Costa-Santos C. Entropy and compression: two measures of complexity. Journal of Evaluation in Clinical Practice 2013;19(6):1101–1106.
57. Soler-Toscano F, Zenil H, Delahaye JP, Gauvrit N. Calculating Kolmogorov complexity from the output frequency distributions of small Turing machines. PloS one 2014;9(5).
58. Zenil H, Hernández-Orozco S, Kiani NA, Soler-Toscano F, Rueda-Toicen A, Tegnér J. A decomposition method for global evaluation of Shannon entropy and local estimations of algorithmic complexity. Entropy 2018;20(8):605.
59. Zenil H, Soler-Toscano F, Dingle K, Louis AA. Correlation of automorphism group size and topological properties with program-size complexity evaluations of graphs and complex networks. Physica A: Statistical Mechanics and its Applications 2014;404:341–358.
60. Kempe V, Gauvrit N, Forsyth D. Structure emerges faster during cultural transmission in children than in adults. Cognition 2015;136:247–254.
61. Zenil H, Soler-Toscano F, Delahaye JP, Gauvrit N. Two-dimensional Kolmogorov complexity and an empirical validation of the Coding theorem method by compressibility. PeerJ Computer Science 2015;1:e23.
62. Bloem P, Mota F, de Rooij S, Antunes L, Adriaans P. A safe approximation for Kolmogorov complexity. In: International Conference on Algorithmic Learning Theory Springer; 2014. p. 336–350.
63. Dougherty ER, Shmulevich I. Genomic signal processing and statistics, vol. 2. Hindawi Publishing Corporation; 2005.
64. Gailly J, Adler M. The gzip home page; accessed May 16, 2020, http://www.gzip.org/.
65. bzip2; accessed May 16, 2020, http://www.bzip.org/.
66. Pavlov I. 7-Zip; accessed May 16, 2020, https://www.7-zip.org/.
67. Grumbach S, Tahi F. Compression of DNA sequences. In: [Proceedings] DCC93: Data Compression Conference IEEE; 1993. p. 340–350.
68. Rieseberg LH. Chromosomal rearrangements and speciation. Trends in ecology & evolution 2001;16(7):351–358.
69. Roeder GS, Fink GR. DNA rearrangements associated with a transposable element in yeast. Cell 1980;21(1):239–249.
70. Hernaez M, Pavlichin D, Weissman T, Ochoa I. Genomic data compression. Annual Review of Biomedical Data Science 2019;2:19–37.
71. Grumbach S, Tahi F. A new challenge for compression algorithms: genetic sequences. Information Processing & Management 1994;30(6):875–886.
72. Manzini G, Rastero M. A simple and fast DNA compressor. Software: Practice and Experience 2004;34(14):1397–1411.
73. Cherniavsky N, Ladner R. Grammar-based compression of DNA sequences. DIMACS Working Group on The Burrows-Wheeler Transform 2004;21.
74. Korodi G, Tabus I. An efficient normalized maximum likelihood algorithm for DNA sequence compression. ACM Transactions on Information Systems (TOIS) 2005;23(1):3–34.
75. Vey G. Differential direct coding: a compression algorithm for nucleotide sequence data. Database 2009;2009.
76. Mishra KN, Aaggarwal A, Abdelhadi E, Srivastava D. An efficient horizontal and vertical method for online DNA sequence compression. International Journal of Computer Applications 2010;3(1):39–46.
77. Rajeswari PR, Apparao A. GENBIT Compress-Algorithm for repetitive and non repetitive DNA sequences. International Journal of Computer Science and Information Technology 2010;2:25–29.
78. Gupta A, Agarwal S. A novel approach for compressing DNA sequences using semi-statistical compressor. International Journal of Computers and Applications 2011;33(3):245–251.
79. Zhu Z, Zhou J, Ji Z, Shi YH. DNA sequence compression using adaptive particle swarm optimization-based memetic algorithm. IEEE Transactions on Evolutionary Computation 2011;15(5):643–658.
80. Pinho AJ, Ferreira PJ, Neves AJ, Bastos CA. On the representability of complete genomes by multiple competing finite-context (Markov) models. PloS one 2011;6(6):e21588.
81. Pratas D, Pinho AJ, Ferreira PJ. Efficient compression of genomic sequences. In: 2016 Data Compression Conference (DCC) IEEE; 2016. p. 231–240.
82. Cao MD, Dix TI, Allison L, Mears C. A simple statistical algorithm for biological sequence compression. In: 2007 Data Compression Conference (DCC'07) IEEE; 2007. p. 43–52.
83. Pratas D, Hosseini M, Silva JM, Pinho AJ. A reference-free lossless compression algorithm for DNA sequences using a competitive prediction of two classes of weighted models. Entropy 2019;21(11):1074.
84. Pratas D, Hosseini M, Pinho AJ. GeCo2: An optimized tool for lossless compression and analysis of DNA sequences. In: International Conference on Practical Applications of Computational Biology & Bioinformatics Springer; 2019. p. 137–145.
85. Silva JM, Pratas D, Antunes R, Matos S, Pinho AJ. Automatic analysis of artistic paintings using information-based measures. Pattern Recognition 2021;114:107864.
86. Pinho AJ, Garcia SP, Pratas D, Ferreira PJ. DNA sequences at a glance. PloS one 2013;8(11):e79922.
87. Almeida JR, Pinho AJ, Oliveira JL, Fajarda O, Pratas D. GTO: a toolkit to unify pipelines in genomic and proteomic research. SoftwareX 2020;12:100535.
88. Romiguier J, Ranwez V, Douzery EJ, Galtier N. Contrasting GC-content dynamics across 33 mammalian genomes: relationship with life-history traits and chromosome sizes. Genome research 2010;20(8):1001–1009.
89. Duret L, Galtier N. Biased gene conversion and the evolution of mammalian genomic landscapes. Annual review of genomics and human genetics 2009;10:285–311.
90. Chen H, Skylaris CK. Analysis of DNA interactions and GC content with energy decomposition in large-scale quan-

tum mechanical calculations. Physical Chemistry Chemical Physics 2021;23(14):8891–8899.
91. Kans J. Entrez direct: E-utilities on the UNIX command line. National Center for Biotechnology Information (US); 2020.
92. McLachlan GJ. Discriminant analysis and statistical pattern recognition, vol. 544. John Wiley & Sons; 2004.
93. Rish I, et al. An empirical study of the naive Bayes classifier. In: IJCAI 2001 workshop on empirical methods in artificial intelligence, vol. 3; 2001. p. 41–46.
94. Guo G, Wang H, Bell D, Bi Y, Greer K. KNN Model-Based Approach in Classification. Springer Berlin Heidelberg 2003;p. 986–996.
95. Cristianini N, Shawe-Taylor J, et al. An introduction to support vector machines and other kernel-based learning methods. Cambridge university press; 2000.
96. Chen T, Guestrin C. XGBoost: A Scalable Tree Boosting System. In: Proceedings of the 22nd ACM SIGKDD International Conference on Knowledge Discovery and Data Mining KDD '16, New York, NY, USA: ACM; 2016. p. 785–794.
97. Mahoney M, Data compression programs. Overview over PAQ based compression software. http://mattmahoney.net/dc . . . ; 2009.
98. Prangishvili D, Rensen E, Mochizuki T, Krupovic M, et al. ICTV virus taxonomy profile: Tristromaviridae. Journal of General Virology 2019;100(2):135–136.
99. Krupovic M, Kuhn JH, Wang F, Baquero DP, Dolja VV, Egelman EH, et al. Adnaviria: a new realm for archaeal filamentous viruses with linear A-form double-stranded DNA genomes. Journal of Virology 2021;p. JVI–00673.
100. Krupovic M, Cvirkaite-Krupovic V, Iranzo J, Prangishvili D, Koonin EV. Viruses of archaea: structural, functional, environmental and evolutionary genomics. Virus research 2018;244:181–193.
101. Ayllón MA, Turina M, Xie J, Nerva L, Marzano SYL, Donaire L, et al. ICTV virus taxonomy profile: Botourmiaviridae. The Journal of general virology 2020;101(5):454.
102. Savin KW, Cocks BG, Wong F, Sawbridge T, Cogan N, Savage D, et al. A neurotropic herpesvirus infecting the gastropod, abalone, shares ancestry with oyster herpesvirus and a herpesvirus associated with the amphioxus genome. Virology journal 2010;7(1):1–9.
103. King AM, Lefkowitz E, Adams MJ, Carstens EB. Virus taxonomy: ninth report of the International Committee on Taxonomy of Viruses, vol. 9. Elsevier; 2011.
104. Pyöriä L, Jokinen M, Toppinen M, Salminen H, Vuorinen T, Hukkanen V, et al. HERQ-9 is a new multiplex PCR for differentiation and quantification of all nine human herpesviruses. Msphere 2020;5(3):e00265–20.
105. Baines JD, Pellett PE. Genetic comparison of human alphaherpesvirus genomes. Human herpesviruses: biology, therapy, and immunoprophylaxis 2007;.
106. Liu X, Kosugi S, Koide R, Kawamura Y, Ito J, Miura H, et al. Endogenization and excision of human herpesvirus 6 in human genomes. PLoS Genetics 2020;16(8):e1008915.
107. Rajaby R, Zhou Y, Meng Y, Zeng X, Li G, Wu P, et al. SurVirus: a repeat-aware virus integration caller. Nucleic acids research 2021;49(6):e33–e33.
108. Aimola G, Beythien G, Aswad A, Kaufer BB. Current understanding of human herpesvirus 6 (HHV-6) chromosomal integration. Antiviral research 2020;176:104720.
109. Lu J, Salzberg SL. Removing contaminants from databases of draft genomes. PLoS computational biology 2018;14(6):e1006277.
110. Knoll B, de Freitas N. A machine learning perspective on predictive coding with PAQ8. In: 2012 Data Compression Conference IEEE; 2012. p. 377–386.
111. Buchner AJ. PAQ; accessed May 16, 2020, https://github.com/JohannesBuchner/paq/.
112. Sanjuán R, Domingo-Calap P. Mechanisms of viral mutation. Cellular and molecular life sciences 2016;73(23):4433–4448.
113. Mahy BW. The evolution and emergence of RNA viruses. Emerging infectious diseases 2010;16(5):899.
114. Simmonds P, Ansari MA. Extensive C-> U transition biases in the genomes of a wide range of mammalian RNA viruses; potential associations with transcriptional mutations, damage-or host-mediated editing of viral RNA. PLoS pathogens 2021;17(6):e1009596.
115. Simmonds P. Rampant C→ U hypermutation in the genomes of SARS-CoV-2 and other coronaviruses: causes and consequences for their short-and long-term evolutionary trajectories. Msphere 2020;5(3):e00408–20.

Supplementary Material

Here, we depict the supplementary material of the article. The supplementary material is described in four main sections: Compression Model Benchmark, Viral Genome Analysis, Classification, Software and Hardware recommendations, and Reproducibility. The Compression Model Benchmark, Viral Genome Analysis and Classification sections have auxiliary material to their corresponding sections of the main article. On the other hand, the Software and Hardware recommendations section defines minimum requirements, and the Reproducibility section describes how to reproduce the results obtained in this article.

Compression Level Benchmark

Herein, it is depicted the supplementary material to the Compression Levels Benchmark of the methods section. Table S1 describes the parameters used in the six costume build levels. The flag *"tm"* is the template of a target context model, the flag *"lr"* defines the learning rate, and the flag *"hs"* defines the number of hidden nodes for the neural network.

Table S1. Depiction of the parameters used in the six costume levels.

Level	Values
1	-tm 1:1:0:0:0.7/0:0:0 -tm 12:20:1:1:0.97/1:1:0.97
2	-tm 1:1:0:0:0.7/0:0:0 -tm 12:20:1:1:0.97/2:1:0.97
3	-tm 1:1:0:0:0.7/0:0:0 -tm 12:50:1:1:0.97/0:0:0.97
4	-tm 1:1:0:0:0.7/0:0:0 -tm 12:20:1:1:0.97/0:0:0.97 -lr 0.05 -hs 40
5	-tm 1:1:0:0:0.7/0:0:0 -tm 12:20:1:1:0.97/0:0:0.97 -lr 0.15 -hs 40
6	-tm 1:1:0:0:0.7/0:0:0 -tm 12:20:1:1:0.97/0:0:0.97 -lr 0.3 -hs 40

Table S2 describes the parameters used in the template of a target context model. The template has the flag *"tm"* and follows the model "[NB_C]:[NB_D]:[NB_I]:[NB_H]:[NB_G]/[NB_S]:[NB_E]:[NB_A]".

Table S2. Depiction of the parameters used in the template of a target context model.

Parameter	Values	Description
**[NB_C]**	integer [1;20]	Order size of the regular context model. The higher the value of the regular context model, the more RAM it uses but, usually, are related to a better compression score.
**[NB_D]**	integer [1;5000]	Denominator to build alpha, which is a parameter estimator. Alpha is given by 1/[NB_D]. Higher values are usually used with higher [NB_C] and are related to sure bets. When [NB_D] is one, the probabilities assume a Laplacian distribution.
**[NB_I]**	integer {0,1,2}	Number to define if a sub-program that addresses the specific properties of DNA sequences (inverted repeats) is used or not. The number 2 turns ON this sub-program without the regular context model (only inverted repeats). The number 1 turns ON the sub-program using at the same time the regular context model. The number 0 does not contemplate its use (inverted repeats OFF). This sub-program increases the necessary time to compress, but it does not affect the RAM.
**[NB_H]**	integer [1;254]	Size of the cache-hash for deeper context models, namely for [NB_C] >14. When the [NB_C] <= 14 use, for example, 1 as a default. The RAM is highly dependent of this value (higher value stand for higher RAM).
**[NB_G]**	real [0;1)	Real number to define gamma. This value represents the decaying forgetting factor of the regular context model in the definition.
**[NB_S]**	integer [0;20]	The maximum number of editions allowed to use a substitutional tolerant model with the same memory model of the regular context model with an order size equal to [NB_C]. The value 0 stands for turning the tolerant context model off. When the model is on, it pauses when the number of editions is higher than [NB_C]. When it is turned on when a full match of size [NB_C] is seen again, this is a probabilistic-algorithmic model advantageous to handle the high substitutional nature of genomic sequences. When [NB_S] >0, the compressor used more processing time but used the same RAM and, usually, achieved a substantial higher compression ratio. The impact of this model is usually only noticed for [NB_C] >= 14.
**[NB_E]**	integer [1;5000]	Denominator to build alpha for substitutional tolerant context model. It is analogous to [NB_D]. However, it is only used in the probabilistic model for computing the statistics of the substitutional tolerant context model.
**[NB_A]**	real [0;1)	Real number to define gamma. This value represents the decaying forgetting factor of the substitutional tolerant context model in the definition. Its definition and use are analogous to [NB_G].

Viral Genome Analysis

We present the supplementary material discussed in the Viral Genome Analysis of this main article. Table S3 depicts the genome types ordered by the highest normalized compression (NC), normalized compression capacity (*NCC*) and *difference*. *NCC* is computed by $NCC = 1 - NC_{IR_2} > 0$, and the difference as $difference = NC_{IR_0} - NC_{IR_1}$. Furthermore, the Table shows the genomes' average Sequence Length (SL) and GC-Content (GC).

Table S4 depicts the top Normalized Compression (NC) values by taxonomic group. Three main groups separate the Table. The first represents the highest 10 NC values using standard settings NC (best performing model); the second group shows the top 10 lowest NC values obtained using the IR_2 subprogram. Finally, the third group shows the top 10 highest values of the difference between NC using IR_0 and IR_1 subprograms.

Tables S5,S6,and S7 organize the top taxa (by taxonomic group) regarding their normalized compression (NC), normalized compression capacity (*NCC*) and *difference*. The tables also shows the genomes' average Sequence Length and GC-Content.

Finally, Figure S1 depicts the phylogenetic tree with average NC *difference* ($NC_{IR_0} - NC_{IR_1} > 0$) for each viral taxonomic group up to the viral genus. The colour red depicting the highest NC *difference*, and the blue the lowest.

Table S3. Depiction of the genome type by the highest normalized compression (NC), normalized compression capacity (*NCC*) and *difference*. *NCC* is computed by $NCC = 1 - NC_{IR_2} > 0$, and the difference as $difference = NC_{IR_0} - NC_{IR_1}$. Furthermore, the Table shows the genomes' average Sequence Length (SL) and GC-Content (GC).

**Normalized Compression**				**Inverted Repeats**				**Difference**			
**Genome**	**NC**	**SL**	**GC**	**Genome**	**NCC**	**SL**	**GC**	**Genome**	**difference**	**SL**	**GC**
**ssDNA**	1.065	3282	0.447	**dsDNA**	0.029	84721	0.485	**ssDNA**	0.006	4672	0.435
**mixedDNA**	1.050	3258	0.491	**ssDNA**	0.026	5981	0.389	**dsDNA**	0.006	80636	0.470
**dsRNA**	1.047	8377	0.456	**ssRNA**	0.015	13425	0.393	**mixedDNA**	0.002	3311	0.434
**ssRNA**	1.013	9564	0.437	**dsRNA**	0.015	19911	0.396	**dsRNA**	0.001	6186	0.431
**dsDNA**	0.977	70353	0.481					**ssRNA**	0.001	10197	0.433

Table S4. Depiction of the top NC values by taxonomic group. Three main groups separate the Table. The first represents the highest 10 NC values using standard settings NC (best performing model); the second group shows the top 10 lowest NC values obtained using the IR_2 subprogram. Finally, the third group shows the top 10 highest values of the difference between NC using IR_0 and IR_1 subprograms.

NC	Top	Realm	Kingdom	Phylum	Class	Order	Family	Genus
Best performing model	1	Ribozyviria	Shotokuvirae	Lenarviricota	Miaviricetes	Ourlivirales	Botourmiaviridae	Clostunsatellite
	2	Monodnaviria	Sangervirae	Cressdnaviricota	Arfiviricetes	Cirlivirales	Alphasatellitidae	Milvetsatellite
	3	Riboviria	Orthornavirae	Duplornaviricota	Chunqiuviricetes	Cremevirales	Tolecusatellitidae	Aumaivirus
	4	Duplodnaviria	Pararnavirae	Phixviricota	Magsaviricetes	Muvirales	Circoviridae	Virtovirus
	5	Varidnaviria	Loebvirae	Kitrinoviricota	Amabiliviricetes	Nodamuvirales	Genomoviridae	Mivedwarsatellite
	6	Adnaviria	Trapavirae	Cossaviricota	Duplopiviricetes	Wolframvirales	Nodaviridae	Babusatellite
	7	-	Heunggongvirae	Pisuviricota	Allassoviricetes	Durnavirales	Kolmioviridae	Fabenesatellite
	8	-	Bamfordvirae	Negarnaviricota	Repensiviricetes	Levivirales	Smacoviridae	Ourmiavirus
	9	-	Helvetiavirae	Artverviricota	Yunchangviricetes	Geplafuvirales	Qinviridae	Albetovirus
	10	-	Zilligvirae	Hofneiviricota	Insthoviricetes	Goujianvirales	Narnaviridae	Geminialphasatellitinae
Inverted Repeat Subprogram (NC_{IR_2})	1	Adnaviria	Loebvirae	Peploviricota	Pokkesviricetes	Imitervirales	Mimiviridae	Betaentomopoxvirus
	2	Varidnaviria	Zilligvirae	Nucleocytoviricota	Herviviricetes	Chitovirales	Rudiviridae	Oryzopoxvirus
	3	Duplodnaviria	Helvetiavirae	Hofneiviricota	Maveriviricetes	Herpesvirales	Poxviridae	Vespertilionpoxvirus
	4	Monodnaviria	Bamfordvirae	Taleaviricota	Mouviricetes	Priklausovirales	Malacoherpesviridae	Simplexvirus
	5	Riboviria	Heunggongvirae	Dividoviricota	Faserviricetes	Polivirales	Plectroviridae	Cafeteriavirus
	6	Ribozyviria	Trapavirae	Uroviricota	Tokiviricetes	Ligamenvirales	Mononiviridae	Mardivirus
	7	-	Shotokuvirae	Saleviricota	Laserviricetes	Tubulavirales	Herpesviridae	Cervidpoxvirus
	8	-	Pararnavirae	Preplasmiviricota	Megaviricetes	Halopanivirales	Lavidaviridae	Varicellovirus
	9	-	Orthornavirae	Negarnaviricota	Naldaviricetes	Pimascovirales	Bidnaviridae	Ostreavirus
	10	-	Sangervirae	Cossaviricota	Milneviricetes	Lefavirales	Polydnaviridae	Vespertiliovirus
$NC_{IR_0} - NC_{IR_1}$	1	Adnaviria	Zilligvirae	Peploviricota	Herviviricetes	Herpesvirales	Malacoherpesviridae	Mardivirus
	2	Varidnaviria	Trapavirae	Taleaviricota	Mouviricetes	Polivirales	Herpesviridae	Ostreavirus
	3	Duplodnaviria	Bamfordvirae	Nucleocytoviricota	Tokiviricetes	Chitovirales	Rudiviridae	Iltovirus
	4	Monodnaviria	Heunggongvirae	Saleviricota	Pokkesviricetes	Ligamenvirales	Bidnaviridae	Leporipoxvirus
	5	Ribozyviria	Shotokuvirae	Cossaviricota	Quintoviricetes	Piccovirales	Poxviridae	Simplexvirus
	6	Riboviria	Helvetiavirae	Dividoviricota	Huolimaviricetes	Haloruvirales	Polydnaviridae	Varicellovirus
	7	-	Loebvirae	Hofneiviricota	Megaviricetes	Cirlivirales	Ampullaviridae	Aurivirus
	8	-	Sangervirae	Cressdnaviricota	Laserviricetes	Pimascovirales	Nudiviridae	Oryzopoxvirus
	9	-	Orthornavirae	Preplasmiviricota	Arfiviricetes	Algavirales	Parvoviridae	Vespertilionpoxvirus
	10	-	Pararnavirae	Duplornaviricota	Faserviricetes	Kalamavirales	Ascoviridae	Entnonagintavirus

Table S5. Depiction of the taxonomic groups with the highest NC values. The Table shows each group's average Normalized Compression, Sequence Length and GC-Content.

Taxonomic Group	Taxonomic Name	Normalized Compression	Sequence Length	GC-Content
**Super-Realm**	Viruses	1.007	36067	0.460
**Realm**	Ribozyviria	1.080	1682	0.588
	Monodnaviria	1.046	4380	0.450
	Riboviria	1.016	9332	0.438
	Duplodnaviria	0.972	78102	0.500
	Varidnaviria	0.957	109560	0.448
	Adnaviria	0.948	33068	0.353
**Kingdom**	Shotokuvirae	1.049	4200	0.447
	Sangervirae	1.026	5518	0.435
	Orthornavirae	1.018	9472	0.438
	Pararnavirae	0.995	7787	0.433
	Loebvirae	0.994	7332	0.483
	Trapavirae	0.993	10151	0.564
	Heunggongvirae	0.972	78102	0.500
	Bamfordvirae	0.957	112955	0.441
	Helvetiavirae	0.949	24833	0.665
	Zilligvirae	0.948	33068	0.353
**Phylum**	Lenarviricota	1.094	2654	0.476
	Cressdnaviricota	1.067	3134	0.453
	Duplornaviricota	1.045	9418	0.456
	Phixviricota	1.026	5518	0.435
	Kitrinoviricota	1.018	8548	0.474
	Cossaviricota	1.013	6260	0.436
	Pisuviricota	1.012	10580	0.442
	Negarnaviricota	1.012	9620	0.397
	Artverviricota	0.995	7787	0.433
	Hofneiviricota	0.994	7332	0.483
**Class**	Miaviricetes	1.151	1792	0.514
	Arfiviricetes	1.085	2557	0.464
	Chunqiuviricetes	1.075	3870	0.503
	Magsaviricetes	1.073	3730	0.513
	Amabiliviricetes	1.072	2703	0.586
	Duplopiviricetes	1.066	3298	0.467
	Allassoviricetes	1.063	3753	0.493
	Repensiviricetes	1.063	3281	0.451
	Yunchangviricetes	1.061	3987	0.358
	Insthoviricetes	1.054	5784	0.425
**Order**	Ourlivirales	1.151	1792	0.514
	Cirlivirales	1.103	1864	0.471
	Cremevirales	1.078	2572	0.478
	Muvirales	1.075	3870	0.503
	Nodamuvirales	1.073	3730	0.513
	Wolframvirales	1.072	2703	0.586
	Durnavirales	1.066	3298	0.467
	Levivirales	1.063	3753	0.493
	Geplafuvirales	1.063	3281	0.451
	Goujianvirales	1.061	3987	0.358
**Family**	Botourmiaviridae	1.151	1792	0.514
	Alphasatellitidae	1.143	1296	0.418
	Tolecusatellitidae	1.116	1347	0.389
	Circoviridae	1.103	1864	0.471
	Genomoviridae	1.096	2201	0.517
	Nodaviridae	1.080	3368	0.514
	Kolmioviridae	1.080	1682	0.588
	Smacoviridae	1.078	2572	0.478
	Qinviridae	1.075	3870	0.503
	Narnaviridae	1.072	2703	0.586
**Genus**	Clostunsatellite	1.192	1008	0.423
	Milvetsatellite	1.186	1022	0.402
	Aumaivirus	1.185	1168	0.510
	Virtovirus	1.180	1150	0.442
	Mivedwarsatellite	1.179	1014	0.402
	Babusatellite	1.178	1104	0.437
	Fabenesatellite	1.176	1007	0.385
	Ourmiavirus	1.167	1605	0.519
	Albetovirus	1.167	1221	0.426
	Geminialphasatellitinae	1.131	1370	0.418

Table S6. Depiction of the taxonomic groups with the highest normalized compression capacity (*NCC*) using only the inverted repeats subprogram IR_2. The top results were obtained by $NCC = 1 - NC_{IR_2} > 0$. Besides the normalized compression capacity, the Table shows each group's average Sequence Length and GC-Content.

Group	Taxonomic Group	$NCC = 1 - NC_{IR_2} > 0$	Sequence Legth	GC-Content
**Super-Realm**	Viruses	0.026	66796	0.462
**Realm**	Adnaviria	0.052	33068	0.353
	Varidnaviria	0.038	110591	0.447
	Duplodnaviria	0.028	82677	0.499
	Monodnaviria	0.022	6958	0.399
	Riboviria	0.015	13682	0.391
**Kingdom**	Loebvirae	0.053	7371	0.385
	Zilligvirae	0.052	33068	0.353
	Helvetiavirae	0.050	24833	0.665
	Bamfordvirae	0.038	114079	0.440
	Heunggongvirae	0.028	82677	0.499
	Trapavirae	0.021	12225	0.577
	Shotokuvirae	0.016	6184	0.378
	Pararnavirae	0.016	9610	0.378
	Orthornavirae	0.015	14012	0.393
	Sangervirae	0.005	4421	0.321
**Phylum**	Peploviricota	0.068	168832	0.534
	Nucleocytoviricota	0.063	210417	0.389
	Hofneiviricota	0.053	7371	0.385
	Taleaviricota	0.052	33068	0.353
	Dividoviricota	0.050	24833	0.665
	Uroviricota	0.026	79042	0.497
	Saleviricota	0.021	12225	0.577
	Preplasmiviricota	0.017	32147	0.483
	Negarnaviricota	0.016	12180	0.376
	Cossaviricota	0.016	6128	0.378
**Class**	Pokkesviricetes	0.072	190762	0.365
	Herviviricetes	0.068	168832	0.534
	Maveriviricetes	0.066	18227	0.290
	Mouviricetes	0.066	8377	0.299
	Faserviricetes	0.053	7371	0.385
	Tokiviricetes	0.052	33068	0.353
	Laserviricetes	0.050	24833	0.665
	Megaviricetes	0.046	248459	0.436
	Naldaviricetes	0.040	132022	0.410
	Milneviricetes	0.029	11079	0.349
**Order**	Imitervirales	0.109	899501	0.256
	Chitovirales	0.091	193551	0.356
	Herpesvirales	0.068	168832	0.534
	Priklausovirales	0.066	18227	0.290
	Polivirales	0.066	8377	0.299
	Ligamenvirales	0.055	34464	0.343
	Tubulavirales	0.053	7371	0.385
	Halopanivirales	0.050	24833	0.665
	Pimascovirales	0.043	162587	0.456
	Lefavirales	0.040	132022	0.410
**Family**	Mimiviridae	0.109	899501	0.256
	Rudiviridae	0.103	30804	0.299
	Poxviridae	0.091	193551	0.356
	Malacoherpesviridae	0.091	209479	0.427
	Plectroviridae	0.080	7045	0.248
	Mononiviridae	0.077	41178	0.275
	Herpesviridae	0.074	158421	0.539
	Lavidaviridae	0.066	18227	0.290
	Bidnaviridae	0.066	8377	0.299
	Polydnaviridae	0.055	306235	0.377
**Genus**	Betaentomopoxvirus	0.174	247441	0.195
	Oryzopoxvirus	0.164	185139	0.236
	Vespertilionpoxvirus	0.156	176688	0.236
	Simplexvirus	0.144	148626	0.694
	Cafeteriavirus	0.127	617453	0.233
	Mardivirus	0.121	177993	0.509
	Cervidpoxvirus	0.115	166259	0.262
	Varicellovirus	0.107	139331	0.560
	Ostreavirus	0.107	207439	0.387
	Vespertiliovirus	0.103	7970	0.228

Classification

Herein, we show the supplementary classification tables that are discussed in the classification subsection of this article.

Figure S2 represents the number of samples (genome sequences) per viral genus.

Table S8 and Table S9 show the values obtained using different classifiers for accuracy and F1-score, respectively. In both cases, the XGBoost classifier had the best performance.

Table S7. Depiction of the taxonomic groups with the highest difference of values between $NC_{IR_0} - NC_{IR_1}$. The Table shows each group's average *difference* = $NC_{IR_0} - NC_{IR_1}$, Sequence Length and GC-Content.

Taxonomic Group	Taxonomic Name	$NC_{IR_0} - NC_{IR_1} > 0$	Sequece Length	GC-Content
**Super-Realm**	Viruses	0.004	44293	0.451
**Realm**	Adnaviria	0.019	35299	0.322
	Varidnaviria	0.007	111364	0.443
	Duplodnaviria	0.007	78316	0.512
	Monodnaviria	0.005	5359	0.436
	Ribozyviria	0.002	1682	0.588
	Riboviria	0.001	9847	0.431
**Kingdom**	Zilligvirae	0.019	35299	0.322
	Trapavirae	0.009	16113	0.503
	Bamfordvirae	0.007	114249	0.437
	Heunggongvirae	0.007	78316	0.512
	Shotokuvirae	0.005	5124	0.434
	Helvetiavirae	0.004	27439	0.664
	Loebvirae	0.002	8519	0.453
	Sangervirae	0.001	4552	0.426
	Orthornavirae	0.001	10049	0.430
	Pararnavirae	0.001	8050	0.435
**Phylum**	Peploviricota	0.050	159507	0.557
	Taleaviricota	0.019	35299	0.322
	Nucleocytoviricota	0.013	210797	0.381
	Saleviricota	0.009	16113	0.503
	Cossaviricota	0.007	5450	0.433
	Dividoviricota	0.004	27439	0.664
	Hofneiviricota	0.002	8519	0.453
	Cressdnaviricota	0.002	4539	0.438
	Preplasmiviricota	0.002	32788	0.483
	Duplornaviricota	0.001	8140	0.389
**Class**	Herviviricetes	0.050	159507	0.557
	Mouviricetes	0.029	8377	0.299
	Tokiviricetes	0.019	35299	0.322
	Pokkesviricetes	0.017	193309	0.354
	Quintoviricetes	0.011	5164	0.446
	Huolimaviricetes	0.009	16113	0.503
	Megaviricetes	0.005	247791	0.441
	Laserviricetes	0.004	27439	0.664
	Arfiviricetes	0.004	5459	0.432
	Faserviricetes	0.002	8519	0.453
**Order**	Herpesvirales	0.050	159507	0.557
	Polivirales	0.029	8377	0.299
	Chitovirales	0.022	196072	0.341
	Ligamenvirales	0.019	35299	0.322
	Piccovirales	0.011	5164	0.446
	Haloruvirales	0.009	16113	0.503
	Cirlivirales	0.008	2114	0.476
	Pimascovirales	0.005	169619	0.458
	Algavirales	0.005	339710	0.413
	Kalamavirales	0.004	15181	0.459
**Family**	Malacoherpesviridae	0.062	209479	0.427
	Herpesviridae	0.050	155406	0.564
	Rudiviridae	0.035	30804	0.299
	Bidnaviridae	0.029	8377	0.299
	Poxviridae	0.022	196072	0.341
	Polydnaviridae	0.019	306235	0.377
	Ampullaviridae	0.012	23814	0.346
	Nudiviridae	0.012	127615	0.416
	Parvoviridae	0.011	5164	0.446
	Ascoviridae	0.010	172411	0.453
**Genus**	Mardivirus	0.103	177993	0.509
	Ostreavirus	0.072	207439	0.387
	Iltovirus	0.070	155856	0.546
	Leporipoxvirus	0.066	160815	0.415
	Simplexvirus	0.061	148626	0.694
	Varicellovirus	0.061	139331	0.560
	Aurivirus	0.052	211518	0.468
	Oryzopoxvirus	0.050	185139	0.236
	Vespertilionpoxvirus	0.046	176688	0.236
	Entnonagintavirus	0.036	29564	0.558

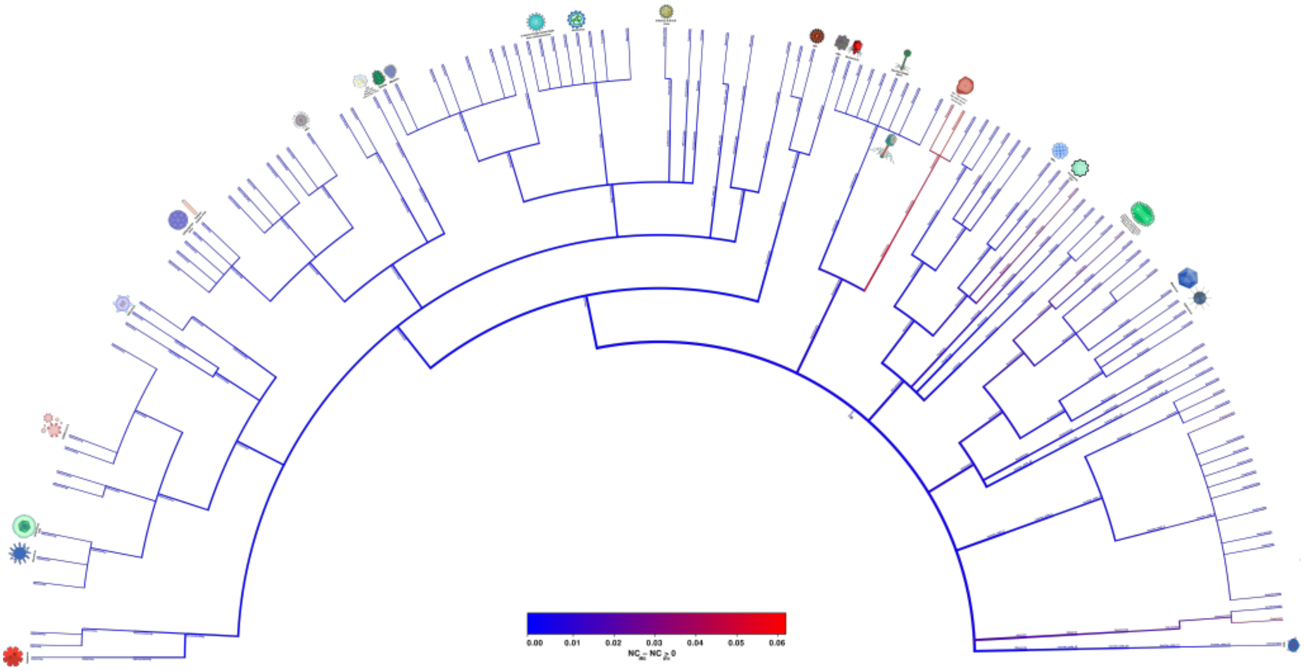

Figure S1. Phylogenetic tree showing average *difference* ($NC_{IR_0} - NC_{IR_1} > 0$).The colour red depicts the branches where on average, the genome possesses more inverted repetitions than internal repetitions (higher difference), whereas the blue colour represents the branches with fewer inverted repetitions than internal repetitions (smaller difference).

Table S10 displays the XGBoost classifier F1-score results when using different sets of features. With the notable exception of the type of genome classification, the best results were obtained using all features.

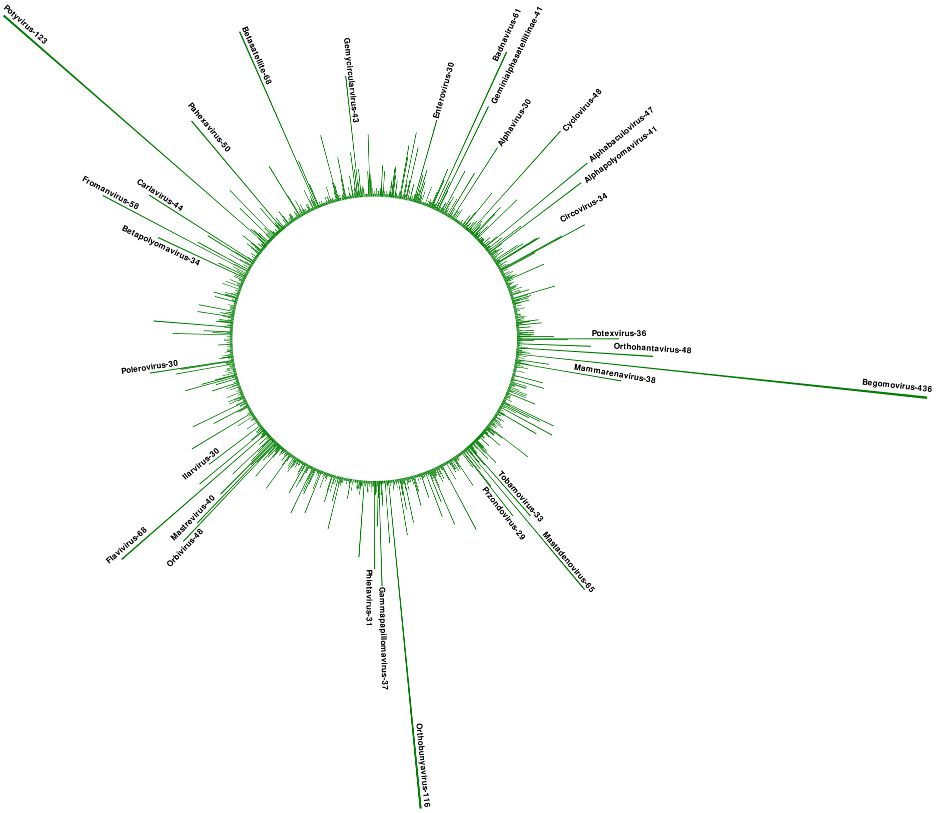

Figure S2. Frequency of genome sequences per viral genus.

Table S8. Accuracy (ACC) results obtained for viral taxonomic classification task regarding genome type, realm, kingdom, phylum, class, order, family, and genus. The classifiers used were Linear Discriminant Analysis (LDA), Gaussian Naive Bayes (GNB), K-Nearest Neighbors (KNN), Support Vector Machine (SVM) and XGBoost classifier (XGB).

Classification	N. Classes	N. Samples	ACC_{LDA}	ACC_{GNB}	ACC_{SVM}	ACC_{KNN}	ACC_{XGB}
**Genome**	5	6089	66.17	73.32	72.58	84.24	**87.09**
**Realm**	5	5799	76.38	80.69	80.34	89.31	**92.41**
**Kingdom**	10	5788	72.97	78.76	78.67	86.01	**90.89**
**Phylum**	17	5778	60.90	57.44	57.70	70.16	**83.39**
**Class**	34	5845	50.98	52.52	49.36	64.24	**80.47**
**Order**	48	5838	49.32	55.48	48.54	60.53	**79.52**
**Family**	102	5990	37.15	43.49	28.71	43.41	**74.53**
**Genus**	360	4673	45.03	35.51	18.82	17.54	**68.42**

Table S9. F1-score (F1) results obtained for viral taxonomic classification task regarding genome type, realm, kingdom, phylum, class, order, family, and genus. The classifiers used were Linear Discriminant Analysis (LDA), Gaussian Naive Bayes (GNB), K-Nearest Neighbors (KNN), Support Vector Machine (SVM) and XGBoost classifier (XGB).

Classification	N. Classes	N. Samples	$F1_{LDA}$	$F1_{GNB}$	$F1_{SVM}$	$F1_{KNN}$	$F1_{XGB}$
**Genome**	5	6089	0.6461	0.7306	0.7006	0.8368	**0.8645**
**Realm**	5	5799	0.7545	0.7996	0.7861	0.8881	**0.9214**
**Kingdom**	10	5788	0.7200	0.7630	0.7543	0.8411	**0.9031**
**Phylum**	17	5778	0.5763	0.5275	0.4822	0.6741	**0.8295**
**Class**	34	5845	0.4709	0.4526	0.4034	0.5969	**0.7983**
**Order**	48	5838	0.4418	0.4773	0.3870	0.5474	**0.7874**
**Family**	102	5990	0.3062	0.3573	0.1686	0.3456	**0.7325**
**Genus**	360	4673	0.3633	0.2815	0.0698	0.0610	**0.6525**

Table S10. F1-score (F1) obtained for the viral taxonomic classification task regarding genome type, realm, kingdom, phylum, class, order, family, and genus. The features used were the genome's sequence length (SL), the GC-content (GC) and the Normalized Compression (NC) values for the best model, the same model with IR configuration to 0, 1 and 2.

Classification	N. Classes	N. Samples	$F1_{NC}$	$F1_{NC+SL+GC}$	$F1_{AllFeatures}$
**Genome**	5	6089	0.7481	0.8642	**0.8645**
**Realm**	5	5799	0.7738	0.9194	**0.9214**
**Kingdom**	10	5788	0.7496	0.8999	**0.9031**
**Phylum**	17	5778	0.6248	0.8197	**0.8295**
**Class**	34	5845	0.5761	0.7837	**0.7983**
**Order**	48	5838	0.5557	0.7718	**0.7874**
**Family**	102	5990	0.4122	0.7155	**0.7325**
**Genus**	360	4673	0.3230	0.6378	**0.6525**

Software and Hardware recommendations

The experiences of the manuscript can be replicated using a laptop, desktop, or server computer running Arch linux or Linux Ubuntu (for example, 18.04 LTS or higher) with GCC (`https://gcc.gnu.org`),git and git LFS, Conda (`https://docs.conda.io`) and python version 3.6. The hardware must contain at least 8 GB of RAM and a 100 GB disk.

Reproducibility

Creating Project and intalling tools

The descriptions of reproducion is depicted bellow, for more detail see https://github.com/jorgeMFS/canvas. Install Git LFS:

```
mkdir -p gitLFS
cd gitLFS/
wget https://github.com/git-lfs/git-lfs/releases/download/v2.9.0/git-lfs-linux-amd64-v2.9.0.tar.gz
tar -xf git-lfs-linux-amd64-v2.9.0.tar.gz
chmod 755 install.sh
sudo ./install.sh
```

Get CANVAS project, create the docker and run it:

```
git clone https://github.com/jorgeMFS/canvas.git
cd canvas
docker-compose build
docker-compose up -d && docker exec -it canvas bash && docker-compose down
```

Inside the docker, give run permissions to the files and install tools using :

```
chmod +x *.sh
bash Make.sh;
```

Result Replication

The code was created in order to allow independent replication and reproduction of each step, this was done due to the extensive processing time required to filter and rearrange viral DB and extract the features and taxonomic information of each viral sequence. If you wish to rebuild database and feature reports extracted see the Database reconstruction subsection.

To obtain the Compression Benchmark plots run:

```
cd python || exit;
python select_best_nc_model.py;
```

To perform the synthetic sequence test run:

```
cd scripts || exit;
bash Stx_seq_test.sh;
```

To perform classification run the following code:

```
cd python || exit;
python prepare_classification.py; #recreate classification dataset
python classifier.py; #perform classifications
```

To perform the complete IR analysis and create:

- boxplots;
- 2d scatter plots;
- 3d scatter plots;
- top taxonomic group lists;
- Occurrence of each Genus.

Execute this code:

```
cd python || exit;
python ir_analysis.py; # Performs complete IR analysis
```

To perform the Human Herpesvirus analysis and obtain the plots run:

```
cd scripts || exit;
bash Herpesvirales.sh;
```

Database reconstruction

To run the pipeline and obtain all the Reports in the folder reports, use the following commands. Note that if you wish to recreate the features reports, you must perform the database reconstruction task.

If you wish to reconstruct the viral database, run the following script:

```
cd scripts || exit;
bash Build_DB.sh;
```

To create the features for analysis and classification (very time consuming, can take several days) run:

```
cd scripts || exit;
bash Process_features.sh;
```

To recreate the compression reports used for benchmark (very time consuming, can take several hours) run:

```
cd scripts || exit;
bash Compress.sh;
```

Phylogenetic Trees

The Phylogenetic Trees require GUI application. As such, the reproduction of the trees has to be performed outside of the docker on the Ubuntu system on the /canvas folder:

```
bash so_dependencies.sh #install Ubuntu system dependencies required for the script to run and Anaconda
conda create -n canvas python=3.6
conda activate canvas
bash Make.sh #install python libs
bash Install_programs.sh #install tools using conda
```

Afterwards, to obtain the Phylogenetic Tree plots run:

```
cd python || exit;
python phylo_tree.py;
```

Click here

C
li
OXFORD

Click here to access/download;Figure;boxplot_1.pdf

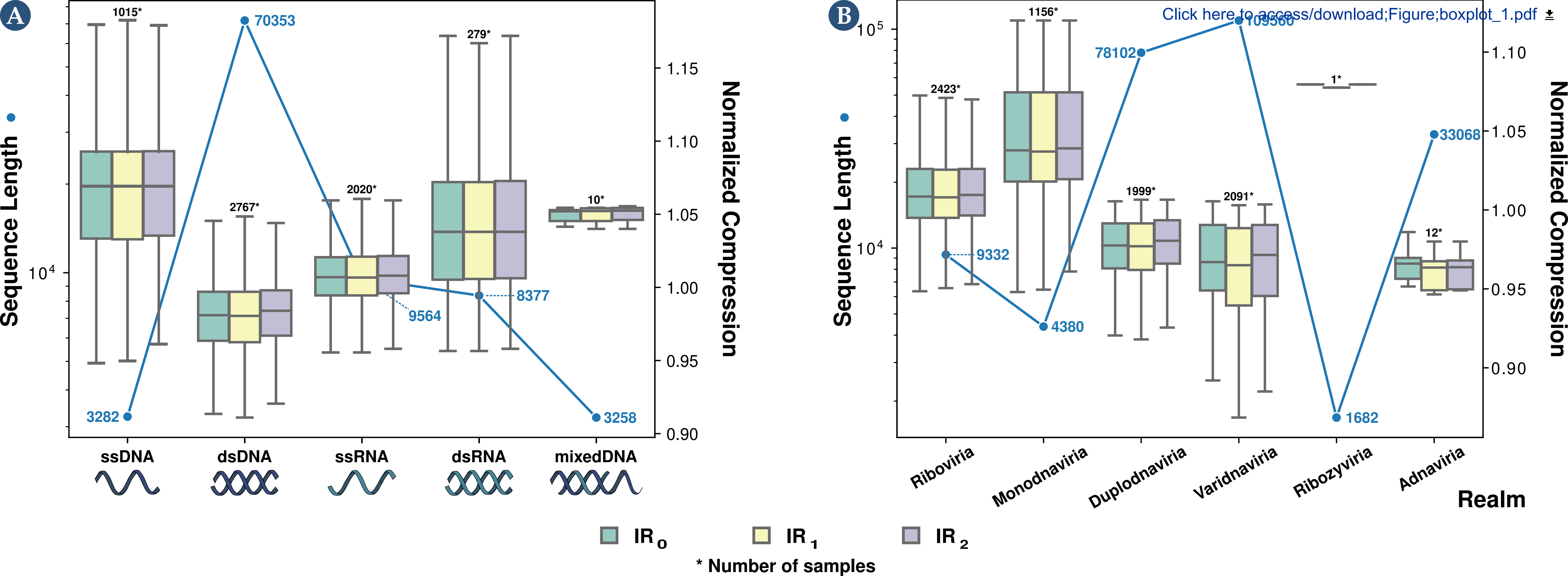

Click here to access/download;Figure;boxplot_2.pdf

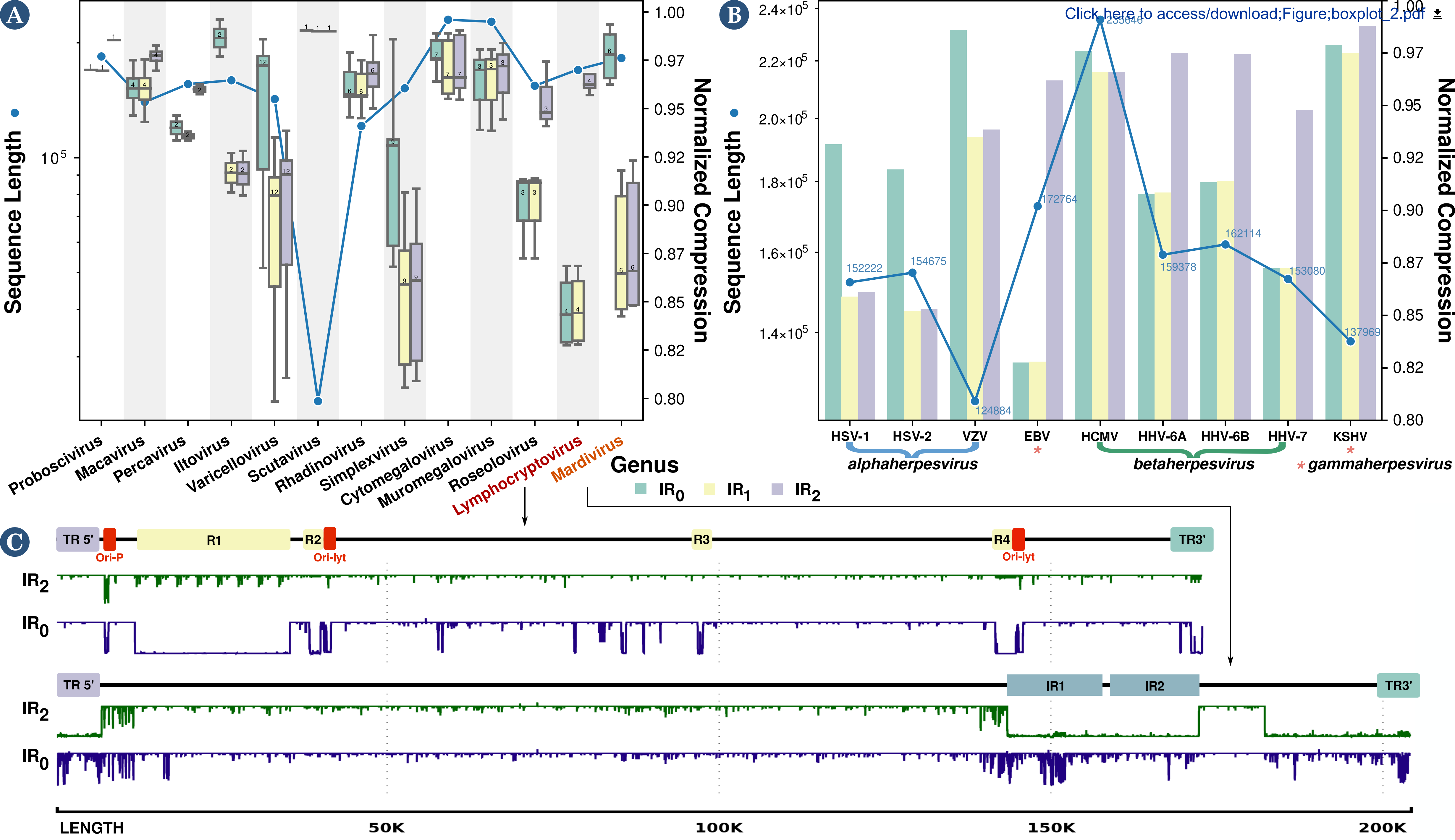

Click here to access/download;Figure;diff.png

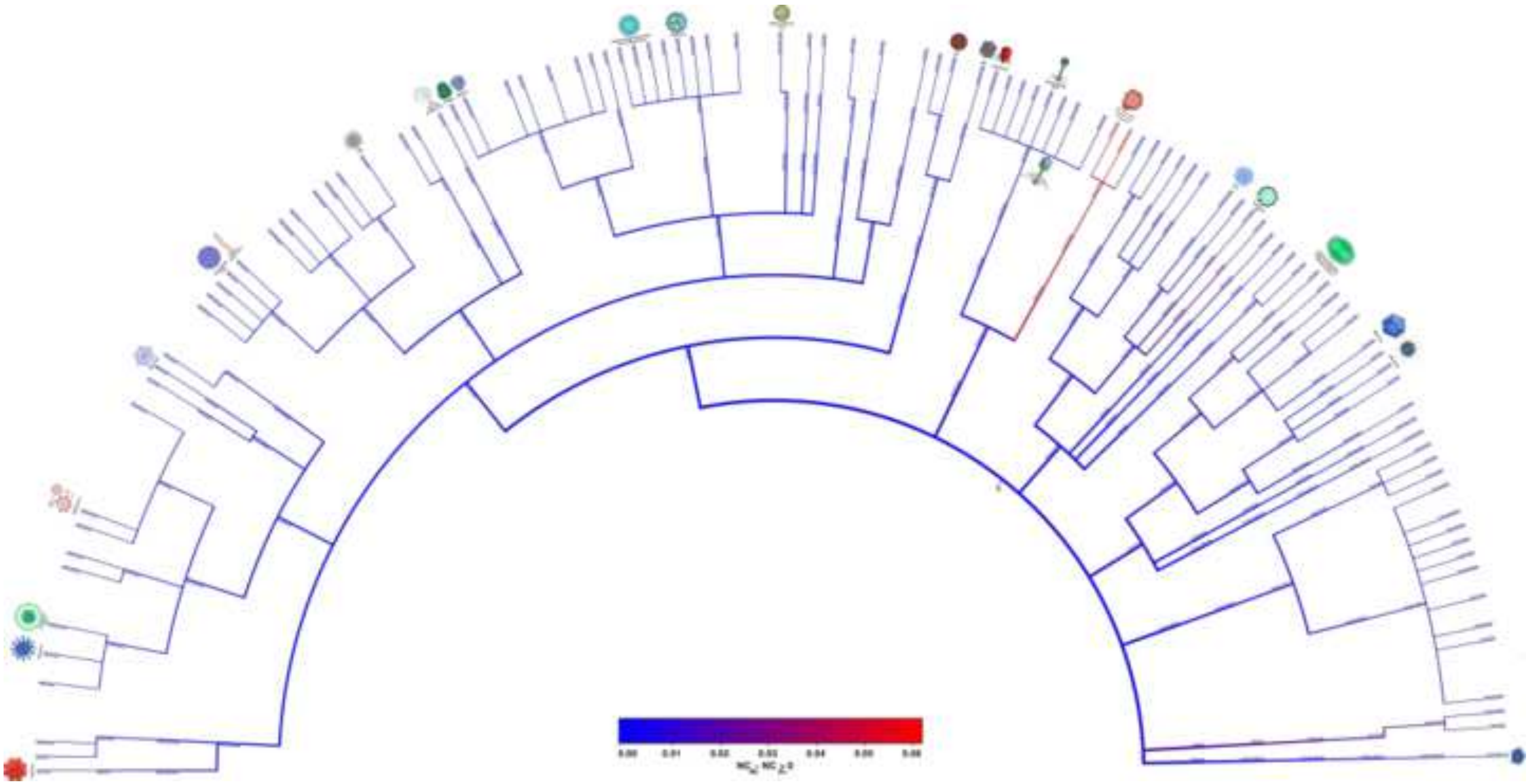

Click here to access/download;Figure;HHV.pdf

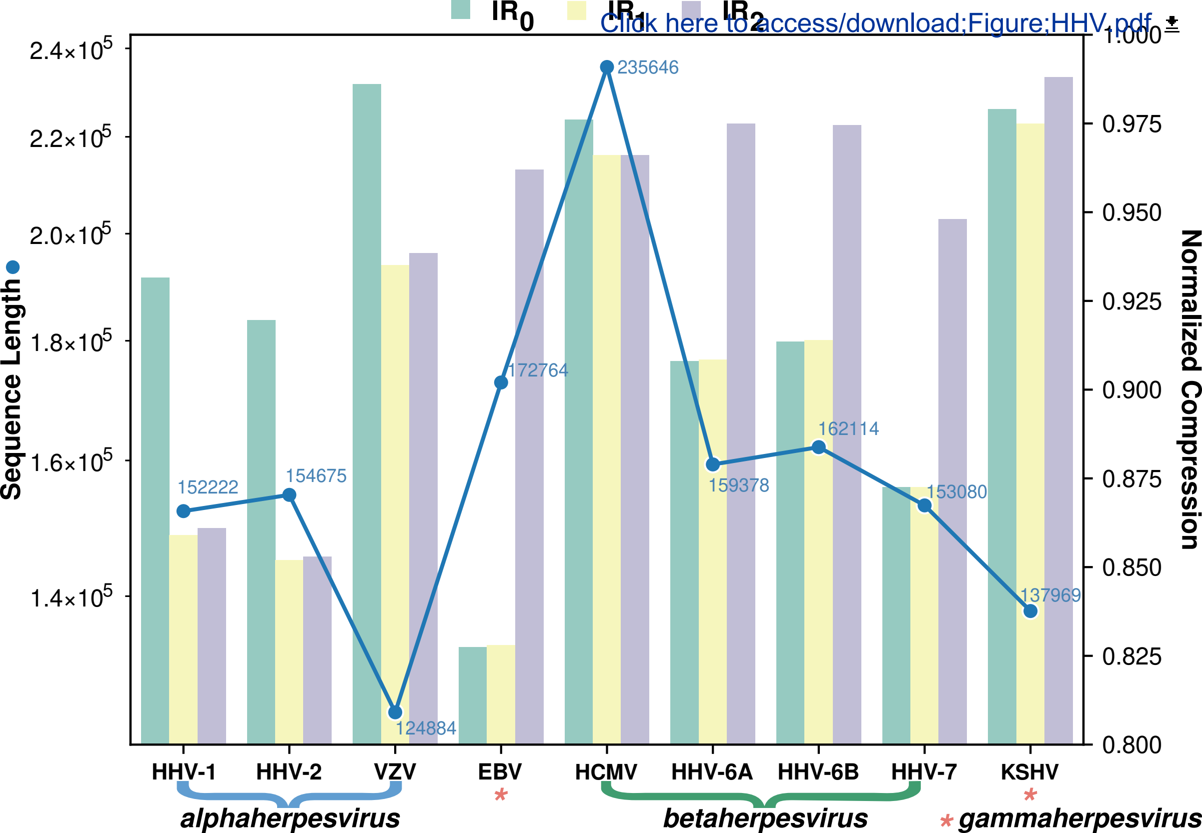

Click here to access/download;Figure;Model_selection.pdf

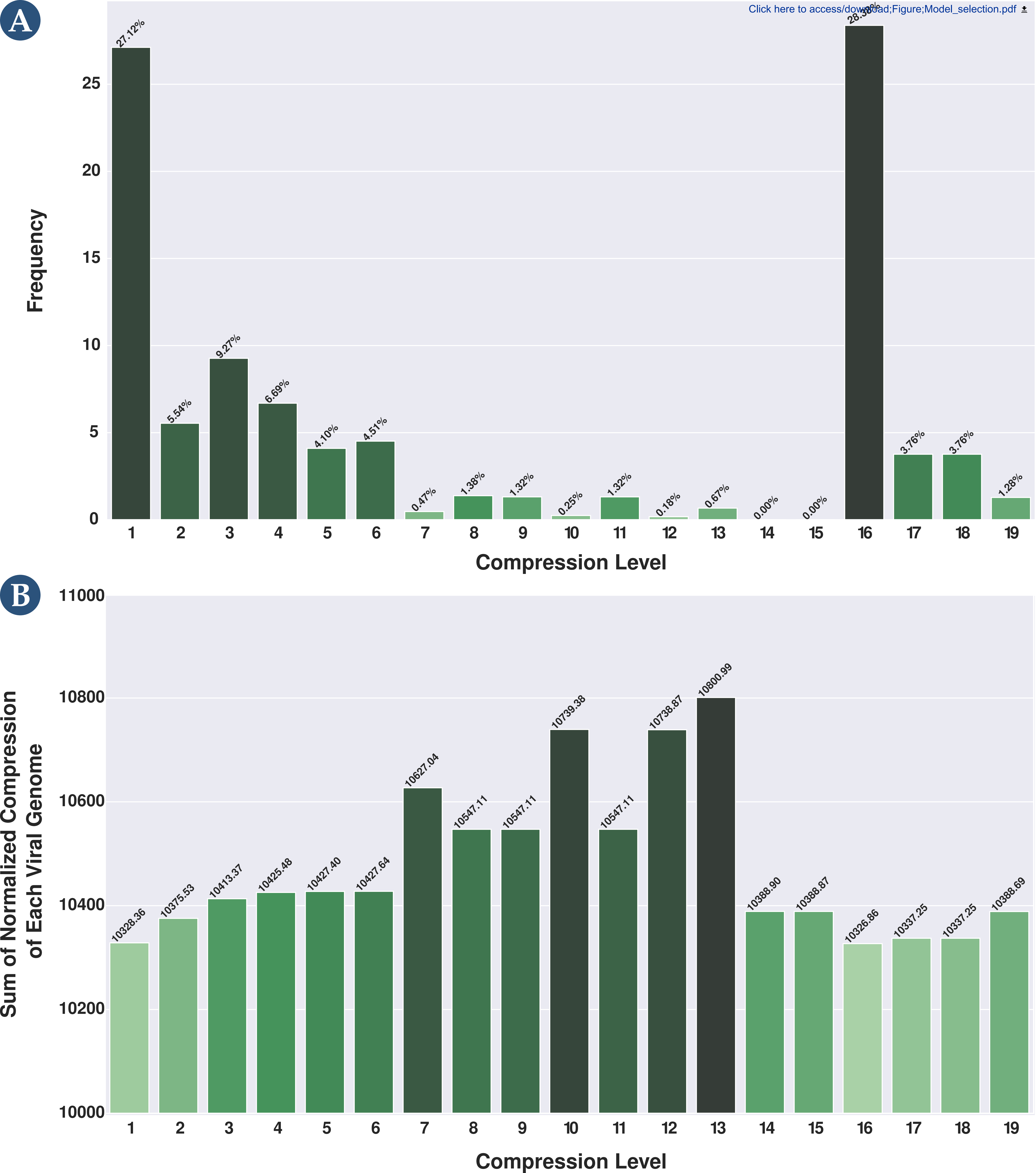

Click here to access/download;Figure;occuranc

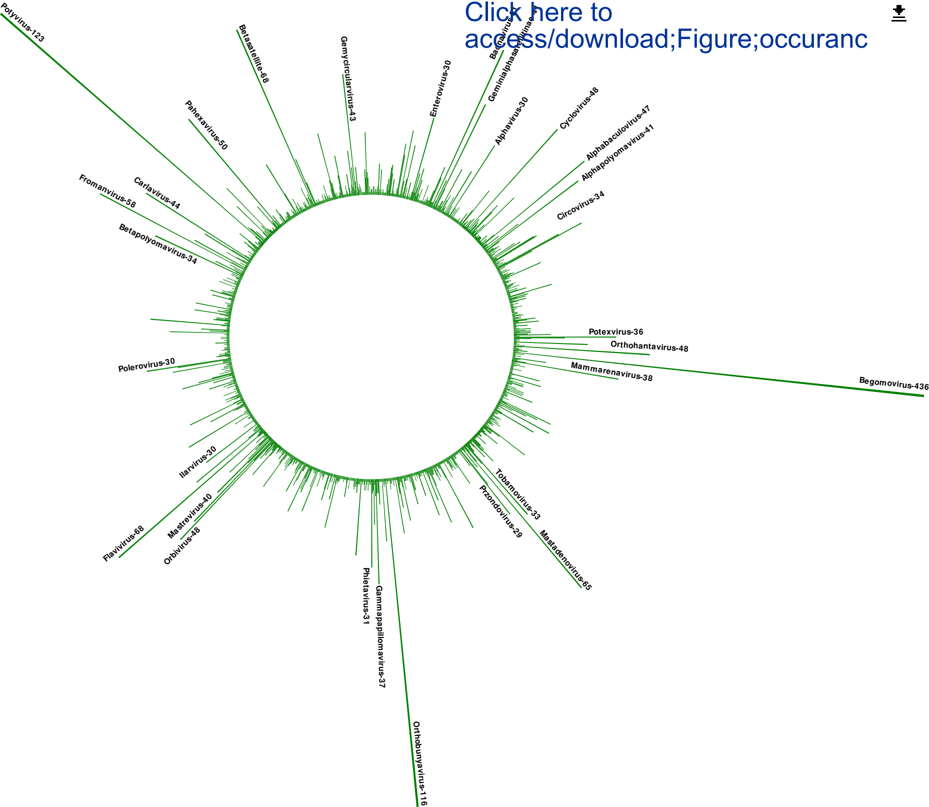

Click here to access/download;Figure;Realm.pdf

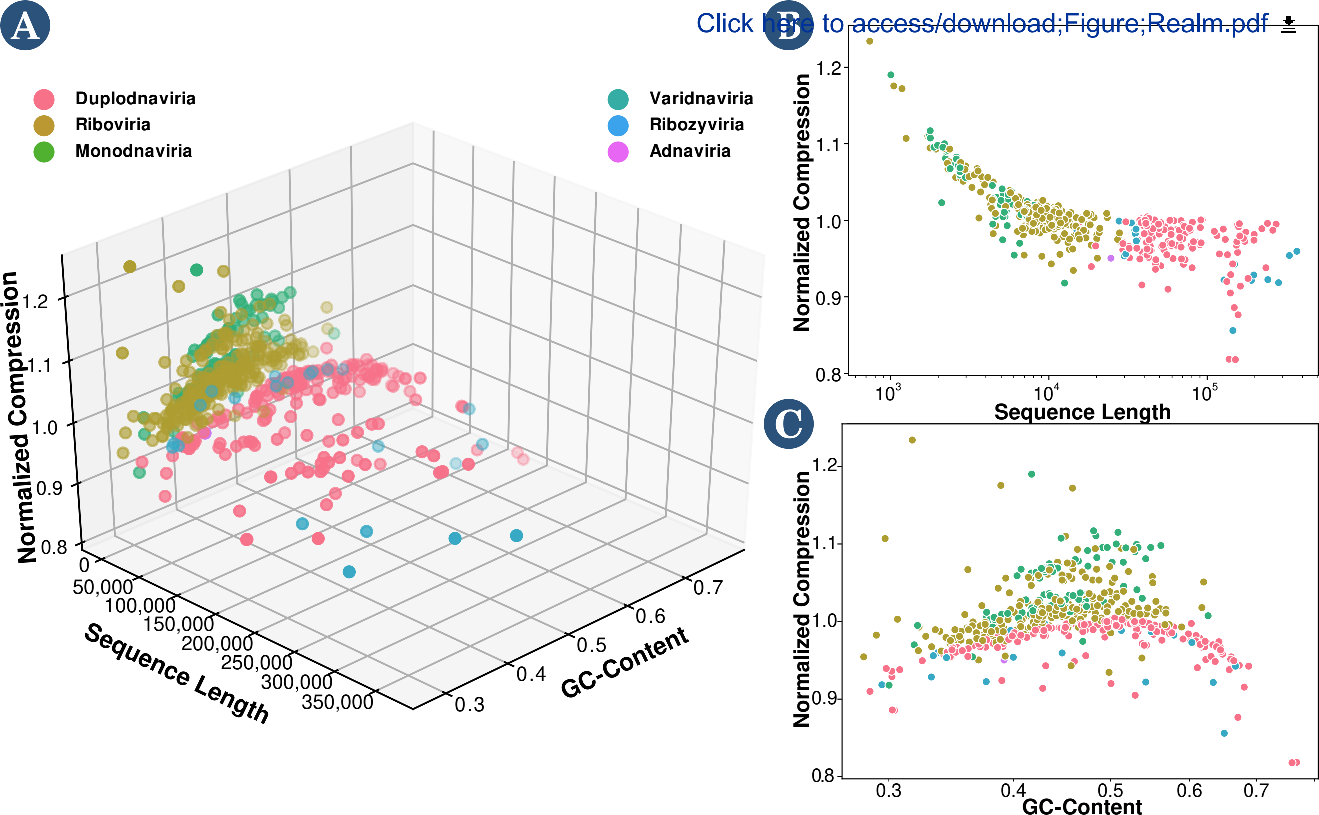

Click here to access/download;Figure;Synthetic_Seq

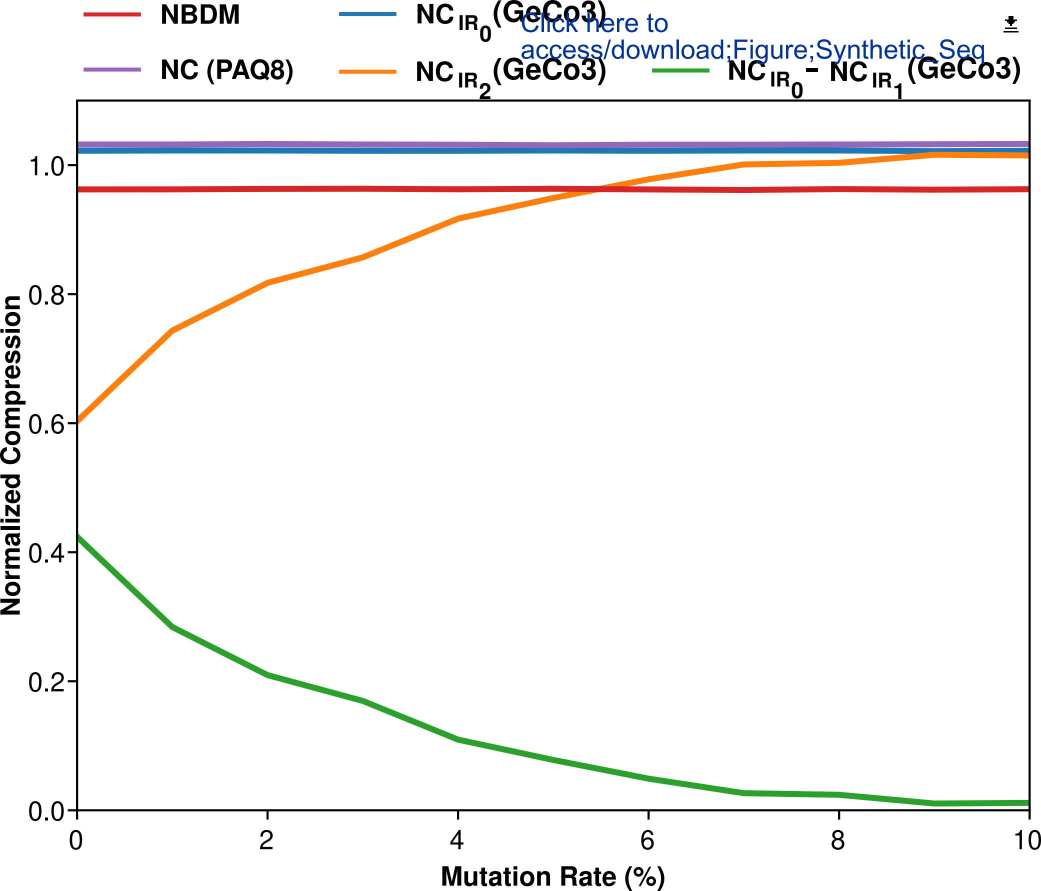

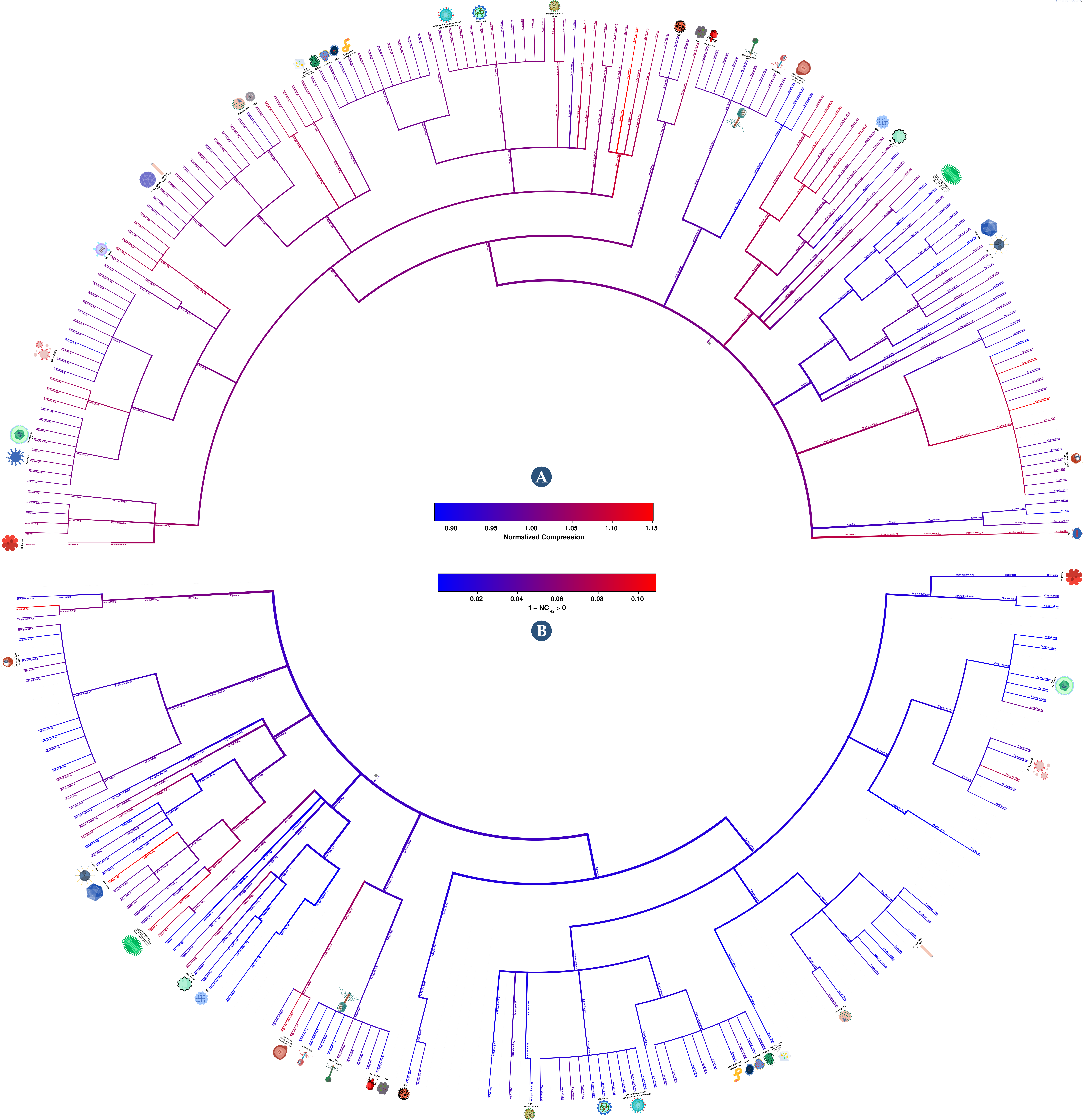

A
0.90
0.95
1.00
1.05
1.10
1.15
Normalized Compression
0.02
0.04
0.06
0.08
0.10
1 – NC_IR2 > 0
B

Click here to access/download;Figure;types_of_virus.pdf

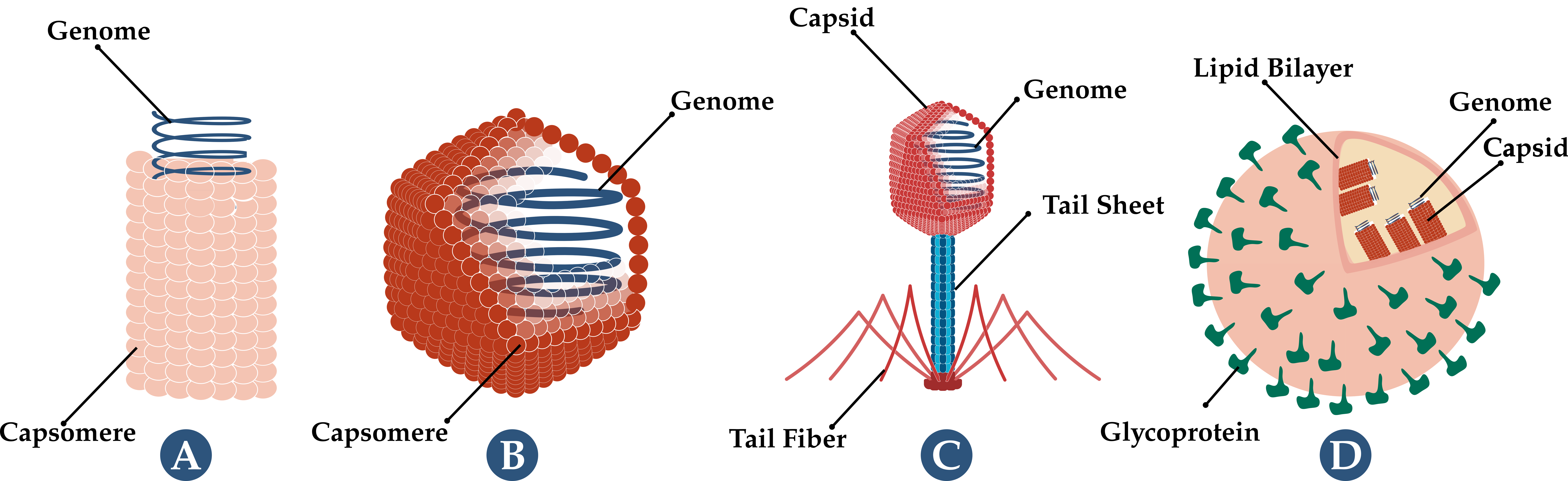

Supplement: giac079_GIGA-D-22-00044_Original_Submission [file giac079_giga-d-22-00044_original_submission.pdf]
