# Supplementary material for: The complexity landscape of viral genomes
Source: Gigascience. 2022 Aug 11;11:giac079. doi: 10.1093/gigascience/giac079 (PMC9366995; doi:10.1093/gigascience/giac079)
Supplement: giac079_Response_to_Reviewer_Comments_Original_Submission [file giac079_response_to_reviewer_comments_original_submission.pdf]

Author's Response To Reviewer Comments

Close

Answers to the editor and reviewers of:
"The complexity landscape of viral genomes"
J. M. Silva, D. Pratas, T. Caetano, S. Matos

Dear Editor,

We greatly appreciate the opportunity given to review our manuscript. We would like to thank the Reviewers, whose suggestions allowed us to improve our manuscript in many ways. We have now addressed the points raised by them, as outlined in blue in this revision letter. Following their suggestions, appropriate changes have been introduced to the manuscript, as shown in orange. We also added some classification results and improved the prior ones slightly. We hope we have been able to address their concerns and that our manuscript is now suitable for publication in GigaScience.

Reviewer 1

Reviewer: This manuscript presents a complexity analysis of virus genomes. Due to the fast evolution of viruses, alternative measures for comparing genomes are of interest. Nevertheless the authors could improve the presentation of the biological insights gained with this new approach.

Authors: We thank the reviewer for the revision and appreciate the comments. We trust that the changes introduced in the manuscript and the answers below address these comments.

Reviewer: It would be of interest to the reader which previous conclusions were drawn from the complexity approach. Although references are mentioned (7-13), this section is very short in the introduction.

Authors: We thank the reviewer for the pertinent comment. The information was expanded to encompass the conclusions drawn from each work (page 2).

Text added to the manuscript: There are many examples of these studies. Specifically, compression has been used to detect repeated sequences in the Plasmodium falciparum DNA, and observed patterns were related to large-scale chromosomal organization and gene expression control [28]. The XMAligner tool [3] was created for pairwise genome local alignment, which considers a pair of nucleotides from two sequences related if their mutual information in context is significant. To measure the information content of nucleotides in sequences, they used a lossless compression method. Graph compression was used for comparing large biological networks [11]. This method was done by compressing the original network structure and then measuring the similarity of the two networks using the compression ratio of the concatenated networks. The method was applied to several organisms, and the results showed that this method could efficiently measure the similarities between metabolic networks. Compression was used to approximate the Kolmogorov complexity and applied to data derived from sequence alignment data [2]. This process identified a novel way of predicting three different aspects of protein structure: secondary structures, inter-residue contacts and the dynamics of switching between different protein states. An analysis of the complexity of different DNA genomes was performed, demonstrating various evolution-related findings linked with complexity, notably that archaea have a higher relative complexity than bacteria and eukaryotes on a global scale [22]. Metagenomic composition analysis of a sedimentary ancient DNA sample was performed using relative compression of whole-genome sequences [21]. The results showed that several viruses and bacteria expressed high levels of similarity relative to the samples. Finally, an alignment-free tool was created to accurately find genomic rearrangements of DNA sequences following previous studies, which took alignment-based approaches or performed FISH [14].

Reviewer: The paper is very narrative and contains too many background in details in many places. This distracts the reader and impedes the flow of the paper. I would suggest to shorten it substantially for conciseness. E.g. the section "Viruses Microbiology" is mainly text book knowledge, only mention what is

important for the manuscript; the section “Kolmogorov Complexity and Data Compression” can be shortened; only describe the approaches in “Classification” that are used in the paper. The paper also contains repetitions, e.g., that classes with less than four samples are discarded is mentioned twice in “Viral Classification”. Furthermore the manuscript often contains announcements which can be dropped, e.g., the last paragraph of the introduction or the first sentence of the Methods.

Authors: We thank the reviewer for the comment. We looked for and removed unnecessary repetitions in the text. Although we understand the reviewer’s opinion, since the article has several areas involved, namely, Kolmogorov complexity, compression, genomics, and virology, we think it is essential for the reader to have an overview description of each since the background of each reader can differ.

Reviewer: The statement “an organism with a genome high in GC-content is rich in energy and more prone to mutation” is unclear.

Authors: We thank the reviewer for the comment. We tried to clarify the manuscript (page 5-6), and hopefully, it is now better explained.

Text added to the manuscript: GC-content is variable between different organisms and correlates with the organism’s life-history traits, genome size [24], and GC-biased gene conversion [8]. Furthermore, in RNA viruses, excess C to U substitutions accounted for 11–14% of the sequence variability of viruses, indicating that a decrease in GC-content is a potent driver of RNA viruses’ diversification and longer-term evolution [26]. As such, this measure helps perform viral classification. On the other hand, it was shown that the number of base stackings (typical arrangement of nucleobases found in the three-dimensional structure of nucleic acids) is one of the most critical elements contributing to the thermal stability of double-stranded nucleic acids. Furthermore, due to the relative locations of exocyclic groups, GC pairings have higher stacking energy than AT or AU pairs [30]. This energy accumulation in the GC pair in an organism’s genome makes the DNA more prone to mutation. Thus, over time, a species tends to decrease its GC content to become more stable [5], giving us further information regarding viral characterization.

Reviewer: The “Synthetic sequence benchmark” section is not related to virus genomes. The authors simulate long inverted repeats of 5000nt, it is thus unclear how the results are relevant for the viral complexity analysis.

Authors: Thanks for the comment. One of the main goals of our manuscript was to identify and quantify inverted repeats abundance in viral genomes. Thus, it was necessary to verify and select which compressors were capable of identifying them. As such, we analysed the benchmark provided by [19] and selected from the pool of compressors the ones with the highest compression ratio and that we had the best experience as developers (GeCo3, cmix, PAQ8). The synthetic sequence test with inverted repeats and increased mutation proves that GeCo3 is well suitable for the task. Furthermore, we compared cmix and GeCo3 regarding compressibility and computational time. The results showed that GeCo3 slightly outperformed in compression capability, and its computational time is three orders of magnitude faster than cmix. With these considerations, several overall improvements to the manuscript have been made to explain this better.

Reviewer: The authors find differences in complexity for different viral groups and mention that this is related to sequence length. Although this is expected, I think that it is worthwhile to describe this relationship more deeply. In Fig. 4 only one sequence length of each viral group is shown. This is misleading, since viruses inside a group can have a wide distribution of sequence lengths. To find genomes with interesting complexity patterns, it would instead be interesting to look at the relationship of length and complexity more
in detail within each group.

Authors: We thank the reviewer for the comment. Image 4 shows the broad picture for Genome type and Realm of viruses. This relation can indeed be observed in-depth and in detail for each group. Unfortunately, showing and discussing all this massive information is unfeasible in a single manuscript. We try to overcome this through supplementary tables (S5, S6 and s7), which show the top results of each taxonomic group, and through the extensive site, where each taxon in a specific taxonomic group (from Realm to family) has a similar plot describing the average length-complexity relationship.

Reviewer: By definition, phylogenies display the evolutionary relationships among organisms. I am not convinced that the NC measure used here to estimate trees, does indeed aim to reflect evolutionary

relationships. Instead it aims to show similarities and I thus think that the trees shown are rather clustering trees instead of phylogenetic trees.

Authors: Thank you for the comment. These charts reflect evolutionary relationships since their branch structure was created purely based on taxonomic information provided by NCBI. The colour shows the viral complexity or the abundance of IRs. However, as pointed out by another reviewer, the term phylogenetic tree is not the most correct to describe our graphs, but cladogram. Since, contrarily to our graphs, the branch lengths are proportional to the extent of difference between sequences or the time passed since divergence in phylogenetic trees. As such, the name term was updated in the manuscript.

Reviewer: In the viral classification section it is unclear at which level the 80-20 train test split was performed. Randomly choosing genomes from the set before dereplicating them can be misleading since highly similar genomes can be in the test and training data set.

Authors: The 80-20 train-test split was performed randomly but in a stratified way to ensure the representability of each class in both the training and test sets. This type of splitting was performed independently for each taxonomic classification task. In addition, and to ensure robustness of the results, the values presented represent the average accuracy and F1-score over 50 executions of each classification task. Regarding the presence of similar genomes, although possible, it is not so common since we are working with complete reference genomes, which are very few per viral species (usually 1 or 2). They are unique, meaning they possess mutations that differ them from one another, and by performing stratification of the unbalanced dataset, we are ensuring that the split possesses representability of all classes.

Reviewer: The focus on inverted repeats is not completely clear. Are they detected in the viruses known to have ITRs?

Authors: We thank the reviewer for the comment. To sum it up and answer the reviewer's question, yes, they are detected in viruses known to have ITRs. Furthermore, this detection is shown in synthetic and natural sequences in the manuscript. As explained in the background section, inverted repeats play many essential roles in organisms, one of which is to serve as inverted terminal repeats. A good example is provided by Toppinen et al. [29], where it was found that the inverted terminal repeat (ITR) sequences were crucial for B19V replication. By quantifying inverted repeats in the genome using GeCo3, we show novel behaviours and possible functions in viral groups associated with the IRs.

Reviewer: The manuscripts presents a Discussion section. Nevertheless the Results section already contains lots of discussion. A "Results and Discussion" section might be more appropriate.

Authors: We thank the reviewer for the pertinent comments. Despite being true, we consider separating both to be more helpful since we can emphasise the most relevant topics in the discussion section while being more detailed in the analysis performed in the results. We hope the reviewer understands our position.

Reviewer: The paper is accompanied by a website. Although visually appealing, the precise usage of the website is unclear. There is no search function if the user would be interested in a particular genome. Inside a family, one can see the tree with virus names but there is no link to the genomes that went into the analysis.

Authors: We thank the reviewer for the comment. Although the reviewer has mentioned interesting ideas, they would go beyond the scope of this work. The site's goal is to show the entirety of the results obtained by the analysis of this work. We did this to complement our work since discussing and showing all the results in the manuscript would be unfeasible. All plots shown on the website are replicable through the source code. Furthermore, as the reviewer pointed out, the website does have not a search tool function. However, the content is easy to find by navigating the website since all the content is catalogued and organized by taxonomic group and alphabetic order.

Reviewer: Fig. 1 The letters in the figure are not matching the letters in the legend.

Authors: We thank the reviewer for such a pertinent comment. The legend has been updated accordingly.

Reviewer 2

Reviewer: In this study the authors investigate the complexity of viral genomes. I think the topic is interesting and the performed analysis is comprehensive.

Authors: We thank the reviewer for these comments, which helped improving the quality of the work and its presentation.

Reviewer: 1. It’s nice that PAQ8 was included. I think it would be interesting to include cmix, as it seems to generally provide a stronger compression and therefore a better approximation of Kolmogorov’s complexity.

Authors: We thank the reviewer for the comments. We have now added support for cmix and used it in the synthetic data analysis and tested it in a sample of viral genomic sequences. Unfortunately, cmix could not obtain the desired performance in this specific case. As can now be observed in Figure 3, it did not outperform PAQ8 in the case of synthetic data. Furthermore, it cannot detect inverted repeats, which is an essential aspect of this work. Furthermore, as can be observed in Figure S1 added in the supplementary material, cmix takes significantly more computational time than GeCo3 (on average, three orders of magnitude faster than cmix). Additionally, on average, it did not provide a better compression ratio, at least in the small sample tests (HHV, supplementary material Figure S1). We conclude that the computational time of cmix makes its use in the large dataset used in this study an unfeasible task.

Text added to manuscript (page 7): Cmix and GeCo3 are state-of-the-art genomic compressors. To assess the viability of each compressor, we tested their computational time and NC values on a small sample consisting of 8 medium size viral genomes. The results, presented in Figure S1 of the supplementary material, show that the compression ratio of GeCo3 is, on average, slightly better, with a much more reasonable computational time (on average, three orders of magnitude faster than cmix). As such, for the remaining of the work, we consider the GeCo3 compressor.

(...) iii) Cmix (...) All other compressors (cmix and PAQ8) could not detect IRs and compress the sequence.

Reviewer: 2. P6. “Currently, the state-of-the-art genomic compressors apply statistical and algorithmic model mixtures combined with arithmetic encoding.” - This sentence seems to use “state-of-the-art” in a narrow sense, referring to just compression strength. Other kinds of state-of-the-art compressors exist, for example those that prioritize a combination of compression strength and decompression speed, such as NAF (https://github.com/KirillKryukov/naf , https://doi.org/10.1093/bioinformatics/btz144). NAF uses a dictionary-based compression. Perhaps this part can modified to make it more clear that it discusses compressors providing the best compression strength.

Authors: We agree and updated the text accordingly (page 4).

Text added to the manuscript: Currently, state-of-the-art compressors have different objectives, such as optimizing for compression strength or prioritizing a balance between compression speed and compression capability. Examples of the latter are NAF (Nucleotide Archival Format) [18, 17] and MBGC (Multiple Bacteria Genome Compressor) [10], which are more suitable for collections of data and frequently used by computational biologists. Compressors focused on compressibility at the expense of more computational resources, on the other hand, generally apply statistical and algorithmic model mixtures combined with arithmetic encoding.

Reviewer: 3. P6. “The best compression ratio performance for various genomic sequences is provided by XM [82], Jarvis [83], and Geco3 [14].” - As shown in Sequence Compression Benchmark, cmix provides stronger compression than these compressors. (http://kirr.dyndns.org/sequence-compression-benchmark/ , https://doi.org/10.1093/gigascience/giaa072).

Authors: We thank the reviewer for such a pertinent comment. The text has been updated to accommodate the cmix compressor (page 4).

Text added to the manuscript: Among the best compressors regarding compression ratio performance for various genomic sequences, the best results are provided by cmix [15], XM [4], Jarvis [23], and

Geco3 [25].
For additional information regarding data compressors' compressibility capacity of genomic sequences, see [19]. Cmix [15] is a general-purpose lossless data compression program that optimises compression ratio at the cost of high CPU/memory usage. It is based on PAQ compressors [16, 1] but dramatically increases the amount of processing per input bit and computational memory. Current updates include LSTM (Long Short-Term Memory) based models [13].

Reviewer: 4. P6. "An efficient compressor, C(x), provides an upper bound approximation for the Kolmogorov complexity" - In the following text and formula, C(x) seems to be used as a number. Therefore the sentence is confusing. It seems that C(x) is not "an afficient compressor", but rather, size of data compressed with an efficient compressor.

Authors: We thank the reviewer for such a pertinent comment. The text has been updated to accommodate the changes (page 5).

Text added to the manuscript: An efficient compressor provides an upper bound approximation for the Kolmogorov complexity. Specifically, $K(x) < C(x) \leq |x| \log 2 |A|$, where K(x), is the Kolmogorov complexity of the string x in bits, C(x) is the compressed size of x in bits, and —x— is the length of string x in the appropriate scale.

Reviewer: 5. P6. "$K(x) < C(x) <= |x|$" - I think pigeonhole principle implies that you can't design an efficient compressor such that C(x) is always <= |x|. For some inputs C(x) must be greater than |x|. Also, 5by chance you may produce C(x) that is identical to K(x), therefore "$K(x) <= C(x)$" would probably be more accurate.

Authors: We thank the reviewer for such a pertinent comment. In fact, we had an error in our expression, since $K(x) < C(x) <= |x|$ is only correct for binary. The correct expression being $K(x) < C(x) \leq |x| \log 2 |\Sigma|$. This expression considers asymptotic entries. Also, we removed constants that asymptotically become irrelevant. We can always create a program that the compressed measure is the message itself plus a small constant. The changes to the manuscript are shown in the previous answer (page 5).

Reviewer:6. P6. "The normalized version, known as the Normalized Compression (NC)" - Normalized Compression implies the process or method of compression. but here it is used to represent "Normalized Compressed Size", or something like that. This confusing terminology does not help the reader. It seems that NC here refers to the inverse of Compression Ratio (Original data size / Size of compressed data), which would be more natural and easy to understand. On P8 you write "We evaluated the frequency where each level yielded the lowest NC (provided the best compression for a given sequence; Figure 2 A)" - Lower NC means higher compression - which is counterintuitive. Higher "compression" should correspond to stronger compression, which would be the case if Compression Ratio was used as a measure instead.

Authors: We thank the reviewer for the comments. Normalized Compression (NC) was first defined in [22]. It is analogous to Normalized compression distance (NCD) [6]. However, instead of providing a comparative measure, it gives us a compression ratio by the way it is normalized.

Reviewer:7. P7. "There was a need to determine the sequences with the highest normalized compression capacity (NCC) in some cases. When the compressor was only using the subprogram IR2 , NCC was computed as $NCCIR2 (x) = 1 - NCIR2$." - The purpose of this derived measure is not clear. NC value is confusing enough by itself already, why 1 - NC is needed? Since NCC only depends on NC, why not simply use NC by itself? In paper you then use both NC and NCC side by side, which have the opposite scales: Stronger compression gives smaller NC, but larger NCC.

Authors: We thank the reviewer for the comments. The NCC results were obtained by $NCC = 1 - NCIR2 > 0$. Since IR2 uses an IR detection sub-program without regular context models, a lower NC indicates a higher compression and, therefore, the presence/detection of inverted repeats. In addition, by discarding negative values, we have a sample of only sequences that have detected IRs, making this part of the analysis more accessible and creating a more explicit depiction of the viral groups with IRs when observing Figure 6. We have tried to simplify the text description (page 5).

Reviewer: 8. P7. "The dataset is composed of 12,163 complete reference genomes from 9,605 viral taxa retrieved from NCBI database on 22 of January 2021 using the following url

https://tinyurl.com/ncbidtbs." - Please include the actual url in the methods section, rather than depending on tinyurl.

Authors: We thank the reviewer for the comment. The text has been updated accordingly.

Reviewer: 9. P7. "Secondly, a filter was applied to remove outlier sequences. Specifically, after computing all sequences' length, GC-Content, and Normalized Complexities, sequences whose measure fell outside 3 (approximately 0.03% of all sequences) of any measure were removed. After filtering, 6,091 of the initial 12,163 sequences were kept." - This seems to be a bit of circular logic, regarding classification accuracy. When designing an automatic virus genome classifier, arbitrary precision can be achieved by removing various amounts of outlier sequences beforehand.

Authors: We thank the reviewer for the comment. We changed the text to try to improve the overall filtering explanation (page 6). The vast majority of the discarded sequences were the ones that did not meet the first requirement: "Firstly, using the taxonomic metadata, sequences that did not hold complete taxonomic information down to the genus rank and any sequences that maintained a taxonomic description of unclassified were removed." A minimal number of sequences was removed in the second filtering (182 sequences). As such, this second process was intended to remove sequences which most probably had errors in the assembly process and therefore have a high probability of being inaccurate or incorrectly constructed.

Text added to the manuscript: A total of 182 sequences were removed since they most likely have errors in the assembly process or contamination.

Reviewer: 10. P11. "Furthermore, we performed classification using seven different features: sequence length (SL), GC-content (GC), the Normalized Compression (NC) values for the best performing model, and the NC of the same model with IR configuration to 0, 1 and 2." - It's unfortunate that sequence length was included among the features used for classification, as this significantly reduces the value of this method. In actual analysis of viral sequences (both environmental, and integrated in genomes), we often don't know the full length of the original viral genome, but only see a DNA or RNA fragment. Designing a classifier that does not need sequence length would be potentially much more useful in practice.

Authors: We thank the reviewer for such a pertinent comment. We have taken into account the insights provided by the reviewer and now show the results obtained without the sequence length feature. Although we obtain a lower accuracy and F1-score, these results are still reliable as a fast and efficient identification method for viral taxonomic identification in the case of environmental or integrated genome samples. We discuss further this results in the discussion section (page 12).

Text added to the manuscript: Furthermore, when analysing viral sequences from environmental samples or integrated genome samples, the length of the original viral genome is often not known. Therefore, we computed the accuracy of a model that does not include this feature. Although we obtain a lower accuracy and F1-score, the results indicate that the method is still reliable for fast and efficient viral taxonomic identification in these scenarios.

Reviewer: 11. "As far as we know, this is the first attempt at performing this type of reference-free classification. As such, for comparison purposes, we assessed the outcomes obtained using a random classifier." -There are many studies on alignment-free sequence comparison and classification. Some examples specifically for viruses: https://doi.org/10.1016/j.meegid.2021.105106 , https://doi.org/10.1016/j.csbj.2021.10.029, https://doi.org/10.1038/srep40712 , https://doi.org/10.1515/sagmb-2018-0004 .

Authors: We thank the reviewer for these insights. Our method is not only alignment-free but also feature-based, which provides a higher level of flexibility since it does not resort directly to the reference genomes but instead to features that the biological sequences share. Nevertheless, we updated the article's information to accommodate more information and results regarding alignment-free methods (page 10-11).

Text added to the manuscript: Although sequence alignment is essential for genomic analysis, the fact that pairwise and multiple alignment methods are often slow methods led to the popularization of fast alignment-free methods for sequence comparison. Most alignment-free methods are based on word frequencies for words of a fixed length or word-matching statistics. Others use the length of maximal

word matches, and others rely on spaced-word matches (SpaM). These inexact word matches allow mismatches at certain predefined positions and can accurately estimate phylogenetic distances between DNA or protein sequences using a stochastic model of molecular evolution [20]. This approach has also been updated as the Multiple Spaced-Word Matches (Multi-SpaM) method, which is based on multiple sequence comparison and maximum likelihood [7]. Regarding viral sequences, many studies were performed on alignment-free sequence comparison and classification. For instance, Garcia et al. [9] developed a dynamic programming algorithm for creating a classification tree using metagenome viruses. For the classification tree creation, k-mer profiles of each metagenome virus were created, and proportional similarity scores were generated and clustered. Using the JGI metagenomic and NCBI databases, the authors were able to identify the correct virus (including its parent in the classification tree) 82% of the time. Zhang et al. [31] created an alignment-free method that employed k-mers as genomic features for a large-scale comparison of complete viral genomes.
After determining the optimal k for all 3,905 complete viral genomes, a dendrogram was created, which shows consistency with the viral taxonomy of the ICTV and the Baltimore classification of viruses. He et al. [12] proposed an alignment-free sequence comparison method for viral genomes based on the location correlation coefficient. When applied to the evolutionary analysis of the common human viruses, including SARS-CoV-2, Dengue virus, Hepatitis B virus, and human rhinovirus and achieves the same or even better results than alignment-based methods. Finally, Huang et al. [27] proposed a classification method based on discriminant analysis employing the first and second moments of positions of each nucleotide of the genome sequences as features and performed classification of genomes regarding their Baltimore classification and family (12 families) and obtained a maximum value of accuracy of 88.65% and 85.91%, respectively. Despite being pertinent, the alignment-free studies are not directly comparable due to sample size, absence of classification metrics and source code. Furthermore, the method proposed in this work is not only alignment-free but also feature-based, providing a higher level of flexibility since it does not resort directly to the reference genomes but instead to features that the biological sequences share. Therefore, we compared our results with the outcome obtained using a random classifier as a measure of comparison.

Reviewer: 12. P12. “Figure 6. Phylogenetic tree showing average NC of each viral group (A), and the normalized compression capacity (NCC) (B).” - What Figure 6 shows is more accurately described as a cladogram, not a phylogenetic tree. In a phylogenetic tree, branch lengths are proportional to the extent of difference between sequences, or to time passed since divergence. However in Figure 6 all branches are of the same length, implying that probably simply taxonomic structure is shown.

Authors: We thank the reviewer for the comment. The text has been updated accordingly.

Reviewer: 13. P12. “The usage of a specialized compressor is crucial to quantify the complexity present in a genome accurately. Specialized compressors outperform general-purpose compressors because they take into account the intrinsic nature of the data.” - General-purpose cmix currently outperforms specialized compressors in the Sequence Compression Benchmark.

Authors: We thank the reviewer for such a pertinent comment. The text has been updated accordingly (page 12).

Text added to the manuscript: The usage of a specialized compressor is crucial to accurately quantify the complexity present in a genome and detect the intrinsic algorithmic nature of the data.

Reviewer: 14. P3. “Using a state-of-the-art genomic compressor on an extensive viral genomes database, we show that dsDNA viruses are on average the most redundant viruses while ssDNA viruses are the lowest.” - Maybe replace ”lowest” with ”least”, or otherwise rephrase.

Authors: We thank the reviewer for such a pertinent comment. The text has been updated accordingly.

Reviewer: 15. P4. “their understanding is still relatively limited” => our understanding of viruses is still relatively limited”.

Authors: We thank the reviewer for such a pertinent comment. The text has been updated accordingly.

Reviewer: 16. Overall, the text is unnecessarily complicated. Many parts can be simplified and described in more simple terms. E.g. P13 “a lower NC and abundance of inversions present in herpesvirus” => ”a higher compressibility and abundance of inversions present in herpesvirus”.

Authors: We thank the reviewer for such a pertinent comment. The text has been updated accordingly.

References

[1] Avatar Johannes Buchner. PAQ. accessed May 16, 2020. URL: https://github.com/JohannesBuchner/paq/.
[2] Robert Paul Bywater. "Prediction of protein structural features from sequence data based on Shannon entropy and Kolmogorov complexity". In: PloS one 10.4 (2015), e0119306.
[3] Minh Duc Cao, Trevor I Dix, and Lloyd Allison. "A genome alignment algorithm based on compression". In: BMC bioinformatics 11.1 (2010), pp. 1–16.
[4] Minh Duc Cao et al. "A simple statistical algorithm for biological sequence compression".In: 2007 Data Compression Conference (DCC'07). IEEE. 2007, pp. 43–52.
[5] Han Chen and Chris-Kriton Skylaris. "Analysis of DNA interactions and GC content with energy decomposition in large-scale quantum mechanical calculations". In: Physical Chemistry Chemical Physics 23.14 (2021), pp. 8891–8899.
[6] Rudi Cilibrasi and Paul MB Vitányi. "Clustering by compression". In: IEEE Transactions on Information theory 51.4 (2005), pp. 1523–1545.
[7] Thomas Dencker et al. "'Multi-SpaM': a maximum-likelihood approach to phylogeny reconstruction using multiple spaced-word matches and quartet trees". In: NAR Genomics and Bioinformatics 2.1 (Oct. 2019). lqz013. ISSN: 2631-9268. DOI: 10.1093/nargab/lqz013. eprint: https://academic.oup.com/nargab/article-pdf/2/1/lqz013/34054190/lqz013.pdf.
[8] Laurent Duret and Nicolas Galtier. "Biased gene conversion and the evolution of mammalian genomic landscapes". In: Annual review of genomics and human genetics 10 (2009), pp. 285–311.
[9] Benjamin J Garcia et al. "A k-mer based approach for classifying viruses without taxonomy identifies viral associations in human autism and plant microbiomes". In: Computational and structural biotechnology journal 19 (2021), pp. 5911–5919.
[10] Szymon Grabowski and Tomasz M Kowalski. "MBGC: Multiple Bacteria Genome Compressor". In: GigaScience 11 (2022).
[11] Morihiro Hayashida and Tatsuya Akutsu. "Comparing biological networks via graph compression". In: BMC systems biology. Vol. 4. 2. BioMed Central. 2010, pp. 1–11.
[12] Lily He et al. "Alignment-free sequence comparison for virus genomes based on location correlation coefficient". In: Infection, Genetics and Evolution 96 (2021), p. 105106.
[13] Sepp Hochreiter and Jürgen Schmidhuber. "Long short-term memory". In: Neural computation 9.8 (1997), pp. 1735–1780.
[14] Morteza Hosseini et al. "Smash++: an alignment-free and memory-efficient tool to find genomic rearrangements". In: GigaScience 9.5 (2020), giaa048.
[15] Byron Knoll. Byronknoll/cmix: Cmix is a lossless data compression program aimed at optimizing compression ratio at the cost of high CPU/memory usage.Byron Knoll. accessed May 5, 2022. URL: https://github.com/byronknoll/cmix.
[16] Byron Knoll and Nando de Freitas. "A machine learning perspective on predictive coding with PAQ8". In: 2012 Data Compression Conference. IEEE. 2012, pp. 377–386.
[17] Kirill Kryukov. Kirillkryukov/NAF: Nucleotide archival format - compressed file format for DNA/RNA/protein sequences. accessed May 5, 2022. URL: https://github.com/KirillKryukov/naf.
10[18] Kirill Kryukov et al. "Nucleotide Archival Format (NAF) enables efficient lossless reference-free compression of DNA sequences". In: Bioinformatics 35.19 (2019), pp. 3826–3828.
[19] Kirill Kryukov et al. "Sequence Compression Benchmark (SCB) database—A comprehensive evaluation of reference-free compressors for FASTA-formatted sequences". In: GigaScience 9.7 (2020), giaa072.
[20] Burkhard Morgenstern. "Sequence comparison without alignment: The SpaM approaches". In: Multiple Sequence Alignment. Springer, 2021, pp. 121–134.
[21] Diogo Pratas and Armando J Pinho. "Metagenomic composition analysis of sedimentary ancient DNA from the Isle of Wight". In: 2018 26th European Signal Processing Conference (EUSIPCO). IEEE. 2018, pp. 1177–1181.
[22] Diogo Pratas and Armando J Pinho. "On the approximation of the Kolmogorov complexity for DNA sequences". In: Iberian Conference on Pattern Recognition and Image Analysis. Springer. 2017, pp. 259–266.
[23] Diogo Pratas et al. "A reference-free lossless compression algorithm for DNA sequences using a competitive prediction of two classes of weighted models". In: Entropy 21.11 (2019), p. 1074.
[24] Jonathan Romiguier et al. "Contrasting GC-content dynamics across 33 mammalian genomes: relationship with life-history traits and chromosome sizes". In: Genome research 20.8 (2010), pp. 1001–1009.

[25] Milton Silva, Diogo Pratas, and Armando J Pinho. “Efficient DNA sequence compression with neural networks”. In: GigaScience 9.11 (Nov. 2020). giaa119. ISSN: 2047-217X. DOI:10.1093/gigascience/giaa119. eprint: https://academic.oup.com/gigascience/article-pdf/9/11/giaa119/34251844/giaa119.pdf.
[26] Peter Simmonds and M Azim Ansari. “Extensive C-¿ U transition biases in the genomes of a wide range of mammalian RNA viruses; potential associations with transcriptional mutations, damage-or host-mediated editing of viral RNA”. In: PLoS pathogens 17.6 (2021), e1009596.
[27] Gordon K Smyth. “Statistical applications in genetics and molecular biology”. In: Linear models and empirical Bayes methods for assessing differential expression in microarray experiments (2004).
[28] Linda Stern et al. “Discovering patterns in Plasmodium falciparum genomic DNA”. In: Molecular and Biochemical Parasitology 118.2 (2001), pp. 175–186.
[29] Mari Toppinen et al. “Parvoviral genomes in human soft tissues and bones over decades”. PhD thesis. Helsingin yliopisto, 2021.
[30] Peter Yakovchuk, Ekaterina Protozanova, and Maxim D Frank-Kamenetskii. “Base-stacking and base-pairing contributions into thermal stability of the DNA double helix”. In: Nucleic acids research 34.2 (2006), pp. 564–574.
[31] Qian Zhang et al. “Viral phylogenomics using an alignment-free method: A three-step approach to determine optimal length of k-mer”. In: Scientific reports 7.1 (2017), pp. 1–13.

Close
