# Supplementary material for: The complexity landscape of viral genomes
Source: Gigascience. 2022 Aug 11;11:giac079. doi: 10.1093/gigascience/giac079 (PMC9366995; doi:10.1093/gigascience/giac079)
Supplement: giac079_Response_to_Reviewer_Comments_Revision_1 [file giac079_response_to_reviewer_comments_revision_1.pdf]

Author's Response To Reviewer Comments

Close

Dear Editor,

We greatly appreciate the opportunity given to review our manuscript. We want to thank the reviewer, whose suggestions allowed us to improve our manuscript. We have now addressed the points he raised, as outlined in this revision. Following the reviewer's suggestions, appropriate changes have been introduced to the manuscript. We hope we have addressed the reviewer's concerns and that our manuscript is now suitable for publication in GigaScience.

Reviewer

Reviewer: I thank the authors for taking all my comments into consideration. Unfortunately I find that only a subset of them are addressed in the current version of the manuscript. My main concern is that the main point of the manuscript is not clearly elaborated in the paper. The abstract, Key points, and the main message in the Results and Discussion should be consistent.

Authors: We thank the reviewer for the revision and appreciate the comments. We trust that the changes introduced in the manuscript and the answers below address these comments.

Reviewer: 1. I previously noted that the paper is very narrative and contains too many background details. As I do not have a version with tracked changes, I do not see if it has been shortened as some points. However, I notice that it still contains textbook knowledge, e.g., in the "Viruses Microbiology" section. I understand that this is an interdisciplinary manuscript, but in this case, reviews or textbooks can be cited for the relevant knowledge, but they do not need to be repeated in the manuscript.

Authors: We thank the reviewer for the comment. Considering this comment, we moved to the supplementary material the description regarding "Viruses Microbiology". We believe that having this supporting information at hand could be helpful for many readers.

Reviewer: 2. For the "Synthetic sequence benchmark" a repeat length of 5,000 has been simulated. Why has this length been chosen, is it representative of inverted repeats in viruses?

Authors: This value was selected since we think it represents a viral sequence size, given that the median sequence length of the viral genomes in the dataset used is 9,836 bases. Therefore, having a 5,000 sequence repeat would mean that the total synthetic sequence size is 10,000 bases, which is very close to the median value of the sequence length.
We added this information to the article.

Text added to the manuscript: To benchmark the capability of different programs to quantify IRs accurately, we created a genomic sequence of 10,000 nucleotides in which the last 5,000 were inverted repeats of the first 5,000. This size was chosen since the median size of the viral genomes is 9,836 bases, which is close to the total size of the synthetic sequence generated.

Reviewer: 3. In the answers, the authors wrote "One of the main goals of our manuscript was to identify and quantify inverted repeats abundance in viral genomes." However this goes is not mentioned under "Key Points" and is also only a minor point in the abstract. It would help the readers of the manuscript if the main goals of the manuscript were clearly described in the abstract and in the "Key points".

Authors: We thank the reviewer for the comment. We revised the abstract to reinforce this idea, as shown below. Furthermore, we have included as one of the key points:
"We identify and quantify inverted repeats abundance in viral genomes."

Text added to the manuscript:This work provides a comprehensive landscape of the viral genome's complexity (or quantity of information), identifying the most redundant and complex groups regarding

their genome sequence while providing their distribution and characteristics at a large and local scale. Moreover, we identify and quantify inverted repeats abundance in viral genomes.

Reviewer: 4. I previously suggested to analyze the relationship of sequence length and complexity, which would contribute to the first key point ("We provide a comprehensive landscape of the viral genomes complexity"). Although it is possible to browse all this information, this relationship could be more described in the paper.

Authors: We thank the reviewer for the comment and appreciate the importance of analysing the relationship between sequence length and complexity, which we believe to be well explored throughout the paper.
Besides the text (pages 7-9) and Figures 3 and 4, we specifically illustrate the correlation between size and complexity through a scatter plot (Figure 6B) and discuss it further: "Analyzing the sequence length projections (Figure 6 B), it is evident that there is a logarithmic downtrend of the NC with the increase in sequence length. Thus, although longer sequences have, on average, greater complexity (absolute quantities), they have higher redundancy, which the data compressor takes advantage of to perform a better compression. "

Close
