# Supplementary material for: The complexity landscape of viral genomes
Source: Gigascience. 2022 Aug 11;11:giac079. doi: 10.1093/gigascience/giac079 (PMC9366995; doi:10.1093/gigascience/giac079)
Supplement: giac079_Response_to_Reviewer_Comments_Revision_2 [file giac079_response_to_reviewer_comments_revision_2.pdf]

Author's Response To Reviewer Comments

Close

We greatly appreciate the opportunity given to review our manuscript. We hope we have been able to address the editor's concerns and that our manuscript is now suitable for publication in GigaScience.

Close
