# Supplementary material for: The complexity landscape of viral genomes
Source: Gigascience. 2022 Aug 11;11:giac079. doi: 10.1093/gigascience/giac079 (PMC9366995; doi:10.1093/gigascience/giac079)
Supplement: giac079_Reviewer_1_Report_Original_Submission — Anne Kupczok -- 4/10/2022 Reviewed [file giac079_reviewer_1_report_original_submission.pdf]

Reviewer Report

Title: The complexity landscape of viral genomes

Version: Original Submission   Date: 4/10/2022

Reviewer name: Anne Kupczok

Reviewer Comments to Author:

This manuscript presents a complexity analysis of virus genomes. Due to the fast evolution of viruses, alternative measures for comparing genomes are of interest. Nevertheless the authors could improve the presentation of the biological insights gained with this new approach.
It would be of interest to the reader which previous conclusions were drawn from the complexity approach. Although references are mentioned (7-13), this section is very short in the introduction.
The paper is very narrative and contains too many background in details in many places. This distracts the reader and impedes the flow of the paper. I would suggest to shorten it substantially for conciseness. E.g. the section "Viruses Microbiology" is mainly text book knowledge, only mention what is important for the manuscript; the section "Kolmogorov Complexity and Data Compression" can be shortened; only describe the approaches in "Classification" that are used in the paper. The paper also contains repetitions, e.g., that classes with less than four samples are discarded is mentioned twice in "Viral Classification".
Furthermore the manuscript often contains announcements which can be dropped, e.g., the last paragraph of the introduction or the first sentence of the Methods.
The statement "an organism with a genome high in GC-content is rich in energy and more prone to mutation" is unclear.
The "Synthetic sequence benchmark" section is not related to virus genomes. The authors simulate long inverted repeats of 5000nt, it is thus unclear how the results are relevant for the viral complexity analysis.
The authors find differences in complexity for different viral groups and mention that this is related to sequence length. Although this is expected, I think that it is worthwhile to describe this relationship more deeply. In Fig. 4 only one sequence length of each viral group is shown. This is misleading, since viruses inside a group can have a wide distribution of sequence lengths. To find genomes with interesting complexity patterns, it would instead be interesting to look at the relationship of length and complexity more in detail within each group.
By definition, phylogenies display the evolutionary relationships among organisms. I am not convinced that the NC measure used here to estimate trees, does indeed aim to reflect evolutionary relationships. Instead it aims to show similarities and I thus think that the trees shown are rather clustering trees instead of phylogenetic trees.
In the viral classification section it is unclear at which level the 80-20 train test split was performed. Randomly choosing genomes from the set before dereplicating them can be misleading since highly similar genomes can be in the test and training data set.
The focus on inverted repeats is not completely clear. Are they detected in the viruses known to have

ITRs?
The manuscripts presents a Discussion section. Nevertheless the Results section already contains lots of discussion. A "Results and Discussion" section might be more appropriate.
The paper is accompanied by a website. Although visually appealing, the precise usage of the website is unclear. There is no search function if the user would be interested in a particular genome. Inside a family, one can see the tree with virus names but there is no link to the genomes that went into the analysis.
Fig. 1 The letters in the figure are not matching the letters in the legend.

Methods

Are the methods appropriate to the aims of the study, are they well described, and are necessary controls included? Choose an item.

Conclusions

Are the conclusions adequately supported by the data shown? Choose an item.

Reporting Standards

Does the manuscript adhere to the journal's guidelines on minimum standards of reporting? Choose an item.

Choose an item.

Statistics

Are you able to assess all statistics in the manuscript, including the appropriateness of statistical tests used? Choose an item.

Quality of Written English

Please indicate the quality of language in the manuscript: Choose an item.

Declaration of Competing Interests

Please complete a declaration of competing interests, considering the following questions:

- Have you in the past five years received reimbursements, fees, funding, or salary from an organisation that may in any way gain or lose financially from the publication of this manuscript, either now or in the future?
- Do you hold any stocks or shares in an organisation that may in any way gain or lose financially from the publication of this manuscript, either now or in the future?
- Do you hold or are you currently applying for any patents relating to the content of the manuscript?

- Have you received reimbursements, fees, funding, or salary from an organization that holds or has applied for patents relating to the content of the manuscript?
- Do you have any other financial competing interests?
- Do you have any non-financial competing interests in relation to this paper?

If you can answer no to all of the above, write 'I declare that I have no competing interests' below. If your reply is yes to any, please give details below.

I declare that I have no competing interests

I agree to the open peer review policy of the journal. I understand that my name will be included on my report to the authors and, if the manuscript is accepted for publication, my named report including any attachments I upload will be posted on the website along with the authors' responses. I agree for my report to be made available under an Open Access Creative Commons CC-BY license (http://creativecommons.org/licenses/by/4.0/). I understand that any comments which I do not wish to be included in my named report can be included as confidential comments to the editors, which will not be published.

Choose an item.

To further support our reviewers, we have joined with Publons, where you can gain additional credit to further highlight your hard work (see: https://publons.com/journal/530/gigascience). On publication of this paper, your review will be automatically added to Publons, you can then choose whether or not to claim your Publons credit. I understand this statement.

Yes Choose an item.
