# Supplementary material for: The complexity landscape of viral genomes
Source: Gigascience. 2022 Aug 11;11:giac079. doi: 10.1093/gigascience/giac079 (PMC9366995; doi:10.1093/gigascience/giac079)
Supplement: giac079_Reviewer_1_Report_Revision_1 — Anne Kupczok -- 6/6/2022 Reviewed [file giac079_reviewer_1_report_revision_1.pdf]

Reviewer Report

Title: The complexity landscape of viral genomes

Version: Revision 1 **Date:** 6/6/2022

Reviewer name: Anne Kupczok

Reviewer Comments to Author:

I thank the authors for taking all my comments into consideration. Unfortunately I find that only a subset of them are addressed in the current version of the manuscript. My main concern is that the main point of the manuscript is not clearly elaborated in the paper. The abstract, Key points, and the main message in the Results and Discussion should be consistent.

1. I previously noted that the paper is very narrative and contains too many background details. As I do not have a version with tracked changes, I do not see if it has been shortened as some points. However, I notice that it still contains textbook knowledge, e.g., in the "Viruses Microbiology" section. I understand that this is an interdisciplinary manuscript, but in this case, reviews or textbooks can be cited for the relevant knowledge, but they do not need to be repeated in the manuscript.
2. For the "Synthetic sequence benchmark" a repeat length of 5,000 has been simulated. Why has this length been chosen, is it representative of inverted repeats in viruses?
3. In the answers, the authors wrote "One of the main goals of our manuscript was to identify and quantify inverted repeats abundance in viral genomes." However this goes is not mentioned under "Key Points" and is also only a minor point in the abstract. It would help the readers of the manuscript if the main goals of the manuscript were clearly described in the abstract and in the "Key points".
4. I previously suggested to analyze the relationship of sequence length and complexity, which would contribute to the first key point ("We provide a comprehensive landscape of the viral genomes complexity"). Although it is possible to browse all this information, this relationship could be more described in the paper.

Methods

Are the methods appropriate to the aims of the study, are they well described, and are necessary controls included? Choose an item.

Conclusions

Are the conclusions adequately supported by the data shown? Choose an item.

Reporting Standards

Does the manuscript adhere to the journal's guidelines on minimum standards of reporting? Choose an item.

Choose an item.

Statistics

Are you able to assess all statistics in the manuscript, including the appropriateness of statistical tests used? Choose an item.

Quality of Written English

Please indicate the quality of language in the manuscript: Choose an item.

Declaration of Competing Interests

Please complete a declaration of competing interests, considering the following questions:

- Have you in the past five years received reimbursements, fees, funding, or salary from an organisation that may in any way gain or lose financially from the publication of this manuscript, either now or in the future?
- Do you hold any stocks or shares in an organisation that may in any way gain or lose financially from the publication of this manuscript, either now or in the future?
- Do you hold or are you currently applying for any patents relating to the content of the manuscript?
- Have you received reimbursements, fees, funding, or salary from an organization that holds or has applied for patents relating to the content of the manuscript?
- Do you have any other financial competing interests?
- Do you have any non-financial competing interests in relation to this paper?

If you can answer no to all of the above, write 'I declare that I have no competing interests' below. If your reply is yes to any, please give details below.

I declare that I have no competing interests

I agree to the open peer review policy of the journal. I understand that my name will be included on my report to the authors and, if the manuscript is accepted for publication, my named report including any attachments I upload will be posted on the website along with the authors' responses. I agree for my report to be made available under an Open Access Creative Commons CC-BY license (http://creativecommons.org/licenses/by/4.0/). I understand that any comments which I do not wish to be included in my named report can be included as confidential comments to the editors, which will not be published.

Choose an item.

To further support our reviewers, we have joined with Publons, where you can gain additional credit to further highlight your hard work (see: https://publons.com/journal/530/gigascience). On publication of this paper, your review will be automatically added to Publons, you can then choose whether or not to claim your Publons credit. I understand this statement.

Yes Choose an item.
