# Supplementary material for: The complexity landscape of viral genomes
Source: Gigascience. 2022 Aug 11;11:giac079. doi: 10.1093/gigascience/giac079 (PMC9366995; doi:10.1093/gigascience/giac079)
Supplement: giac079_Reviewer_2_Report_Original_Submission — Kirill Kryukov, Ph.D. -- 4/12/2022 Reviewed [file giac079_reviewer_2_report_original_submission.pdf]

Reviewer Report

Title: The complexity landscape of viral genomes

Version: Original Submission **Date:** 4/12/2022

Reviewer name: Kirill Kryukov, Ph.D.

Reviewer Comments to Author:

Review of the manuscript "GIGA-D-22-00044" titled "The complexity landscape of viral genomes".
In this study the authors investigate the complexity of viral genomes. I think the topic is interesting and the performed analysis is comprehensive.
Comments:
1. It's nice that PAQ8 was included. I think it would be interesting to include cmix, as it seems to generally provide a stronger compression and therefore a better approximation of Kolmogorov's complexity.
2. P6. "Currently, the state-of-the-art genomic compressors apply statistical and algorithmic model mixtures combined with arithmetic encoding." - This sentence seems to use "state-of-the-art" in a narrow sense, referring to just compression strength. Other kinds of state-of-the-art compressors exist, for example those that prioritize a combination of compression strength and decompression speed, such as NAF (https://github.com/KirillKryukov/naf , https://doi.org/10.1093/bioinformatics/btz144). NAF uses a dictionary-based compression. Perhaps this part can modified to make it more clear that it discusses compressors providing the best compression strength.
3. P6. "The best compression ratio performance for various genomic sequences is provided by XM [82], Jarvis [83], and Geco3 [14]." - As shown in Sequence Compression Benchmark, cmix provides stronger compression than these compressors. (http://kirr.dyndns.org/sequence-compression-benchmark/ , https://doi.org/10.1093/gigascience/giaa072).
4. P6. "An efficient compressor, C(x), provides an upper bound approximation for the Kolmogorov complexity" - In the following text and formula, C(x) seems to be used as a number. Therefore the sentence is confusing. It seems that C(x) is not "an afficient compressor", but rather, size of data compressed with an efficient compressor.
5. P6. "K(x) < C(x) <= |x|" - I think pigeonhole principle implies that you can't design an efficient compressor such that C(x) is always <= |x|. For some inputs C(x) must be greater than |x|. Also, by chance you may produce C(x) that is identical to K(x), therefore "K(x) <= C(x)" would probably be more accurate.
6. P6. "The normalized version, known as the Normalized Compression (NC)" - Normalized Compression implies the process or method of compression. but here it is used to represent "Normalized Compressed Size", or something like that. This confusing terminology does not help the reader. It seems that NC here refers to the inverse of Compression Ratio (Original data size / Size of compressed data), which would be more natural and easy to understand. On P8 you write "We evaluated the frequency where each level yielded the lowest NC (provided the best compression for a given sequence; Figure 2 A)" - Lower NC means higher compression - which is counterintuitive. Higher "compression" should correspond to

stronger compression, which would be the case if Compression Ratio was used as a measure instead.
7. P7. "There was a need to determine the sequences with the highest normalized compression capacity (NCC) in some cases. When the compressor was only using the subprogram IR2, NCC was computed as NCCIR2 (x) = 1 - NCIR2 ." - The purpose of this derived measure is not clear. NC value is confusing enough by itself already, why 1 - NC is needed? Since NCC only depends on NC, why not simply use NC by itself? In paper you then use both NC and NCC side by side, which have the opposite scales: Stronger compression gives smaller NC, but larger NCC.
8. P7. "The dataset is composed of 12,163 complete reference genomes from 9,605 viral taxa retrieved from NCBI database on 22 of January 2021 using the following url https://tinyurl.com/ncbidtbs." - Please include the actual url in the methods section, rather than depending on tinyurl.
9. P7. "Secondly, a filter was applied to remove outlier sequences. Specifically, after computing all sequences' length, GC-Content, and Normalized Complexities, sequences whose measure fell outside 3 (approximately 0.03% of all sequences) of any measure were removed. After filtering, 6,091 of the initial 12,163 sequences were kept." - This seems to be a bit of circular logic, regarding classification accuracy. When designing an automatic virus genome classifier, arbitrary precision can be achieved by removing various amounts of outlier sequences beforehand.
10. P11. "Furthermore, we performed classification using seven different features: sequence length (SL), GC-content (GC), the Normalized Compression (NC) values for the best performing model, and the NC of the same model with IR configuration to 0, 1 and 2." - It's unfortunate that sequence length was included among the features used for classification, as this significantly reduces the value of this method. In actual analysis of viral sequences (both environmental, and integrated in genomes), we often don't know the full length of the original viral genome, but only see a DNA or RNA fragment. Designing a classifier that does not need sequence length would be potentially much more useful in practice.
11. "As far as we know, this is the first attempt at performing this type of reference-free classification. As such, for comparison purposes, we assessed the outcomes obtained using a random classifier." - There are many studies on alignment-free sequence comparison and classification. Some examples specifically for viruses: https://doi.org/10.1016/j.meegid.2021.105106 , https://doi.org/10.1016/j.csbj.2021.10.029 , https://doi.org/10.1038/srep40712 , https://doi.org/10.1515/sagmb-2018-0004 .
12. P12. "Figure 6. Phylogenetic tree showing average NC of each viral group (A), and the normalized compression capacity (NCC) (B)." - What Figure 6 shows is more accurately described as a cladogram, not a phylogenetic tree. In a phylogenetic tree, branch lengths are proportional to the extent of difference between sequences, or to time passed since divergence. However in Figure 6 all branches are of the same length, implying that probably simply taxonomic structure is shown.
13. P12. "The usage of a specialized compressor is crucial to quantify the complexity present in a genome accurately. Specialized compressors outperform general-purpose compressors because they take into account the intrinsic nature of the data." - General-purpose cmix currently outperforms specialized compressors in the Sequence Compression Benchmark.
14. P3. "Using a state-of-the-art genomic compressor on an extensive viral genomes database, we show that dsDNA viruses are on average the most redundant viruses while ssDNA viruses are the lowest." - Maybe replace "lowest" with "least", or otherwise rephrase.
15. P4. "their understanding is still relatively limited" => our understanding of viruses is still relatively

limited".
16. Overall, the text is unnecessarily complicated. Many parts can be simplified and described in more simple terms. E.g. P13 "a lower NC and abundance of inversions present in herpesvirus" => "a higher compressibility and abundance of inversions present in herpesvirus".

Methods

Are the methods appropriate to the aims of the study, are they well described, and are necessary controls included? Choose an item.

Conclusions

Are the conclusions adequately supported by the data shown? Choose an item.

Reporting Standards

Does the manuscript adhere to the journal's guidelines on minimum standards of reporting? Choose an item.

Choose an item.

Statistics

Are you able to assess all statistics in the manuscript, including the appropriateness of statistical tests used? Choose an item.

Quality of Written English

Please indicate the quality of language in the manuscript: Choose an item.

Declaration of Competing Interests

Please complete a declaration of competing interests, considering the following questions:

- Have you in the past five years received reimbursements, fees, funding, or salary from an organisation that may in any way gain or lose financially from the publication of this manuscript, either now or in the future?
- Do you hold any stocks or shares in an organisation that may in any way gain or lose financially from the publication of this manuscript, either now or in the future?
- Do you hold or are you currently applying for any patents relating to the content of the manuscript?
- Have you received reimbursements, fees, funding, or salary from an organization that holds or has applied for patents relating to the content of the manuscript?
- Do you have any other financial competing interests?

- Do you have any non-financial competing interests in relation to this paper?

If you can answer no to all of the above, write 'I declare that I have no competing interests' below. If your reply is yes to any, please give details below.

I declare that I have no competing interests

I agree to the open peer review policy of the journal. I understand that my name will be included on my report to the authors and, if the manuscript is accepted for publication, my named report including any attachments I upload will be posted on the website along with the authors' responses. I agree for my report to be made available under an Open Access Creative Commons CC-BY license (http://creativecommons.org/licenses/by/4.0/). I understand that any comments which I do not wish to be included in my named report can be included as confidential comments to the editors, which will not be published.

Choose an item.

To further support our reviewers, we have joined with Publons, where you can gain additional credit to further highlight your hard work (see: https://publons.com/journal/530/gigascience). On publication of this paper, your review will be automatically added to Publons, you can then choose whether or not to claim your Publons credit. I understand this statement.

Yes Choose an item.
