# Supplementary material for: The complexity landscape of viral genomes
Source: Gigascience. 2022 Aug 11;11:giac079. doi: 10.1093/gigascience/giac079 (PMC9366995; doi:10.1093/gigascience/giac079)
Supplement: giac079_Supplemental_File [file giac079_supplemental_file.pdf]

Supplementary Material

Here, we depict the supplementary material of the article. The supplementary material is described in five main sections: Compression Model Benchmark, Viral Genome Analysis, Classification, Software and Hardware recommendations, and Reproducibility. The Compression Model Benchmark, Viral Genome Analysis and Classification sections have auxiliary material to their corresponding sections of the main article. On the other hand, the Software and Hardware recommendations section defines minimum requirements, and the Reproducibility section describes how to reproduce the results obtained in this article.

Viruses Microbiology Additional Information.

Viruses can exist outside of their host in the form of independent particles named virions composed of the genetic material (DNA or RNA) enclosed by the capsid. This protein shell protects the viral genome, and at the same time, it is extracellular and promotes its entry into the host cells [1].

Most of the viruses possess capsids with helical (Figure S1 A) or icosahedral (Figure S1 B) arrangements [2, 3]. Different viruses, like bacteriophages, have developed other structures composed of elongated capsids attached to a cylindrical tailed sheet (Figure S1 C) [4]. Others have an outer lipid bilayer named viral envelope (Figure S1 D), which is constituted by a modified form of the host's cell membranes. Viroids have naked genomes without any protective layer. Like viruses, they use the host's machinery to replicate, but their genomes do not encode proteins [5]. Furthermore, some viruses are dependent on another virus species in the host cell to be transmitted to new cells. They were named 'satellites' and may represent evolutionary intermediates of viroids and viruses [6, 7]. Regarding mutability, the viral and viroid realm is the most mutable [8] of the realms.

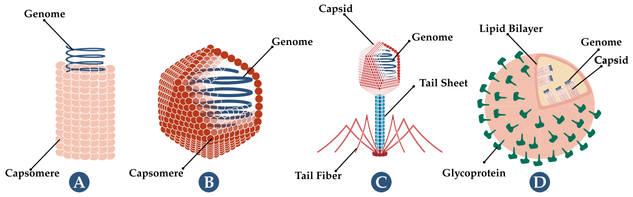

Figure S1. Illustrations of types of virus morphology. Virus (**A**) is a helical virus, where the capsoid has a helical shape that envelops the genomic material, virus (**B**) is icosahedral following cubic symmetry, (**C**) depicts a complex virus, namely a bacteriophage with a prolate capsid protecting the genomic material, and (**D**) is virus covered by a viral envelop.

Viral genomes can be of double-stranded DNA (dsDNA), single-stranded DNA (ssDNA), double-stranded RNA (dsRNA) or single-stranded RNA (ssRNA) nature, being linear or circular molecules [9]. The ssRNA viruses can be further classified as positive- or negative-ssRNA, depending on the sense of their RNA strand. These features determine the viral replication and mRNA synthesis pathways. For instance, (+)-ssRNA is directly translated into proteins by the host cell's ribosomes, acting as mRNA. On the other hand, (-)-ssRNA needs to be converted to a (+)-ssRNA by an RNA-dependent RNA polymerase (RdRp) before translation. RdRp also transcribes dsRNA to mRNA (using the negative strand as a template), and it is indispensable for the replication of RNA viral genomes. Finally, ssDNA and dsDNA usually use the host's DNA-dependent RNA polymerase to form mRNA. However, before this process, ssDNA is converted to a dsDNA by a DNA polymerase upon cell invasion [10], which is also the enzyme involved in the replication of DNA viruses. The RdRps have a high error rate due to their low proofreading activity and, therefore, replication of RNA viruses is much more prone to mutation than that of DNA viruses [11].

Data compressors and Level selection benchmark

Herein, it is depicted the supplementary material to the Data compressors and Level selection benchmark.

Figure S2 shows the compression-time and compression-ratio of various Human Herpesviruses genome sequences between cmix and GeCo3 compression.

Table S1 describes the parameters used in the six custom build levels. The flag *"tm"* is the template of a target context model, the flag *"lr"* defines the learning rate, and the flag *"hs"* defines the number of hidden nodes for the neural network.

Table S1. Depiction of the parameters used in the six custom levels.

Level	Values
1	-tm 1:1:0:0:0.7/0:0:0 -tm 12:20:1:1:0.97/1:1:0.97
2	-tm 1:1:0:0:0.7/0:0:0 -tm 12:20:1:1:0.97/2:1:0.97
3	-tm 1:1:0:0:0.7/0:0:0 -tm 12:50:1:1:0.97/0:0:0.97
4	-tm 1:1:0:0:0.7/0:0:0 -tm 12:20:1:1:0.97/0:0:0.97 -lr 0.05 -hs 40
5	-tm 1:1:0:0:0.7/0:0:0 -tm 12:20:1:1:0.97/0:0:0.97 -lr 0.15 -hs 40
6	-tm 1:1:0:0:0.7/0:0:0 -tm 12:20:1:1:0.97/0:0:0.97 -lr 0.3 -hs 40

Table S2 describes the parameters used in the template of a target context model. The template has the flag *"tm"* and follows the

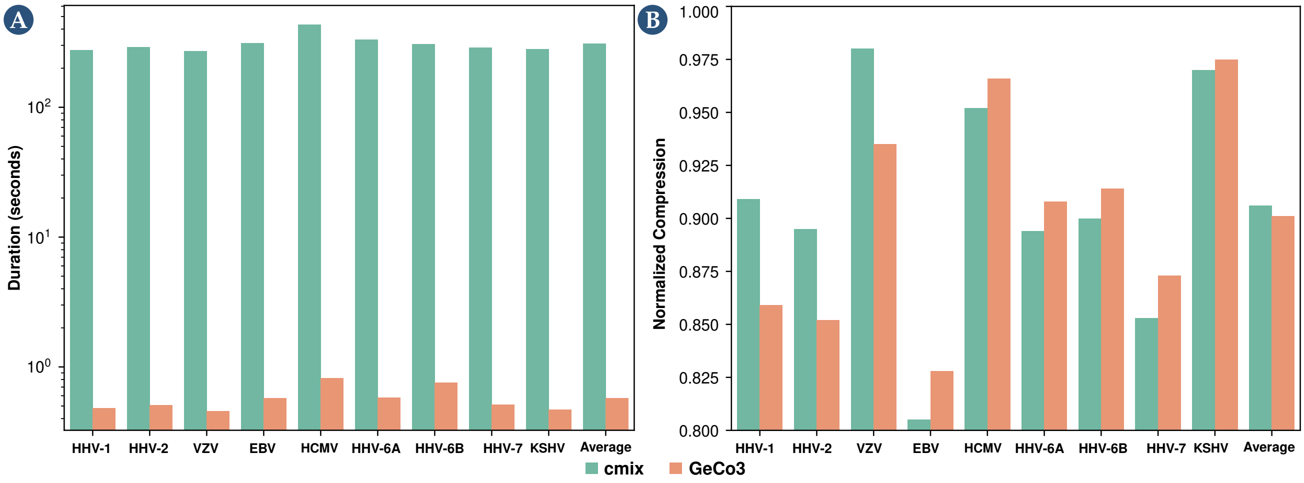

Figure S2. Comparison between cmix and GeCo3 when applied to various Human Herpesviruses regarding computational time and compression ratio obtained (NC).

model "[NB_C]:[NB_D]:[NB_I]:[NB_H]:[NB_G]/[NB_S]:[NB_E]:[NB_A]".

Table S2. Depiction of the parameters used in the template of a target context model.

Parameter	Values	Description
**[NB_C]**	integer [1;20]	Order size of the regular context model. The higher the value of the regular context model, the more RAM it uses but, usually, are related to a better compression score.
**[NB_D]**	integer [1;5000]	Denominator to build alpha, which is a parameter estimator. Alpha is given by 1/[NB_D]. Higher values are usually used with higher [NB_C] and are related to sure bets. When [NB_D] is one, the probabilities assume a Laplacian distribution.
**[NB_I]**	integer {0,1,2}	Number to define if a sub-program that addresses the specific properties of DNA sequences (inverted repeats) is used or not. The number 2 turns ON this sub-program without the regular context model (only inverted repeats). The number 1 turns ON the sub-program using at the same time the regular context model. The number 0 does not contemplate its use (inverted repeats OFF). This sub-program increases the necessary time to compress, but it does not affect the RAM.
**[NB_H]**	integer [1;254]	Size of the cache-hash for deeper context models, namely for [NB_C] >14. When the [NB_C] <= 14 use, for example, 1 as a default. The RAM is highly dependent of this value (higher value stand for higher RAM).
**[NB_G]**	real [0;1)	Real number to define gamma. This value represents the decaying forgetting factor of the regular context model in the definition.
**[NB_S]**	integer [0;20]	The maximum number of editions allowed to use a substitutional tolerant model with the same memory model of the regular context model with an order size equal to [NB_C]. The value 0 stands for turning the tolerant context model off. When the model is on, it pauses when the number of editions is higher than [NB_C]. When it is turned on when a full match of size [NB_C] is seen again, this is a probabilistic-algorithmic model advantageous to handle the high substitutional nature of genomic sequences. When [NB_S] >0, the compressor used more processing time but used the same RAM and, usually, achieved a substantial higher compression ratio. The impact of this model is usually only noticed for [NB_C] >= 14.
**[NB_E]**	integer [1;5000]	Denominator to build alpha for substitutional tolerant context model. It is analogous to [NB_D]. However, it is only used in the probabilistic model for computing the statistics of the substitutional tolerant context model.
**[NB_A]**	real [0;1)	Real number to define gamma. This value represents the decaying forgetting factor of the substitutional tolerant context model in the definition. Its definition and use are analogous to [NB_G].

Viral Genome Analysis

We present the supplementary material discussed in the Viral Genome Analysis of this main article. Table S3 depicts the genome types ordered by the highest normalized compression (NC), normalized compression capacity (*NCC*) and *difference*. *NCC* is computed by $NCC = 1 - NC_{IR_2} > 0$, and the difference as $difference = NC_{IR_0} - NC_{IR_1}$. Furthermore, the Table shows the genomes' average Sequence Length (SL) and GC-Content (GC).

Table S4 depicts the top Normalized Compression (NC) values by taxonomic group. Three main groups separate the Table. The first represents the highest 10 NC values using standard settings NC (best performing model); the second group shows the top 10 lowest NC values obtained using the IR_2 subprogram. Finally, the third group shows the top 10 highest values of the difference between NC using IR_0 and IR_1 subprograms.

Tables S5,S6,and S7 organize the top taxa (by taxonomic group) regarding their normalized compression (NC), normalized compression capacity (*NCC*) and *difference*. The tables also shows the genomes' average Sequence Length and GC-Content.

Finally, Figure S3 depicts the cladogram with average NC *difference* ($NC_{IR_0} - NC_{IR_1} > 0$) for each viral taxonomic group up to the viral genus. The colour red depicting the highest NC *difference*, and the blue the lowest.

Table S3. Depiction of the genome type by the highest normalized compression (NC), normalized compression capacity (*NCC*) and *difference*. *NCC* is computed by $NCC = 1 - NC_{IR_2} > 0$, and the difference as $difference = NC_{IR_0} - NC_{IR_1}$. Furthermore, the Table shows the genomes' average Sequence Length (SL) and GC-Content (GC).

**Normalized Compression**				**Inverted Repeats**				**Difference**			
**Genome**	**NC**	**SL**	**GC**	**Genome**	**NCC**	**SL**	**GC**	**Genome**	**difference**	**SL**	**GC**
**ssDNA**	1.065	3282	0.447	**dsDNA**	0.029	84721	0.485	**ssDNA**	0.006	4672	0.435
**mixedDNA**	1.050	3258	0.491	**ssDNA**	0.026	5981	0.389	**dsDNA**	0.006	80636	0.470
**dsRNA**	1.047	8377	0.456	**ssRNA**	0.015	13425	0.393	**mixedDNA**	0.002	3311	0.434
**ssRNA**	1.013	9564	0.437	**dsRNA**	0.015	19911	0.396	**dsRNA**	0.001	6186	0.431
**dsDNA**	0.977	70353	0.481					**ssRNA**	0.001	10197	0.433

Table S4. Depiction of the top NC values by taxonomic group. Three main groups separate the Table. The first represents the highest 10 NC values using standard settings NC (best performing model); the second group shows the top 10 lowest NC values obtained using the IR_2 subprogram. Finally, the third group shows the top 10 highest values of the difference between NC using IR_0 and IR_1 subprograms.

NC	Top	Realm	Kingdom	Phylum	Class	Order	Family	Genus
Best performing model	1	Ribozyviria	Shotokuvirae	Lenarviricota	Miaviricetes	Ourlivirales	Botourmiaviridae	Clostunsatellite
	2	Monodnaviria	Sangervirae	Cressdnaviricota	Arfiviricetes	Cirlivirales	Alphasatellitidae	Milvetsatellite
	3	Riboviria	Orthornavirae	Duplornaviricota	Chunqiuviricetes	Cremevirales	Tolecusatellitidae	Aumaivirus
	4	Duplodnaviria	Pararnavirae	Phixviricota	Magsaviricetes	Muvirales	Circoviridae	Virtovirus
	5	Varidnaviria	Loebvirae	Kitrinoviricota	Amabiliviricetes	Nodamuvirales	Genomoviridae	Mivedwarsatellite
	6	Adnaviria	Trapavirae	Cossaviricota	Duplopiviricetes	Wolframvirales	Nodaviridae	Babusatellite
	7	-	Heunggongvirae	Pisuviricota	Allassoviricetes	Durnavirales	Kolmioviridae	Fabenesatellite
	8	-	Bamfordvirae	Negarnaviricota	Repensiviricetes	Levivirales	Smacoviridae	Ourmiavirus
	9	-	Helvetiavirae	Artverviricota	Yunchangviricetes	Geplafuvirales	Qinviridae	Albetovirus
	10	-	Zilligvirae	Hofneiviricota	Insthoviricetes	Goujianvirales	Narnaviridae	Geminialphasatellitinae
Inverted Repeat Subprogram (NC_{IR_2})	1	Adnaviria	Loebvirae	Peploviricota	Pokkesviricetes	Imitervirales	Mimiviridae	Betaentomopoxvirus
	2	Varidnaviria	Zilligvirae	Nucleocytoviricota	Herviviricetes	Chitovirales	Rudiviridae	Oryzopoxvirus
	3	Duplodnaviria	Helvetiavirae	Hofneiviricota	Maveriviricetes	Herpesvirales	Poxviridae	Vespertilionpoxvirus
	4	Monodnaviria	Bamfordvirae	Taleaviricota	Mouviricetes	Priklausovirales	Malacoherpesviridae	Simplexvirus
	5	Riboviria	Heunggongvirae	Dividoviricota	Faserviricetes	Polivirales	Plectroviridae	Cafeteriavirus
	6	Ribozyviria	Trapavirae	Uroviricota	Tokiviricetes	Ligamenvirales	Mononiviridae	Mardivirus
	7	-	Shotokuvirae	Saleviricota	Laserviricetes	Tubulavirales	Herpesviridae	Cervidpoxvirus
	8	-	Pararnavirae	Preplasmiviricota	Megaviricetes	Halopanivirales	Lavidaviridae	Varicellovirus
	9	-	Orthornavirae	Negarnaviricota	Naldaviricetes	Pimascovirales	Bidnaviridae	Ostreavirus
	10	-	Sangervirae	Cossaviricota	Milneviricetes	Lefavirales	Polydnaviridae	Vespertiliovirus
$NC_{IR_0} - NC_{IR_1}$	1	Adnaviria	Zilligvirae	Peploviricota	Herviviricetes	Herpesvirales	Malacoherpesviridae	Mardivirus
	2	Varidnaviria	Trapavirae	Taleaviricota	Mouviricetes	Polivirales	Herpesviridae	Ostreavirus
	3	Duplodnaviria	Bamfordvirae	Nucleocytoviricota	Tokiviricetes	Chitovirales	Rudiviridae	Iltovirus
	4	Monodnaviria	Heunggongvirae	Saleviricota	Pokkesviricetes	Ligamenvirales	Bidnaviridae	Leporipoxvirus
	5	Ribozyviria	Shotokuvirae	Cossaviricota	Quintoviricetes	Piccovirales	Poxviridae	Simplexvirus
	6	Riboviria	Helvetiavirae	Dividoviricota	Huolimaviricetes	Haloruvirales	Polydnaviridae	Varicellovirus
	7	-	Loebvirae	Hofneiviricota	Megaviricetes	Cirlivirales	Ampullaviridae	Aurivirus
	8	-	Sangervirae	Cressdnaviricota	Laserviricetes	Pimascovirales	Nudiviridae	Oryzopoxvirus
	9	-	Orthornavirae	Preplasmiviricota	Arfiviricetes	Algavirales	Parvoviridae	Vespertilionpoxvirus
	10	-	Pararnavirae	Duplornaviricota	Faserviricetes	Kalamavirales	Ascoviridae	Entnonagintavirus

Table S5. Depiction of the taxonomic groups with the highest NC values. The Table shows each group's average Normalized Compression, Sequence Length and GC-Content.

Taxonomic Group	Taxonomic Name	Normalized Compression	Sequence Length	GC-Content
**Super-Realm**	Viruses	1.007	36067	0.460
**Realm**	Ribozyviria	1.080	1682	0.588
	Monodnaviria	1.046	4380	0.450
	Riboviria	1.016	9332	0.438
	Duplodnaviria	0.972	78102	0.500
	Varidnaviria	0.957	109560	0.448
	Adnaviria	0.948	33068	0.353
**Kingdom**	Shotokuvirae	1.049	4200	0.447
	Sangervirae	1.026	5518	0.435
	Orthornavirae	1.018	9472	0.438
	Pararnavirae	0.995	7787	0.433
	Loebvirae	0.994	7332	0.483
	Trapavirae	0.993	10151	0.564
	Heunggongvirae	0.972	78102	0.500
	Bamfordvirae	0.957	112955	0.441
	Helvetiavirae	0.949	24833	0.665
	Zilligvirae	0.948	33068	0.353
**Phylum**	Lenarviricota	1.094	2654	0.476
	Cressdnaviricota	1.067	3134	0.453
	Duplornaviricota	1.045	9418	0.456
	Phixviricota	1.026	5518	0.435
	Kitrinoviricota	1.018	8548	0.474
	Cossaviricota	1.013	6260	0.436
	Pisuviricota	1.012	10580	0.442
	Negarnaviricota	1.012	9620	0.397
	Artverviricota	0.995	7787	0.433
	Hofneiviricota	0.994	7332	0.483
**Class**	Miaviricetes	1.151	1792	0.514
	Arfiviricetes	1.085	2557	0.464
	Chunqiuviricetes	1.075	3870	0.503
	Magsaviricetes	1.073	3730	0.513
	Amabiliviricetes	1.072	2703	0.586
	Duplopiviricetes	1.066	3298	0.467
	Allassoviricetes	1.063	3753	0.493
	Repensiviricetes	1.063	3281	0.451
	Yunchangviricetes	1.061	3987	0.358
	Insthoviricetes	1.054	5784	0.425
**Order**	Ourlivirales	1.151	1792	0.514
	Cirlivirales	1.103	1864	0.471
	Cremevirales	1.078	2572	0.478
	Muvirales	1.075	3870	0.503
	Nodamuvirales	1.073	3730	0.513
	Wolframvirales	1.072	2703	0.586
	Durnavirales	1.066	3298	0.467
	Levivirales	1.063	3753	0.493
	Geplafuvirales	1.063	3281	0.451
	Goujianvirales	1.061	3987	0.358
**Family**	Botourmiaviridae	1.151	1792	0.514
	Alphasatellitidae	1.143	1296	0.418
	Tolecusatellitidae	1.116	1347	0.389
	Circoviridae	1.103	1864	0.471
	Genomoviridae	1.096	2201	0.517
	Nodaviridae	1.080	3368	0.514
	Kolmioviridae	1.080	1682	0.588
	Smacoviridae	1.078	2572	0.478
	Qinviridae	1.075	3870	0.503
	Narnaviridae	1.072	2703	0.586
**Genus**	Clostunsatellite	1.192	1008	0.423
	Milvetsatellite	1.186	1022	0.402
	Aumaivirus	1.185	1168	0.510
	Virtovirus	1.180	1150	0.442
	Mivedwarsatellite	1.179	1014	0.402
	Babusatellite	1.178	1104	0.437
	Fabenesatellite	1.176	1007	0.385
	Ourmiavirus	1.167	1605	0.519
	Albetovirus	1.167	1221	0.426
	Geminialphasatellitinae	1.131	1370	0.418

Table S6. Depiction of the taxonomic groups with the highest normalized compression capacity (*NCC*) using only the inverted repeats subprogram IR_2. The top results were obtained by $NCC = 1 - NC_{IR_2} > 0$. Besides the normalized compression capacity, the Table shows each group's average Sequence Length and GC-Content.

Group	Taxonomic Group	$NCC = 1 - NC_{IR_2} > 0$	Sequence Legth	GC-Content
**Super-Realm**	Viruses	0.026	66796	0.462
**Realm**	Adnaviria	0.052	33068	0.353
	Varidnaviria	0.038	110591	0.447
	Duplodnaviria	0.028	82677	0.499
	Monodnaviria	0.022	6958	0.399
	Riboviria	0.015	13682	0.391
**Kingdom**	Loebvirae	0.053	7371	0.385
	Zilligvirae	0.052	33068	0.353
	Helvetiavirae	0.050	24833	0.665
	Bamfordvirae	0.038	114079	0.440
	Heunggongvirae	0.028	82677	0.499
	Trapavirae	0.021	12225	0.577
	Shotokuvirae	0.016	6184	0.378
	Pararnavirae	0.016	9610	0.378
	Orthornavirae	0.015	14012	0.393
	Sangervirae	0.005	4421	0.321
**Phylum**	Peploviricota	0.068	168832	0.534
	Nucleocytoviricota	0.063	210417	0.389
	Hofneiviricota	0.053	7371	0.385
	Taleaviricota	0.052	33068	0.353
	Dividoviricota	0.050	24833	0.665
	Uroviricota	0.026	79042	0.497
	Saleviricota	0.021	12225	0.577
	Preplasmiviricota	0.017	32147	0.483
	Negarnaviricota	0.016	12180	0.376
	Cossaviricota	0.016	6128	0.378
**Class**	Pokkesviricetes	0.072	190762	0.365
	Herviviricetes	0.068	168832	0.534
	Maveriviricetes	0.066	18227	0.290
	Mouviricetes	0.066	8377	0.299
	Faserviricetes	0.053	7371	0.385
	Tokiviricetes	0.052	33068	0.353
	Laserviricetes	0.050	24833	0.665
	Megaviricetes	0.046	248459	0.436
	Naldaviricetes	0.040	132022	0.410
	Milneviricetes	0.029	11079	0.349
**Order**	Imitervirales	0.109	899501	0.256
	Chitovirales	0.091	193551	0.356
	Herpesvirales	0.068	168832	0.534
	Priklausovirales	0.066	18227	0.290
	Polivirales	0.066	8377	0.299
	Ligamenvirales	0.055	34464	0.343
	Tubulavirales	0.053	7371	0.385
	Halopanivirales	0.050	24833	0.665
	Pimascovirales	0.043	162587	0.456
	Lefavirales	0.040	132022	0.410
**Family**	Mimiviridae	0.109	899501	0.256
	Rudiviridae	0.103	30804	0.299
	Poxviridae	0.091	193551	0.356
	Malacoherpesviridae	0.091	209479	0.427
	Plectroviridae	0.080	7045	0.248
	Mononiviridae	0.077	41178	0.275
	Herpesviridae	0.074	158421	0.539
	Lavidaviridae	0.066	18227	0.290
	Bidnaviridae	0.066	8377	0.299
	Polydnaviridae	0.055	306235	0.377
**Genus**	Betaentomopoxvirus	0.174	247441	0.195
	Oryzopoxvirus	0.164	185139	0.236
	Vespertilionpoxvirus	0.156	176688	0.236
	Simplexvirus	0.144	148626	0.694
	Cafeteriavirus	0.127	617453	0.233
	Mardivirus	0.121	177993	0.509
	Cervidpoxvirus	0.115	166259	0.262
	Varicellovirus	0.107	139331	0.560
	Ostreavirus	0.107	207439	0.387
	Vespertiliovirus	0.103	7970	0.228

Table S7. Depiction of the taxonomic groups with the highest difference of values between $NC_{IR_0} - NC_{IR_1}$. The Table shows each group's average *difference* = $NC_{IR_0} - NC_{IR_1}$, Sequence Length and GC-Content.

Taxonomic Group	Taxonomic Name	$NC_{IR_0} - NC_{IR_1} > 0$	Sequece Length	GC-Content
**Super-Realm**	Viruses	0.004	44293	0.451
**Realm**	Adnaviria	0.019	35299	0.322
	Varidnaviria	0.007	111364	0.443
	Duplodnaviria	0.007	78316	0.512
	Monodnaviria	0.005	5359	0.436
	Ribozyviria	0.002	1682	0.588
	Riboviria	0.001	9847	0.431
**Kingdom**	Zilligvirae	0.019	35299	0.322
	Trapavirae	0.009	16113	0.503
	Bamfordvirae	0.007	114249	0.437
	Heunggongvirae	0.007	78316	0.512
	Shotokuvirae	0.005	5124	0.434
	Helvetiavirae	0.004	27439	0.664
	Loebvirae	0.002	8519	0.453
	Sangervirae	0.001	4552	0.426
	Orthornavirae	0.001	10049	0.430
	Pararnavirae	0.001	8050	0.435
**Phylum**	Peploviricota	0.050	159507	0.557
	Taleaviricota	0.019	35299	0.322
	Nucleocytoviricota	0.013	210797	0.381
	Saleviricota	0.009	16113	0.503
	Cossaviricota	0.007	5450	0.433
	Dividoviricota	0.004	27439	0.664
	Hofneiviricota	0.002	8519	0.453
	Cressdnaviricota	0.002	4539	0.438
	Preplasmiviricota	0.002	32788	0.483
	Duplornaviricota	0.001	8140	0.389
**Class**	Herviviricetes	0.050	159507	0.557
	Mouviricetes	0.029	8377	0.299
	Tokiviricetes	0.019	35299	0.322
	Pokkesviricetes	0.017	193309	0.354
	Quintoviricetes	0.011	5164	0.446
	Huolimaviricetes	0.009	16113	0.503
	Megaviricetes	0.005	247791	0.441
	Laserviricetes	0.004	27439	0.664
	Arfiviricetes	0.004	5459	0.432
	Faserviricetes	0.002	8519	0.453
**Order**	Herpesvirales	0.050	159507	0.557
	Polivirales	0.029	8377	0.299
	Chitovirales	0.022	196072	0.341
	Ligamenvirales	0.019	35299	0.322
	Piccovirales	0.011	5164	0.446
	Haloruvirales	0.009	16113	0.503
	Cirlivirales	0.008	2114	0.476
	Pimascovirales	0.005	169619	0.458
	Algavirales	0.005	339710	0.413
	Kalamavirales	0.004	15181	0.459
**Family**	Malacoherpesviridae	0.062	209479	0.427
	Herpesviridae	0.050	155406	0.564
	Rudiviridae	0.035	30804	0.299
	Bidnaviridae	0.029	8377	0.299
	Poxviridae	0.022	196072	0.341
	Polydnaviridae	0.019	306235	0.377
	Ampullaviridae	0.012	23814	0.346
	Nudiviridae	0.012	127615	0.416
	Parvoviridae	0.011	5164	0.446
	Ascoviridae	0.010	172411	0.453
**Genus**	Mardivirus	0.103	177993	0.509
	Ostreavirus	0.072	207439	0.387
	Iltovirus	0.070	155856	0.546
	Leporipoxvirus	0.066	160815	0.415
	Simplexvirus	0.061	148626	0.694
	Varicellovirus	0.061	139331	0.560
	Aurivirus	0.052	211518	0.468
	Oryzopoxvirus	0.050	185139	0.236
	Vespertilionpoxvirus	0.046	176688	0.236
	Entnonagintavirus	0.036	29564	0.558

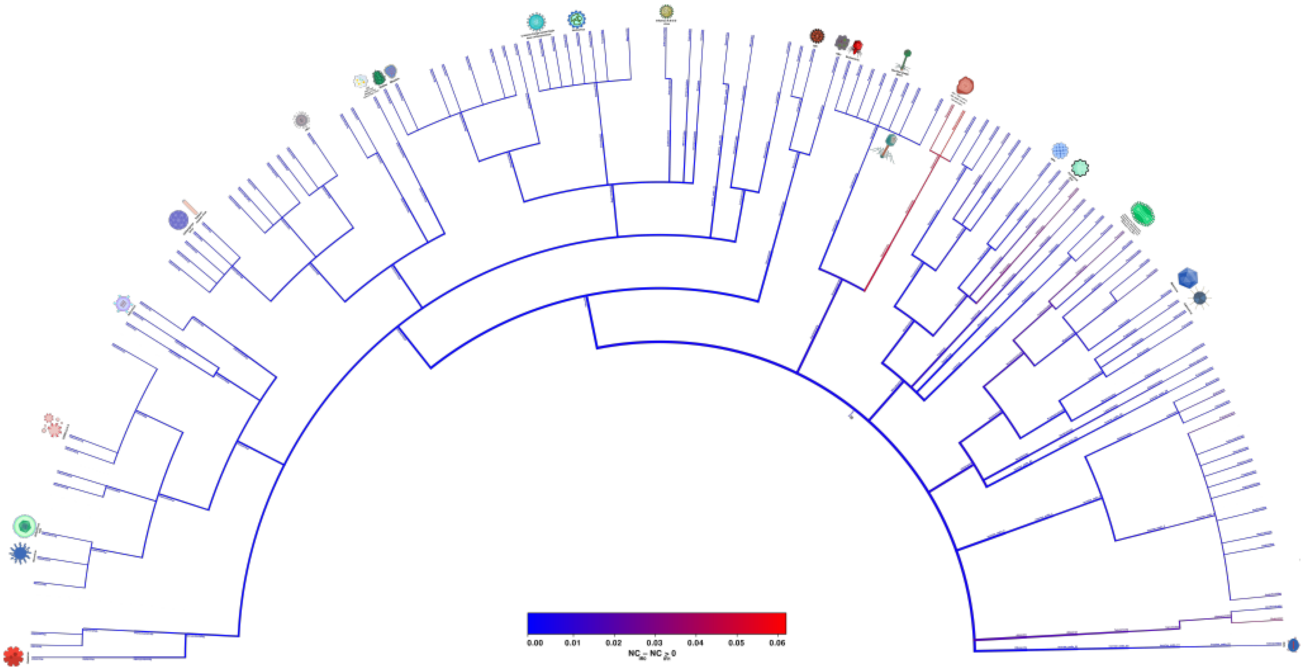

Figure S3. Cladogram showing average *difference* ($NC_{IR_0} - NC_{IR_1} > 0$).The colour red depicts the branches where on average, the genome possesses more inverted repetitions than internal repetitions (higher difference), whereas the blue colour represents the branches with fewer inverted repetitions than internal repetitions (smaller difference).

Classification

Herein, we show the supplementary classification tables that are discussed in the classification subsection of this article.

Figure S4 represents the number of samples (genome sequences) per viral genus.

Table S8 and Table S9 show the values obtained using different classifiers for accuracy and F1-score, respectively. In both cases, the XGBoost classifier had the best performance.

Table S10 displays the XGBoost classifier F1-score results when using different sets of features. With the notable exception of the type of genome classification, the best results were obtained using all features.

Table S8. Accuracy (ACC) results obtained for viral taxonomic classification tasks regarding genome type, realm, kingdom, phylum, class, order, family, and genus. The classifiers used were Linear Discriminant Analysis (LDA), Gaussian Naive Bayes (GNB), K-Nearest Neighbors (KNN), Support Vector Machine (SVM), and XGBoost classifier (XGB).

Classification	N. Classes	N. Samples	ACC_{LDA}	ACC_{GNB}	ACC_{SVM}	ACC_{KNN}	ACC_{XGB}
**Genome**	5	6089	67.32	74.14	72.41	84.4	**87.25**
**Realm**	5	5799	75.95	80.95	81.38	88.71	**92.57**
**Kingdom**	10	5788	73.49	78.76	78.41	85.49	**90.96**
**Phylum**	17	5778	61.59	56.75	55.88	71.28	**83.41**
**Class**	34	5845	51.15	52.95	47.56	63.47	**80.23**
**Order**	48	5838	48.89	55.65	48.89	60.62	**79.62**
**Family**	102	5990	36.64	43.24	27.05	42.99	**74.46**
**Genus**	360	4673	44.6	36.79	18.82	17.65	**68.71**

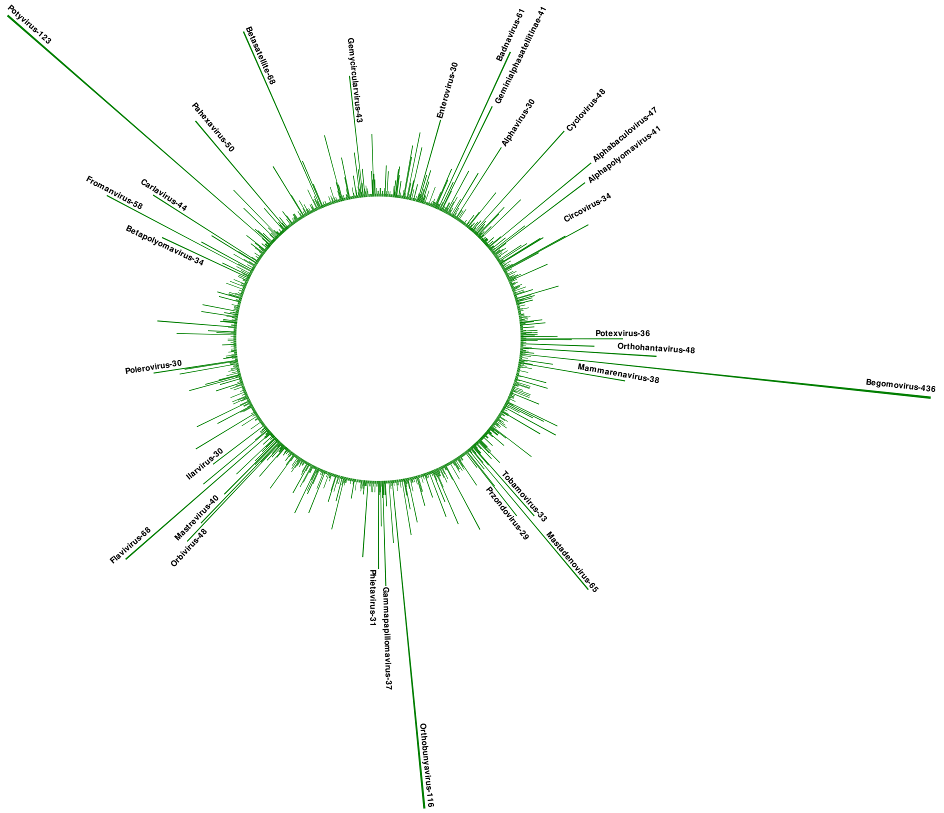

Figure S4. Frequency of genome sequences per viral genus.

Table S9. F1-score (F1) results obtained for viral taxonomic classification tasks regarding genome type, realm, kingdom, phylum, class, order, family, and genus. The classifiers used were Linear Discriminant Analysis (LDA), Gaussian Naive Bayes (GNB), K-Nearest Neighbors (KNN), Support Vector Machine (SVM), and XGBoost classifier (XGB).

Classification	N. Classes	N. Samples	$F1_{LDA}$	$F1_{GNB}$	$F1_{SVM}$	$F1_{KNN}$	$F1_{XGB}$
**Genome**	5	6089	0.6549	0.736	0.6989	0.836	**0.8662**
**Realm**	5	5799	0.7496	0.8001	0.7949	0.8817	**0.9234**
**Kingdom**	10	5788	0.7238	0.7640	0.7512	0.8410	**0.9039**
**Phylum**	17	5778	0.5824	0.5226	0.4435	0.6891	**0.8299**
**Class**	34	5845	0.4780	0.4562	0.3803	0.5896	**0.7963**
**Order**	48	5838	0.4435	0.4798	0.3832	0.5462	**0.7884**
**Family**	102	5990	0.3042	0.3517	0.1681	0.3429	**0.7323**
**Genus**	360	4673	0.3600	0.2956	0.0682	0.0621	**0.6561**

Table S10. F1-score (F1) obtained for the viral taxonomic classification task regarding genome type, realm, kingdom, phylum, class, order, family, and genus. The features used were the genome's sequence length (SL), the GC-content (GC) and the Normalized Compression (NC) values for the best model, the same model with IR configuration to 0, 1 and 2.

Classification	N. Classes	N. Samples	$F1_{NC}$	$F1_{NC+GC}$	$F1_{NC+SL+GC}$	$F1_{All\ without\ SQ}$	$F1_{AllFeatures}$
**Genome**	5	6089	0.7490	0.7988	0.8649	0.8051	**0.8662**
**Realm**	5	5799	0.7726	0.8401	0.9200	0.8569	**0.9234**
**Kingdom**	10	5788	0.7518	0.8131	0.9026	0.8295	**0.9039**
**Phylum**	17	5778	0.6234	0.6926	0.8194	0.7188	**0.8299**
**Class**	34	5845	0.5742	0.6404	0.7844	0.6705	**0.7963**
**Order**	48	5838	0.5568	0.6292	0.7736	0.6598	**0.7884**
**Family**	102	5990	0.4112	0.5187	0.7118	0.5636	**0.7323**
**Genus**	360	4673	0.3248	0.4661	0.6417	0.5089	**0.6561**

Software and Hardware recommendations

The experiences of the manuscript can be replicated using a laptop, desktop, or server computer running Arch linux or Linux Ubuntu (for example, 18.04 LTS or higher) with GCC (`https://gcc.gnu.org`), git and git LFS, Conda (`https://docs.conda.io`) and python version 3.6. The hardware must contain at least 8 GB of RAM and a 100 GB disk.

Reproducibility

Creating Project and intalling tools

The descriptions of reproducion is depicted bellow, for more detail see https://github.com/jorgeMFS/canvas. Install Git LFS:

```
mkdir -p gitLFS
cd gitLFS/
wget https://github.com/git-lfs/git-lfs/releases/download/v2.9.0/git-lfs-linux-amd64-v2.9.0.tar.gz
tar -xf git-lfs-linux-amd64-v2.9.0.tar.gz
chmod 755 install.sh
sudo ./install.sh
```

Get CANVAS project, create the docker and run it:

```
git clone https://github.com/jorgeMFS/canvas.git
cd canvas
docker-compose build
docker-compose up -d && docker exec -it canvas bash && docker-compose down
```

Inside the docker, give run permissions to the files and install tools using :

```
chmod +x *.sh
bash Make.sh;
```

Replication of the Results

The code was created in order to allow independent replication and reproduction of each step, this was done due to the extensive processing time required to filter and rearrange viral DB and extract the features and taxonomic information of each viral sequence. If you wish to rebuild database and feature reports extracted, see the Database reconstruction subsection.

To obtain the Human Herpesvirus, plot run:

```
cd python || exit;
python compare_cmix_hhv.py
```

To obtain the Compression Benchmark plots, run:

```
cd python || exit;
python select_best_nc_model.py;
```

To perform the synthetic sequence test, run:

```
cd scripts || exit;
bash Stx_seq_test.sh;
```

To perform classification, run the following code:

```
cd python || exit;
python prepare_classification.py; #recreate classification dataset
python classifier.py; #perform classifications
```

To perform the complete IR analysis and create:

- boxplots;
- 2d scatter plots;
- 3d scatter plots;
- top taxonomic group lists;
- Occurrence of each Genus.

Execute this code:

```
cd python || exit;
python ir_analysis.py; # Performs complete IR analysis
```

To perform the Human Herpesvirus analysis and obtain the plots, run:

```
cd scripts || exit;
bash Herpesvirales.sh;
```

Database reconstruction

To run the pipeline and obtain all the Reports in the folder reports, use the following commands. Note that if you wish to recreate the features reports, you must perform the database reconstruction task first.

If you wish to reconstruct the viral database, run the following script:

```
cd scripts || exit;
bash Build_DB.sh;
```

To create the features for analysis and classification (very time consuming, can take several days), run:

```
cd scripts || exit;
bash Process_features.sh;
```

To recreate the compression reports used for benchmark (very time consuming, can take several hours), run:

```
cd scripts || exit;
bash Compress.sh;
```

Cladograms

The Cladograms require GUI application. As such, the reproduction of the cladograms has to be performed outside of the docker on the Ubuntu system on the /canvas folder:

```
chmod +x *.sh
bash so_dependencies.sh #install Ubuntu system dependencies required for the script to run and Anaconda
conda create -n canvas python=3.6
conda activate canvas
bash Make.sh #install python libs
bash Install_programs.sh #install tools using conda
```

Afterwards, to obtain the Cladogram plots, run:

```
cd python || exit;
python phylo_tree.py;
```

References

1. Strauss JH, Strauss EG. CHAPTER 1 - Overview of Viruses and Virus Infection. In: Strauss JH, Strauss EG, editors. Viruses and Human Disease (Second Edition), second edition ed. London: Academic Press; 2008.p. 1–33.
2. Lidmar J, Mirny L, Nelson DR. Virus shapes and buckling transitions in spherical shells. Physical Review E 2003;68(5):051910.
3. Vernizzi G, de la Cruz MO. Faceting ionic shells into icosahedra via electrostatics. Proceedings of the National Academy of Sciences 2007;104(47):18382–18386.
4. Luque A, Reguera D. The structure of elongated viral capsids. Biophysical journal 2010;98(12):2993–3003.
5. Tsagris EM, Martínez de Alba ÁE, Gozmanova M, Kalantidis K. Viroids. Cellular microbiology 2008;10(11):2168–2179.
6. Krupovic M, Cvirkaite-Krupovic V. Virophages or satellite viruses? Nature Reviews Microbiology 2011;9(11):762–763.
7. Dimmock NJ, Easton AJ, Leppard KN. Introduction to modern virology. John Wiley & Sons; 2016.
8. Gago S, Elena SF, Flores R, Sanjuán R. Extremely high mutation rate of a hammerhead viroid. Science 2009;323(5919):1308–1308.
9. Simón D, Cristina J, Musto H. Nucleotide composition and codon usage across viruses and their respective hosts. Frontiers in Microbiology 2021;12.
10. Baltimore D. Expression of animal virus genomes. Bacteriological reviews 1971;35(3):235–241.
11. Peck KM, Lauring AS. Complexities of viral mutation rates. Journal of virology 2018;92(14):e01031–17.
